# Changes in concentrations of cervicovaginal immune mediators across the menstrual cycle: a systematic review and meta-analysis of individual patient data

**DOI:** 10.1186/s12916-022-02532-9

**Published:** 2022-10-05

**Authors:** Sean M. Hughes, Claire N. Levy, Ronit Katz, Erica M. Lokken, Melis N. Anahtar, Melissa Barousse Hall, Frideborg Bradley, Philip E. Castle, Valerie Cortez, Gustavo F. Doncel, Raina Fichorova, Paul L. Fidel, Keith R. Fowke, Suzanna C. Francis, Mimi Ghosh, Loris Y. Hwang, Mariel Jais, Vicky Jespers, Vineet Joag, Rupert Kaul, Jordan Kyongo, Timothy Lahey, Huiying Li, Julia Makinde, Lyle R. McKinnon, Anna-Barbara Moscicki, Richard M. Novak, Mickey V. Patel, Intira Sriprasert, Andrea R. Thurman, Sergey Yegorov, Nelly Rwamba Mugo, Alison C. Roxby, Elizabeth Micks, Florian Hladik, The Consortium for Assessing Immunity Across the Menstrual Cycle, Salim S. Abdool Karim, Max Abou, Sharon M. Anderson, Aura Andreasen, Trong T. Ao, David F. Archer, Kevin K. Arien, Kelly B. Arnold, Susana Asin, Susan Baden, Bernard S. Bagaya, Kathy Baisley, Emma Barnard, Angela Bartolf, Brian A. Bernick, Kenzie Birse, Andrea K. Boggild, Genevieve Boily-Larouche, Lucy A. Boksa, Brittany A. Bowman, Fredrick P. Bowman, Kristina Broliden, Adam D. Burgener, Jozefien Buyze, Elizabeth H. Byrne, Neelima Chandra, Stacey Chapman, Hua Yun Chen, Juliana Cheruiyot, Ralph R. Chesson, Kathleen E. Cohen, Piet Cools, Catherine Cosgrove, Gary R. Coulton, Peggy A. Crowley-Nowick, Tania Crucitti, Tina D. Cunningham, Susan Cu-Uvin, Hassan Y. Dawood, Sinead Delany-Moretlwe, Krista L. Dong, Betty A. Donoval, Brenden Dufault, Kathleen Dunlap, Laura J. Dunphy, Robert P. Edwards, Lars Engstrand, Terri Espinosa, John V. Fahey, Titilayo Fashemi, J. Dennis Fortenberry, Jamie L. Freiermuth, Ronald M. Galiwango, Musie S. Ghebremichael, Sara V. Good, Odin Goovaerts, Parrie J. Graham, Liselotte Hardy, Klara Hasselrot, Richard J. Hayes, Betsy C. Herold, Carolina Herrera, Ronald C. Hershow, Allan Hildesheim, Sharon Hillier, Yanwen Hou, Hazel Huang, Sean M. Hughes, Loris Y. Hwang, Andrea Introini, Nasreen Ismail, Terry Jacot, Mariel Jais, Vicky Jespers, Vineet Joag, Christine Johnston, Clifford Jones, Sarah Joseph, Saidi Kapiga, John C. Kappes, Joshua Kimani, Makobu Kimani, Thomas Kimble, Noah Kiwanuka, Monika Kowatsch, Jessie Kwatampora, Douglas S. Kwon, Julie Lajoie, Alan Landay, Douglas A. Lauffenburger, Dara A. Lehman, Alasdair Leslie, Lenine J. Liebenberg, Jay A. Lieberman, Vitali Lounev, Yifei Ma, Amanda Mabhula, Jennifer Mabuka, Kaballa Maganja, Jeanne Marrazzo, Lindi Masson, Kenneth H. Mayer, Stuart McCorrister, Joris Menten, Pedro M. M. Mesquita, Johan Michiels, Sebastian Mirkin, Amber Moodley, Juliet Mpendo, Lucy R. Mukura, Mary Mwaura, Gilles Ndayisaba, Thumbi Ndung’u, Jane Njoki, Laura Noel-Romas, Billy Nyanga, Christina Ochsenbauer, Katherine Odem-Davis, Gregory S. Olson, Kenneth Omollo, Donald P. Orr, Julie Overbaugh, Julius Oyugi, Nikita Padavattan, Tarita Pakrashi, Urvashi Pandey, Jo-Ann S. Passmore, Terri Pustilnik, Lorna Rabe, Nicola Richardson-Harman, Christiane Rollenhagen, Laura Romas, Richard M. Rossoll, Jill L. Schwartz, Mark E. Scott, Maike Seifert, A. Shah, Kamnoosh Shahabi, Robin J. Shattock, Zheng Shen, Baochen Shi, Sengeziwe Sibeko, Yan Song, Gregory Spear, Brian S. Starkman, Howard D. Strickler, Jan L. Sumerel, Egbert Tannich, Katherine P. Theall, Annelie Tjernlund, Janneke van de Wijgert, Barbara Van Der Pol, Guido Vanham, Bruce D. Walker, Joan L. Walker, Deborah Watson-Jones, Hugo Wefer, Garrett R. Westmacott, Charles R. Wira, Peter F. Wright, Naji Younes, Nazita Yousefieh

**Affiliations:** 1 Department of Obstetrics and Gynecology, University of Washington, Seattle, WA USA; 2 Department of Global Health, University of Washington, Seattle, WA USA; 3 Ragon Institute of MIT and Harvard, Massachusetts General Hospital, Boston, MA USA; 4 University of Louisville, Louisville, KY USA; 5 Department of Medicine, Solna, Karolinska Institutet, Karolinska University Hospital, Stockholm, Sweden; 6 Division of Cancer Prevention, National Cancer Institute, National Institutes of Health, Rockville, MD USA; 7 Division of Cancer Epidemiology and Genetics, National Cancer Institute, National Institutes of Health, Rockville, MD USA; 8 Department of Molecular, Cell & Developmental Biology, University of California, Santa Cruz, Santa Cruz, CA USA; 9 CONRAD, Eastern Virginia Medical School, Norfolk, VA USA; 10 Department of Obstetrics, Gynecology and Reproductive Biology, Harvard Medical School, Brigham and Women’s Hospital, Boston, MA USA; 11 Louisiana State University Health, New Orleans, LA USA; 12 Department of Medical Microbiology & Infectious Diseases, University of Manitoba, Winnipeg, Manitoba Canada; 13 MRC International Statistics and Epidemiology Group, London School of Hygiene and Tropical Medicine, London, UK; 14 Department of Epidemiology, The George Washington University, Washington, DC USA; 15 Department of Pediatrics, University of California, Los Angeles, Los Angeles, CA USA; 16 Office of Laboratory Safety, The George Washington University, Washington, DC USA; 17 Institute of Tropical Medicine, Antwerp, Belgium; 18 Department of Microbiology and Immunology, University of Minnesota, Minneapolis, MN USA; 19 Department of Medicine, University of Toronto, Toronto, Ontario Canada; 20 Virology Unit, Department of Biomedical Sciences, Institute of Tropical Medicine, Antwerp, Belgium; 21 University of Vermont Larner College of Medicine, Burlington, VT USA; 22 Department of Molecular and Medical Pharmacology, Crump Institute for Molecular Imaging, University of California, Los Angeles, Los Angeles, CA USA; 23 IAVI Human Immunology Laboratory, Imperial College, London, England, UK; 24 IAVI, New York, NY USA; 25 Centre for the AIDS Programme of Research in South Africa (CAPRISA), Durban, South Africa; 26 Department of Medical Microbiology and Immunology, University of Nairobi, Nairobi, Kenya; 27 University of Illinois, Chicago, IL USA; 28 Geisel School of Medicine at Dartmouth, Lebanon, NH USA; 29 Department of OB/GYN, Keck School of Medicine, University of Southern California, Los Angeles, CA USA; 30 Department of Biochemistry and Biomedical Sciences, McMaster University, Hamilton, Ontario Canada; 31 Center for Clinical Research, Kenya Medical Research Institute, Nairobi, Kenya; 32 Department of Medicine, University of Washington, Seattle, WA USA; 33 Vaccine and Infectious Disease Division, Fred Hutch, Seattle, WA USA

**Keywords:** Menstrual cycle, Cytokine, Chemokine, Cervix, vagina, Female genital tract, Systematic review, Meta-analysis

## Abstract

**Background:**

Hormonal changes during the menstrual cycle play a key role in shaping immunity in the cervicovaginal tract. Cervicovaginal fluid contains cytokines, chemokines, immunoglobulins, and other immune mediators. Many studies have shown that the concentrations of these immune mediators change throughout the menstrual cycle, but the studies have often shown inconsistent results. Our understanding of immunological correlates of the menstrual cycle remains limited and could be improved by meta-analysis of the available evidence.

**Methods:**

We performed a systematic review and meta-analysis of cervicovaginal immune mediator concentrations throughout the menstrual cycle using individual participant data. Study eligibility included strict definitions of the cycle phase (by progesterone or days since the last menstrual period) and no use of hormonal contraception or intrauterine devices. We performed random-effects meta-analyses using inverse-variance pooling to estimate concentration differences between the follicular and luteal phases. In addition, we performed a new laboratory study, measuring select immune mediators in cervicovaginal lavage samples.

**Results:**

We screened 1570 abstracts and identified 71 eligible studies. We analyzed data from 31 studies, encompassing 39,589 concentration measurements of 77 immune mediators made on 2112 samples from 871 participants. Meta-analyses were performed on 53 immune mediators.

Antibodies, CC-type chemokines, MMPs, IL-6, IL-16, IL-1RA, G-CSF, GNLY, and ICAM1 were lower in the luteal phase than the follicular phase. Only IL-1α, HBD-2, and HBD-3 were elevated in the luteal phase. There was minimal change between the phases for CXCL8, 9, and 10, interferons, TNF, SLPI, elafin, lysozyme, lactoferrin, and interleukins 1β, 2, 10, 12, 13, and 17A. The GRADE strength of evidence was moderate to high for all immune mediators listed here.

**Conclusions:**

Despite the variability of cervicovaginal immune mediator measurements, our meta-analyses show clear and consistent changes during the menstrual cycle. Many immune mediators were lower in the luteal phase, including chemokines, antibodies, matrix metalloproteinases, and several interleukins. Only interleukin-1α and beta-defensins were higher in the luteal phase. These cyclical differences may have consequences for immunity, susceptibility to infection, and fertility. Our study emphasizes the need to control for the effect of the menstrual cycle on immune mediators in future studies.

**Supplementary Information:**

The online version contains supplementary material available at 10.1186/s12916-022-02532-9.

## Background

### Rationale

It is important to understand immunity in the cervicovaginal tract (CVT) given its key role in pathogen entry for sexually transmitted infections (STIs). A clear understanding of CVT biology is crucial for intervention studies with immune outcomes (such as HIV pre-exposure prophylaxis, treatment of bacterial vaginosis, and mucosal vaccination). In addition, understanding the immune consequences of new forms of hormonal contraception requires understanding this natural baseline.

The menstrual cycle has important effects on CVT immunity. The follicular or proliferative phase of the menstrual cycle starts on the first day of menstrual bleeding and is characterized by increasing estradiol and low progesterone. The luteal or secretory phase of the cycle begins following ovulation and is characterized by high progesterone. Multiple studies suggest that immunity changes in the CVT across the menstrual cycle, but it is unclear whether STI risk peaks at a particular stage of the menstrual cycle. One hypothesis holds that the luteal phase represents a “window of vulnerability” to STIs, where immunity is suppressed to allow tolerance of a possible embryoblast [1]. This hypothesis, while plausible, remains unproven, with evidence mainly from studies of non-human primates [2–4] and from conflicting human studies [5–7].

Many published studies describe how immune mediators (cytokines, chemokines, immunoglobulins, and other factors) in the CVT change during the menstrual cycle [8–33]. Despite this abundance of studies, our knowledge of the immunological impact of the menstrual cycle remains somewhat lacking and could be improved by a systematic compilation of results from all studies. Moreover, for some immune mediators, data interpretation is complicated at times by conflicting results between studies. For example, four studies have observed higher interleukin 6 (IL-6) concentrations during the follicular phase [19, 21, 23, 26], while two other studies have observed higher IL-6 concentrations in the luteal phase [11, 12].

One reason for the variability observed in studies of immune mediators in the CVT may be the diversity of the experimental approaches used to collect and measure immune mediators. Sample types include cervicovaginal lavage (CVL), menstrual cup, brush, and swab. Assay types include ELISA, bead-based platforms (such as Luminex), and other antibody-based techniques. Menstrual cycle phase has been determined by the date of last menstrual period and by serum or urine hormone levels. Outcomes include raw immune mediator concentrations or levels normalized to total protein. Determining which of these approaches to specimen collection and testing best capture the underlying biological changes would be of benefit to future studies.

To address these important gaps, we performed a systematic review and meta-analysis of individual participant data (IPD) of immune mediators in the CVT during the menstrual cycle. The primary objective of this study was to estimate differences in concentrations of immune mediators between the follicular and luteal phases of the menstrual cycle. The secondary objectives of this study were to compare how four technical factors (sample type, assay type, method of determining menstrual cycle phase, and normalization of immune mediator concentrations to total protein) influence the results and affect our conclusions about the changes that occur throughout the menstrual cycle.

In addition to summarizing previous studies, we performed a new study of 200 paired cervicovaginal lavage samples from the follicular and luteal phases. This study had an exploratory component, where we measured immune mediators included in only few previous studies, and a validation component, where we specifically tested immune mediators estimated by the meta-analysis to differ across the menstrual cycle. By performing this additional study, we confirmed the accuracy of the meta-analysis and broadened our knowledge of immune changes across the menstrual cycle.

## Methods

### Protocol for systematic review and meta-analysis

This methods section constitutes a protocol for a systematic review and meta-analysis. This protocol was drafted in advance of performing the review and submitted as a registered report. At the time of submission (July 2020), tests of the search strategy and of the abstract and manuscript screening systems had been performed, but formal abstract screening had not begun. Prior to drafting the protocol, we performed a pilot meta-analysis with data obtained from several studies [10–12, 15, 19, 21, 23, 26]. These studies were screened in the same way as all other search results.

This protocol is in compliance with the Preferred Reporting Items for Systematic Review and Meta-Analysis Protocols (PRISMA-P) guidelines [34]. The final manuscript complies with the Preferred Reporting Items for a Systematic Review and Meta-Analysis of Individual Participant Data (PRISMA-IPD) guidelines [35]. Completed checklists are available in Additional file 1. The overall study design is shown in Table 1.

**Table 1:** Study design

Stage	Step
1	Protocol development
2	Database searches
3	Screen abstract search results
4	Screen full-text manuscripts against eligibility criteria
5	Data extraction, risk of bias assessment, request IPD
6	Database searches with updated terms
7	Individual study analysis
8	Interim meta-analysis
9	Choose immune mediators for exploratory and validation study
10	Wet lab: Perform exploratory and validation study
11	Incorporate exploratory and validation study results into final meta-analysis
12	Grade strength of evidence

While performing the study, we needed to amend this protocol. In Additional file 2, we gave the date of each amendment, described the change, and gave the rationale. Changes were not incorporated into this methods section.

### Eligibility criteria

#### Study eligibility criteria

We included studies reporting original data on any immune mediator concentrations by menstrual cycle phase (determined by date of last menstrual period [LMP] or hormone levels, including progesterone, estradiol, and/or luteinizing hormone) in CVT samples from menstruating women. Immune mediators were defined as immune-related proteins, including cytokines, chemokines, immunoglobulins, antimicrobial peptides, and growth factors. We only included studies that measured concentrations using antibody-based methods (such as ELISAs, Luminex and other bead-based assays, and MSD assays). We did not include studies using other methods, such as gene expression or mass spectrometry-based proteomics or metabolomics. CVT samples were defined as secretions or fluid, such as CVL, menstrual cup, or swab. We included unpublished studies that met our eligibility criteria.

#### Participant eligibility criteria

Participant-level eligibility criteria allowed us to include subsets of participants from studies where only some subjects were eligible (such as studies comparing pre- and post-menopausal women, where only the pre-menopausal women were included). Eligible participants were post-menarche, pre-menopausal, non-pregnant women not using hormonal contraception or an intrauterine device (IUD) and not receiving other exogenous hormones. Because intra-study comparisons of follicular and luteal phases were performed, each study had to have both follicular and luteal phase samples, but single samples from individual participants were eligible. We excluded participants who received a vaginal intervention (including placebo), but participants receiving no treatment or a systemic placebo were eligible. Baseline, pre-intervention visits were acceptable (such as if all participants had baseline visits, a cross-sectional analysis could be performed). Samples from women with cervical or vaginal pathology, such as bacterial vaginosis, vulvovaginal candidiasis, STIs, or cervical dysplasia, were eligible. We chose to include such samples because cervical or vaginal pathology is a normal part of life for most women at some point. In addition, we expected pathology to have no association with cycle phase (for example, we expected BV to be equally common in both phases of the cycle), so it would not confound our menstrual cycle analysis.

### Information sources

We searched PubMed, Web of Science, Embase, and the Global Health Database for articles and conference abstracts published in English since 2000 (inclusive). We also reviewed the bibliographies of included studies and relevant reviews to identify additional studies. As recommended in chapter 4 of the Cochrane Handbook for Systematic Reviews of Interventions, we circulated our list of included studies to authors when requesting individual participant data and asked for recommendations of additional studies, whether published or unpublished [36].

### Search strategy

Complete search strategies are listed in Additional file 1. These search strategies were designed in advance of performing the study. We planned in advance that we could update the search strategies during the course of the study: specifically, if we found published studies through review of bibliographies or author recommendations that were not captured by our search strategy. In that case, we could update the search terms near the completion of this project so that the search would capture most of these additional studies as well. We would then screen all additional results found by the updated strategy.

### Study records

#### Data management and selection process

Search results were de-duplicated using PubMed IDs, the text of the titles and abstracts, and manual review of duplicate DOIs. Abstracts were loaded into abstrackr [37] for screening. Two reviewers (CNL and SMH) independently screened all abstracts for eligibility. We obtained the full text of all articles identified as potentially eligible by either reviewer. Both reviewers independently reviewed full texts, guided by a Google Forms questionnaire (Additional file 1) to determine eligibility and record study information. We recorded reasons for exclusion of a study in the questionnaire. Differences in opinion were resolved by discussion. If the two reviewers were unable to agree, a third study author (FH) made the final decision. If conference abstracts appeared to meet inclusion criteria, but could not be linked to a publication, we contacted the authors to locate the publication. We attempted to extract summary data from all studies using the Google Forms questionnaire (Additional file 1). Specifically, if available, we extracted estimates of the difference in concentrations of each immune mediator between the follicular and luteal phases, as well as the statistical methodology used to generate that estimate. We anticipated that this summary data would be unavailable from many manuscripts.

#### Data collection process and individual participant data integrity

We requested individual participant data (IPD) from study authors via email, following up at least three times. We accepted data in any format provided. After receipt of IPD, we prepared a data summary document (including the number of samples, number of immune mediators, menstrual phase, covariate summaries, the number of samples below LOD, and the immune mediator means and 95% CIs). We sent this summary document to the study authors and requested that they confirm that we received the complete and correct data. We also compared the IPD we received and the results of our analyses to published reports, where available, to confirm that the data we received was correct.

If we were unable to obtain IPD for a particular study, we recorded the reasons that prevented obtaining the data and attempted to extract IPD from the published article. Two reviewers independently extracted the data and discussed differences, with a third reviewer resolving discrepant results and disagreements when necessary. Data were extracted from published figures using software such as WebPlotDigitizer [38], if appropriate.

If IPD was unavailable from the authors and could not be extracted from the published article, we recorded the reasons that prevented obtaining the data. If summary data was available (differences between follicular and luteal phases, extracted for all studies as described above) and matched the study-level analyses described below, we included the study at the meta-analysis level in the two-stage approach described below. For papers where only quantile statistics were reported, we obtained means and standard deviations (necessary for meta-analysis) using previously devised methods [39–42].

#### Data items

We collected the following study-level data items:

- Method of determination of menstrual phase (date of last menstrual period or hormone levels including sample type and specific hormones measured)
- Sample type (cervicovaginal lavage [including clinician- or participant-collected, volume, and lavage buffer], swab [ectocervical, endocervical, or vaginal], menstrual cup, other)
- Country or countries of clinical sites (grouped into the geographical region)

We collected the following sample-level data items:

- Immune mediator concentrations (pg/mL)
- Menstrual phase (luteal/secretory, follicular/proliferative, periovulatory)
- Additional covariates (when collected): total protein concentrations, age, bacterial vaginosis status, vulvovaginal candidiasis status, sexually transmitted infection status (including gonorrhea, chlamydia, trichomoniasis, herpes simplex virus, HIV), race/ethnicity, recent sexual contact, condom use, vaginal pH, hemoglobin contamination, and any other available covariates from each study.

We collected the following immune mediator-level data items:

- Assay method (ELISA, bead-based [e.g., Luminex], MSD, possibly others)
- Lower limits of detection

#### Data standardization

The definition of menstrual phase was standardized across studies and based on either serum progesterone level, days since luteinizing hormone (LH) surge, or days since the start of the last menstrual period (LMP). If multiple measures were available, we defined the menstrual phase based on hormone levels. For serum progesterone, the follicular phase was defined as serum progesterone < 1 ng/mL, and luteal was defined as serum progesterone ≥ 3 ng/mL. We chose these criteria based on a study [43] showing that the vast majority of pre-ovulatory samples have progesterone levels below 1 ng/mL and the vast majority of post-ovulatory samples have progesterone levels above 3 ng/mL. We excluded samples falling in the 1–3 ng/mL window, because these typically occur beginning on the day of the luteinizing hormone peak and ending two days after. For studies reporting LH surge without progesterone levels, follicular was defined as after menses and prior to LH surge, while luteal was defined as 2–12 days following LH surge. For studies reporting LMP, we only included participants reporting regular menstrual cycles. Follicular phase included days 5–12 (inclusive) since the start of the last menstrual period, and luteal phase included days 19–24 (inclusive) since the start of the last menstrual period. In some circumstances, decisions about sample inclusion were made on a case-by-case basis by discussion between two reviewers. The circumstances could include (1) samples falling outside the windows for days since the last menstrual period, LH surge, or progesterone concentration; (2) studies where hormone concentrations or days since LMP were used to determine menstrual phase, but those data are no longer available; or (3) studies where menstrual phase was determined by another method, such as urinary progesterone metabolite concentration.

We included periovulatory samples as a third phase, with this phase defined by LH levels above 20 mIU/mL in serum [44] or 25 mIU/mL in urine [45].

All additional variables were standardized across studies to the extent possible, based on the data. We defined assay type, sample type, and method of determination of menstrual phase as described above. We treated swabs from different anatomic sites (ectocervical, endocervical, vaginal) as different sample types. CVLs were considered a single sample type, but differences in methods of collection were explored in sensitivity analysis as described below. We assigned consistent cross-study definitions to additional covariates as much as possible based on the data collected. For example, for bacterial vaginosis (BV), if one study reported Nugent scores and another study reported BV based on Amsel criteria, we converted these variables into a single variable for BV, with values of positive, indeterminate, and negative.

If the limits of detection were unavailable, we attempted to obtain the information from the manufacturer of the assay. If the limits of detection were not available from the manufacturer, we classified the values as follows: undetectable when two or more samples have the lowest reported concentration for a given immune mediator in a particular study. Otherwise, samples were classified as detectable.

#### Outcomes and prioritization

##### Primary outcome

For immune mediators that were detectable in ≥ 50% of samples, the outcome was the difference in mean log2 concentration between the follicular and luteal phases. For immune mediators detectable in < 50% of samples, the outcome was risk ratio of detection between the follicular and luteal phases, with risk defined as the number of samples in which the immune mediator was detected out of the total number of samples. In addition, we compared periovulatory samples to follicular and luteal phase samples.

##### Secondary outcomes

- For sample type and assay type, the outcomes were effect size for concentration and detectability (higher concentrations and levels of detectability were considered superior) from meta-regression. A second outcome was the standard error of the menstrual cycle effect sizes from subgroup analysis (lower standard errors were considered superior).
- For menstrual phasing method and normalization to total protein, the outcomes were within-study comparisons of the standard error of the menstrual cycle effect sizes (lower standard errors were considered superior). For menstrual phasing method, we also assessed misclassification rates from studies that reported both days since last menstrual period and hormone levels.

#### Risk of bias of individual studies

We assessed the risk of bias in each study using a custom tool adapted from the Newcastle Ottawa scale (Additional file 1). This information was used in determining the strength of evidence.

### Data analysis

#### Criteria for quantitative synthesis

We performed meta-analysis for all immune mediators present in at least two included studies. Data analysis was performed using R version 4.0.0.

#### Data handling, combination, and summary measures

Data processing: Sample wells falling below the lower limit of detection were assigned a value of the study-specific lower limit of detection divided by 2. Wells falling above the upper limit of detection were assigned a value of the study-specific upper limit of detection multiplied by 2. If replicate wells were run for a given sample, the raw concentrations were averaged. Data was then log2-transformed. Each sample was also scored as “detectable” or “non-detectable”, with the sample counting as detectable if it was detected in at least one well.

#### Primary outcome analysis plan

We used a two-stage approach for meta-analysis: first analyzing each study separately and then combining the summary statistics from each study to generate meta-estimates of effect. We chose this approach to allow inclusion of studies where summary data was available but IPD was not.

- Study level: We fit a separate linear mixed-effects model for each immune mediator, with participant as a random effect and menstrual phase as a fixed effect. The primary analysis was unadjusted. For immune mediators that were detectable in ≥ 50% of samples, the model outcome was the difference in mean log2 concentration between the follicular and luteal phases. For immune mediators detectable in < 50% of samples, mixed logistic models were used to compare the risk of detection (i.e., likelihood of detection) between the follicular and luteal phases using a risk ratio. Specifically, risk of detection was defined as the number of samples in which the immune mediator was detected out of the total number of samples.
- Meta-analysis level: We performed random effects meta-analysis using inverse-variance pooling to estimate the pooled mean difference in log2 concentrations of each immune mediator between the follicular and luteal phases. We reported meta-effect sizes and their 95% CIs and displayed forest plots. We reported raw *p*-values as well as *p*-values adjusted for the number of immune mediators with the Holm and false discovery rate methods. We reported two analyses: an unadjusted analysis and an analysis adjusted by meta-regression for assay type, sample type, method of determining menstrual phase, and geographical region.

#### Secondary outcome analysis plan

For assay type and sample type, we performed meta-regression after the two-stage approach described above. In addition, we performed subgroup analysis stratifying by each covariate (assay type, sample type) and compared the standard error of the menstrual cycle effect sizes.

For the method of the menstrual phase, we analyzed studies that reported both hormone levels and days since the first day of LMP. For those studies, we performed the menstrual cycle analysis separately using each method of determining the menstrual phase. We then compared the standard errors within study.

For normalization to total protein, we only used data from studies reporting total protein concentrations. We performed the menstrual cycle analysis separately on the raw immune mediator concentrations and on the immune mediator concentrations normalized to total protein. We then compared the standard errors within each study.

#### Exploration of variation in effects

We reported *χ*^2^ tests and the *I*^2^ statistic to summarize between-study heterogeneity in the menstrual cycle effect. For immune mediators with high levels of heterogeneity (*I*^2^ > 75%), we attempted to explain the heterogeneity through subgroup or sensitivity analysis.

Sensitivity Analyses: The goal of the sensitivity analyses was to determine how robust the results were to analytic assumptions. We compared the results of several alternative analyses to the primary analysis described above.

- Sample-level covariates: Because the available participant-level covariates differed between studies, our primary study-level analysis did not include any fixed effects except for the menstrual phase. Here, we repeated the study-level analyses and included all relevant covariates for each study. We then performed a meta-analysis on the effect of the menstrual cycle phase as estimated in these models and compared the results to our primary analysis.
- One-stage vs. two-stage meta-analysis: Rather than analyze each study separately, we pooled the raw data from all studies and assessed the effect of the menstrual phase in a single model per immune mediator, with participant and study as random effects.
- Variation in CVL methods: We compared different methods of obtaining CVLs, including participant- vs. clinician-collected sample, lavage volume, and lavage medium. It was difficult to predict in advance how many studies would be available in each category, so we grouped CVL methods into categories once we collected the studies. The outcomes were effect size for concentration and detectability (higher concentrations and levels of detectability were be considered superior) from meta-regression.

#### Alternative to quantitative synthesis

Immune mediators measured in only one study or that could not be included in the meta-analysis for any other reason were listed as areas for further research.

### Data integrity and evidence strength

#### Meta-biases

We assessed publication bias and selective outcome reporting. We attempted to limit bias due to selective outcome reporting by requesting IPD for all immune mediators measured, regardless of which were reported in published studies. To attempt to limit publication bias, we sought out unpublished studies by requesting them from authors who contributed IPD from published studies and by including conference abstracts in our search strategy. To assess publication bias, we reported Egger’s test and funnel plots for immune mediators where ten or more studies existed.

#### IPD integrity

If any issues with study data were uncovered when we checked the IPD, we reported these issues and any corrective actions taken.

#### Assessment of strength of the body of evidence

We assessed the strength of the body of evidence using the GRADE methodology [46], with the instrument shown in Additional file 1. Two reviewers (CNL and SMH) performed the assessments independently and then came to a consensus, with disagreements resolved by a third author (FH).

We assessed the strength of the body of evidence for each immune mediator in five domains (risk of bias, inconsistency, indirectness, imprecision, and publication bias), each of which could lead to downgrading of the strength of evidence. We also assessed domains which could lead to upgrading of the strength of evidence, including large magnitude of effect (defining large as 5-fold and very large as 10-fold) and residual confounding that would be likely to strengthen the observed effect (or lack thereof). Randomization and dose responses were not be taken into account as they are not relevant for these studies (participants cannot be randomized to a particular phase of the cycle and dose is irrelevant for the cycle).

We assigned an overall strength of evidence score to each immune mediator based on a four-star scale as follows: high (further research is unlikely to change our confidence or the estimate of the effect), moderate (further research may change our confidence and the estimate of the effect), low (further research will likely change our confidence and the estimate of the effect), and very low (further research will very likely change our confidence and the estimate of the effect).

### Additional wet lab assays

#### Sample cohort

As part of this review and meta-analysis, we performed one additional study including an exploratory and a validation component. We used CVL samples from the Kenya Girls Study, a longitudinal cohort study of adolescent girls followed for acquisition of sexually transmitted infections [47]. We chose samples using the following requirements: no use of hormonal contraception, at least one follicular and one luteal phase sample available from the same participant (based on the date of LMP), STI testing and Nugent scoring for BV performed, and non-intermediate vaginal flora (Nugent score either 0–3 or 7–10). We measured serum progesterone to assign samples to the follicular or luteal phase. We measured total protein concentrations in CVL samples. Because sexual activity and exposure to semen may affect CVT immunity, we measured kallikrein-3 (also known as prostate-specific antigen). Similarly, blood contamination of the samples may influence immune mediator concentrations, so we measured hemoglobin. The sample size was designed to be approximately 200 samples from approximately 100 women. This size was determined based on feasibility and cost. All participants provided written, informed consent in the Kenya Girls Study as described in the main manuscript for that study [47]. Only deidentified samples were used as part of this study.

#### Exploratory study

The purpose of the exploratory component of the study was to increase the strength of evidence for immune mediators that were measured in only few studies. We selected the mediators to be measured after we obtained data from all studies for meta-analysis. We chose approximately ten immune mediators that were measured in only 1–2 studies, with the total number of immune mediators determined based on cost and feasibility. We gave preference to mediators of particular biological interest based on the literature and preliminary results of the meta-analysis. We incorporated the measurements from the exploratory study into the final meta-analysis as an additional study.

#### Validation study

We expected that the meta-analysis would identify a number of immune mediators that differed in concentration across the menstrual cycle. In the validation component of the study, we experimentally tested the accuracy of the meta-analysis by selecting 2–3 immune mediators that changed across the menstrual cycle and measuring them in the cohort described above. We determined the statistical power and expected result for each selected immune mediator before performing the measurements, but after performing the meta-analysis. The expected result was a direction of effect (increased or decreased in the luteal phase compared to the follicular). The power was determined using the sample size we selected above and the effect size and standard deviation from the meta-analysis. We only performed validation measurements for immune mediators where we had power greater than 90%. We considered the results to validate the meta-analysis for those immune mediators where we observed an effect in the predicted direction with a *p*-value < 0.05. Measurements from the validation study were incorporated as an additional study into the meta-analysis.

#### Immune mediator quantification using MSD and ELISA

Concentrations of selected immune mediators were measured using Meso Scale Discovery (MSD) R-Plex/U-Plex kits and ELISA. MSD assays were used where available because they allow simultaneous detection of multiple immune mediators in the same well. ELISA was used for immune mediators that were unavailable or cost-prohibitive by MSD. When ELISAs were used, they were purchased from R&D Systems wherever possible. To measure kallikrein-3, we used the Human Kallikrein 3/PSA DuoSet ELISA (R&D Systems, catalog DY1344). To measure progesterone, we used the Progesterone ELISA kit (Enzo Life Sciences, catalog ADI-901-011). We planned to measure hemoglobin in an MSD panel with other immune mediators, if compatible, or by Hemastix Blood ID Reagent Strips (Siemens).

Prior to running all of the samples, we chose the appropriate dilution for each analyte by running a pilot set of samples run with no dilution, 1:10 dilution, and 1:100 dilution (greater dilutions performed as needed). The diluent for CVL samples was 1% bovine serum albumin in phosphate buffered saline, unless a different diluent was required for a particular kit. The diluent for serum samples was the assay buffer provided with the Progesterone ELISA kit. We chose the dilution for each analyte that resulted in the largest proportion of tested samples in the detectable range.

MSD and ELISA were performed according to the protocols provided by the manufacturers. To limit batch/plate effects, we ran all samples from a given donor on the same plate, and we distributed follicular and luteal phase samples across plates.

The MSD data was analyzed using MSD Discovery Workbench software using the built-in concentration interpolation (typically four-parameter polynomial curve) and the concentrations were exported. For ELISA, concentrations were determined using a four-parameter polynomial curve. We analyzed the data from the exploratory and validation components of the study using the same two-stage process as for the studies collected from the literature, as described above. As for all other studies, the primary analysis was unadjusted, and in sensitivity analysis, we adjusted for covariates including hemoglobin, recent sexual contact, STI, and BV status.

## Results

### Protocol amendments

Several small changes and corrections to the protocol became necessary during the course of the study. These amendments are described in Additional file 2.

### Systematic review

**Fig. 1:**
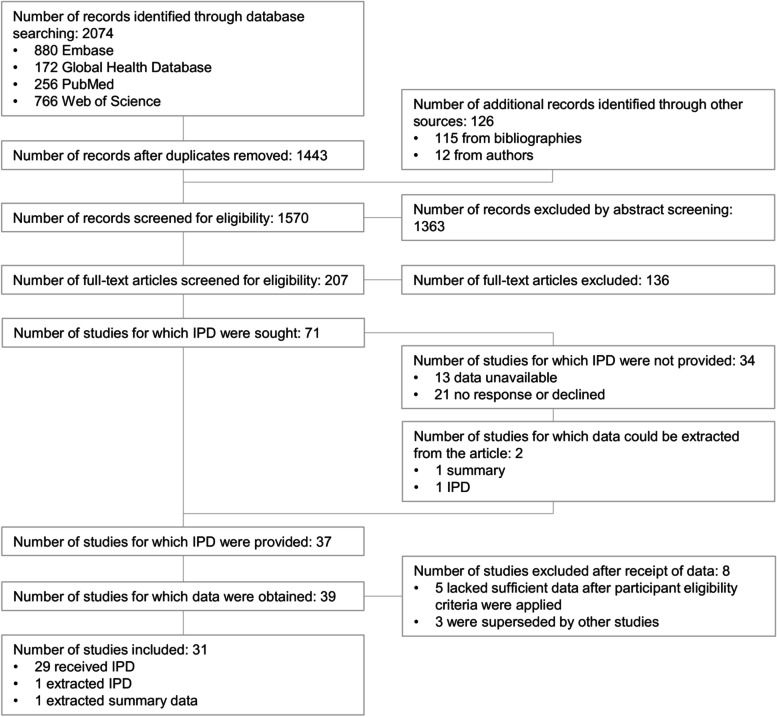
PRISMA-IPD flow diagram. Identification and selection of included studies

As shown in Fig. 1, we searched Embase (880 records), the Global Health Database (172 records), PubMed (256 records), and Web of Science (766 records) on April 22, 2020, and August 30, 2021, using the search strings described in Additional file 1. We did not need to update our search strategy. In total, 2074 records were retrieved. After de-duplication and removal of reviews and editorials, 1443 records remained. We identified an additional 126 records from review of bibliographies and author suggestions. In total, we reviewed 1570 abstracts. We excluded 1363 records after review of abstracts and 136 after review of full-text articles. We sought individual participant data (IPD) from 71 studies and received it from 37. We extracted data from publications of 2 additional studies where IPD was unavailable. Of these 39 studies, we removed 8 because of a lack of sufficient data remaining after participant-level eligibility criteria were applied (≤1 sample remaining per phase) or because the dataset overlapped with another included study. In total, data were available from 31 studies, of which 29 were IPD provided by the authors [10–15, 19, 21, 23, 26, 27, 48–62], 1 was IPD extracted from a paper [63], and 1 was summary data extracted from a paper [31]. Three of these data sets were previously unpublished. Including our validation and exploratory experiments described below as an additional study, we used data from 32 studies.

Table 2 shows the characteristics of the included studies. In total, the IPD consisted of 82,271 concentration measurements of 77 immune mediators from 4403 samples from 1600 participants. We excluded samples based on the pre-registered criteria described in the Methods, including use of hormonal contraception and samples collected outside of our cycle phase definitions. IPD were checked for integrity and no important issues were identified. After excluding samples, 39,589 measurements (48% of total) from 2112 samples (48%) from 871 participants (54%) were eligible for inclusion in the primary analysis.

**Table 2:** Characteristics of studies

Study	Participants	Immune factors	Sample type	Assay method	Phasing method	Luteal samples	Follicular samples	Periovulatory samples	Countries	Data source	Risk of bias^**e**^
Arnold-2016 [48]	23	14	CVL	MSD	Days since LMP	7	16	-	Kenya	IPD	4
Barousse-2007 [49]	22	16	CVL	ELISA	Progesterone	15	16	-	USA	IPD	5
Boily-Larouche-2019 [10]	26	19	CVL	Luminex^b^	Progesterone	23	14	-	Kenya	IPD	4
Bradley-2018 [11]	16	10	Swab^c^	Luminex	Progesterone plus LH	14	22	5	Sweden	IPD	6
Byrne-2016 [12]	49	14	CVL	Luminex	Progesterone^a^	16	33	-	South Africa	IPD	6
Castle-2002 [13]	11	4	Sponge^d^	ELISA	Days since LMP	5	6	-	USA	IPD	4
Cortez-2014 [14]	14	26	Swab^c^	Luminex	Days since LH peak	143	136	27	Kenya	IPD	5
Fidel-2003 [50]	112	12	CVL	ELISA	Progesterone plus LH	36	58	18	USA	IPD	4
Francis-2016 [15]	42	45	CVL	Luminex	Urine PDG/CRT	206	131	-	Tanzania	IPD	5
Ghosh-2010 [51]	16	4	CVL	ELISA	Days since LMP	6	10	-	USA	IPD	5
Hughes-2021 [52]	28	15	Menstrual cup	ELISA, MSD	Progesterone	19	27	-	USA	IPD	6
Hughes-unpublished	90	20	CVL	MSD	Progesterone	65	88	-	Kenya	IPD	5
Hwang-2011 [53]	8	11	CVL	Luminex	Days since LMP	5	3	-	USA	IPD	4
Jais-2016 [19]	20	11	CVL	ELISA	Days since LMP	20	20	-	USA	IPD	5
Jais-2017 [54]	20	2	CVL	ELISA	Days since LMP	20	20	-	USA	IPD	5
Jespers-2017 [21]	37	12	CVL	Luminex, MSD, ELISA	Days since LMP	74	110	-	Rwanda, South Africa, Kenya	IPD	5
Joag-unpublished	18	16	Menstrual cup	MSD	Progesterone	12	21	-	Kenya	IPD	5
Kyongo-2012 [23]	31	12	CVL	Luminex, ELISA	Days since LMP^a^	59	87	-	Belgium	IPD	5
Lahey-2012 [55]	16	3	CVL	ELISA	Days since LMP	6	10	-	USA	IPD	4
Lieberman-2008 [56]	8	14	Sponge^d^	Luminex	Days since LMP	3	5	-	USA	IPD	5
Makinde-2018 [26]	7	38	Menstrual cup	Luminex	Days since LMP^a^	7	7	-	UK	IPD	4
Moscicki-2020 [57]	18	13	CVL	Luminex	Days since LMP	20	21	-		IPD	5
Novak-2007 [58]	49	4	Sponge^d^, CVL	ELISA	Days since LMP	19	33	-	USA	IPD	5
Patel-2014 [27]	4	6	Menstrual cup	ELISA	Days since LMP^a^	4	8	-	USA	IPD	3
Safaeian-2009 [63]	23	2	Sponge^d^	ELISA	Days since LH peak^a^	23	23	23	Costa Rica	Extracted IPD	5
Sriprasert-2020 [59]	7	2	CVL	ELISA	Progesterone	5	15	-	USA	IPD	5
Thurman-2015 [60]	13	17	CVL	MSD	Days since LMP	8	5	-	USA	IPD	4
Thurman-2017 [61]	20	14	CVL	ELISA, Luminex	Progesterone	15	22	-	USA	IPD	6
Thurman-unpublished	15	16	CVL	ELISA, Luminex	Days since LMP	11	15	-	USA	IPD	5
Yegorov-2019 [62]	9	19	Menstrual cup	ELISA, MSD	Days since end of LMP	3	6	-	Uganda	IPD	4
Shust-2010 [31]	9	16	CVL	ELISA, Luminex	Progesterone^a^	26	25	-	USA	Extracted summary	6
New wet lab data^f^	99	13	CVL	ELISA, MSD	Progesterone	80	102	-	Kenya	IPD	5

The number of samples shown includes only those that were eligible for inclusion in the primary analysis. *CVL* cervicovaginal lavage, *LMP* last menstrual period, *LH* luteinizing hormone, *PDG* Pregnanediol-3-Glucuronide, *CRT* creatinine, *MSD* Meso Scale Discovery

^a^Progesterone concentrations or days since LMP/LH peak were unavailable, so samples were phased based on the phases assigned by the study authors

^b^“Luminex” includes other bead-based immunoassays

^c^Both swab studies used vaginal swabs

^d^All sponge studies sampled the endocervix or the cervical os

^e^Risk of bias scale: high = 0–1, medium = 2–3, low = 4–6

^f^The exploratory and validation experiments performed for this article

All code and data necessary to reproduce the analyses shown in this paper are included in Additional file 3, including IPD for those studies where study investigators agreed to publish.

### Primary result

**Fig. 2:**
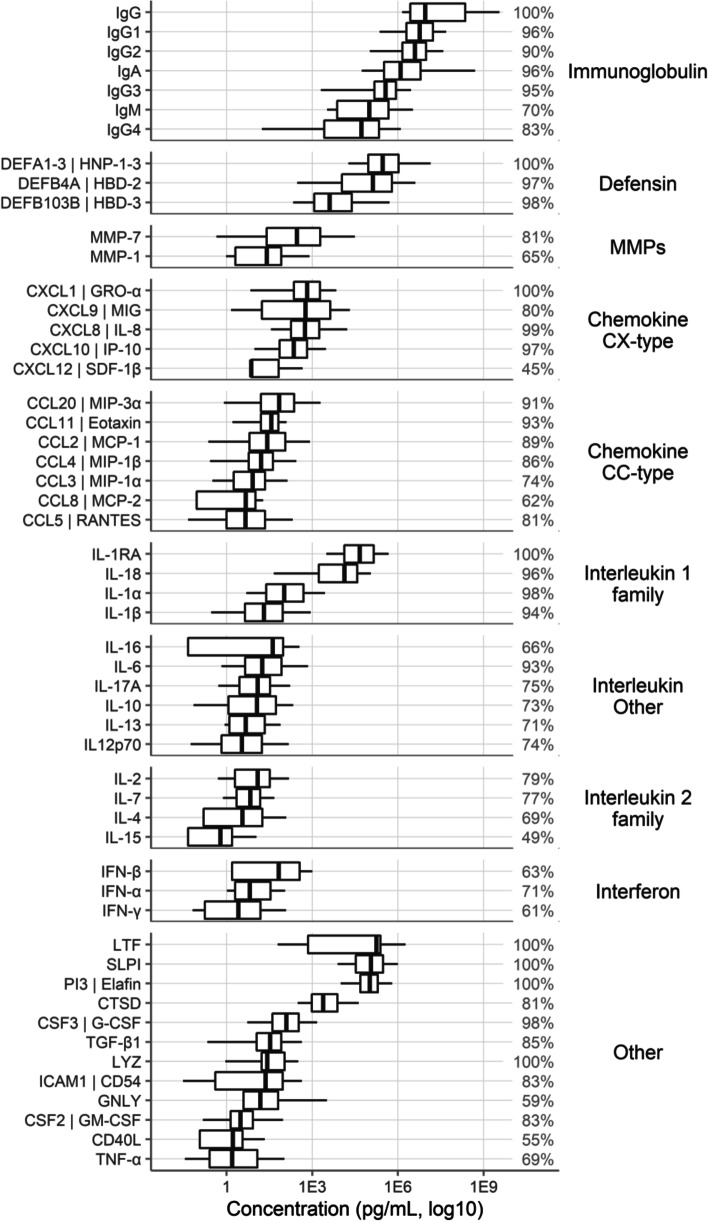
Concentrations of immune mediators. Concentration ranges for all immune mediators measured in at least 2 studies. The box-and-whisker plots show concentrations including all studies and sample types. The middle bar shows the median, with the edges of the box ranging from the 25th to the 75th percentiles and the whiskers ranging from the 5th to the 95th percentiles. The percentages shown at the right reflect the percent of samples detected above the lower limit of detection

A total of 53 of the 77 immune mediators (69%) were measured in at least two studies. The concentration ranges for these factors are shown in Fig. 2. Immunoglobulins were the most abundant immune mediators, followed by defensins, lactoferrin, SLPI, elafin, and IL-1RA.

**Fig. 3:**
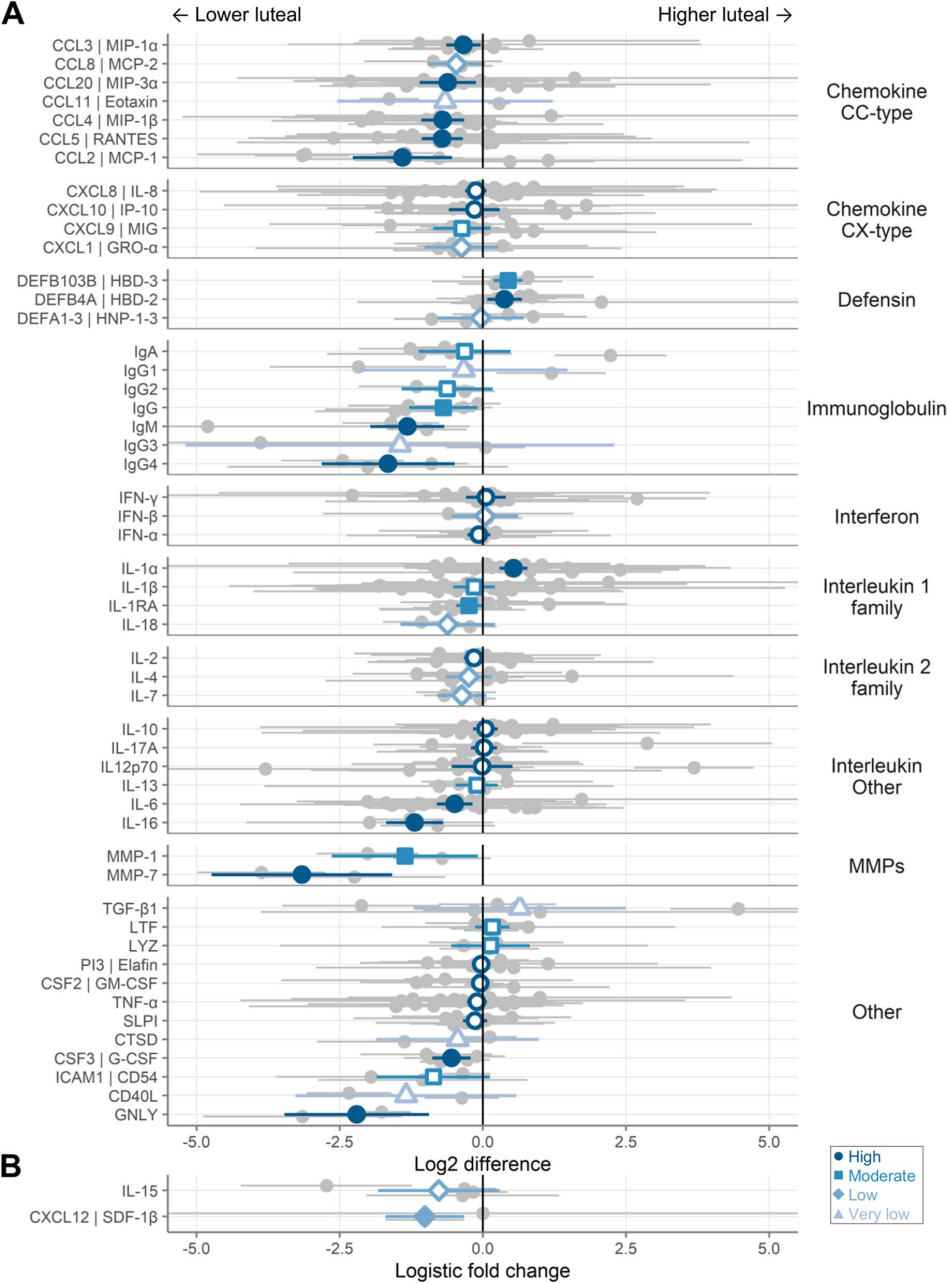
Primary meta-analyses. **A** The log2 difference between phases (log2-pg/mL of the luteal phase minus log2-pg/mL of the follicular phase). **B** The logistic difference between phases (log-odds of proportion detectable in luteal vs. follicular phase). Each row represents a different immune mediator, with the symbols showing the mean and the lines showing the 95% confidence intervals. Gray symbols indicate individual studies and blue the meta-estimates as determined by inverse-variance pooling random effects models. Filled symbols indicate *p* < 0.05 while open symbols indicate *p* > 0.05. Positive numbers indicate higher during the luteal phase (relative to the follicular phase), while negative numbers indicate lower during the luteal phase (relative to the follicular phase). Symbol shape and shade of blue indicate the GRADE strength of evidence

Of these factors, 51 were detectable in at least half of all samples. As shown in Fig. 3A, a number of immune mediators were lower in the luteal phase than in the follicular phase, including chemokines (especially CC-type), immunoglobulins, IL-6, IL-16, IL-18, GNLY, G-CSF, and MMPs. In contrast, only IL-1α, HBD-2, and HBD-3 were higher in luteal phase samples compared to follicular phase samples. As shown in Table 3, which also lists the full name for each factor, 18 immune mediators were different between the phases with *p*<0.05, of which 12 remained *p*<0.05 after adjustment by FDR and 8 after adjustment by Holm-Bonferroni.

**Table 3:** Summary of primary meta-analyses (linear models)

Category	Immune mediator	Name	Log2 difference	Standard error	***P***-value	FDR	Holm-Bonferroni	***I***^**2**^	Number of studies	GRADE
Chemokine CC-type	CCL3 | MIP-1α	C-C motif chemokine ligand 3	− 0.34	0.15	0.027	0.086	0.968	53	10	High
	CCL8 | MCP-2	C-C motif chemokine ligand 8	− 0.47	0.26	0.069	0.184	1	0	2	Low
	CCL20 | MIP-3α	C-C motif chemokine ligand 20	− 0.61	0.25	0.014	0.055	0.553	41	13	High
	CCL11 | Eotaxin	C-C motif chemokine ligand 11	− 0.66	0.96	0.492	0.66	1	98	2	Very low
	CCL4 | MIP-1β	C-C motif chemokine ligand 4	− 0.7	0.19	2.3E-4	0.002	0.01	73	14	High
	CCL5 | RANTES	C-C motif chemokine ligand 5	− 0.71	0.18	9.7E−5	9.9E−4	0.005	23	15	High
	CCL2 | MCP-1	C-C motif chemokine ligand 2	− 1.4	0.44	0.001	0.007	0.06	81	8	High
Chemokine CX-type	CXCL8 | IL-8	C-X-C motif chemokine ligand 8	− 0.12	0.09	0.193	0.365	1	10	24	High
	CXCL10 | IP-10	C-X-C motif chemokine ligand 10	− 0.15	0.23	0.514	0.66	1	65	13	High
	CXCL9 | MIG	C-X-C motif chemokine ligand 9	− 0.37	0.26	0.152	0.31	1	47	8	Moderate
	CXCL1 | GRO-α	C-X-C motif chemokine ligand 1	− 0.37	0.33	0.259	0.426	1	0	4	Low
Defensin	DEFB103B | HBD-3	defensin beta 103B	0.44	0.13	6.5E−4	0.004	0.028	0	4	Moderate
	DEFB4A | HBD-2	defensin beta 4A	0.38	0.16	0.015	0.055	0.571	2	8	High
	DEFA1-3 | HNP-1-3	Human neutrophil peptides 1-3	− 0.04	0.38	0.921	0.94	1	73	4	Low
Immunoglobulin	IgA	Immunoglobulin A	− 0.32	0.41	0.438	0.653	1	82	7	Moderate
	IgG1	Immunoglobulin G1	− 0.33	0.92	0.718	0.799	1	86	3	Very low
	IgG2	Immunoglobulin G2	− 0.62	0.41	0.124	0.276	1	53	2	Moderate
	IgG	Immunoglobulin G	− 0.69	0.3	0.023	0.079	0.862	58	5	Moderate
	IgM	Immunoglobulin M	− 1.32	0.33	6.3E−5	9.9E−4	0.003	44	3	High
	IgG3	Immunoglobulin G3	−1.45	1.91	0.448	0.653	1	75	2	Very low
	IgG4	Immunoglobulin G4	−1.65	0.59	0.005	0.022	0.206	68	3	High
Interferon	IFN-γ	Interferon gamma	0.05	0.18	0.758	0.823	1	51	16	High
	IFN-β	Interferon beta 1	0.04	0.3	0.904	0.94	1	0	2	Low
	IFN-α	Interferon alpha 2	−0.07	0.1	0.49	0.66	1	0	6	High
Interleukin 1 family	IL-1α	Interleukin 1 alpha	0.54	0.12	1.4E−5	3.6E−4	7.0E−4	51	20	High
	IL-1β	Interleukin 1 beta	−0.15	0.19	0.409	0.632	1	40	20	Moderate
	IL-1RA	Interleukin 1 receptor antagonist	−0.24	0.11	0.034	0.101	1	39	10	Moderate
	IL-18	Interleukin 18	−0.61	0.42	0.146	0.31	1	76	2	Low
Interleukin 2 family	IL-2	Interleukin 2	−0.16	0.09	0.08	0.205	1	0	11	High
	IL-4	Interleukin 4	−0.25	0.21	0.227	0.4	1	76	8	Low
	IL-7	Interleukin 7	−0.37	0.22	0.092	0.213	1	73	3	Low
Interleukin Other	IL-10	Interleukin 10	0.05	0.11	0.681	0.799	1	15	18	High
	IL-17A	Interleukin 17A	0.02	0.11	0.857	0.911	1	39	8	High
	IL12p70	Interleukin 12 p70	−0.01	0.27	0.959	0.959	1	78	16	High
	IL-13	Interleukin 13	−0.11	0.19	0.573	0.695	1	0	4	Moderate
	IL-6	Interleukin 6	−0.49	0.16	0.002	0.009	0.083	60	21	High
	IL-16	Interleukin 16	−1.19	0.25	2.9E−6	1.5E−4	1.5E−4	0	3	High
MMPs	MMP-1	Matrix metallopeptidase 1	−1.36	0.65	0.037	0.104	1	77	2	Moderate
	MMP-7	Matrix metallopeptidase 7	−3.16	0.8	8.6E−5	9.9E−4	0.004	63	2	High
Other	TGF-β1	Transforming growth factor beta 1	0.64	0.94	0.495	0.66	1	91	6	Very low
	LTF	Lactotransferrin	0.17	0.15	0.28	0.447	1	0	5	Moderate
	LYZ	Lysozyme	0.13	0.35	0.703	0.799	1	0	3	Moderate
	PI3 | Elafin	Peptidase inhibitor 3	−0.03	0.08	0.72	0.799	1	25	10	High
	CSF2 | GM-CSF	Colony-stimulating factor 2	−0.05	0.07	0.517	0.66	1	27	8	High
	TNF-α	Tumor necrosis factor	−0.1	0.09	0.248	0.421	1	23	19	High
	SLPI	Secretory leukocyte peptidase inhibitor	−0.14	0.11	0.208	0.38	1	11	10	High
	CTSD	Cathepsin D	−0.44	0.72	0.542	0.674	1	70	2	Very low
	CSF3 | G-CSF	Colony-stimulating factor 3	−0.55	0.17	0.001	0.007	0.06	55	5	High
	ICAM1 | CD54	Intercellular adhesion molecule 1	−0.86	0.51	0.087	0.211	1	46	3	Moderate
	CD40L	CD40 ligand	−1.34	0.98	0.172	0.338	1	93	2	Very low
	GNLY	Granulysin	−2.2	0.64	6.2E−4	0.004	0.028	56	2	High

Log2 difference, difference between phases (log2-pg/mL of the luteal phase minus log2-pg/mL of the follicular phase) with positive numbers indicating higher concentrations in the luteal phase (relative to the follicular phase), while negative numbers indicate lower concentrations in the luteal phase (relative to the follicular phase); *FDR* false discovery rate, *I*^*2*^ statistical heterogeneity between studies, from low (0) to high (100), *GRADE* Grading of Recommendations, Assessment, Development and Evaluations strength of evidence framework (very low, low, moderate, high)

Two additional immune mediators were detectable in less than half of all samples. These immune mediators were analyzed with logistic models and are shown in Fig. 3B and Table 4.

**Table 4:** Summary of primary meta-analyses (logistic models)

Category	Immune mediator	Name	Log2 difference	Standard error	***P***-value	FDR	Holm-Bonferroni	I^**2**^	Number of studies	GRADE
Chemokine CX-type	CXCL12 | SDF-1β	C-X-C motif chemokine ligand 12	− 1.01	0.35	0.004	0.008	0.008	0	2	Low
Interleukin 2 family	IL-15	interleukin 15	− 0.77	0.54	0.155	0.155	0.155	70	4	Low

Logistic fold change, difference between phases (log-odds of proportion detectable in luteal vs. follicular phase) with positive numbers indicating higher concentrations in the luteal phase (relative to the follicular phase) and negative numbers indicating lower concentrations in the luteal phase (relative to the follicular phase); *FDR* False discovery rate, *I*^*2*^ statistical heterogeneity between studies, from low (0) to high (100), *GRADE* Grading of Recommendations, Assessment, Development and Evaluations strength of evidence framework (very low, low, moderate, high)

The meta-analysis reported in this section includes all eligible data from all studies, including the validation and exploratory experiments described below.

Additional file 4 contains comprehensive overviews of each immune mediator, including raw concentration data (IPD) and detailed meta-analysis forest plots. These overviews show the difference between phases separately for each immune mediator within each study, as well as the weighting of each study in the overall meta-estimate.

The remaining 24 of the 77 immune mediators (31%) were measured in only single studies and meta-analysis could not be performed. These immune mediators and the results from the single studies are shown in Table S1.

### Risks of bias and strength of evidence

#### Risk of publication bias

We assessed whether there was evidence of non-publication of results (i.e., publication bias) for all immune mediators that were measured in at least 10 studies. The risk of publication bias was assessed using Egger’s tests and funnel plots, where asymmetry would be suggestive of possible publication bias (Fig. S1). There was no evidence of publication bias for any of these immune mediators.

#### Risk of bias

The overall risk of bias at the study level was assessed using the instrument in Additional file 1. The risk of bias was generally low in these studies, as shown in the last column of Table 2.

#### Strength of evidence

We used the GRADE framework to assess the quality of evidence for all immune mediators as described in the methods. The GRADE ratings are listed in Tables 3 and 4 and Fig. 3. Overall, the evidence strength was high for 26 immune mediators, moderate for 12, low for 9, and very low for 6.

### Periovulatory results

Only four studies included periovulatory samples and the number of included samples was small (Table 2). Meta-analysis was possible for ten immune mediators, comparing follicular samples to periovulatory samples (Fig. S2A; Table S2) and comparing luteal samples to periovulatory samples (Fig. S2B; Table S3). The confidence intervals were quite wide in many cases, as were the I^2^ values, indicating substantial heterogeneity between studies and low confidence. By *p*-value, the strongest results were higher levels of IL4 in the follicular phase than the periovulatory phase, as well as higher levels of CXCL8 in both the luteal and follicular phases than the periovulatory phase.

### Additional wet lab experiments

We selected our validation and exploratory immune mediators based on an interim version of the meta-analysis, which contained data from all studies that were available at the time (29 of the 32 studies included in the final version).

#### Pre-registered validation experiment

**Fig. 4:**
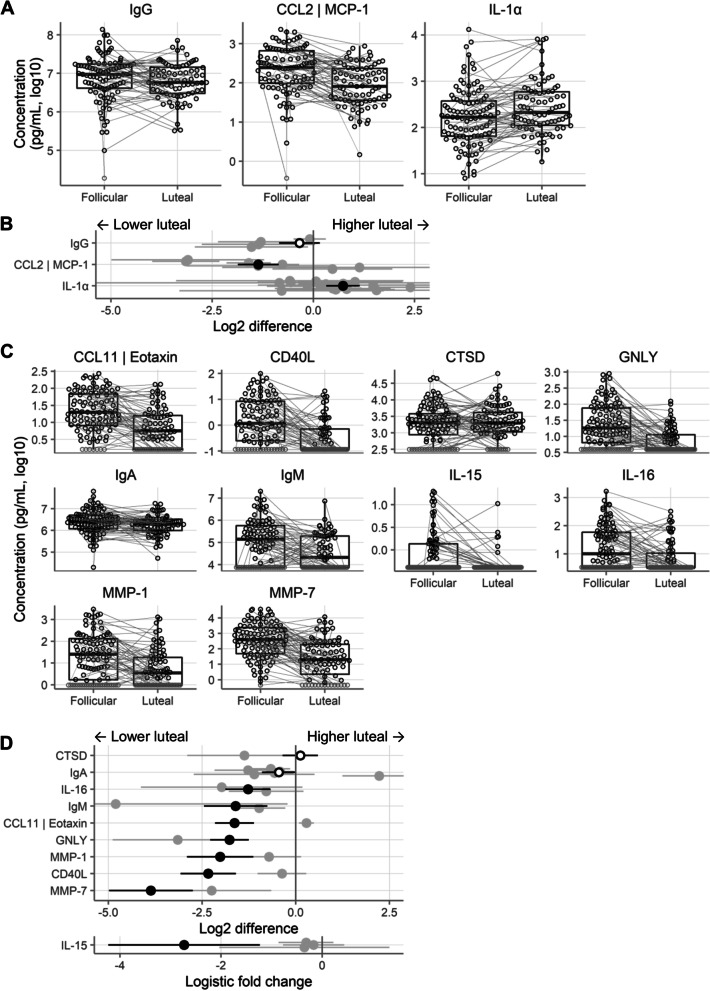
Validation and exploratory experiments. **A** Concentrations of validation cytokines. Each symbol shows the concentration in a single sample. Lines connect samples from the same participant. Pale grey symbols are below the lower limit of detection. **B** Differences in concentrations between phases of the menstrual cycle for the validation cytokines. Black shows the new data generated in this study, gray shows all other studies. Error bars for several CCL2 and IL-1α studies extend off-scale. Black-filled symbols indicate *p*<0.05, open symbols indicate *p*>0.05. **C** Concentrations of exploratory cytokines, as in **A**. **D** Differences in concentrations between phases of the menstrual cycle for the exploratory cytokines, as in **B**. Error bars for several IgA and IgM studies extend off-scale

Based on this interim meta-analysis, we met our pre-registered statistical power threshold of 0.9 for one immune mediator, total IgG (power = 0.96). Therefore, we only performed a validation experiment for a single immune mediator, rather than 2–3 as specified in the protocol. We predicted that IgG would be lower in the luteal phase. We measured IgG by MSD in 200 CVL samples from 100 participants from Kenya (Fig. 4A), with a final sample size of 178 CVL samples from 99 participants after excluding samples with insufficient volume or where serum progesterone levels fell outside the limits of our menstrual phase definitions. We found that IgG was 0.342 log2 units lower in the luteal phase than the follicular phase, with *p* = 0.183 (Fig. 4B), so the direction of effect was as predicted, but the *p*-value did not meet our specified threshold for statistical significance of 0.05.

#### Non-pre-registered validation experiments

We measured two additional validation immune mediators despite not meeting the pre-registered threshold for power. We felt that the experiments had the potential to be instructive and would at minimum contribute additional data to the meta-analysis. We chose the two immune mediators with the highest estimated statistical power other than total IgG: CCL2 (expected to be lower luteal; Fig. 4A) and IL-1α (expected to be higher luteal; Fig. 4A). We measured each by MSD and confirmed CCL2 to be lower in the luteal phase (−1.36 log2 units, *p* = 9.4E−7) and IL-1α to be higher in the luteal phase (0.73 log2 units, *p* = 8.0E−4; Fig. 4B).

#### Exploratory experiments

We used these same samples for exploratory experiments of immune mediators that were measured in few studies. We chose the following immune mediators (all measured in 1–2 studies at the time the reagents were ordered): MMP1, MMP7, CCL11, CD40L, IL-15, IL-16, and IgM (all by MSD), as well as GNLY and CTSD by ELISA. We also measured IgA, even though it did not meet our criteria for validation (power >0.9) or exploratory (measured in 1–2 studies) experiments; we included it because it was included in the multiplex IgA, IgG, and IgM MSD kit. As described in the methods, we also measured total protein concentrations by BCA assay, PSA levels by ELISA, and hemoglobin A by MSD. Measurements were available from 175 to 182 samples from 98 to 99 participants per immune mediator after excluding samples as described above or that failed QC. Concentrations of these immune mediators are shown in Fig. 4C. IL-15 was detected in fewer than 50% of samples, so it was analyzed using logistic models. All of these immune mediators were lower in the luteal phase than the follicular phase, except for CTSD (Fig. 4D). The data from this experiment is included in the main meta-analysis in Fig. 3, substantially increasing the number of samples as well as the list of immune mediators included in the final meta-analysis.

### Subgroup analysis

We next conducted univariate subgroup analyses to determine whether the effect of the menstrual cycle phase was modified by any of four key study-level covariates: sample type, assay method, geographical region, or method of determining the menstrual cycle phase. These subgroup analyses replace the planned meta-regression analysis as described in Additional file 2.

For the subgroup analyses, we performed separate meta-analyses within each subgroup for each immune mediator. For example, in analyzing the sample type covariate for CCL2, at least two studies were performed using CVL samples and at least two using menstrual cups. We performed separate meta-analyses for the CVL studies and the menstrual cup studies. We then compared those results to a meta-analysis of all of the CCL2 studies combined. We repeated this process for each immune mediator and for each of the four study-level covariates.

**Fig. 5:**
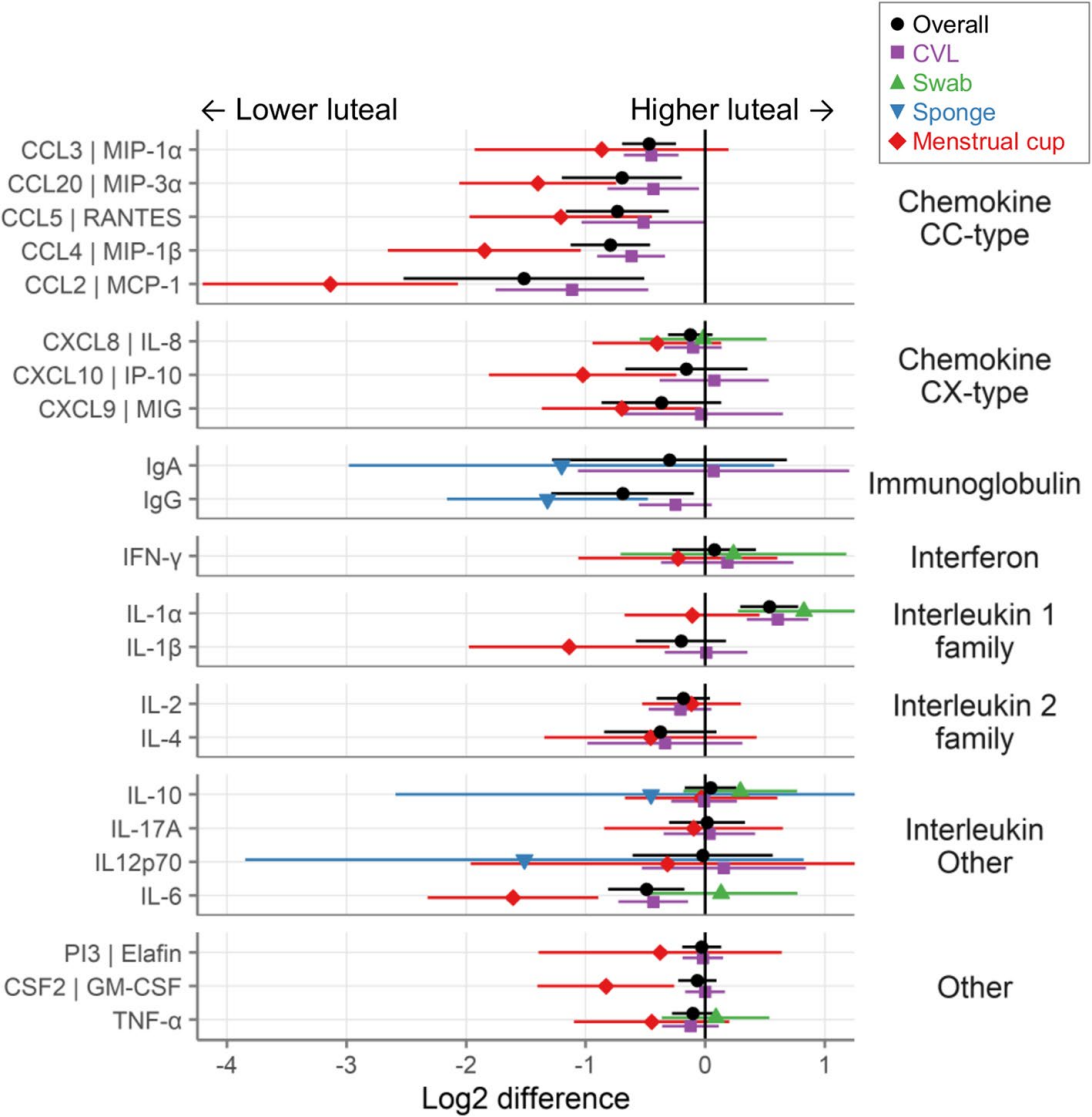
Subgroup analysis: Does the effect of menstrual cycle differ by sample type? Meta-analyses comparing all studies (black circles) to studies grouped by sample type (menstrual cup: red diamonds; sponge: blue inverted triangles; CVL: purple squares; swab: green triangles)

Figure 5 shows the subgroup analysis for sample type. In general, the directions of the effects are the same regardless of sample type. For example, CCL2 is lower in the luteal phase than the follicular phase whether measured in CVL samples or in menstrual cup samples. However, there is a general pattern of a greater effect in menstrual cup samples than in CVL samples. For example, CC-type chemokines were all lower in the luteal phase than the follicular phase, but this difference is more pronounced in menstrual cup samples than in CVL samples. A similar effect is seen for cervical sponge samples, but not for vaginal swabs, though the numbers of studies using sponges or swabs were low. This pattern held for most immune mediators, but not all (e.g., IL-4, IL-2).

The subgroup analyses of the assay method (Fig. S3A), geographical region of sample origin (Fig. S3B), and menstrual cycle phasing method (Fig. S3C) did not identify any consistent patterns of these variables modifying the effect of the menstrual cycle phase.

### Sensitivity analyses

#### One-stage meta-analysis

**Fig. 6:**
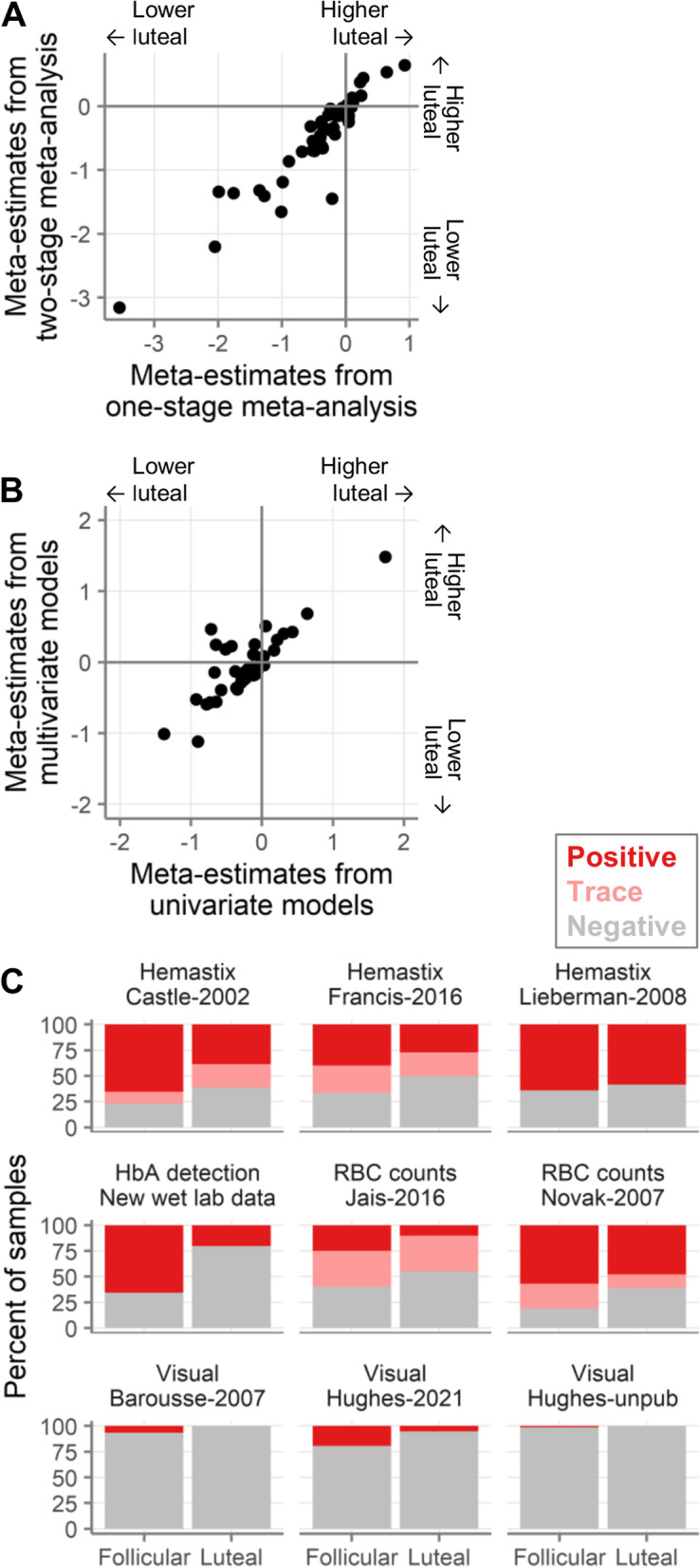
Sensitivity analyses. **A** Correlation of effect sizes (log2-pg/mL of the luteal phase minus log2-pg/mL of the follicular phase) of meta-estimates derived from one- and two-stage meta-analysis. Each symbol indicates an immune mediator. **B** Correlation of effect sizes (log2-pg/mL of the luteal phase minus log2-pg/mL of the follicular phase) of meta-estimates derived from underlying univariate models or multivariate models adjusted for relevant covariates. Each symbol indicates an immune mediator. **C** Percentage of samples with red blood cells detected using the indicated detection methods. Dark red indicates positive, light red indicates trace detection, and grey indicates negative

As a pre-specified sensitivity analysis, we performed a one-stage meta-analysis. Specifically, we pooled the raw data from all studies and assessed the effect of the menstrual phase in a single model per immune mediator, with participant and study as random effects. This approach differs from our primary analysis reported above, where we used a two-stage approach, first analyzing each study separately and then combining the results by meta-analysis. The results of this one-stage meta-analysis confirmed the results of our primary analysis (Fig. 6A, Pearson correlation coefficient *r* = 0.93 for correlation of effect sizes between one- and two-stage analyses).

#### Accounting for possible underlying confounding variables with multivariate study-level models

Because different covariates were measured in each study, our primary analysis did not adjust for covariates. To test whether the observed differences in immune mediator concentrations between phases were affected by covariates, we re-analyzed each study, adjusting for all relevant covariates for each study. The exact covariates adjusted for in each study are listed in Table S4. The most common covariates were bacterial vaginosis and detection of red blood cells (RBCs). Several studies were omitted, either because no covariates were reported or because there were too few samples to perform multivariate analysis. In addition, many samples had to be omitted due to missing covariate information. Because some samples had to be omitted in the multivariate analysis, we repeated our univariate meta-analysis on just the samples that could be included in the multivariate analysis, to allow for direct comparison. Thus, the univariate meta-analysis reported in this section differs slightly from the primary analysis, due to the smaller sample size used here. The meta-estimates of effect size were highly correlated between the univariate and multivariate analyses (Fig. 6B; Pearson *r* = 0.82), confirming our primary results. However, the covariates measured in each study were highly variable and the sample size per study was often limited.

We noticed that one covariate in particular was associated with cycle phase: presence of RBCs or hemoglobin in the samples (Fig. 6C). Therefore, we assessed this covariate further in an exploratory analysis that was not preplanned. Six studies used methods that could detect microscopic levels of blood (hemastix, hemoglobin A MSD assay, or RBC counts), and three used visual inspection. Microscopic levels of RBCs were detected in more than half of the samples. In contrast, visual inspection classified few samples as containing blood. Across all methods, there was a consistent pattern of greater RBC detection in follicular phase samples.

### Exploration of variation in effects

Ten immune mediators had high levels of heterogeneity (*I*^2^ statistic > 75%; Table 3). For six of these immune mediators, we were able to attribute most of the heterogeneity to one of three factors: inconsistent levels of detectability between studies, variation between sample types, and single study outliers. We were unable to explain the high levels of statistical heterogeneity for the remaining four immune mediators (CCL11, IL-4, IL-18, and IgG1).

The statistical heterogeneity for CD40L and MMP1 was primarily due to differences in detection between studies. Both immune mediators were only measured in two studies and there were considerable differences in the proportion of samples where the immune mediator was detected between studies (CD40L: 27% vs 64%; MMP1 49% vs 70%). In both cases, replacing the linear models with logistic models substantially reduces the heterogeneity (I^2^ to 55% for CD40L and 37% for MMP1) and results in statistically significant (*p *< 0.05) decreases in the luteal phase for both factors.

The statistical heterogeneity for CCL2 was primarily due to variations in effect by sample type. As previously discussed (Fig. 5), we observed differences in effect between sample types, with larger effects seen in menstrual cup samples. That difference drives the heterogeneity for CCL2, where the heterogeneity within each sample type is low to moderate (*I*^2^ 0–54%) and the high overall heterogeneity is caused by differences across sample types.

The statistical heterogeneity for IgA, IL-12, and TGF-β1 was primarily caused by single studies that differed substantially from the other studies (shown in Additional file 4). For IgA, omitting a single small study (less than 10 samples) reduces the heterogeneity to 0 and results in a statistically significant decrease of IgA in the luteal phase of −0.56 log2 units (*p*<0.05). For IL-12 and TGF-β1, dropping a single outlier study reduces *I*^2^ to 22% and 71%, respectively. Variation from sample type may additionally be contributing to residual statistical heterogeneity for TGF-β1, but the number of studies in each group is too small to draw confident conclusions.

### Secondary outcomes

#### Sample type

As a secondary outcome, we wished to determine whether one type of sample yielded higher concentrations and detection rates for immune mediators (regardless of menstrual phase). Thus, we compared the immune mediator concentrations detected by menstrual cup, sponge, and swab to CVL (which was by far the most common sample type). For this analysis, we included all immune mediators that were measured in at least two sample types and where each sample type was used in at least two studies.

**Fig. 7:**
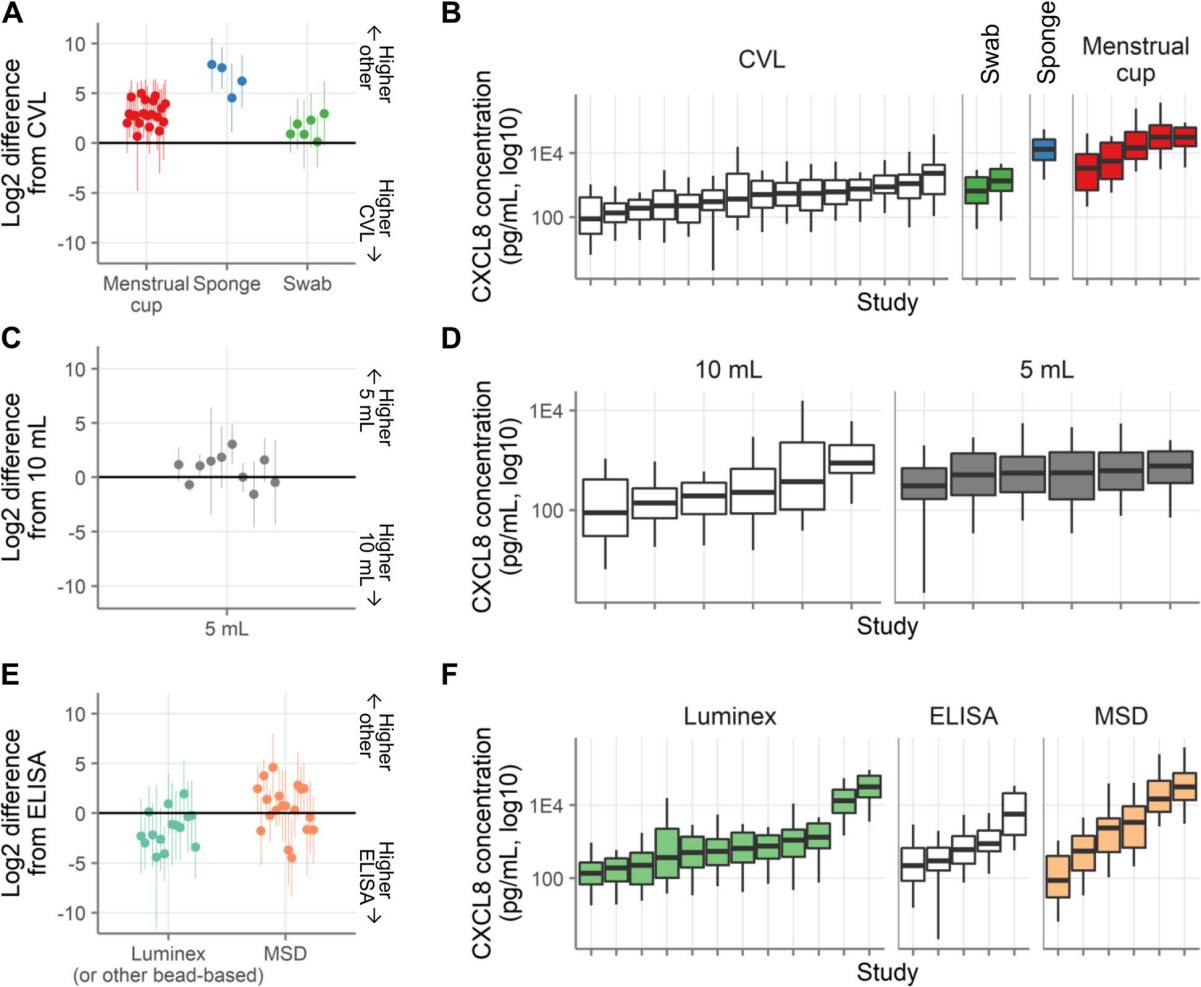
Secondary outcomes: Sample type and assay method comparison. **A** Comparison of concentrations recovered from CVLs to concentrations recovered from other sample types. Each symbol represents one immune mediator. The circles show the mean log2 difference between the indicated sample types and CVLs. **B** CXCL8 concentrations recovered by sample type. Each box plot shows a single study, colored by sample type in that study, with menstrual cup shown in red, sponge shown in blue, swab shown in green, and CVL shown in white. The studies are sorted by median concentration. **C** Comparison of concentrations recovered from 10 mL CVLs to concentrations recovered from 5 mL CVLs. Each symbol represents one immune mediator. The circles show the mean log2 difference between 5 and 10 mL CVLs. **D** CXCL8 concentrations recovered by CVL volume. Each box plot shows a single study, colored by CVL volume in that study, with 5 mL shown in grey and 10 mL shown in white. The studies are sorted by median concentration. **E** Comparison of concentrations detected by ELISA to concentrations detected by other assays. Each symbol represents one immune mediator. The circles show the mean log2 difference between the indicated assays and ELISAs. **F** CXCL8 concentrations measured by assay type. Each box plot shows a single study, colored by assay type in that study, with Luminex shown in green, MSD shown in orange, and ELISA shown in white. The studies are sorted by median concentration

As shown in Fig. 7A, menstrual cup, sponge, and swab consistently resulted in higher total concentrations than CVL, as expected. For all three sample types, the concentrations were higher than CVL for every immune mediator (*p*<0.05 for 12/20 immune mediators by menstrual cup, 4/4 by sponge, and 0/6 by swab). The study-level concentrations are illustrated for one representative immune mediator (CXCL8, selected because it was the immune mediator measured in the most studies) in Fig. 7B.

#### Variation in CVL methods

All studies used clinician-collected CVLs. The CVL medium was saline in 19 studies, phosphate-buffered saline in 2 studies, and unspecified in another study. Thus, we did not have sufficient variation in methods to assess the effect of clinician- vs. self-collection or of lavage medium.

There was more variation in volume of CVL collected: 10 studies used 10 mL, 8 studies used 5 mL, 2 studies used 4 mL, 1 study used 2 mL, and 1 study did not specify. We were therefore able to compare concentrations of immune mediators recovered from 5 and 10 mL lavages (including all immune mediators that were measured in at least two studies at each volume). As shown in Fig. 7C, there was not a consistent difference between the concentrations of immune mediators detected in 5 and 10 mL CVLs (concentrations higher in 5 mL CVLs for 6/10 immune mediators, with *p*<0.05 for 1 of these; concentrations higher in 10 mL CVLs for the other 4 immune mediators with all *p*>0.05). This is illustrated at the level of individual studies in Fig. 7D, where the concentrations of CXCL8 detected in each study are shown stratified by CVL volume.

#### Assay method

As an additional secondary outcome, we sought to determine whether one assay method yielded higher concentrations than the others. We compared the immune mediator concentrations detected by Luminex and MSD to ELISA (regardless of menstrual phase). For this analysis, we included all immune mediators that were measured using at least two assay methods, with each assay method being used in at least two studies.

As shown in Fig. 7E, Luminex gave lower total concentrations than ELISA for 12/15 immune mediators (*p*<0.05 for 3) and higher concentrations for 3/15 immune mediators (all *p*>0.05). MSD was mixed, with lower concentrations for 7/19 immune mediators (*p*<0.05 for 1) and higher concentrations for 12 (*p*<0.05 for 2 of these). This is illustrated at the level of individual studies in Fig. 7F, using CXCL8 as a representative example. As discussed in the Subgroup Analysis section above, the effect of menstrual cycle did not differ by assay method.

#### Method of determining menstrual phase

**Fig. 8:**
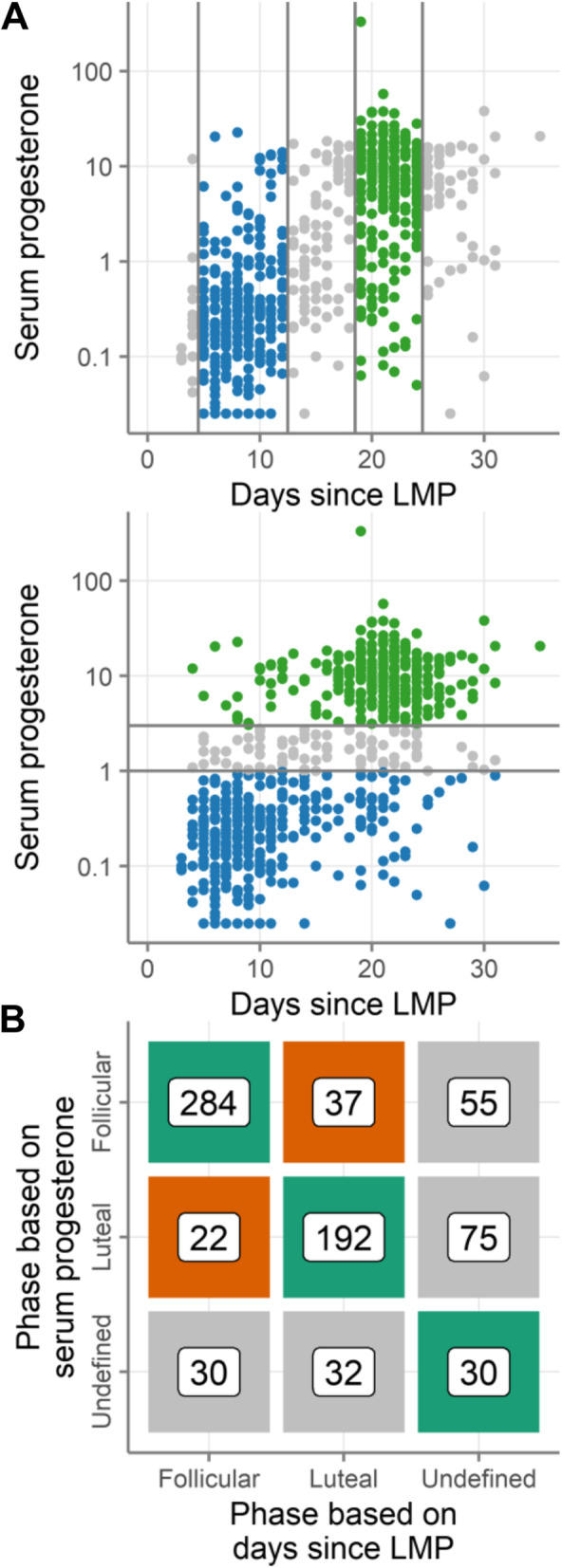
Secondary outcomes: Method of determining menstrual phase. **A** Progesterone concentrations (log10 ng/mL) and days since last menstrual period. Each symbol is a single sample. Samples are categorized into follicular (blue) or luteal (green) phases based on days since LMP (top) or serum progesterone (bottom). The same samples are shown in both plots. Gray symbols have undefined phase. **B** The number of samples categorized as follicular phase, luteal phase, or undefined by serum progesterone and by days since LMP. Squares are colored based on whether the methods categorized those samples as the same phase (green), opposite phases (orange), or one method was undefined (gray)

We next compared different methods of determining the menstrual cycle phase. Nine studies reported both days since the last menstrual period and serum progesterone levels. We used these studies to compare these two methods directly. Figure 8A shows all of the samples from those studies with their phases assigned by days since LMP (top) or by serum progesterone levels (bottom). Figure 8B shows that samples were rarely classified as opposite phases by the two methods: of the 535 samples that were assigned a phase (i.e., not undefined) by both methods, only 59 samples (11%) were assigned discordant phases. However, days since LMP lost many more samples to the undefined category. The two methods both designated 30 samples as undefined; an additional 130 were undefined by days since LMP, compared to only 62 by serum progesterone.

Menstrual phasing method did not have a consistent effect on the standard errors of the menstrual cycle effect sizes of individual immune factors across studies (Fig. S4A, difference between methods = 0.002, *p* = 0.87 by mixed model with study and immune factor as random effects, taken across all studies and immune factors). Within studies, the effect was consistent and dependent on sample size. In most studies, there were fewer undefined samples by serum progesterone than by days since LMP (for example, the studies Bradley, Cortez, and Hughes-unpublished). These studies tended to have lower standard errors in the analysis with phase determined by serum progesterone, consistent with the larger sample sizes in that analysis. Only one study had fewer undefined samples by days since LMP than by progesterone (Boily-Larouche). That study had lower standard errors in the analysis with phase determined by days since LMP. In addition, the effect sizes correlated well between the analyses performed with both phasing methods with Pearson r between 0.5 and 0.97 for all studies (not shown).

#### Normalization to total protein

We next wished to determine whether immune mediator concentrations should be normalized to the total concentration of protein in the samples. Normalization to total protein did not have a consistent effect on the standard errors of the menstrual cycle effect sizes (Fig. S4B, difference between normalized and non-normalized = 0.011, *p* = 0.67, mixed model with study and immune factor as random effects, taken across all studies and immune factors). In most studies, the standard errors were very similar whether the analysis was performed on raw or normalized concentrations. In addition, the effect sizes were very strongly correlated between normalized and raw concentrations with Pearson *r* > 0.9 for all studies (not shown).

## Discussion

### Summary

Our systematic review and meta-analyses of cervicovaginal immune mediators demonstrate clear and consistent changes across the menstrual cycle, the most striking being a widespread decrease in immune mediator concentrations in the luteal phase compared to the follicular phase. Chemokines, antibodies, MMPs, and several interleukins all decreased in the luteal phase, while only IL-1α and beta-defensins increased in the luteal phase. These cyclical differences may have consequences for immunity, susceptibility to infection, and fertility. We additionally identified immune mediators with stable levels across the cycle, and some requiring further research. Our study emphasizes the need to take the effect of the menstrual cycle into account in future studies and lays a foundation for future research to elucidate the biological basis for and consequences of these changes.

### Primary outcomes

We had high to moderate confidence that CC-type chemokines, antibodies, MMPs, IL-6, IL-16, IL-1RA, G-CSF, GNLY, and ICAM1 were lower in the luteal phase compared to the follicular phase. In contrast, there was high or moderate evidence of higher levels in the luteal phase for only three immune mediators: IL-1α, HBD-2, and HBD-3. There were also a large number of immune mediators where we have high to moderate confidence that levels change minimally between the phases: CXCL8, 9, and 10, interferons, TNF, SLPI, elafin, lysozyme, lactoferrin, and interleukins 1β, 2, 10, 12, 13, and 17A. In addition, we identified a number of immune mediators where additional research needs to be done due to low strength of evidence (Tables 3 and 4) or where the immune mediators were measured in only single studies (Table S1).

We conducted validation experiments for IgG, IL-1α, and CCL2. The directions of change were as predicted for all three and the differences were statistically significant for IL-1α and CCL2.

Our pre-specified sensitivity analyses supported the main outcomes of the primary analysis, adding confidence to our conclusions. In particular, there was little change in our results after adjusting for covariates, including BV and STIs. IPD were available for more than half of the studies we identified as potentially eligible. Access to IPD was a major benefit, because it allowed the analysis of all data in a uniform manner and enabled the inclusion of many studies where the published reports alone did not include sufficient information for meta-analysis.

### Biological significance of major differences between phases

CC-type chemokines were consistently reduced in the luteal phase, particularly those that bind to chemokine receptors 1, 2, 3, 5, and 6. These chemokines play roles in monocyte/macrophage and NK cell migration as well as Th2 and Th17 responses [64], suggesting recruitment of these cell types during the follicular phase. In addition, spermatozoa express chemokine receptors, such as CCR5 [65] and CCR6 [66], so chemokine expression in the CVT could be involved in regulation of sperm migration.

We observed a consistent pattern of immunoglobulins being reduced in the luteal phase, which is consistent with earlier studies [67, 68]. While it is clear that IgA can be produced locally in the CVT [69] and that systemic vaccination can induce antibody responses in the CVT [70–72], the antigens to which the majority of these antibodies react is unknown. The question of antibody specificity is of particular interest given the abundance of immunoglobulins in the CVT, the concentrations of which are orders of magnitude higher than most other immune mediators (Fig. 2).

The matrix metalloproteinases 1 and 7 were highly reduced in the luteal phase. These proteases degrade the extracellular matrix. In the uterus, they are important for remodeling of the endometrium during the cycle, in particular with breakdown of the lining during menses, and are tightly regulated by progesterone and cytokines [73]. Their role in the vaginal cavity is unclear, but their cyclical changes in expression in the vagina appear to match that seen in the endometrium [73].

The beta-defensins HBD-2 and HBD-3 were higher in the luteal phase, and among the most abundantly expressed immune mediators, suggesting a prominent role. These proteins are made by epithelial cells and disrupt microbial membranes. The mechanism for their induction during the luteal phase is unclear, as conflicting results have been observed with in vitro hormonal treatment of vaginal epithelial cells; presence of LPS could be involved [74, 75]. Increased levels of these antimicrobial effectors during the luteal phase may partially compensate for reduced levels of other immune mediators during that phase.

The other prominent increase in the luteal phase was of IL-1α. The IL-1 family as a whole underwent complex changes throughout the cycle: increase of IL-1α in the luteal phase combined with decrease of its antagonist IL-1RA suggests strong increases of IL-1α signaling in the luteal phase relative to the follicular phase. However, the decrease in IL-1RA is very small, with unclear biological significance. In addition, IL-1β had little to no change between the phases. The reason for this disconnect between IL-1α and IL-1β expression is unclear; perhaps it is related to IL-1α’s role in regulating MMP expression [73]. Notably, IL-1RA is the interleukin with the highest level of expression, dramatically higher than all other interleukins except IL-18.

A limitation of our study is the binary comparison between two narrowly defined phases of the menstrual cycle. While this approach was necessary for the study design, it obscures the fact that the cycle is a continuum made up of multiple different and overlapping biological processes, rather than two discrete phases.

### Subgroup analyses: sample type

We observed that sample type significantly modified the effect of the menstrual cycle: cyclical differences were much greater in menstrual cups and cervical sponges than in CVL and vaginal swabs. This result suggests that there are differences in the fluid collected by each sample type. These differences may include anatomical origin of the fluid (suggesting that the menstrual cycle has stronger effects in some areas of the CVT), effects of sample dilution, or differential presence of contaminating or interfering factors by sample type. Whatever the underlying explanation, this finding emphasizes the importance of sample type in understanding cyclical differences in CVT immune mediators.

### Detection of red blood cells/hemoglobin

The presence of red blood cells (RBCs) or hemoglobin was measured in nine studies. At a macroscopic level, blood was rare, with visual detection in only a few samples. However, microscopic levels were very common, present in over half of the samples, with a consistent pattern of higher levels during the follicular phase. Even in luteal phase samples, obtained long after the end of menstruation, over half of the samples were positive. Given this result, while it may make sense to exclude visibly bloody samples (if menstrual blood is not the subject of investigation), microscopic levels of blood may need to be regarded as a physiological characteristic of CVT fluid. Indeed, given the more frequent detection of RBCs during the follicular phase, the process underlying the presence of these cells may be part of the causal pathway of differences between phases and is therefore worthy of further study. Because blood was assessed in only a subset of the studies included here, it may be an undetected source of variability in the other studies, which should be assessed in future research.

### Secondary outcomes: detection levels and immune mediator concentrations

CVLs consistently yielded about five times lower immune mediator concentrations than menstrual cups, swabs, or sponges. This finding is expected, given the large volume of media used in the collection of a CVL, and confirms previous findings [20, 76, 77]. However, we saw no consistent difference in immune mediator concentrations between 5 and 10 mL CVLs. In cases where low abundance immune mediators are of primary interest, using a non-CVL sample will maximize detectability. In other cases, there are additional factors to take into account, such as the much higher sample volumes provided by CVL (allowing easier aliquoting and sharing), availability of clinical facilities, and participant preference.

We did not observe any consistent differences in immune mediator concentrations between ELISA and MSD assays. There was some indication that Luminex led to lower concentrations than ELISA, consistent with previous findings [78], but the differences were less consistent than for sample type. Differences between these assay methods are likely to depend more on the immune mediator (i.e., capture and detection antibody-dependent), than on the immunoassay platform.

There was no consistent effect of normalization to total protein, so it is unclear whether such normalization is beneficial. Notably, these observations were almost exclusively from studies using CVL. There was some suggestion of a benefit of normalizing to total protein for the two studies using non-CVL samples (swabs and menstrual cups), but more research is needed.

### Secondary outcomes: optimal phasing method

We found that our criteria for determining menstrual cycle phase by serum progesterone levels or by days since LMP led to similar results, with only 11% of samples categorized as opposite phases by the two methods. Effect sizes for differences between menstrual cycle phases were well correlated. Thus, both methods give consistent results. However, many more samples could not be assigned to a phase by days since LMP, leading to unused samples. Thus, serum progesterone allows a greater proportion of samples to be analyzed. It also allows for more flexibility in scheduling as compared to requiring participants to visit the clinic on a specific day of the cycle. However, measuring progesterone requires a blood draw, which is a disadvantage.

## Conclusions

Our unique study draws on work published in dozens of studies, performed by hundreds of investigators, with samples provided by thousands of participants, representing a remarkable collaboration of scientists from across the field. By collecting and re-analyzing IPD from these studies, we were able to leverage the information from those studies in a new way and make data from many of these studies available for future similar analyses in Additional file 3. We identified immune mediators with dynamic expression during the menstrual cycle as well as others that remain constant throughout. The decreases we observed in many immune mediators during the luteal phase are consistent with prior claims that immunity wanes during the luteal phase, likely creating a more tolerogenic environment for implantation of a semi-allogeneic embryo. In compensation, it appears that innate antimicrobial factors, such as beta-defensins, increase during the luteal phase. Lastly, we found that the magnitude of the cycle’s effect differs by sample type, which should be considered when choosing which type of samples to collect. Our findings open the door to many future research studies exploring the functional consequences of these changes.

## Supplementary Information

**Additional file 1.** Supporting methods documents. PRISMA-P and PRISMA-IPD checklists, database search strategies, manuscript screening form, risk of bias instrument, and strength of evidence tool.

**Additional file 2.** Amendments to the pre-registered protocol.

**Additional file 3.** Code and data. IPD from co-authors who agreed to share it, results of all analyses presented in the paper, and R code files necessary to reproduce the analysis and figures.

**Additional file 4. **Concentration and forest plots for each individual immune mediator. *Concentration plots* - Each symbol shows the concentration of the indicated immune mediator in a single sample. Each study is plotted separately. Lines connect samples from the same participant; in some cases participants provided multiple samples in the same phase, in which case multiple symbols within the same phase may be connected. Pale grey symbols are below the lower limit of detection and are assigned the value of half the lower limit of detection. *Forest plots* - Each row represents a different study, with the vertical line at the middle of each square indicating the mean and the horizontal line indicating the 95% confidence interval. Positive numbers indicate higher concentrations during the luteal phase (compared to the follicular phase), while negative numbers indicate lower concentrations during the luteal phase (compared to the follicular phase). The size of the squares is proportional to how heavily the study is weighted in the meta-analysis. The center of the diamond and the vertical dotted line indicates the meta-effect as determined by the random effects model. The width of the diamond indicates the 95% confidence interval of the meta-effect. A narrow diamond indicates small confidence intervals, a wide diamond indicates large confidence intervals. TE, treatment effect (log2-pg/mL of the luteal phase minus log2-pg/mL of the follicular phase); seTE, standard error of the treatment effect; 95%-CI, 95% confidence interval around the treatment effect; Weight, the percentage of the meta-estimate contributed by each study.

**Additional file 5: Figure S1.** Assessment of publication bias. **A** Funnel plots. Symbols show the effect of the menstrual cycle (x-axis) and the standard error of that effect (y-axis, reversed). Each symbol shows an individual study. Vertical solid line shows no effect. Vertical dashed line shows the meta-estimate of effect. Diagonal dashed lines enclose the region expected to include 95% of studies based on the estimated meta-effect and the standard errors. **B** Results of Egger’s tests for publication bias. **Figure S2.** Periovulatory meta-analyses. **A** The log2 difference between periovulatory and follicular phases (log2-pg/mL of the follicular phase minus log2-pg/mL of the periovulatory phase). For TGF-β1, the error bars for one study and the meta-estimate extend off-scale. **B** The log2 difference between periovulatory and luteal phases (log2-pg/mL of the luteal phase minus log2-pg/mL of the periovulatory phase). For IL-10, the error bars for one study extend off-scale. Each row represents a different immune mediator, with the symbols showing the mean and the lines showing the 95% confidence intervals. Gray symbols indicate individual studies and black the meta-estimates as determined by inverse-variance pooling random effects models. Black filled symbols indicate p < 0.05 while white filled symbols indicate p > 0.05. Positive numbers indicate higher during the follicular or luteal phase, while negative numbers indicate higher during the periovulatory phase. **Fig S3.** Subgroup analysis: Does the effect of menstrual cycle differ by assay method, geographical region, or method of determining menstrual phase? **A** Meta-analyses, comparing all studies (black circles) to studies grouped by assay method (ELISA: blue squares; MSD: yellow triangles; Luminex: green diamonds). **B** Meta-analyses, comparing all studies (black circles) to studies grouped by geographical region of sample origin (Africa: blue diamonds; Europe: red squares; North America: green triangles). **C** Meta-analyses, comparing all studies (black circles) to studies grouped by method of menstrual cycle phasing (Days since LMP: orange squares; Progesterone: pale purple diamonds; Progesterone plus LH: dark purple triangles). **Figure S4.** Secondary outcomes: Method of determining menstrual cycle phase and normalization to total protein. **A** The standard errors of the effect sizes for the difference between menstrual cycle phases, with phases determined by days since last menstrual period (“LMP”) or serum progesterone (“Prog”). Each symbol represents an immune factor, with lines connecting the same immune factor. **B** The standard errors of the effect sizes for the difference between menstrual cycle phases as determined using raw concentration measurements (pg/mL) and concentrations normalized to total protein (pg/pg total protein). Each symbol represents an immune factor, with lines connecting the same immune factor. **Table S1.** Summary of immune mediators measured in single studies. **Table S2.** Summary of follicular vs. periovulatory meta-analyses. **Table S3.** Summary of luteal vs. periovulatory meta-analyses. **Table S4.** Covariates adjusted for in multivariate analysis of each study.

## Data Availability

All R code is included in Additional file 3. All study-level and meta-analysis level outputs (effect of menstrual cycle at the study and meta-analysis level) are included in Additional file 3 to allow replication and updating of the meta-analysis in the future. Raw IPD is included in Additional file 3 for those studies where investigators agreed to release the data.

## Abbreviations

BCA: Bicinchoninic acid
BV: Bacterial vaginosis
CVL: Cervicovaginal lavage
CVT: Cervicovaginal tract
ELISA: Enzyme-linked immunosorbent assay
GRADE: Grading of recommendations, assessment, development and evaluations
HIV: Human immunodeficiency virus
IPD: Individual participant data
IUD: Intrauterine device
LH: Luteinizing hormone
LMP: Last menstrual period
LPS: Lipopolysaccharide
mIU/mL: Milli-international units per milliliter
MSD: Meso scale discovery
NK: Natural killer
pg/mL: Picogram per milliliter
PRISMA-P: Preferred Reporting Items for Systematic Review and Meta-Analysis Protocols
PRISMA-IPD: Preferred Reporting Items for Systematic Review and Meta-Analysis of Individual Participant Data
PSA: Prostate-specific antigen
RBC: Red blood cell
STI: Sexually transmitted infection
