# Supplementary material for: Changes in concentrations of cervicovaginal immune mediators across the menstrual cycle: a systematic review and meta-analysis of individual patient data
Source: BMC Med. 2022 Oct 5;20:353. doi: 10.1186/s12916-022-02532-9 (PMC9533580; doi:10.1186/s12916-022-02532-9)
Supplement: Supplementary file 1 — Additional file 1. Supporting methods documents. PRISMA-P and PRISMA-IPD checklists, database search strategies, manuscript screening form, risk of bias instrument, and strength of evidence tool. [file 12916_2022_2532_MOESM1_ESM.pdf]

# Additional File 1

Contents:

- PRISMA-P checklist
- PRISMA-IPD checklist
- Search strategies
- Manuscript screening form
- Risk of bias instrument
- Strength of evidence tool

# Additional File 1: PRISMA-P checklist

# PRISMA-P 2015 Checklist

This checklist has been adapted for use with protocol submissions to *Systematic Reviews* from Table 3 in Moher D et al: Preferred reporting items for systematic review and meta-analysis protocols (PRISMA-P) 2015 statement. *Systematic Reviews* 2015 **4**:1

| Section/topic | # | Checklist item | Information reported | | Line number(s) |
|---|---|---|---|---|---|
| | | | Yes | No | |
| **ADMINISTRATIVE INFORMATION** | | | | | |
| **Title** | | | | | |
| Identification | 1a | Identify the report as a protocol of a systematic review | ☒ | ☐ | 199 |
| Update | 1b | If the protocol is for an update of a previous systematic review, identify as such | ☐ | ☐ | NA |
| **Registration** | 2 | If registered, provide the name of the registry (e.g., PROSPERO) and registration number in the Abstract | ☐ | ☐ | NA |
| **Authors** | | | | | |
| Contact | 3a | Provide name, institutional affiliation, and e-mail address of all protocol authors; provide physical mailing address of corresponding author | ☒ | ☐ | 6-24 |
| Contributions | 3b | Describe contributions of protocol authors and identify the guarantor of the review | ☒ | ☐ | 28-35 |
| **Amendments** | 4 | If the protocol represents an amendment of a previously completed or published protocol, identify as such and list changes; otherwise, state plan for documenting important protocol amendments | ☒ | ☐ | 213-215 |
| **Support** | | | | | |
| Sources | 5a | Indicate sources of financial or other support for the review | ☒ | ☐ | 41-49 |
| Sponsor | 5b | Provide name for the review funder and/or sponsor | ☒ | ☐ | 41-49 |
| Role of sponsor/funder | 5c | Describe roles of funder(s), sponsor(s), and/or institution(s), if any, in developing the protocol | ☒ | ☐ | 41-49 |
| **INTRODUCTION** | | | | | |
| **Rationale** | 6 | Describe the rationale for the review in the context of what is already known | ☒ | ☐ | 144-177 |
| **Objectives** | 7 | Provide an explicit statement of the question(s) the review will address with reference to participants, interventions, comparators, and outcomes (PICO) | ☒ | ☐ | 179-195 |

BioMed Central
The Open Access Publisher

| Section/topic | # | Checklist item | Information reported | | Line number(s) |
|---|---|---|---|---|---|
| | | | Yes | No | |
| **METHODS** | | | | | |
| **Eligibility criteria** | 8 | Specify the study characteristics (e.g., PICO, study design, setting, time frame) and report characteristics (e.g., years considered, language, publication status) to be used as criteria for eligibility for the review | ☒ | ☐ | 217-241 |
| **Information sources** | 9 | Describe all intended information sources (e.g., electronic databases, contact with study authors, trial registers, or other grey literature sources) with planned dates of coverage | ☒ | ☐ | 243-249 |
| **Search strategy** | 10 | Present draft of search strategy to be used for at least one electronic database, including planned limits, such that it could be repeated | ☒ | ☐ | 251-257 |
| ***STUDY RECORDS*** | | | | | |
| Data management | 11a | Describe the mechanism(s) that will be used to manage records and data throughout the review | ☒ | ☐ | 260-274 |
| Selection process | 11b | State the process that will be used for selecting studies (e.g., two independent reviewers) through each phase of the review (i.e., screening, eligibility, and inclusion in meta-analysis) | ☒ | ☐ | 260-274 |
| Data collection process | 11c | Describe planned method of extracting data from reports (e.g., piloting forms, done independently, in duplicate), any processes for obtaining and confirming data from investigators | ☒ | ☐ | 276-298 |
| **Data items** | 12 | List and define all variables for which data will be sought (e.g., PICO items, funding sources), any pre-planned data assumptions and simplifications | ☒ | ☐ | 300-358 |
| **Outcomes and prioritization** | 13 | List and define all outcomes for which data will be sought, including prioritization of main and additional outcomes, with rationale | ☒ | ☐ | 360-379 |
| **Risk of bias in individual studies** | 14 | Describe anticipated methods for assessing risk of bias of individual studies, including whether this will be done at the outcome or study level, or both; state how this information will be used in data synthesis | ☒ | ☐ | 381-384 |
| ***DATA*** | | | | | |
| **Synthesis** | 15a | Describe criteria under which study data will be quantitatively synthesized | ☒ | ☐ | 387-389 |
| | 15b | If data are appropriate for quantitative synthesis, describe planned summary measures, methods of handling data, and methods of combining data from studies, including any planned exploration of consistency (e.g., $I^2$, Kendall's tau) | ☒ | ☐ | 391-419, 436-439 |
| | 15c | Describe any proposed additional analyses (e.g., sensitivity or subgroup analyses, meta-regression) | ☒ | ☐ | 421-434, 441-452 |
| | 15d | If quantitative synthesis is not appropriate, describe the type of summary planned | ☒ | ☐ | 454-456 |

| Section/topic | # | Checklist item | Information reported |  | Line number(s) |
|---|---|---|---|---|---|
| | | | Yes | No | |
| **Meta-bias(es)** | 16 | Specify any planned assessment of meta-bias(es) (e.g., publication bias across studies, selective reporting within studies) | ☒ | ☐ | 459-466 |
| **Confidence in cumulative evidence** | 17 | Describe how the strength of the body of evidence will be assessed (e.g., GRADE) | ☒ | ☐ | 472-491 |

BioMed Central
The Open Access Publisher

# Additional File 1: PRISMA-IPD checklist

**PRISMA-IPD Checklist of items to include when reporting a systematic review and meta-analysis of individual participant data (IPD)**

| PRISMA-IPD Section/topic | Item No | Checklist item | Reported on page |
|---|---|---|---|
| **Title** | | | |
| Title | 1 | Identify the report as a systematic review and meta-analysis of individual participant data. | 1 |
| **Abstract** | | | |
| Structured summary | 2 | Provide a structured summary including as applicable: | 6 |
| | | **Background**: state research question and main objectives, with information on participants, interventions, comparators and outcomes. | |
| | | **Methods**: report eligibility criteria; data sources including dates of last bibliographic search or elicitation, noting that IPD were sought; methods of assessing risk of bias. | |
| | | **Results**: provide number and type of studies and participants identified and number (%) obtained; summary effect estimates for main outcomes (benefits and harms) with confidence intervals and measures of statistical heterogeneity. Describe the direction and size of summary effects in terms meaningful to those who would put findings into practice. | |
| | | **Discussion:** state main strengths and limitations of the evidence, general interpretation of the results and any important implications. | |
| | | **Other:** report primary funding source, registration number and registry name for the systematic review and IPD meta-analysis. | |
| **Introduction** | | | |
| Rationale | 3 | Describe the rationale for the review in the context of what is already known. | 8 |
| Objectives | 4 | Provide an explicit statement of the questions being addressed with reference, as applicable, to participants, interventions, comparisons, outcomes and study design (PICOS). Include any hypotheses that relate to particular types of participant-level subgroups. | 8 |
| **Methods** | | | |
| Protocol and registration | 5 | Indicate if a protocol exists and where it can be accessed. If available, provide registration information including registration number and registry name. Provide publication details, if applicable. | 10 |
| Eligibility criteria | 6 | Specify inclusion and exclusion criteria including those relating to participants, interventions, comparisons, outcomes, study design and characteristics (e.g. years when conducted, required minimum follow-up). Note whether these were applied at the study or individual level i.e. whether eligible participants were included (and ineligible participants excluded) from a study that included a wider population than specified by the review inclusion criteria. The rationale for criteria should be stated. | 10 |
| Identifying studies - | 7 | Describe all methods of identifying published and unpublished studies including, as applicable: which bibliographic databases were searched with dates of coverage; details of any hand searching including of conference proceedings; use of study registers | 11 |

| | | | |
|---|---|---|---|
| information sources | | and agency or company databases; contact with the original research team and experts in the field; open adverts and surveys. Give the date of last search or elicitation. | |
| Identifying studies - search | 8 | Present the full electronic search strategy for at least one database, including any limits used, such that it could be repeated. | Additional File 1 |
| Study selection processes | 9 | State the process for determining which studies were eligible for inclusion. | 11 |
| Data collection processes | 10 | Describe how IPD were requested, collected and managed, including any processes for querying and confirming data with investigators. If IPD were not sought from any eligible study, the reason for this should be stated (for each such study). | 11-13 |
| | | If applicable, describe how any studies for which IPD were not available were dealt with. This should include whether, how and what aggregate data were sought or extracted from study reports and publications (such as extracting data independently in duplicate) and any processes for obtaining and confirming these data with investigators. | |
| Data items | 11 | Describe how the information and variables to be collected were chosen. List and define all study level and participant level data that were sought, including baseline and follow-up information. If applicable, describe methods of standardising or translating variables within the IPD datasets to ensure common scales or measurements across studies. | 13 |
| IPD integrity | A1 | Describe what aspects of IPD were subject to data checking (such as sequence generation, data consistency and completeness, baseline imbalance) and how this was done. | 11, 16 |
| Risk of bias assessment in individual studies. | 12 | Describe methods used to assess risk of bias in the individual studies and whether this was applied separately for each outcome. If applicable, describe how findings of IPD checking were used to inform the assessment. Report if and how risk of bias assessment was used in any data synthesis. | 14 |
| Specification of outcomes and effect measures | 13 | State all treatment comparisons of interests. State all outcomes addressed and define them in detail. State whether they were pre-specified for the review and, if applicable, whether they were primary/main or secondary/additional outcomes. Give the principal measures of effect (such as risk ratio, hazard ratio, difference in means) used for each outcome. | 13-14 |
| Synthesis methods | 14 | Describe the meta-analysis methods used to synthesise IPD. Specify any statistical methods and models used. Issues should include (but are not restricted to):<br>• Use of a one-stage or two-stage approach.<br>• How effect estimates were generated separately within each study and combined across studies (where applicable).<br>• Specification of one-stage models (where applicable) including how clustering of patients within studies was accounted for.<br>• Use of fixed or random effects models and any other model assumptions, such as proportional hazards.<br>• How (summary) survival curves were generated (where applicable).<br>• Methods for quantifying statistical heterogeneity (such as $I^2$ and $\tau^2$).<br>• How studies providing IPD and not providing IPD were analysed together (where applicable).<br>• How missing data within the IPD were dealt with (where applicable). | 14-15 |

| | | | |
|---|---|---|---|
| Exploration of variation in effects | A2 | If applicable, describe any methods used to explore variation in effects by study or participant level characteristics (such as estimation of interactions between effect and covariates). State all participant-level characteristics that were analysed as potential effect modifiers, and whether these were pre-specified. | 15 |
| Risk of bias across studies | 15 | Specify any assessment of risk of bias relating to the accumulated body of evidence, including any pertaining to not obtaining IPD for particular studies, outcomes or other variables. | 16 |
| Additional analyses | 16 | Describe methods of any additional analyses, including sensitivity analyses. State which of these were pre-specified. | 15 |
| **Results** | | | |
| Study selection and IPD obtained | 17 | Give numbers of studies screened, assessed for eligibility, and included in the systematic review with reasons for exclusions at each stage. Indicate the number of studies and participants for which IPD were sought and for which IPD were obtained. For those studies where IPD were not available, give the numbers of studies and participants for which aggregate data were available. Report reasons for non-availability of IPD. Include a flow diagram. | 19, Figure 1 |
| Study characteristics | 18 | For each study, present information on key study and participant characteristics (such as description of interventions, numbers of participants, demographic data, unavailability of outcomes, funding source, and if applicable duration of follow-up). Provide (main) citations for each study. Where applicable, also report similar study characteristics for any studies not providing IPD. | 19, Table 2 |
| IPD integrity | A3 | Report any important issues identified in checking IPD or state that there were none. | 19 |
| Risk of bias within studies | 19 | Present data on risk of bias assessments. If applicable, describe whether data checking led to the up-weighting or down-weighting of these assessments. Consider how any potential bias impacts on the robustness of meta-analysis conclusions. | Table 2 |
| Results of individual studies | 20 | For each comparison and for each main outcome (benefit or harm), for each individual study report the number of eligible participants for which data were obtained and show simple summary data for each intervention group (including, where applicable, the number of events), effect estimates and confidence intervals. These may be tabulated or included on a forest plot. | 19-20, Additional File 4 |
| Results of syntheses | 21 | Present summary effects for each meta-analysis undertaken, including confidence intervals and measures of statistical heterogeneity. State whether the analysis was pre-specified, and report the numbers of studies and participants and, where applicable, the number of events on which it is based. | 19-20, Fig 3, 5, S2 Tables 3, 4 Additional File 4 |
| | | When exploring variation in effects due to patient or study characteristics, present summary interaction estimates for each characteristic examined, including confidence intervals and measures of statistical heterogeneity. State whether the analysis was pre-specified. State whether any interaction is consistent across trials. | |
| | | Provide a description of the direction and size of effect in terms meaningful to those who would put findings into practice. | |

| | | | |
|---|---|---|---|
| Risk of bias across studies | 22 | Present results of any assessment of risk of bias relating to the accumulated body of evidence, including any pertaining to the availability and representativeness of available studies, outcomes or other variables. | 23-24, Tables 2, 3, Figure 7 |
| Additional analyses | 23 | Give results of any additional analyses (e.g. sensitivity analyses). If applicable, this should also include any analyses that incorporate aggregate data for studies that do not have IPD. If applicable, summarise the main meta-analysis results following the inclusion or exclusion of studies for which IPD were not available. | 22-26 |
| **Discussion** | | | |
| Summary of evidence | 24 | Summarise the main findings, including the strength of evidence for each main outcome. | 27 |
| Strengths and limitations | 25 | Discuss any important strengths and limitations of the evidence including the benefits of access to IPD and any limitations arising from IPD that were not available. | 27 |
| Conclusions | 26 | Provide a general interpretation of the findings in the context of other evidence. | 27-28 |
| Implications | A4 | Consider relevance to key groups (such as policy makers, service providers and service users). Consider implications for future research. | 27 |
| **Funding** | | | |
| Funding | 27 | Describe sources of funding and other support (such as supply of IPD), and the role in the systematic review of those providing such support. | 31 |

**A1 – A3 denote new items that are additional to standard PRISMA items. A4 has been created as a result of re-arranging content of the standard PRISMA statement to suit the way that systematic review IPD meta-analyses are reported.**

© Reproduced with permission of the PRISMA IPD Group, which encourages sharing and reuse for non-commercial purposes

# Additional File 1: Search strategies

*Search 1: PubMed*

((("Immunoproteins"[Mesh] OR "Cytokines"[Mesh] OR "Antimicrobial Cationic Peptides"[Mesh] OR "Immunoassay"[Mesh] OR immunoassay*[tiab] OR cytokine*[tiab] OR interleukin*[tiab] OR immunoprotein*[tiab] OR "immune mediator"[tiab] OR "immune mediators"[tiab] OR "immune biomarker"[tiab] OR "immune biomarkers"[tiab] OR "immune modulator"[tiab] OR "immune modulators"[tiab] OR "immune determinants"[tiab] OR "immune environment"[tiab] OR "immune microenvironment"[tiab] OR complement[tiab] OR immunoglobulin*[tiab] OR antibod*[tiab] OR chemokine* OR interferon* OR lymphokine* OR monokine* OR "tumor necrosis factor" OR "tumor necrosis factors" OR "transforming growth factor" OR "transforming growth factors" OR "antimicrobial peptides" OR "antimicrobial peptide" OR "antimicrobial polypeptide" OR "antimicrobial polypeptides" OR defensin OR defensins)

AND

("Menstrual Cycle"[Mesh] OR menstrua*[tiab] OR premenstrua*[tiab] OR cycling[tiab] OR "follicular phase" OR "follicular phases" OR "luteal phase" OR "luteal phases" OR "secretory phase" OR "secretory phases" OR "proliferative phase" OR "proliferative phases") AND ("Vagina"[Mesh] OR "Cervix Uteri"[Mesh] OR vagina*[tiab] OR cervicovaginal[tiab] OR cervix[tiab] OR cervical[tiab] OR endocervi*[tiab] OR ectocervi*[tiab] OR softcup[tiab] OR "weck cel"))

OR

("Menstrual Cycle/immunology"[Mesh] AND ("Vagina"[Mesh] OR "Cervix Uteri"[Mesh] OR vagina*[tiab] OR cervicovaginal[tiab] OR cervix[tiab] OR cervical[tiab] OR endocervi*[tiab] OR ectocervi*[tiab] OR softcup[tiab] OR "weck cel"))

OR

(("Menstrual Cycle"[Mesh] OR menstrua*[tiab] OR premenstrua*[tiab] OR cycling[tiab] OR "follicular phase" OR "follicular phases" OR "luteal phase" OR "luteal phases" OR "secretory phase" OR "secretory phases" OR "proliferative phase" OR "proliferative phases") AND ("Vagina/immunology"[Mesh] OR "Cervix Uteri/immunology"[Mesh]))

AND

("2000"[Date - Publication] : "3000"[Date - Publication]) AND "English"[la])

NOT

("animals"[mh] NOT "humans"[mh])

*Search 2: Embase*

(('immunoglobulin'/exp OR 'antibody'/exp OR 'complement'/exp OR 'C reactive protein'/exp OR 'eosinophil cationic protein'/exp OR 'eosinophil granule protein'/exp OR 'cytokine'/exp OR 'polypeptide antibiotic agent'/exp OR 'immunoassay'/exp OR "immune mediator":ti,ab OR "immune mediators":ti,ab OR "immune biomarker":ti,ab OR "immune biomarkers":ti,ab OR "immune modulator":ti,ab OR "immune modulators":ti,ab OR "immune determinants":ti,ab OR "immune environment":ti,ab OR "immune microenvironment":ti,ab)

AND

('menstrual cycle'/exp OR menstrua*:ti,ab OR premenstrua*:ti,ab OR cycling:ti,ab OR "follicular phase" OR "follicular phases" OR "luteal phase" OR "luteal phases" OR "secretory phase" OR "secretory phases" OR "proliferative phase" OR "proliferative phases")

AND

('vagina'/exp OR 'uterine cervix'/exp OR vagina*:ti,ab OR cervicovaginal:ti,ab OR cervix:ti,ab OR cervical:ti,ab OR endocervi*:ti,ab OR ectocervi*:ti,ab OR softcup:ti,ab OR 'weck cel')

AND

[english]/lim AND [2000-2020]/py)

NOT

(('nonhuman'/exp OR 'animal'/exp) NOT 'human'/exp)

*Search 3: Web of Science (SCI-EXPANDED index)*
TS=((immunoassay* OR cytokine OR cytokines OR interleukin* OR immunoprotein* OR "immune mediator" OR "immune mediators" OR "immune biomarker" OR "immune biomarkers" OR "immune modulator" OR "immune modulators" OR "immune determinants" OR "immune environment" OR "immune microenvironment" OR complement OR immunoglobulin* OR antibod* OR chemokine* OR interferon* OR lymphokine* OR monokine* OR "tumor necrosis factor" OR "tumor necrosis factors" OR "transforming growth factor" OR "transforming growth factors" OR "antimicrobial peptides" OR "antimicrobial peptide" OR "antimicrobial polypeptide" OR "antimicrobial polypeptides" OR defensin OR defensins) AND (menstrua* OR premenstrua* OR cycling OR "follicular phase" OR "follicular phases" OR "luteal phase" OR "luteal phases" OR "secretory phase" OR "secretory phases" OR "proliferative phase" OR "proliferative phases") AND (vagina* OR cervicovaginal OR cervix OR cervical OR endocervi* OR ectocervi* OR softcup OR "weck cel")) AND TS=(patient* OR women OR human OR clinical) *AND* LANGUAGE: (English)

*Search 4: Global Health Database*

(immunoassay* OR cytokine OR cytokines OR interleukin* OR immunoprotein* OR “immune mediator” OR “immune mediators” OR “immune biomarker” OR “immune biomarkers” OR “immune modulator” OR “immune modulators” OR “immune determinants” OR “immune environment” OR “immune microenvironment” OR complement OR immunoglobulin* OR antibod* OR chemokine* OR interferon* OR lymphokine* OR monokine* OR “tumor necrosis factor” OR “tumor necrosis factors” OR “transforming growth factor” OR “transforming growth factors” OR “antimicrobial peptides” OR “antimicrobial peptide” OR “antimicrobial polypeptide” OR “antimicrobial polypeptides” OR defensin OR defensins) AND (menstrua* OR premenstrua* OR cycling OR “follicular phase” OR “follicular phases” OR “luteal phase” OR “luteal phases” OR “secretory phase” OR “secretory phases” OR “proliferative phase” OR “proliferative phases”) AND (vagina* OR cervicovaginal OR cervix OR cervical OR endocervi* OR ectocervi* OR softcup OR “weck cel”)

# Additional File 1: Manuscript screening form

# Manuscript screening form

* Required

Reviewer name *

- Sean
- Claire

Study ID *

Your answer

Study title *

Your answer

## Meta-analysis eligibility criteria

You can stop if any answer is no.

Immune factor concentrations measured *

- Yes
- No

Antibody-based method *

Check all that apply or no

- [ ] ELISA
- [ ] Luminex (or other bead-based)
- [ ] MSD
- [ ] No
- [ ] Other:

Cervicovaginal secretion sample *

- ( ) CVL
- ( ) Menstrual cup
- ( ) Swab
- ( ) Weck-cel sponge
- ( ) No
- ( ) Other:

At least some participants are post-pubescent, pre-menopausal, non-pregnant *

- ( ) Yes
- ( ) No

At least some participants have no IUD, hormonal birth control, or other exogenous hormone use during the study *

- Yes
- No

At least some participants have no vaginal intervention of any kind (including a placebo). No treatment, systemic placebo, and baseline (pre-treatment) are allowed *

- Yes
- No

Samples are available from both the follicular and luteal phases *

- Yes
- No

Next

Never submit passwords through Google Forms.

This content is neither created nor endorsed by Google. Report Abuse - Terms of Service - Privacy Policy

Google Forms

# Manuscript screening form

* Required

## Study information

You don't need to do this section if you answered "no" to any question in the previous section.

### Population

For example, "women of reproductive age from Seattle"

Your answer

### Countr(ies) of sample origin

Your answer

### Age range

For example, "18-35." Report actual range rather than allowed range if both are available.

Your answer

### Inclusion criteria

Your answer

Exclusion criteria

Your answer

Method of determination of menstrual phase

Choose hormone levels if date and hormone levels were used

- ◯ Hormone levels: progesterone
- ◯ Hormone levels: progesterone and estrogen
- ◯ Hormone levels: LH and progesterone
- ◯ Hormone levels: LH and progesterone and estrogen
- ◯ Date of last menstrual period
- ◯ Not reported
- ◯ Other:

Method of determination of menstrual phase: sample type

- ◯ Blood (serum or plasma)
- ◯ Urine
- ◯ Not applicable
- ◯ Other:

Method of determination of menstrual phase: exact wording *

Copy/paste in from the text of the manuscript how they defined menstrual cycle

Your answer

Required cycle length *

Copy/paste in what they required of the cycle length to be included in the study (e.g. "24-35 day cycle" or "regular menses for the last 3 months". If none, put "None"

Your answer

Limits of detection

If limits of detection are reported, specify where (page or table number)

Your answer

Total protein concentrations measured?

- Yes
- No
- Unclear

If the sample type was a swab or sponge, from where was the sample taken?

- Ectocervix
- Endocervix
- Vagina
- Unspecified
- Not applicable (sample type was not sponge)

If the sample type was a CVL, was the CVL collected by the clinician or the participant?

- ◯ Clinician
- ◯ Participant
- ◯ Unspecified
- ◯ Not applicable (sample type was not CVL)

If the sample type was a CVL, what was the wash medium?

- ◯ PBS
- ◯ HBSS
- ◯ Saline
- ◯ Unspecified
- ◯ Not applicable (sample type was not CVL)
- ◯ Other:

If the sample type was a CVL, what was the volume of lavage?

- ○ 3 mL
- ○ 5 mL
- ○ 10 mL
- ○ 15 mL
- ○ Unspecified
- ○ Not applicable (sample type was not CVL)
- ○ Other:

### Covariates reported

Report here if covariate is used in the data analysis, report in inclusion/exclusion criteria otherwise

- [ ] Bacterial vaginosis (see question below)
- [ ] Microbiome
- [ ] STI status (see question below)
- [ ] Recent sexual contact (participant report)
- [ ] Recent sexual contact (PSA or other assay)
- [ ] Condom use
- [ ] Vaginal washing
- [ ] Race/ethnicity
- [ ] Vaginal pH
- [ ] Cervical mucus
- [ ] Discharge present
- [ ] Epithelial abnormalities
- [ ] Hemoglobin or blood contamination of samples
- [ ] Other:

If STIs were measured, which?

- [ ] Chlamydia
- [ ] Gonorrhea
- [ ] HIV
- [ ] HPV
- [ ] HSV-1/2
- [ ] Trichomonas
- [ ] Yeast
- [ ] Other:

If BV was measured, what method?

- ○ Nugent
- ○ Amsel
- ○ Microbiome measurement
- ○ Not reported
- ○ Other:

Statistical methodology for follicular vs. luteal comparison

- Linear mixed effects model
- Generalized estimating equation
- T-test
- Not applicable
- Other:

If follicular vs. luteal comparisons are reported as numbers, list here.

<analyte name>; <effect size>; SE:<standard error>; CI:<lower>-<upper>. For example: IL1a; 2.5; 1.2; 0.15-4.85. Include all of the semi-colons, just leave a space if data is missing for a field. One analyte per line.

Your answer

List any relevant references from the bibliography

Your answer

Back Next

Never submit passwords through Google Forms.

This content is neither created nor endorsed by Google. Report Abuse - Terms of Service - Privacy Policy

Google Forms

# Manuscript screening form

Risk of bias

You don't need to complete this section if you answered "no" to any question in the first section.

Representativeness of the follicular cohort, maximum of one point

- ◯ Truly representative of the average woman in the community *
- ◯ Somewhat representative of the average woman in the community *
- ◯ Selected group of women, eg. sex workers, common clinical condition
- ◯ No description of the derivation of the follicular cohort

Selection of the luteal cohort, maximum of one point

- ◯ Drawn from the same community as the follicular cohort *
- ◯ Drawn from a different source
- ◯ No description of the derivation of the luteal cohort

Ascertainment of menstrual phase, maximum of one point

- ◯ Independent blind assessment (e.g. hormone levels) *
- ◯ Participant self-report
- ◯ Other:

Comparability of the cohorts (on basis of design or analysis), maximum of two points

- ◯ Study assesses bacterial vaginosis and vulvovaginal candidiasis *
- ◯ Study assesses active bleeding at time of sample collection *
- ◯ Study assesses active STI infection *
- ◯ Study assesses other important factors identified by the investigators as relevant for their cohort * (e.g. HPV, HIV, cervical dysplasia)
- ◯ Study doesn't asses any important factors

Assessment of outcome, maximum of one point

- ◯ Results of all immune mediators measured in the study were reported *
- ◯ Only selected immune mediators were reported

Back Submit

Never submit passwords through Google Forms.

This content is neither created nor endorsed by Google. Report Abuse - Terms of Service - Privacy Policy

Google Forms

# Additional File 1: Risk of bias instrument

## Risk of bias instrument

This instrument was adapted from the Newcastle-Ottawa Scale for cohort studies (http://www.ohri.ca/programs/clinical_epidemiology/nosgen.pdf) with these modifications:

- Selection Q3 was adapted to reflect common methods of ascertaining menstrual phase
- Selection Q4 was omitted for irrelevance
- Comparability Q1 changes "controls for" to "assesses" because controlling at a statistical level will be done in analysis of IPD.
- Outcome Q1 was rephrased to better reflect outcome reporting.
- Outcome Q2 and Q3 were omitted for irrelevance.

The instrument was combined into the data extraction and screening form (Additional File 3) for convenience.

Answers marked with * are worth one point. Answers without * are worth zero. Total possible points: 6
4-6 points = low risk of bias
2-3 points = medium risk of bias
0-1 points = high risk of bias

Selection

1. Representativeness of the follicular cohort, maximum of one point
   a. Truly representative of the average woman in the community *
   b. Somewhat representative of the average woman in the community *
   c. Selected group of women, eg. sex workers, common clinical condition
   d. No description of the derivation of the follicular cohort
2. Selection of the luteal cohort, maximum of one point
   a. Drawn from the same community as the follicular cohort *
   b. Drawn from a different source
   c. No description of the derivation of the luteal cohort
3. Ascertainment of menstrual phase, maximum of one point
   a. Independent blind assessment (e.g. hormone levels) *
   b. Participant self-report
   c. Other

Comparability

1. Comparability of the cohorts (on basis of design or analysis), maximum of two points
   a. Study assesses bacterial vaginosis and vulvovaginal candidiasis *
   b. Study assesses active bleeding at time of sample collection *
   c. Study assesses active STI infection *
   d. Study assesses other important factors identified by the investigators as relevant for their cohort * (e.g. HPV, HIV, cervical dysplasia)
   e. Study doesn't asses any important factors

Outcome

1. Assessment of outcome, maximum of one point
   a. Results of all immune mediators measured in the study were reported *
   b. Only selected immune mediators were reported

# Additional File 1: Strength of evidence tool

# Strength of evidence (GRADE)

* Required

1. Evaluator *

*Mark only one oval.*

- Claire
- Sean

2. Immune mediator *

______________________________

## Downgrading domains

The next five domains can lead to reductions in the strength of evidence

3. Domain 1: Risk of bias *

Risk of bias will be assessed separately for each study using the Risk of Bias tool, specifically the total risk of bias score. An overall estimate of risk of bias will then be made for each immune mediator taking into account the risk of bias across studies.

*Mark only one oval.*

- Not serious (no downgrade)
- Serious (downgrade 1 level)
- Very serious (downgrade 2 levels)

4. Domain 1: Risk of bias (rationale) *

5. Domain 2: Inconsistency *

Inconsistency will be assessed by looking for unexplained heterogeneity, considering whether there is variation in the size of effect (study point estimates vary widely), whether the study-level CIs overlap, whether the statistical test for heterogeneity has $p < 0.05$, and whether I2 is large. If subgroup analysis can explain heterogeneity, we may not downgrade for inconsistency.

*Mark only one oval.*

- Not serious (no downgrade)
- Serious (downgrade 1 level)
- Very serious (downgrade 2 levels)

6. Domain 2: Inconsistency (rationale) *

7. Domain 3: Indirectness *

Indirectness will be assessed by considering whether the evidence comes from the population of interest (women), whether the comparisons are relevant to our question of interest (direct follicular vs. luteal phase comparison), and whether the outcomes of interest (immune mediator concentration) are directly measured.

*Mark only one oval.*

- Not serious (no downgrade)
- Serious (downgrade 1 level)
- Very serious (downgrade 2 levels)

8. Domain 3: Indirectness (rationale) *

9. Domain 4: Imprecision *

Imprecision will be assessed by considering whether the sample size is large enough and the confidence intervals are small enough. This assessment will be based on comparing the total number of samples in the follicular and luteal groups across all studies to a power analysis of how big a single study would need to be to detect the observed meta-effect (when one exists).

*Mark only one oval.*

- Not serious (no downgrade)
- Serious (downgrade 1 level)
- Very serious (downgrade 2 levels)

10. Domain 4: Imprecision (rationale) *

11. Domain 5: Publication bias *

Publication bias will be assessed as described in the methods, using funnel plots and Egger's test where at least ten studies exist for a particular immune mediator.

*Mark only one oval.*

- Undetected (no downgrade)
- Strongly suspected (downgrade 1 level)
- Very strongly suspected (downgrade 2 levels)

12. Domain 5: Publication bias (rationale) *

## Upgrading domains

The next domains can lead to increases in the strength of evidence.

13. Domain 1: Large magnitude of effect *

The evidence can be upgraded if there is a very large magnitude of effect. We define large as 5-fold and very large as 10-fold

*Mark only one oval.*

- ( ) Normal magnitude of effect (no upgrade)
- ( ) Large (five-fold) magnitude of effect (upgrade 1 level)
- ( ) Very large (ten-fold) magnitude of effect (upgrade 2 levels)

14. Domain 1: Large magnitude of effect (rationale) *

15. Domain 2: Removing residual confounding would strengthen evidence *

The evidence can be upgraded if an effect is observed and all plausible residual confounding is likely to be reducing the effect (i.e. you're observing an effect despite the fact that the confounding is making it look smaller). It can also be upgraded if no effect is observed and all plausible residual confounding would suggest a spurious effect (i.e. you didn't observe an effect despite the fact that the confounding would make it look like there were an effect).

*Mark only one oval.*

- ( ) No upgrade
- ( ) An effect is observed and all plausible residual confounding is likely to be reducing the effect (upgrade 1 level)
- ( ) No effect is observed and all plausible residual confounding would suggest a spurious effect (upgrade 1 level)

16. Domain 2: Removing residual confounding would strengthen evidence (rationale) *

## Summary measures

17. Number of downgrades *

18. Number of upgrades *

19. Overall strength of evidence *

*Mark only one oval.*

| | 1 | 2 | 3 | 4 | |
|---|---|---|---|---|---|
| Very low | ( ) | ( ) | ( ) | ( ) | High |

This content is neither created nor endorsed by Google.

Google Forms
