# Supplementary material for: Changes in concentrations of cervicovaginal immune mediators across the menstrual cycle: a systematic review and meta-analysis of individual patient data
Source: BMC Med. 2022 Oct 5;20:353. doi: 10.1186/s12916-022-02532-9 (PMC9533580; doi:10.1186/s12916-022-02532-9)
Supplement: Supplementary file 2 — Additional file 2. Amendments to the pre-registered protocol. [file 12916_2022_2532_MOESM2_ESM.docx]

# Amendments to the protocol

Several changes and corrections to our pre-registered study protocol were necessary. These amendments are described below.

June 7, 2021—Progesterone level measurements: We proposed to use an ELISA to measure serum progesterone concentrations for the exploratory and validation experiments. Initial testing of the ELISA indicated that matrix effects from the serum were causing inaccurately high concentrations. After consultation with experts, we replaced the ELISA with a liquid-chromatography mass spectrometry assay performed at the Endocrine Technology Core at Oregon National Primate Research Center at Oregon Health and Science University.

March 10, 2022—Sample type and assay method: As a secondary outcome, we proposed to compare the concentrations and detectability of immune mediators by sample type and assay method. We incorrectly stated that we would use meta-regression for these analyses, which is not applicable to this question. In fact, the correct method is mixed effects regression with random effects of study and participant.

March 16, 2022—Meta-regression: We proposed to perform multiple meta-regression for assay type, sample type, method of determining menstrual phase, and geographical region. However, the number of studies was insufficient to meaningfully assess the effect of each of these factors simultaneously by multiple meta-regression. We instead explored these factors separately using subgroup analysis.

April 1, 2022—Exclusion of data sets: In three cases, particular immune mediators were poorly detected in one study, but well detected in other studies. The three cases were IFNA2 from Hughes-unpublished, IL17A from Makinde-2018, and TNF from Hwang-2011. The low detection in these studies caused a very low standard error for those factors in these studies. The low standard errors resulted in their being highly weighted in the meta-analysis (>50%), much more highly than studies with larger sample sizes. We chose to remove these three cases, so the other studies would be weighted appropriately. We did not anticipate this issue and it was not part of our pre-registered analysis plan. In all three cases, the meta-estimates changed by less than 0.1 (log2 scale), retained the same direction of change, and their statistical significance did not change.
