# Supplementary material for: Changes in concentrations of cervicovaginal immune mediators across the menstrual cycle: a systematic review and meta-analysis of individual patient data
Source: BMC Med. 2022 Oct 5;20:353. doi: 10.1186/s12916-022-02532-9 (PMC9533580; doi:10.1186/s12916-022-02532-9)
Supplement: Supplementary file 3 — Additional file 3. Code and data. IPD from co-authors who agreed to share it, results of all analyses presented in the paper, and R code files necessary to reproduce the analysis and figures. [file 12916_2022_2532_MOESM3_ESM.zip › MenstrualCycleMetaanalysis/README.html]

README


# README

## Data availability

The data is available in the `data` folder.

The raw data from each study is available in `data\data_complete\01_study`. The files all have a consistent format, where each row represents a single measurement. This means that each sample appears in multiple rows (one for each cytokine that was measured for that sample). The columns are defined in `data\data_helper_files\data_schema.xlsx`. Note: Not every study author agreed to publish the raw data from their study, so raw data only appears for some studies.

The results of all analyses are in the other `data\data_complete\` subfolders.

Additional data files are contained in `data\data_helper_files`.

## Reproducing the analysis

The rest of this document describes how to reproduce the data analysis and figures.

All data go into subfolders of this folder.

## Running the analysis

Open `R/source_all.R` and install all of the packages listed there.

Open `make_paper.R`. Assuming the working directory is the same as the directory containing this file, the file paths will all be correct. To reproduce the analysis, source `make_paper.R`. This will run code to:

- Perform statistical analysis
- Generate all figures
- Generate all statistics reported in the manuscript

It will take a while to run.

Questions? smhughes@uw.edu

## Important caveat

Not all authors agreed to publish the raw data from their studies. To reproduce the statistics and figures, we have therefore provided the results of all analyses performed on the complete data set in `data\data_complete`. When you run the code, it will generate the figures and statistics using those files.

To show how those results were generated, the code will also rerun all analyses on the partial data set (all studies where authors agreed to share the data) and save the results into `data\data_partial`. These results will differ from the results reported in the paper and from the results in `data\data_complete` because they exclude studies where authors did not agree to publish the raw data. The purpose of these files is to show how the results were generated.

## File and directory summary

- `data/` contains the data.
  - `data_complete/` contains the results of the analyses performed on the complete data set
  - `data_partial/` contains the results of the analyses performed on the published data set (see caveat section above)
  - `data_helper_files` contains additional data files
- `figures/images` contains the figures
- `make_paper.R` runs all the code necessary to reproduce the analysis and generate the figures
- `R/` contains code files
  - `source_all.R` loads all the packages and helper functions
  - `helpers_*.R` load helper functions
  - `01_*.R` to `10_.Rmd` run all the analyses and generate the figures

## Session info

The analysis for the paper was run using the following versions of R and the packages:

```
## - Session info -----------------------------------------------------------------------------------
##  setting  value                       
##  version  R version 4.0.0 (2020-04-24)
##  os       Windows 10 x64              
##  system   x86_64, mingw32             
##  ui       RTerm                       
##  language (EN)                        
##  collate  English_United States.1252  
##  ctype    English_United States.1252  
##  tz       America/Los_Angeles         
##  date     2022-06-27                  
## 
## - Packages ---------------------------------------------------------------------------------------
##  package      * version    date       lib source                                    
##  assertthat     0.2.1      2019-03-21 [1] CRAN (R 4.0.0)                            
##  backports      1.1.6      2020-04-05 [1] CRAN (R 4.0.0)                            
##  boot           1.3-24     2019-12-20 [2] CRAN (R 4.0.0)                            
##  broom          0.7.8      2021-06-24 [1] CRAN (R 4.0.5)                            
##  cachem         1.0.5      2021-05-15 [1] CRAN (R 4.0.5)                            
##  cellranger     1.1.0      2016-07-27 [1] CRAN (R 4.0.0)                            
##  cli            3.0.0      2021-06-30 [1] CRAN (R 4.0.5)                            
##  colorspace     1.4-1      2019-03-18 [1] CRAN (R 4.0.0)                            
##  CompQuadForm   1.4.3      2017-04-12 [1] CRAN (R 4.0.0)                            
##  conflicted   * 1.0.4      2019-06-21 [1] CRAN (R 4.0.0)                            
##  crayon         1.3.4      2017-09-16 [1] CRAN (R 4.0.0)                            
##  DBI            1.1.0      2019-12-15 [1] CRAN (R 4.0.0)                            
##  dbplyr         1.4.3      2020-04-19 [1] CRAN (R 4.0.0)                            
##  digest         0.6.25     2020-02-23 [1] CRAN (R 4.0.0)                            
##  dplyr        * 1.0.7      2021-06-18 [1] CRAN (R 4.0.5)                            
##  ellipsis       0.3.2      2021-04-29 [1] CRAN (R 4.0.5)                            
##  evaluate       0.14       2019-05-28 [1] CRAN (R 4.0.0)                            
##  fansi          0.4.1      2020-01-08 [1] CRAN (R 4.0.0)                            
##  fastmap        1.1.0      2021-01-25 [1] CRAN (R 4.0.5)                            
##  forcats      * 0.5.0      2020-03-01 [1] CRAN (R 4.0.0)                            
##  fs             1.5.0      2020-07-31 [1] CRAN (R 4.0.5)                            
##  generics       0.1.0      2020-10-31 [1] CRAN (R 4.0.5)                            
##  ggplot2      * 3.3.5      2021-06-25 [1] CRAN (R 4.0.5)                            
##  glue           1.4.0      2020-04-03 [1] CRAN (R 4.0.0)                            
##  gtable         0.3.0      2019-03-25 [1] CRAN (R 4.0.0)                            
##  haven          2.2.0      2019-11-08 [1] CRAN (R 4.0.0)                            
##  here         * 0.1        2017-05-28 [1] CRAN (R 4.0.0)                            
##  hms            0.5.3      2020-01-08 [1] CRAN (R 4.0.0)                            
##  htmltools      0.5.1.1    2021-01-22 [1] CRAN (R 4.0.5)                            
##  httr           1.4.2      2020-07-20 [1] CRAN (R 4.0.5)                            
##  janitor      * 2.0.1      2020-04-12 [1] CRAN (R 4.0.0)                            
##  jsonlite       1.6.1      2020-02-02 [1] CRAN (R 4.0.0)                            
##  knitr          1.28       2020-02-06 [1] CRAN (R 4.0.0)                            
##  lattice        0.20-41    2020-04-02 [2] CRAN (R 4.0.0)                            
##  lifecycle      1.0.0      2021-02-15 [1] CRAN (R 4.0.5)                            
##  lme4         * 1.1-23     2020-04-07 [1] CRAN (R 4.0.0)                            
##  lmerTest     * 3.1-2      2020-04-08 [1] CRAN (R 4.0.0)                            
##  lubridate    * 1.7.8      2020-04-06 [1] CRAN (R 4.0.0)                            
##  magrittr       1.5        2014-11-22 [1] CRAN (R 4.0.0)                            
##  MASS           7.3-51.5   2019-12-20 [2] CRAN (R 4.0.0)                            
##  mathjaxr       1.4-0      2021-03-01 [1] CRAN (R 4.0.5)                            
##  Matrix       * 1.3-4      2021-06-01 [1] CRAN (R 4.0.5)                            
##  memoise        2.0.0      2021-01-26 [1] CRAN (R 4.0.5)                            
##  meta         * 5.0-1      2021-10-20 [1] CRAN (R 4.0.5)                            
##  metaDigitise * 1.0.1      2021-07-21 [1] Github (daniel1noble/metaDigitise@55d44f3)
##  metafor        3.0-2      2021-06-09 [1] CRAN (R 4.0.5)                            
##  minqa          1.2.4      2014-10-09 [1] CRAN (R 4.0.0)                            
##  modelr         0.1.7      2020-04-30 [1] CRAN (R 4.0.0)                            
##  munsell        0.5.0      2018-06-12 [1] CRAN (R 4.0.0)                            
##  nlme         * 3.1-147    2020-04-13 [2] CRAN (R 4.0.0)                            
##  nloptr         1.2.2.1    2020-03-11 [1] CRAN (R 4.0.0)                            
##  numDeriv       2016.8-1.1 2019-06-06 [1] CRAN (R 4.0.0)                            
##  paletteer    * 1.3.0      2021-01-06 [1] CRAN (R 4.0.4)                            
##  pander       * 0.6.3      2018-11-06 [1] CRAN (R 4.0.0)                            
##  patchwork    * 1.0.0      2019-12-01 [1] CRAN (R 4.0.0)                            
##  pillar         1.6.1      2021-05-16 [1] CRAN (R 4.0.5)                            
##  pkgconfig      2.0.3      2019-09-22 [1] CRAN (R 4.0.0)                            
##  plater       * 1.0.3      2022-01-26 [1] local                                     
##  purrr        * 0.3.4      2020-04-17 [1] CRAN (R 4.0.0)                            
##  R6             2.4.1      2019-11-12 [1] CRAN (R 4.0.0)                            
##  Rcpp           1.0.7      2021-07-07 [1] CRAN (R 4.0.5)                            
##  readr        * 1.3.1      2018-12-21 [1] CRAN (R 4.0.0)                            
##  readxl       * 1.3.1      2019-03-13 [1] CRAN (R 4.0.0)                            
##  rematch2       2.1.2      2020-05-01 [1] CRAN (R 4.0.0)                            
##  reprex         0.3.0      2019-05-16 [1] CRAN (R 4.0.0)                            
##  rlang          0.4.11     2021-04-30 [1] CRAN (R 4.0.5)                            
##  rmarkdown      2.1        2020-01-20 [1] CRAN (R 4.0.0)                            
##  rprojroot      1.3-2      2018-01-03 [1] CRAN (R 4.0.0)                            
##  rstudioapi     0.13       2020-11-12 [1] CRAN (R 4.0.5)                            
##  rvest          0.3.5      2019-11-08 [1] CRAN (R 4.0.0)                            
##  scales         1.1.1      2020-05-11 [1] CRAN (R 4.0.0)                            
##  sessioninfo    1.1.1      2018-11-05 [1] CRAN (R 4.0.0)                            
##  snakecase      0.11.0     2019-05-25 [1] CRAN (R 4.0.0)                            
##  statmod        1.4.34     2020-02-17 [1] CRAN (R 4.0.0)                            
##  stringi        1.4.6      2020-02-17 [1] CRAN (R 4.0.0)                            
##  stringr      * 1.4.0      2019-02-10 [1] CRAN (R 4.0.0)                            
##  tibble       * 3.1.2      2021-05-16 [1] CRAN (R 4.0.5)                            
##  tidyr        * 1.1.3      2021-03-03 [1] CRAN (R 4.0.5)                            
##  tidyselect     1.1.0      2020-05-11 [1] CRAN (R 4.0.0)                            
##  tidyverse    * 1.3.0      2019-11-21 [1] CRAN (R 4.0.0)                            
##  utf8           1.1.4      2018-05-24 [1] CRAN (R 4.0.0)                            
##  vctrs          0.3.8      2021-04-29 [1] CRAN (R 4.0.5)                            
##  withr          2.4.2      2021-04-18 [1] CRAN (R 4.0.5)                            
##  xfun           0.13       2020-04-13 [1] CRAN (R 4.0.0)                            
##  xml2           1.3.2      2020-04-23 [1] CRAN (R 4.0.0)                            
##  yaml           2.2.1      2020-02-01 [1] CRAN (R 4.0.0)                            
## 
## [1] C:/Users/smhughes/Documents/R/win-library/4.0
## [2] C:/Program Files/R/R-4.0.0/library
```
