# Supplementary material for: Changes in concentrations of cervicovaginal immune mediators across the menstrual cycle: a systematic review and meta-analysis of individual patient data
Source: BMC Med. 2022 Oct 5;20:353. doi: 10.1186/s12916-022-02532-9 (PMC9533580; doi:10.1186/s12916-022-02532-9)
Supplement: Supplementary file 4 — Additional file 4. Concentration and forest plots for each individual immune mediator. Concentration plots - Each symbol shows the concentration of the indicated immune mediator in a single sample. Each study is plotted separately. Lines connect samples from the same participant; in some cases participants provided multiple samples in the same phase, in which case multiple symbols within the same phase may be connected. Pale grey symbols are below the lower limit of detection and are assigned the value of half the lower limit of detection. Forest plots - Each row represents a different study, with the vertical line at the middle of each square indicating the mean and the horizontal line indicating the 95% confidence interval. Positive numbers indicate higher concentrations during the luteal phase (compared to the follicular phase), while negative numbers indicate lower concentrations during the luteal phase (compared to the follicular phase). The size of the squares is proportional to how heavily the study is weighted in the meta-analysis. The center of the diamond and the vertical dotted line indicates the meta-effect as determined by the random effects model. The width of the diamond indicates the 95% confidence interval of the meta-effect. A narrow diamond indicates small confidence intervals, a wide diamond indicates large confidence intervals. TE, treatment effect (log2-pg/mL of the luteal phase minus log2-pg/mL of the follicular phase); seTE, standard error of the treatment effect; 95%-CI, 95% confidence interval around the treatment effect; Weight, the percentage of the meta-estimate contributed by each study. [file 12916_2022_2532_MOESM4_ESM.pdf]

Comprehensive overview of each immune factor

Log2-fold difference between luteal and follicular phase

For each immune mediator, there are two pages. The first page shows the individual participant data (concentrations of immune mediators) for each sample in each study. The second page shows a forest plot, showing the difference between luteal and follicular phase for each study separately.

Notes:

- The Shust-2010 study, which appears in some forest plots, does not appear in the concentration plots. This is because individual participant data was unavailable for this study.
- New wet lab data indicates the exploratory and validation measurements performed for this meta-analysis, as described in the results section.

Concentration plots

Each symbol shows the concentration of the indicated immune mediator in a single sample. Each study is plotted separately. Lines connect samples from the same participant; in some cases participants provided multiple samples in the same phase, in which case multiple symbols within the same phase may be connected. Pale grey symbols are below the lower limit of detection and are assigned the value of half the lower limit of detection.

Forest plots

This section shows the meta-analyses for each immune factor where the concentrations of at least half of the samples fell above the limit of detection.

Each row represents a different study, with the vertical line at the middle of each square indicating the mean and the horizontal line indicating the 95% confidence interval. Positive numbers indicate higher concentrations during the luteal phase (compared to the follicular phase), while negative numbers indicate lower concentrations during the luteal phase (compared to the follicular phase). The size of the squares is proportional to how heavily the study is weighted in the meta-analysis.

The center of the diamond and the vertical dotted line indicates the meta-effect as determined by the random effects model. The width of the diamond indicates the 95% confidence interval of the meta-effect. A narrow diamond indicates small confidence intervals, a wide diamond indicates large confidence intervals.

TE, treatment effect (log2-pg/mL of the luteal phase minus log2-pg/mL of the follicular phase); seTE, standard error of the treatment effect; 95%-CI, 95% confidence interval around the treatment effect; Weight, the percentage of the meta-estimate contributed by each study.

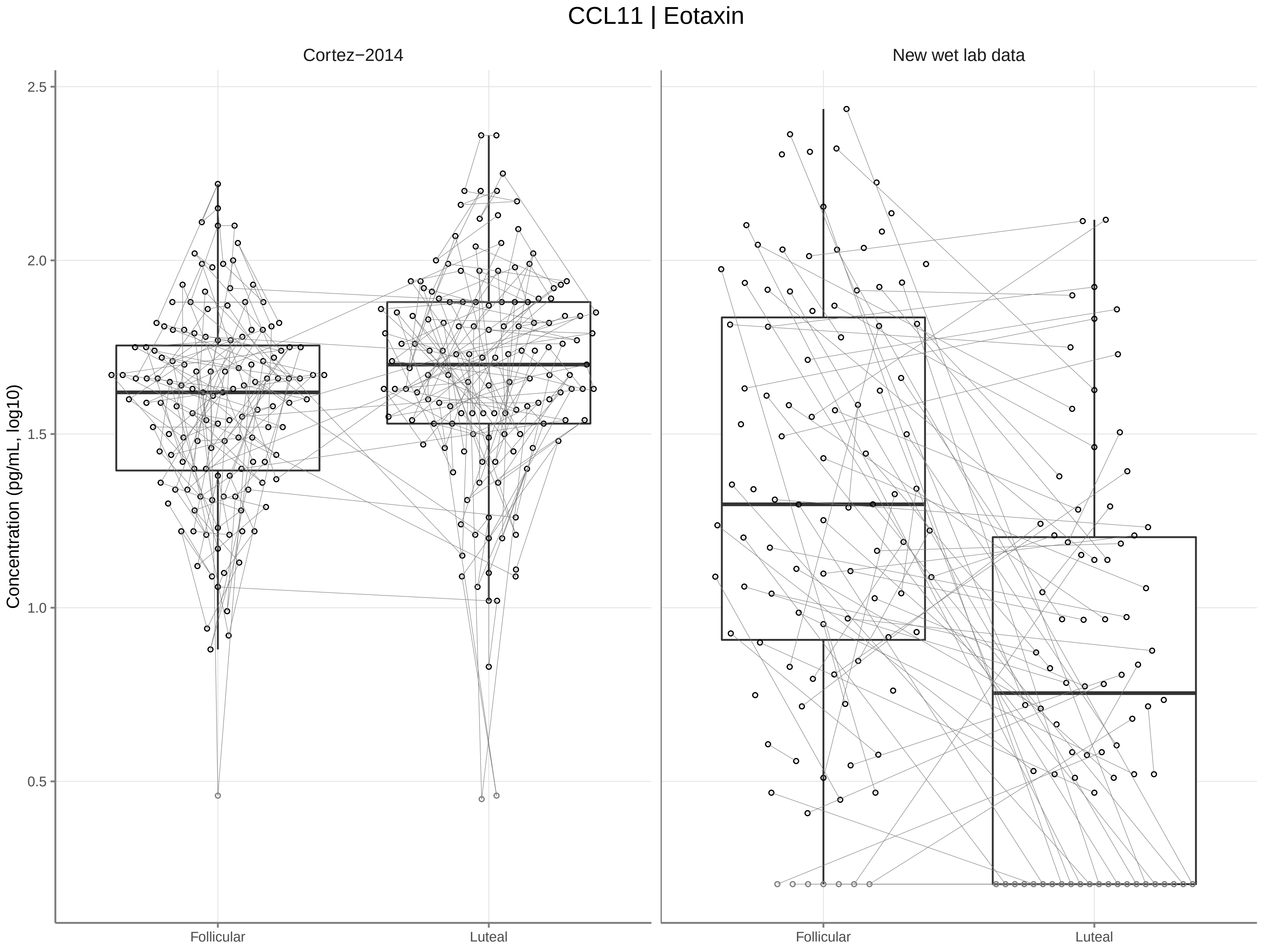
CCL11 | Eotaxin
Cortez−2014
New wet lab data
Concentration (pg/mL, log10)
2.5
2.0
1.5
1.0
0.5
Follicular
Luteal
Follicular
Luteal

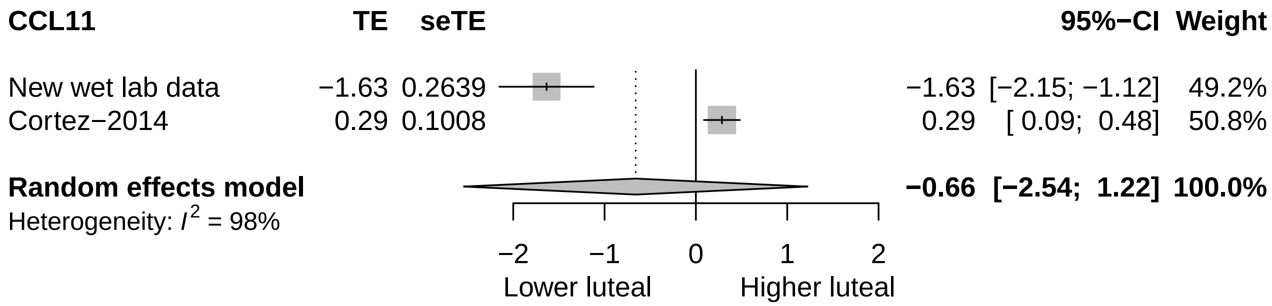
CCL11
TE
seTE
95%−CI
Weight
New wet lab data
−1.63
0.2639
−1.63
[−2.15; −1.12]
49.2%
Cortez−2014
0.29
0.1008
0.29
[0.09; 0.48]
50.8%
Random effects model
−0.66
[−2.54; 1.22]
100.0%
Heterogeneity: I2 = 98%
−2
−1
0
1
2
Lower luteal
Higher luteal

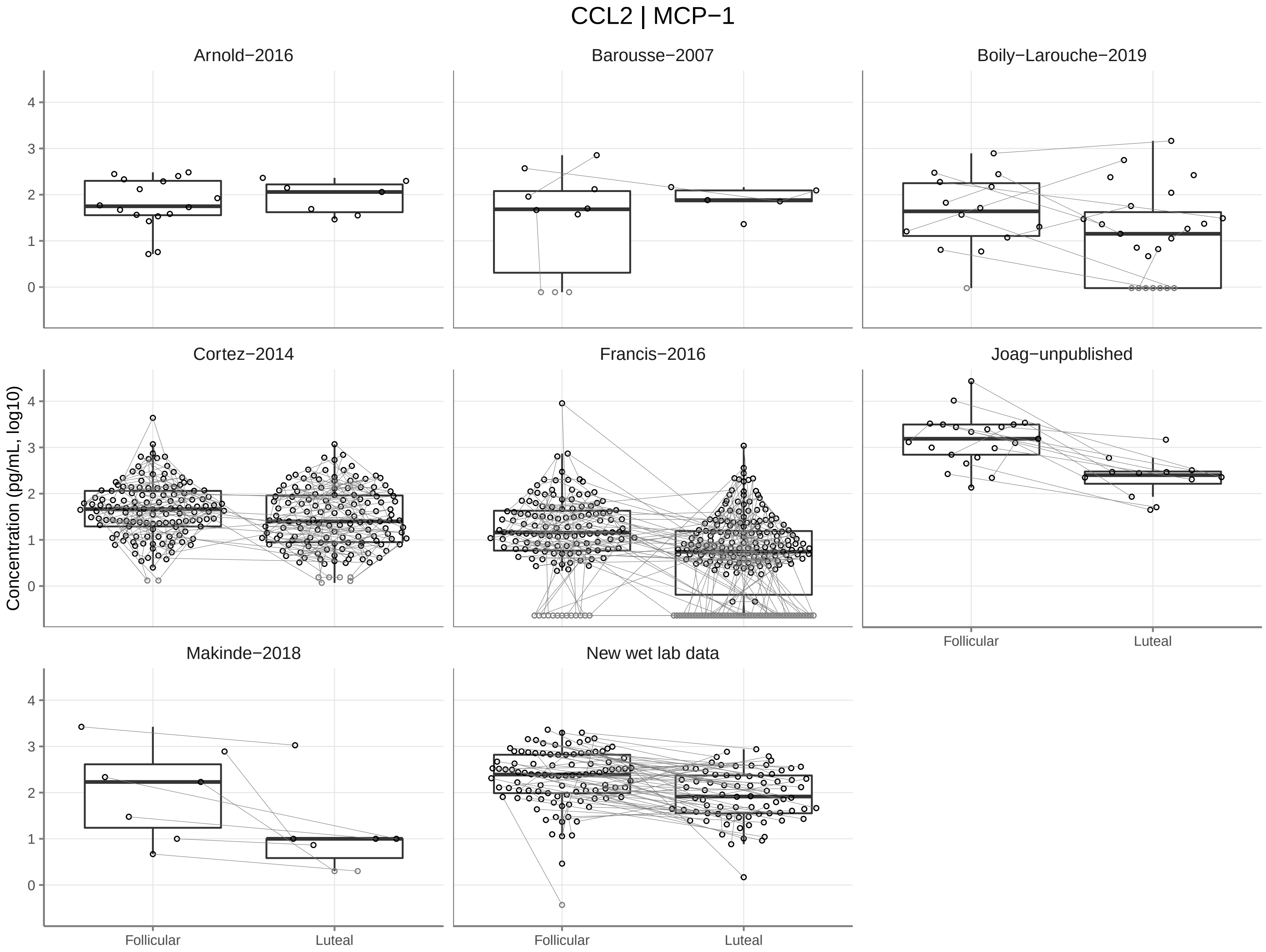
CCL2 | MCP−1
Arnold−2016
Barousse−2007
Boily−Larouche−2019
Cortez−2014
Francis−2016
Joag−unpublished
Makinde−2018
New wet lab data
Concentration (pg/mL, log10)
Follicular
Luteal
0
1
2
3
4

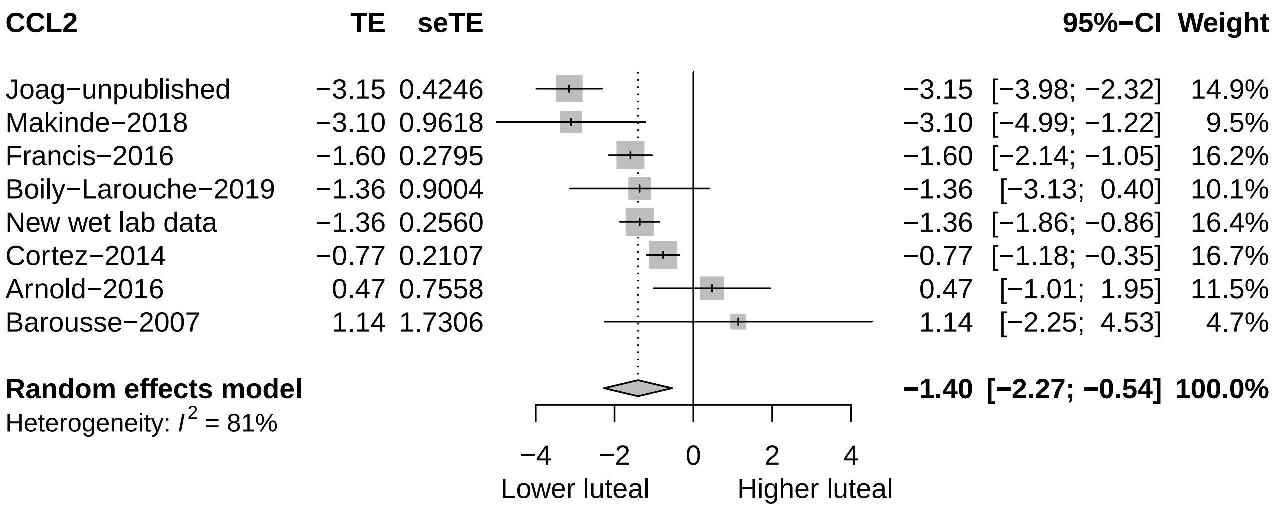
CCL2
TE seTE 95%−CI Weight
Joag−unpublished −3.15 0.4246 −3.15 [−3.98; −2.32] 14.9%
Makinde−2018 −3.10 0.9618 −3.10 [−4.99; −1.22] 9.5%
Francis−2016 −1.60 0.2795 −1.60 [−2.14; −1.05] 16.2%
Boily−Larouche−2019 −1.36 0.9004 −1.36 [−3.13; 0.40] 10.1%
New wet lab data −1.36 0.2560 −1.36 [−1.86; −0.86] 16.4%
Cortez−2014 −0.77 0.2107 −0.77 [−1.18; −0.35] 16.7%
Arnold−2016 0.47 0.7558 0.47 [−1.01; 1.95] 11.5%
Barousse−2007 1.14 1.7306 1.14 [−2.25; 4.53] 4.7%
Random effects model −1.40 [−2.27; −0.54] 100.0%
Heterogeneity: I^2 = 81%
−4 −2 0 2 4
Lower luteal
Higher luteal

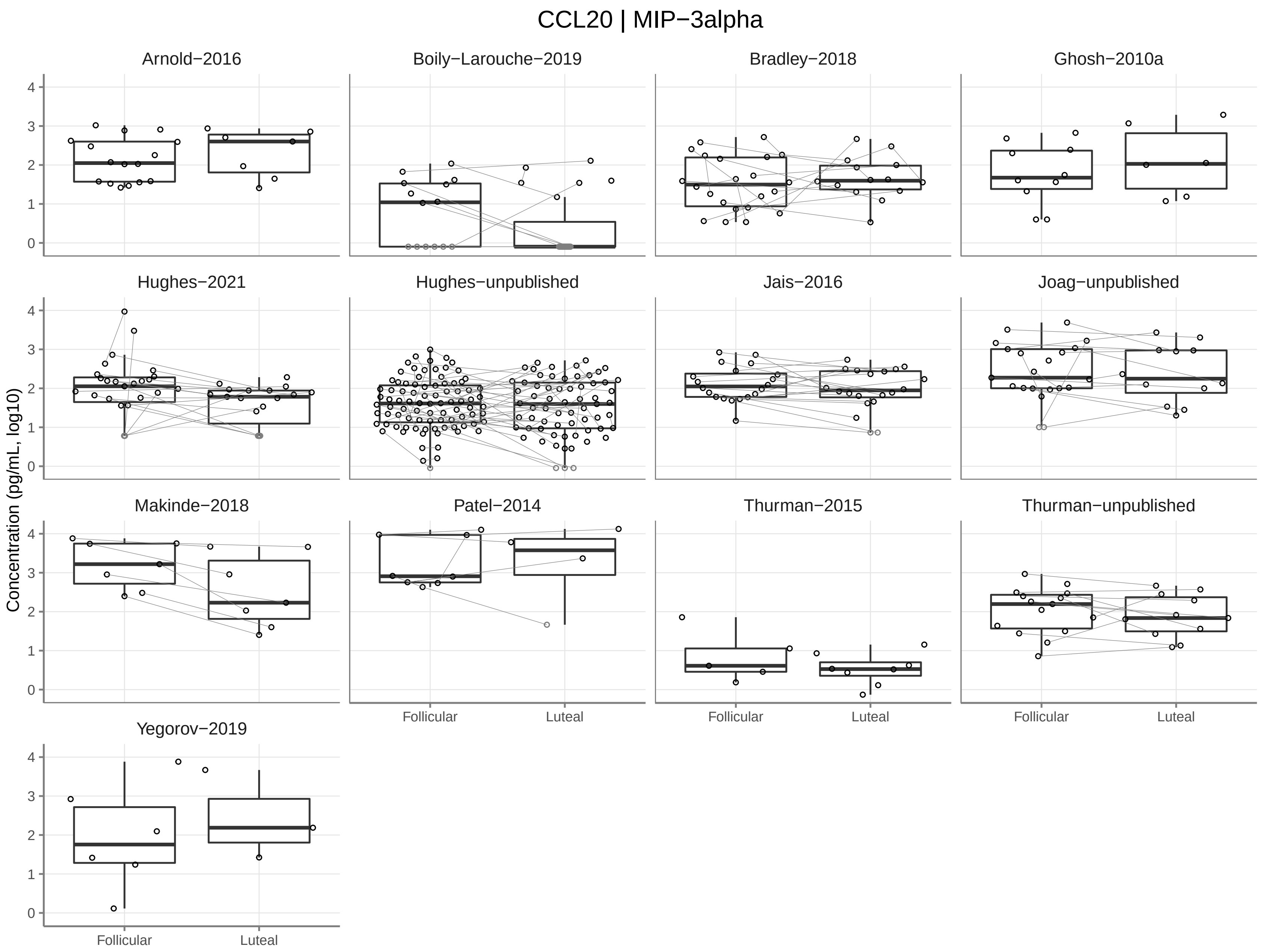

CCL20 | MIP−3alpha
Arnold−2016
Boily−Larouche−2019
Bradley−2018
Ghosh−2010a
Hughes−2021
Hughes−unpublished
Jais−2016
Joag−unpublished
Makinde−2018
Patel−2014
Thurman−2015
Thurman−unpublished
Yegorov−2019
Concentration (pg/mL, log10)
0
1
2
3
4
Follicular
Luteal

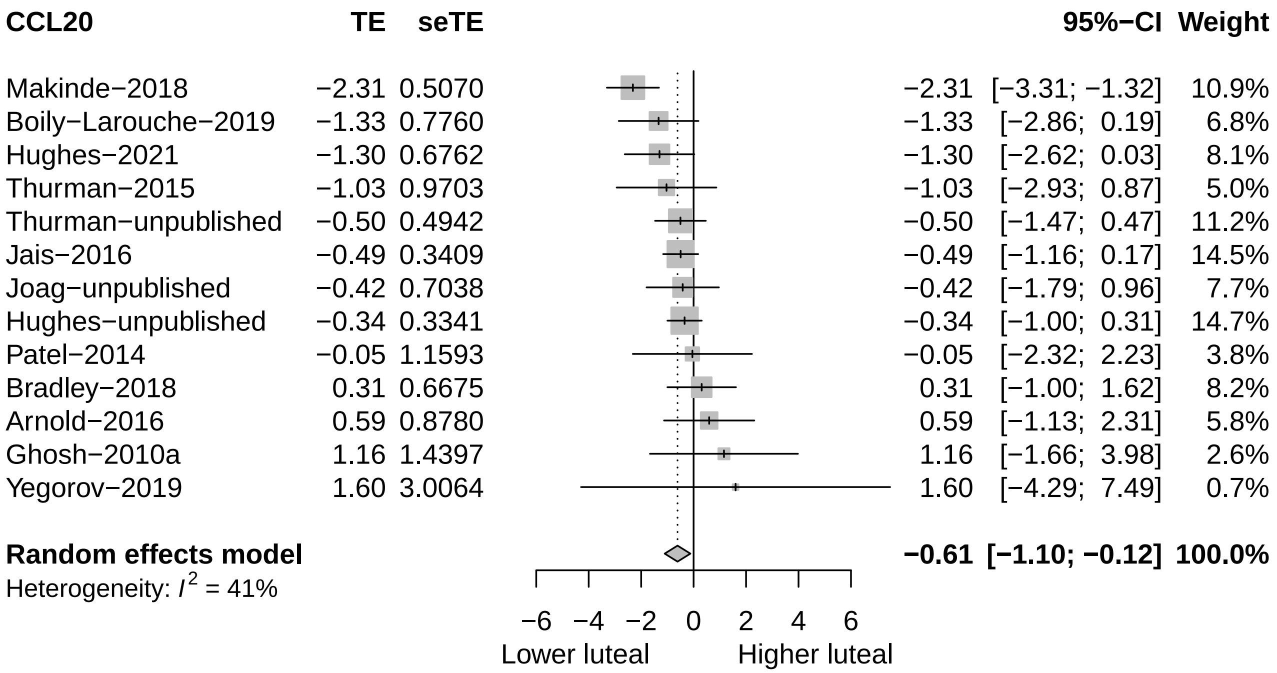
CCL20 TE seTE 95%−CI Weight
Makinde−2018 −2.31 0.5070 −2.31 [−3.31; −1.32] 10.9%
Boily−Larouche−2019 −1.33 0.7760 −1.33 [−2.86; 0.19] 6.8%
Hughes−2021 −1.30 0.6762 −1.30 [−2.62; 0.03] 8.1%
Thurman−2015 −1.03 0.9703 −1.03 [−2.93; 0.87] 5.0%
Thurman−unpublished −0.50 0.4942 −0.50 [−1.47; 0.47] 11.2%
Jais−2016 −0.49 0.3409 −0.49 [−1.16; 0.17] 14.5%
Joag−unpublished −0.42 0.7038 −0.42 [−1.79; 0.96] 7.7%
Hughes−unpublished −0.34 0.3341 −0.34 [−1.00; 0.31] 14.7%
Patel−2014 −0.05 1.1593 −0.05 [−2.32; 2.23] 3.8%
Bradley−2018 0.31 0.6675 0.31 [−1.00; 1.62] 8.2%
Arnold−2016 0.59 0.8780 0.59 [−1.13; 2.31] 5.8%
Ghosh−2010a 1.16 1.4397 1.16 [−1.66; 3.98] 2.6%
Yegorov−2019 1.60 3.0064 1.60 [−4.29; 7.49] 0.7%
Random effects model −0.61 [−1.10; −0.12] 100.0%
Heterogeneity: I^2 = 41%
−6 −4 −2 0 2 4 6
Lower luteal
Higher luteal

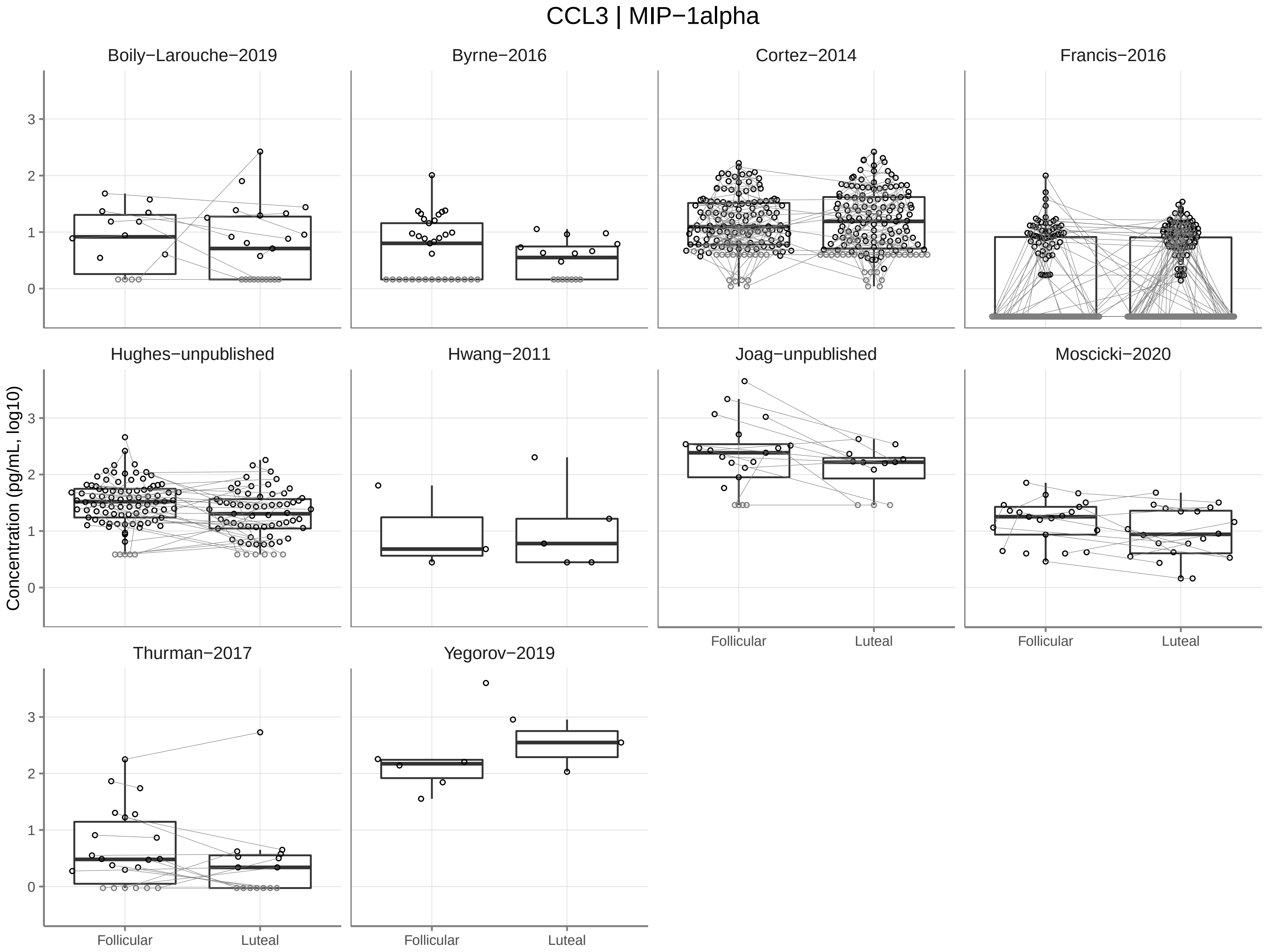

CCL3 | MIP−1alpha
Boily−Larouche−2019
Byrne−2016
Cortez−2014
Francis−2016
Hughes−unpublished
Hwang−2011
Joag−unpublished
Moscicki−2020
Thurman−2017
Yegorov−2019
Concentration (pg/mL, log10)
0
1
2
3
Follicular
Luteal

CCL3	TE	seTE		95%−CI	Weight
Joag−unpublished	−1.11	0.5778	−1.11	[−2.24; 0.02]	5.5%
Hughes−unpublished	−0.63	0.1985	−0.63	[−1.02; −0.24]	18.0%
Moscicki−2020	−0.62	0.2914	−0.62	[−1.19; −0.05]	13.4%
Byrne−2016	−0.61	0.4903	−0.61	[−1.57; 0.35]	7.1%
Thurman−2017	−0.36	0.3543	−0.36	[−1.06; 0.33]	10.9%
Boily−Larouche−2019	−0.30	0.6900	−0.30	[−1.65; 1.05]	4.1%
Francis−2016	−0.21	0.2032	−0.21	[−0.61; 0.19]	17.8%
Cortez−2014	0.19	0.1339	0.19	[−0.07; 0.45]	21.6%
Hwang−2011	0.20	1.8429	0.20	[−3.41; 3.82]	0.7%
Yegorov−2019	0.81	1.5165	0.81	[−2.16; 3.78]	1.0%
**Random effects model**			**−0.34**	**[−0.64; −0.04]**	**100.0%**

Heterogeneity: $I^2 = 53\%$

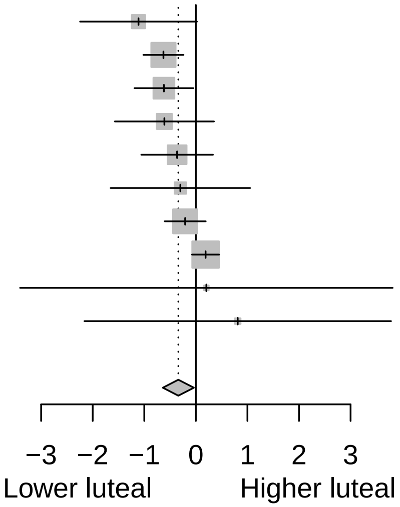

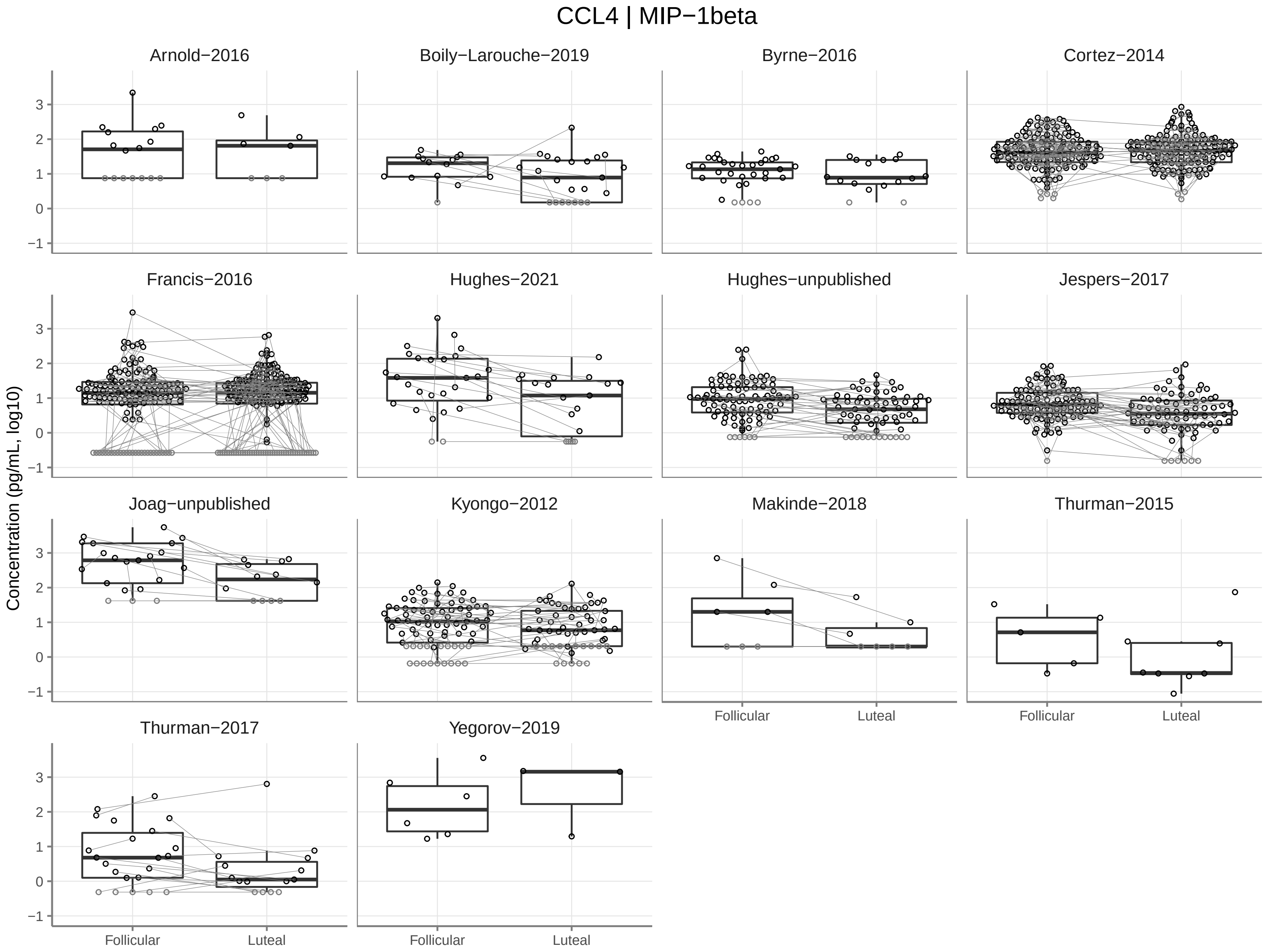
CCL4 | MIP−1beta
Arnold−2016
Boily−Larouche−2019
Byrne−2016
Cortez−2014
Francis−2016
Hughes−2021
Hughes−unpublished
Jespers−2017
Joag−unpublished
Kyongo−2012
Makinde−2018
Thurman−2015
Thurman−2017
Yegorov−2019
Concentration (pg/mL, log10)
3
2
1
0
−1
Follicular
Luteal

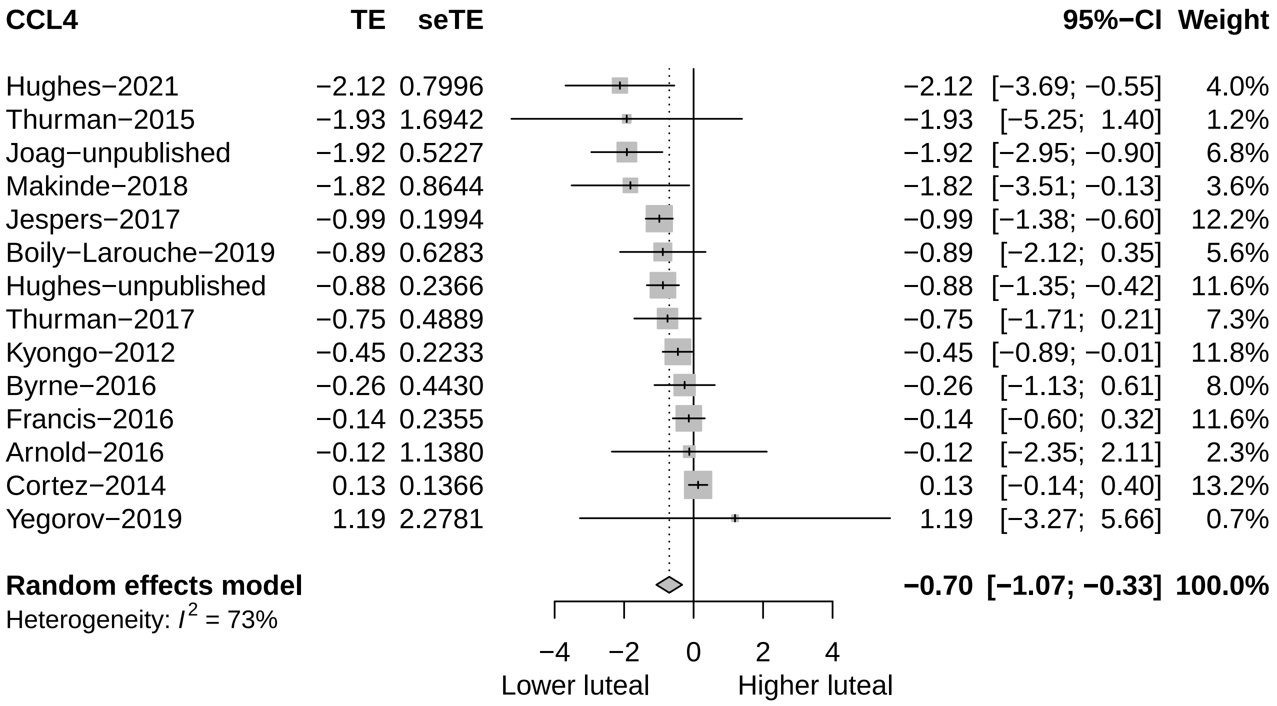
CCL4 TE seTE 95%−CI Weight
Hughes−2021 −2.12 0.7996 −2.12 [−3.69; −0.55] 4.0%
Thurman−2015 −1.93 1.6942 −1.93 [−5.25; 1.40] 1.2%
Joag−unpublished −1.92 0.5227 −1.92 [−2.95; −0.90] 6.8%
Makinde−2018 −1.82 0.8644 −1.82 [−3.51; −0.13] 3.6%
Jespers−2017 −0.99 0.1994 −0.99 [−1.38; −0.60] 12.2%
Boily−Larouche−2019 −0.89 0.6283 −0.89 [−2.12; 0.35] 5.6%
Hughes−unpublished −0.88 0.2366 −0.88 [−1.35; −0.42] 11.6%
Thurman−2017 −0.75 0.4889 −0.75 [−1.71; 0.21] 7.3%
Kyongo−2012 −0.45 0.2233 −0.45 [−0.89; −0.01] 11.8%
Byrne−2016 −0.26 0.4430 −0.26 [−1.13; 0.61] 8.0%
Francis−2016 −0.14 0.2355 −0.14 [−0.60; 0.32] 11.6%
Arnold−2016 −0.12 1.1380 −0.12 [−2.35; 2.11] 2.3%
Cortez−2014 0.13 0.1366 0.13 [−0.14; 0.40] 13.2%
Yegorov−2019 1.19 2.2781 1.19 [−3.27; 5.66] 0.7%
Random effects model −0.70 [−1.07; −0.33] 100.0%
Heterogeneity: I^2 = 73%
−4 −2 0 2 4
Lower luteal
Higher luteal

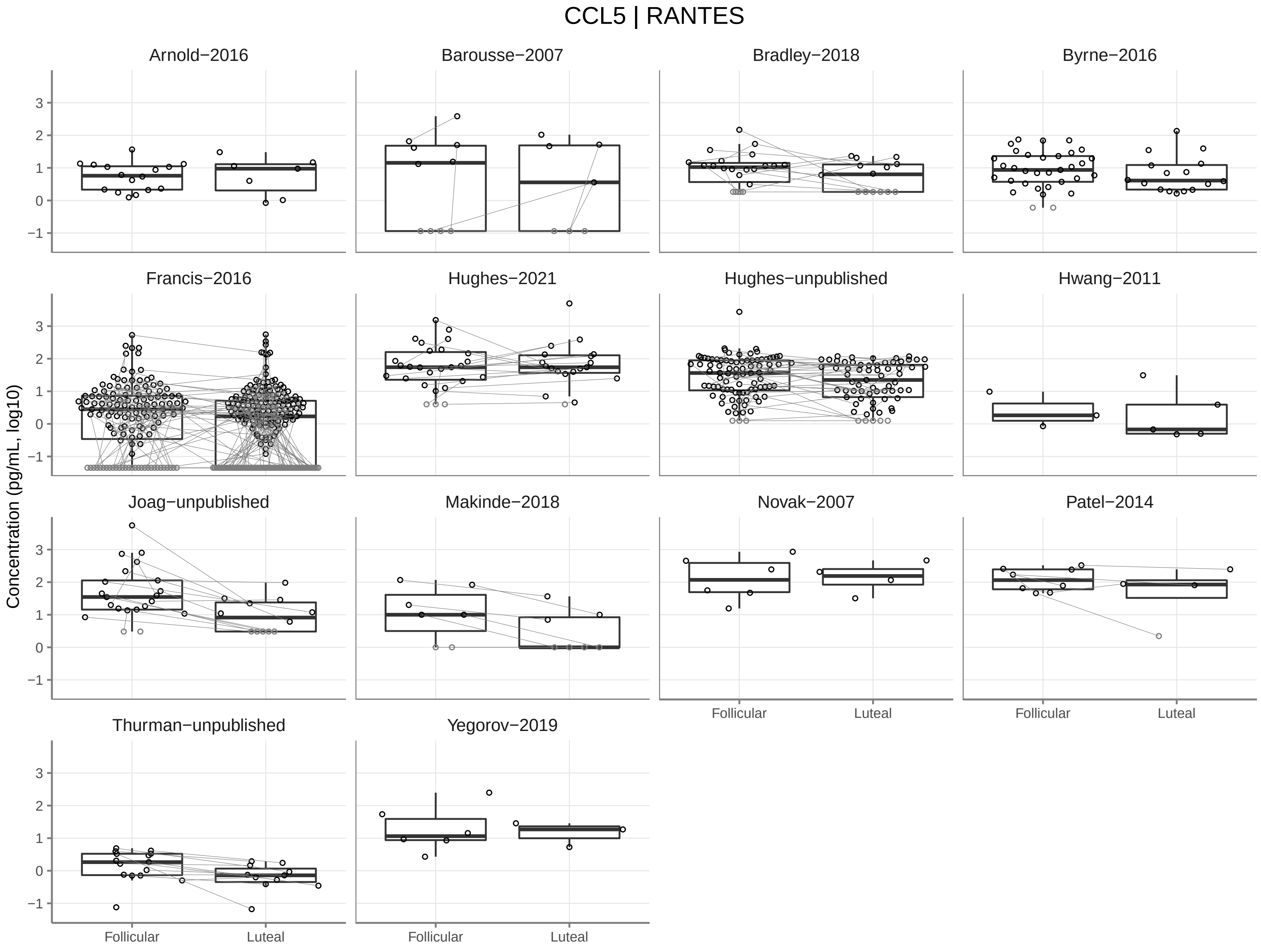
CCL5 | RANTES
Arnold−2016
Barousse−2007
Bradley−2018
Byrne−2016
Francis−2016
Hughes−2021
Hughes−unpublished
Hwang−2011
Joag−unpublished
Makinde−2018
Novak−2007
Patel−2014
Thurman−unpublished
Yegorov−2019
Concentration (pg/mL, log10)
Follicular
Luteal

CCL5	TE	seTE	95%−CI	Weight
Joag−unpublished	−2.60	0.7588	−2.60 [−4.09; −1.12]	4.8%
Makinde−2018	−1.84	0.5517	−1.84 [−2.92; −0.76]	8.0%
Patel−2014	−1.41	1.0384	−1.41 [−3.45; 0.62]	2.8%
Thurman−unpublished	−1.23	0.5442	−1.23 [−2.29; −0.16]	8.2%
Bradley−2018	−0.77	0.5178	−0.77 [−1.79; 0.24]	8.8%
Francis−2016	−0.62	0.3008	−0.62 [−1.21; −0.03]	16.6%
Hughes−unpublished	−0.56	0.2712	−0.56 [−1.10; −0.03]	18.1%
Byrne−2016	−0.45	0.5749	−0.45 [−1.58; 0.67]	7.5%
Hwang−2011	−0.45	1.7300	−0.45 [−3.84; 2.94]	1.1%
Yegorov−2019	−0.40	1.4582	−0.40 [−3.25; 2.46]	1.5%
Shust−2010	0.06	0.6663	0.06 [−1.24; 1.37]	6.0%
Arnold−2016	0.07	0.7271	0.07 [−1.35; 1.50]	5.2%
Novak−2007	0.13	1.2980	0.13 [−2.42; 2.67]	1.9%
Hughes−2021	0.13	0.5174	0.13 [−0.88; 1.14]	8.8%
Barousse−2007	0.18	2.2837	0.18 [−4.29; 4.66]	0.6%
**Random effects model**			**−0.71 [−1.07; −0.35]**	**100.0%**

Heterogeneity: I^2 = 23%

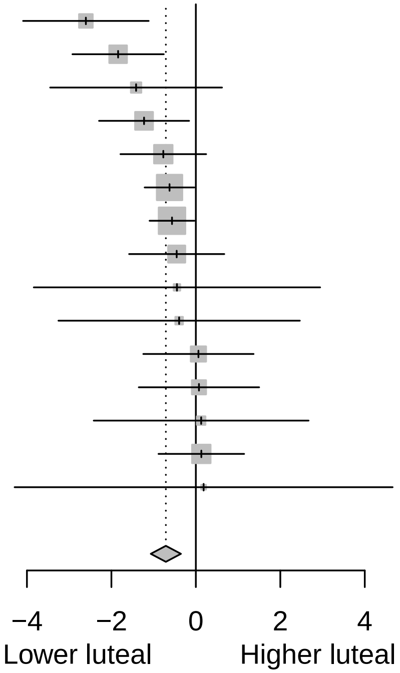

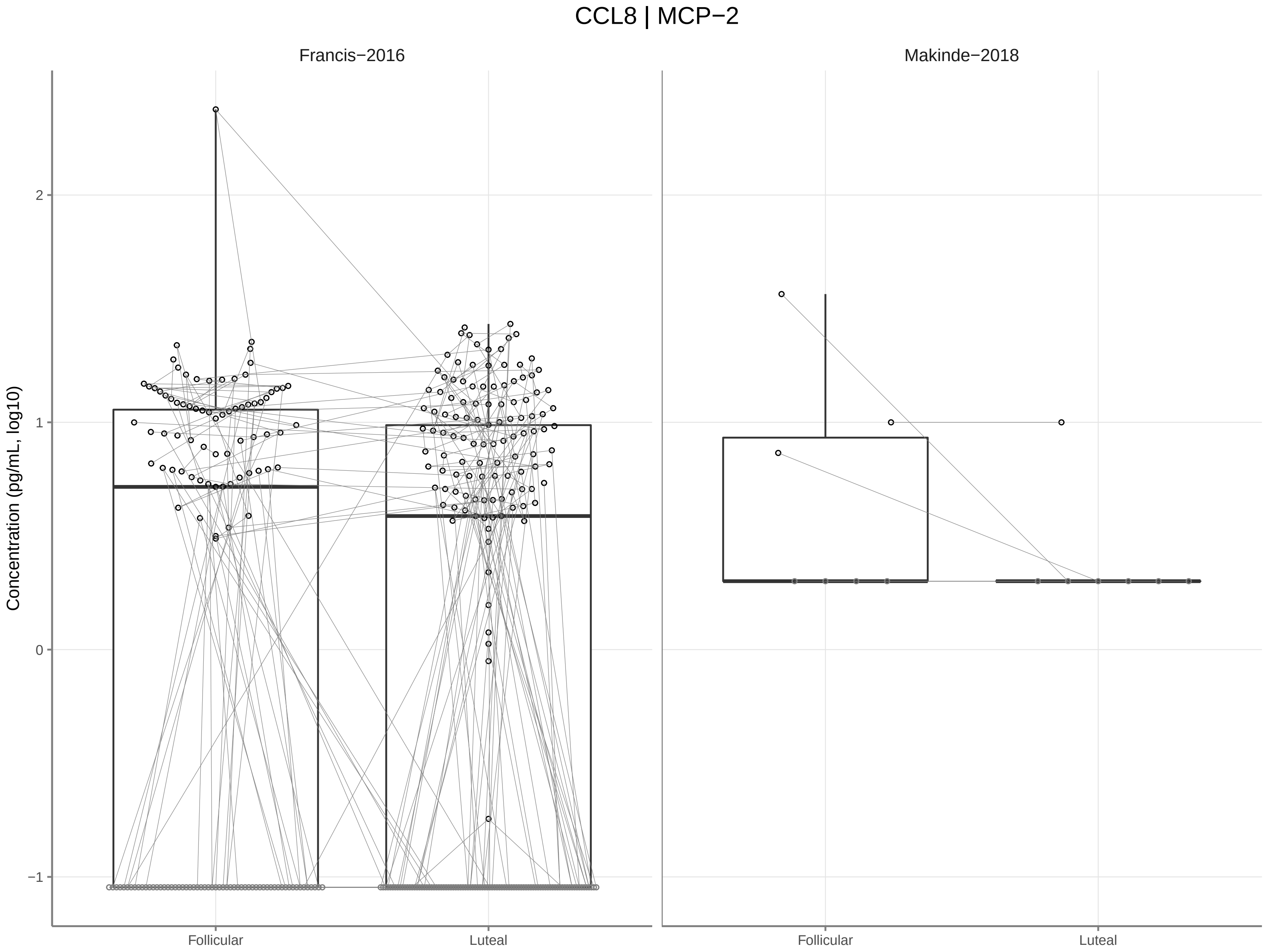
CCL8 | MCP−2
Francis−2016
Makinde−2018
Concentration (pg/mL, log10)
2
1
0
−1
Follicular
Luteal
Follicular
Luteal

CCL8	TE	seTE		95%−CI	Weight
Makinde−2018	−0.87	0.6145		−0.87 [−2.07; 0.34]	17.3%
Francis−2016	−0.38	0.2811		−0.38 [−0.93; 0.17]	82.7%
**Random effects model**				**−0.47 [−0.97; 0.04]**	**100.0%**
Heterogeneity: $I^2 = 0\%$					

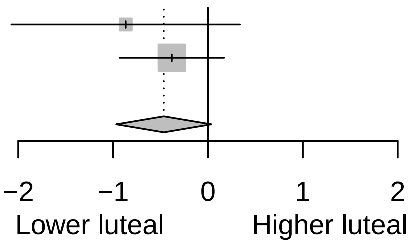

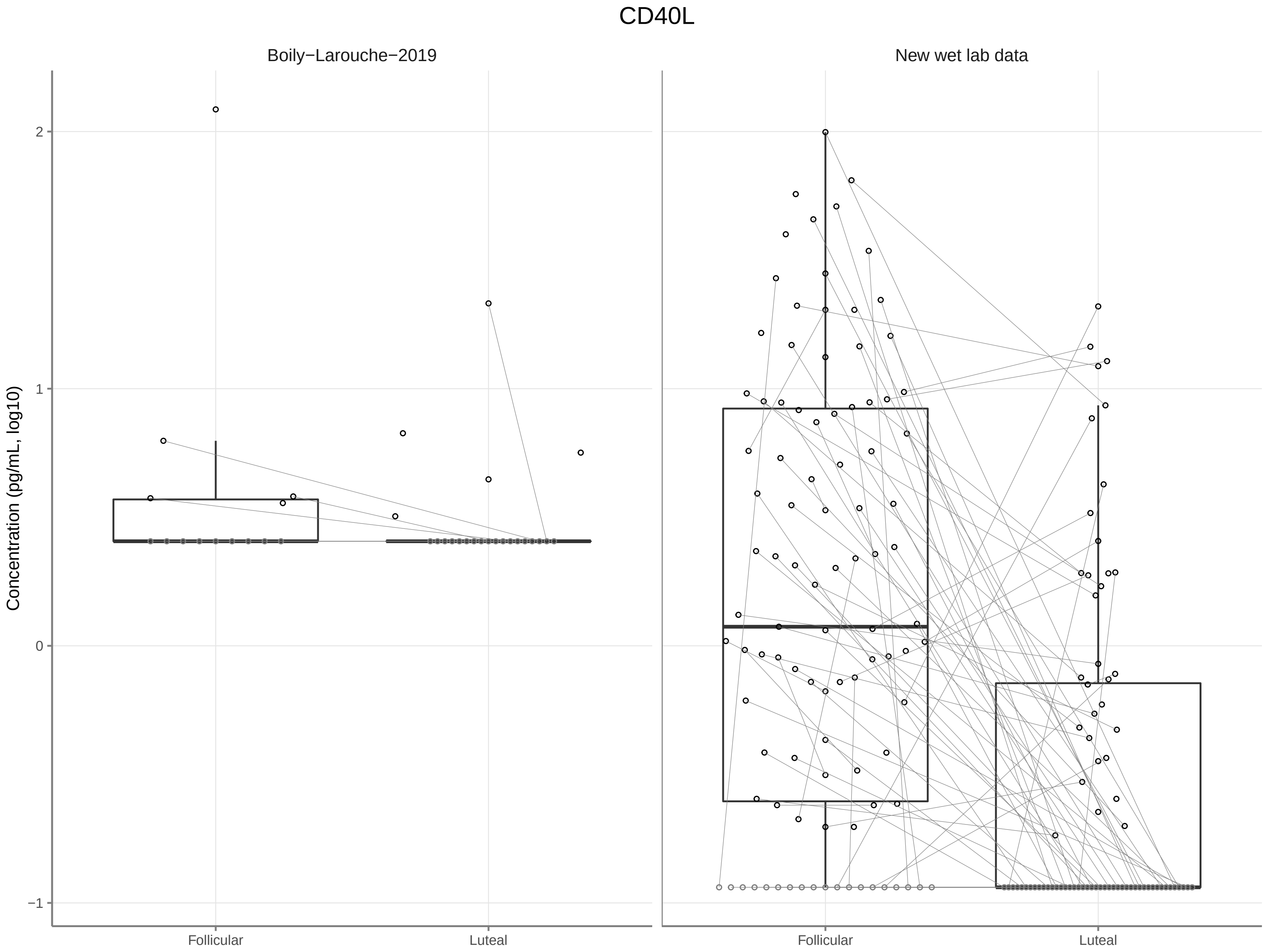
CD40L
Boily−Larouche−2019
New wet lab data
Concentration (pg/mL, log10)
2
1
0
−1
Follicular
Luteal
Follicular
Luteal

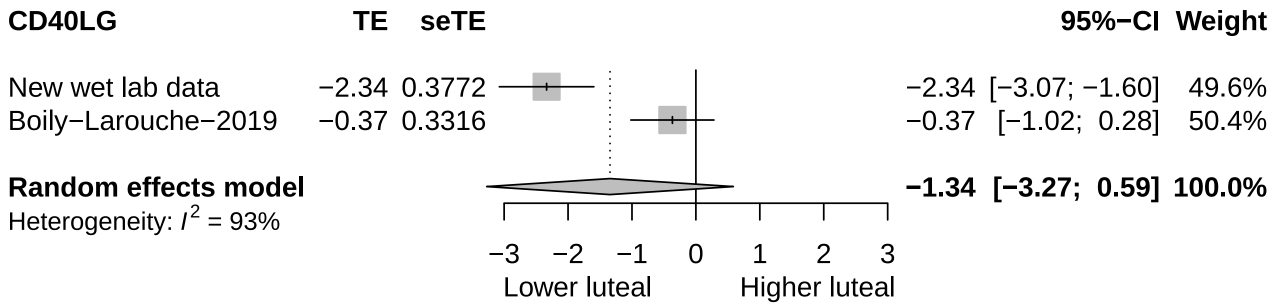
CD40LG
TE seTE
95%−CI Weight
New wet lab data −2.34 0.3772 −2.34 [−3.07; −1.60] 49.6%
Boily−Larouche−2019 −0.37 0.3316 −0.37 [−1.02; 0.28] 50.4%
Random effects model −1.34 [−3.27; 0.59] 100.0%
Heterogeneity: I2 = 93%
−3 −2 −1 0 1 2 3
Lower luteal
Higher luteal

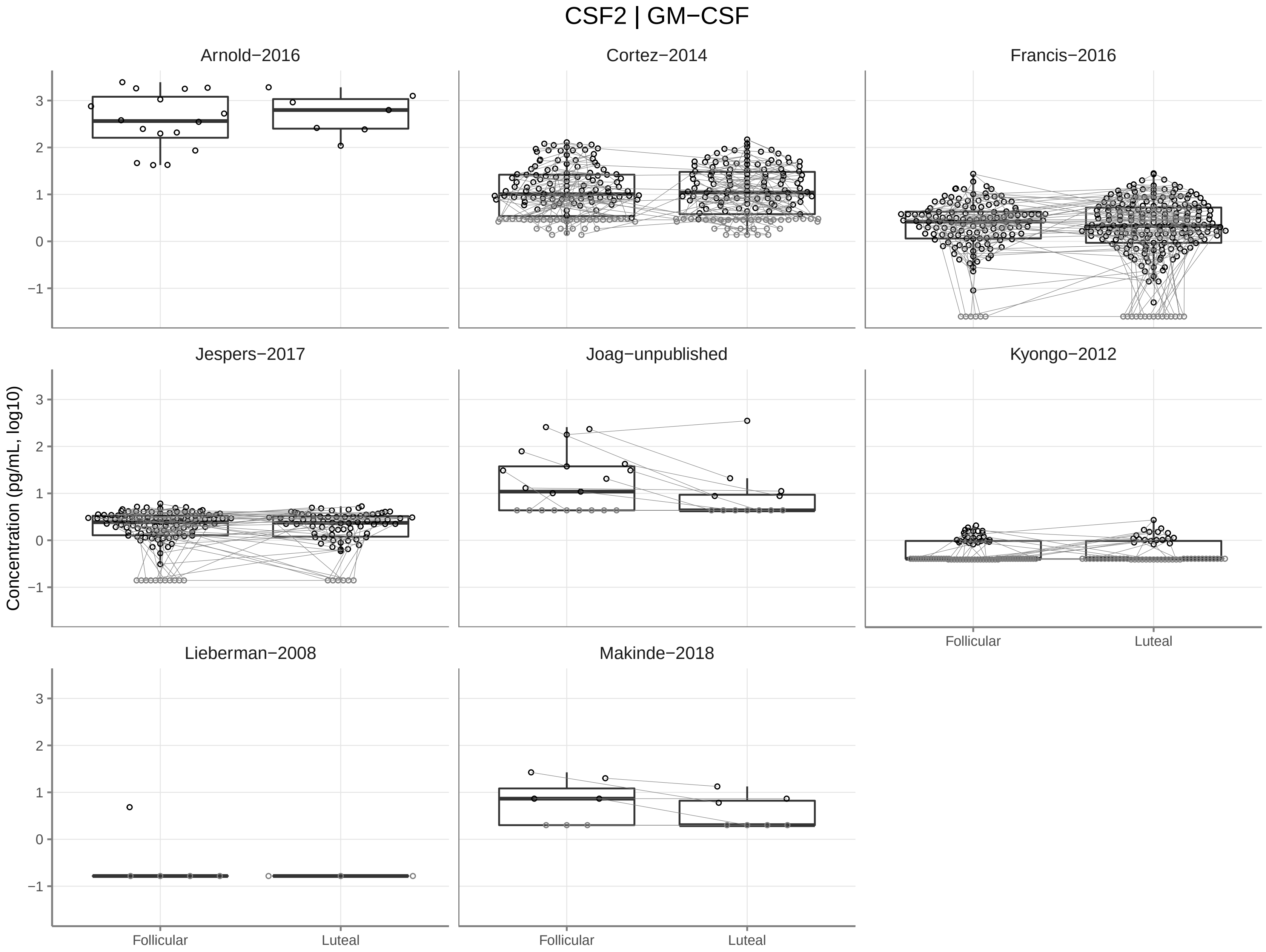
CSF2 | GM−CSF
Arnold−2016
Cortez−2014
Francis−2016
Jespers−2017
Joag−unpublished
Kyongo−2012
Lieberman−2008
Makinde−2018
Concentration (pg/mL, log10)
3
2
1
0
−1
Follicular
Luteal

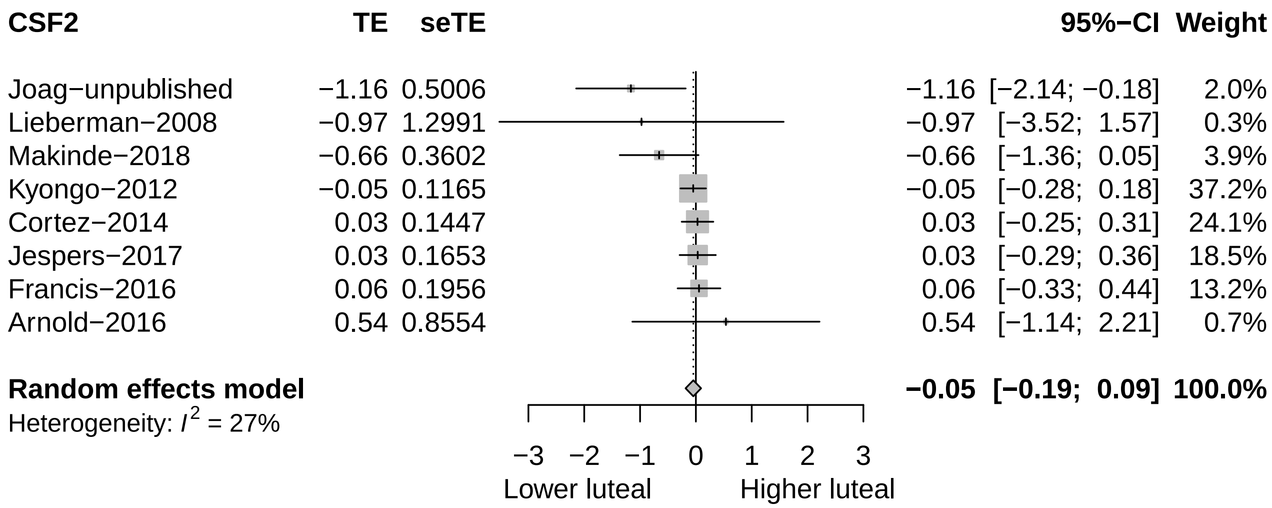
CSF2
TE
seTE
95%−CI
Weight
Joag−unpublished −1.16 0.5006 −1.16 [−2.14; −0.18] 2.0%
Lieberman−2008 −0.97 1.2991 −0.97 [−3.52; 1.57] 0.3%
Makinde−2018 −0.66 0.3602 −0.66 [−1.36; 0.05] 3.9%
Kyongo−2012 −0.05 0.1165 −0.05 [−0.28; 0.18] 37.2%
Cortez−2014 0.03 0.1447 0.03 [−0.25; 0.31] 24.1%
Jespers−2017 0.03 0.1653 0.03 [−0.29; 0.36] 18.5%
Francis−2016 0.06 0.1956 0.06 [−0.33; 0.44] 13.2%
Arnold−2016 0.54 0.8554 0.54 [−1.14; 2.21] 0.7%
Random effects model −0.05 [−0.19; 0.09] 100.0%
Heterogeneity: I2 = 27%
−3 −2 −1 0 1 2 3
Lower luteal
Higher luteal

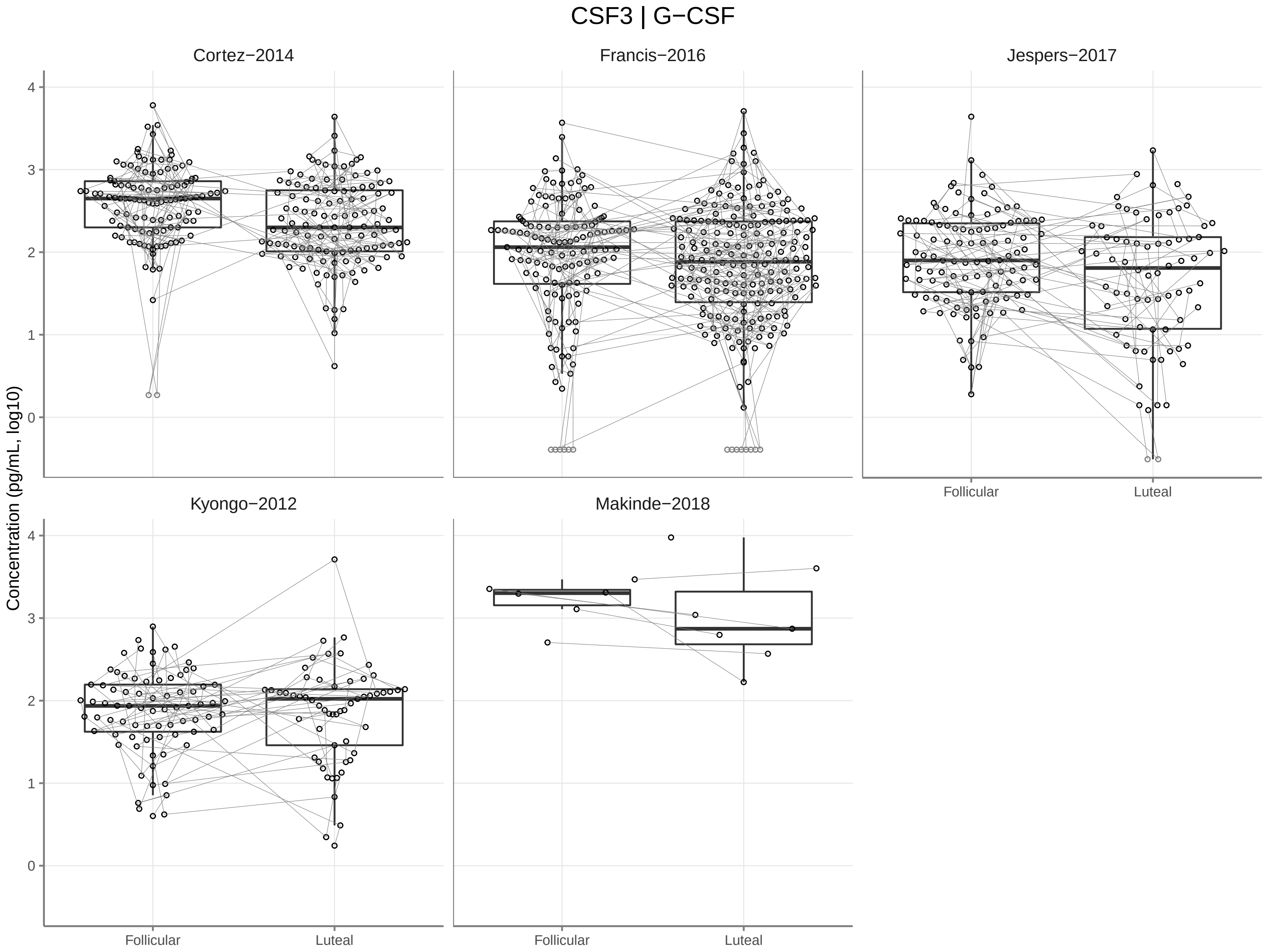

CSF3 | G−CSF
Cortez−2014
Francis−2016
Jespers−2017
Kyongo−2012
Makinde−2018
Concentration (pg/mL, log10)
4
3
2
1
0
Follicular
Luteal

CSF3	TE	seTE		95%−CI	Weight
Makinde−2018	−0.99	0.5865	−0.99	[−2.14; 0.16]	6.9%
Jespers−2017	−0.91	0.2391	−0.91	[−1.38; −0.44]	21.6%
Cortez−2014	−0.74	0.1857	−0.74	[−1.10; −0.37]	26.0%
Francis−2016	−0.27	0.2010	−0.27	[−0.67; 0.12]	24.7%
Kyongo−2012	−0.10	0.2498	−0.10	[−0.59; 0.39]	20.8%
**Random effects model**			**−0.55**	**[−0.88; −0.21]**	**100.0%**

Heterogeneity: I^2 = 55%

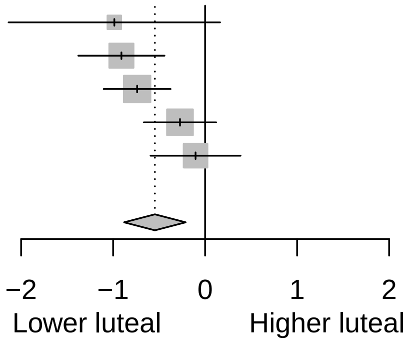

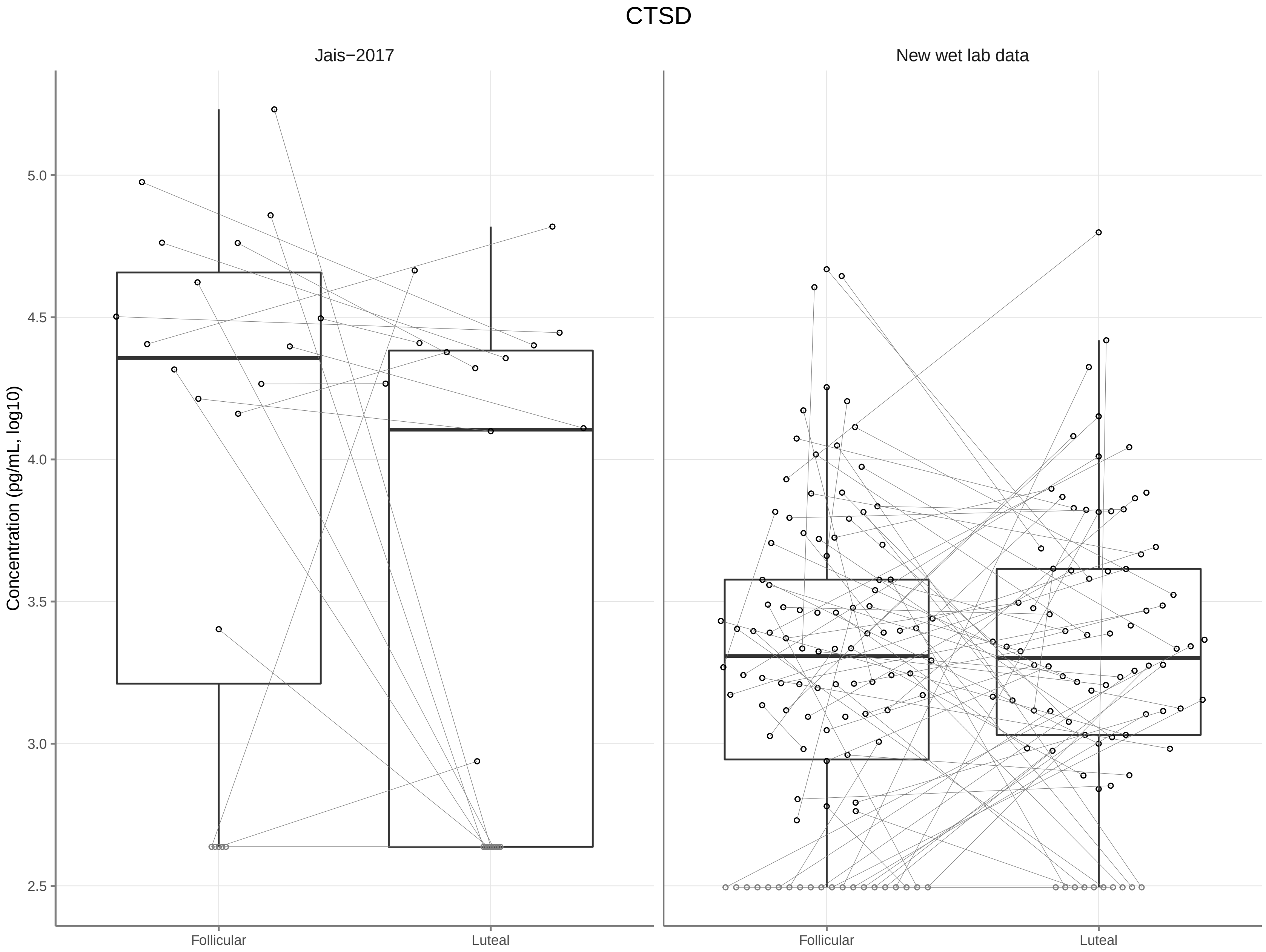

CTSD
Jais−2017
New wet lab data
Concentration (pg/mL, log10)
5.0
4.5
4.0
3.5
3.0
2.5
Follicular
Luteal
Follicular
Luteal

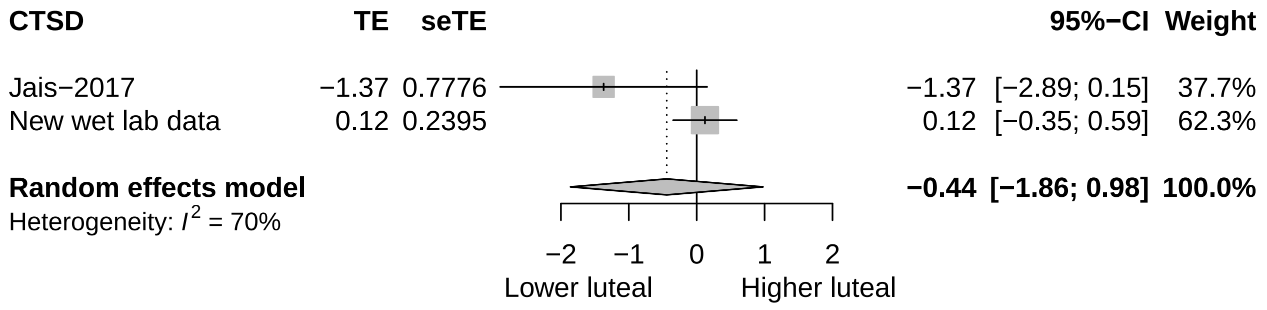
CTSD
TE
seTE
95%−CI
Weight
Jais−2017 −1.37 0.7776 −1.37 [−2.89; 0.15] 37.7%
New wet lab data 0.12 0.2395 0.12 [−0.35; 0.59] 62.3%
Random effects model −0.44 [−1.86; 0.98] 100.0%
Heterogeneity: I^2 = 70%
−2 −1 0 1 2
Lower luteal
Higher luteal

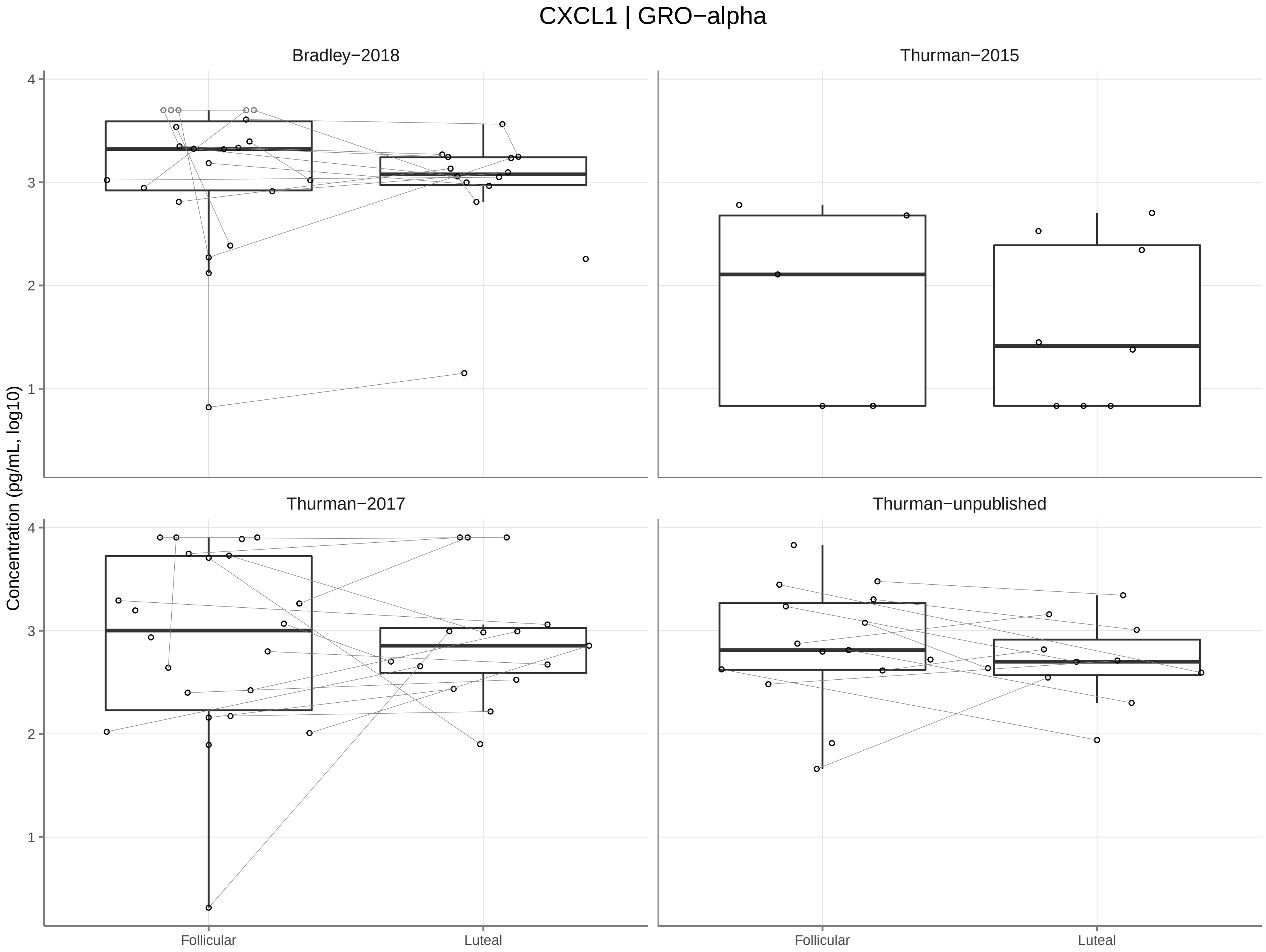

CXCL1 | GRO−alpha
Bradley−2018
Thurman−2015
Thurman−2017
Thurman−unpublished
Concentration (pg/mL, log10)
4
3
2
1
Follicular
Luteal

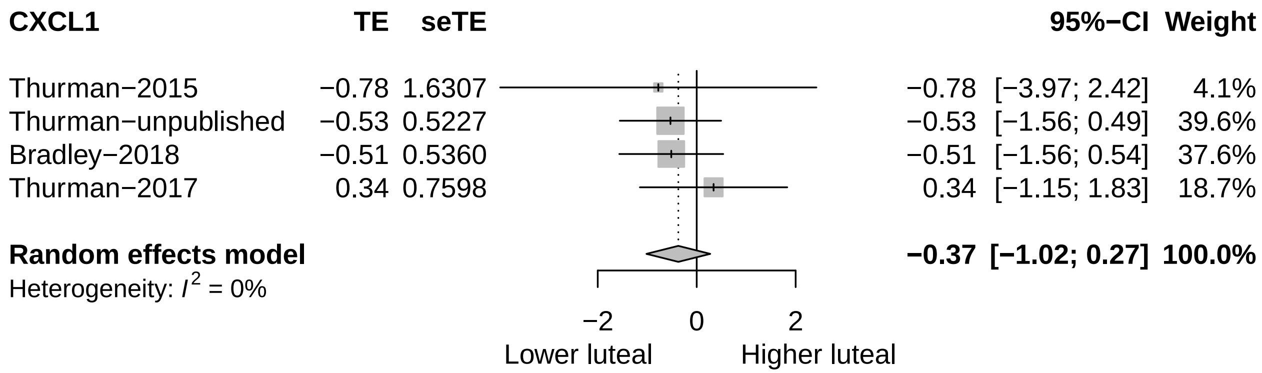
CXCL1
TE
seTE
95%−CI
Weight
Thurman−2015 −0.78 1.6307 −0.78 [−3.97; 2.42] 4.1%
Thurman−unpublished −0.53 0.5227 −0.53 [−1.56; 0.49] 39.6%
Bradley−2018 −0.51 0.5360 −0.51 [−1.56; 0.54] 37.6%
Thurman−2017 0.34 0.7598 0.34 [−1.15; 1.83] 18.7%
Random effects model −0.37 [−1.02; 0.27] 100.0%
Heterogeneity: I^2 = 0%
−2
0
2
Lower luteal
Higher luteal

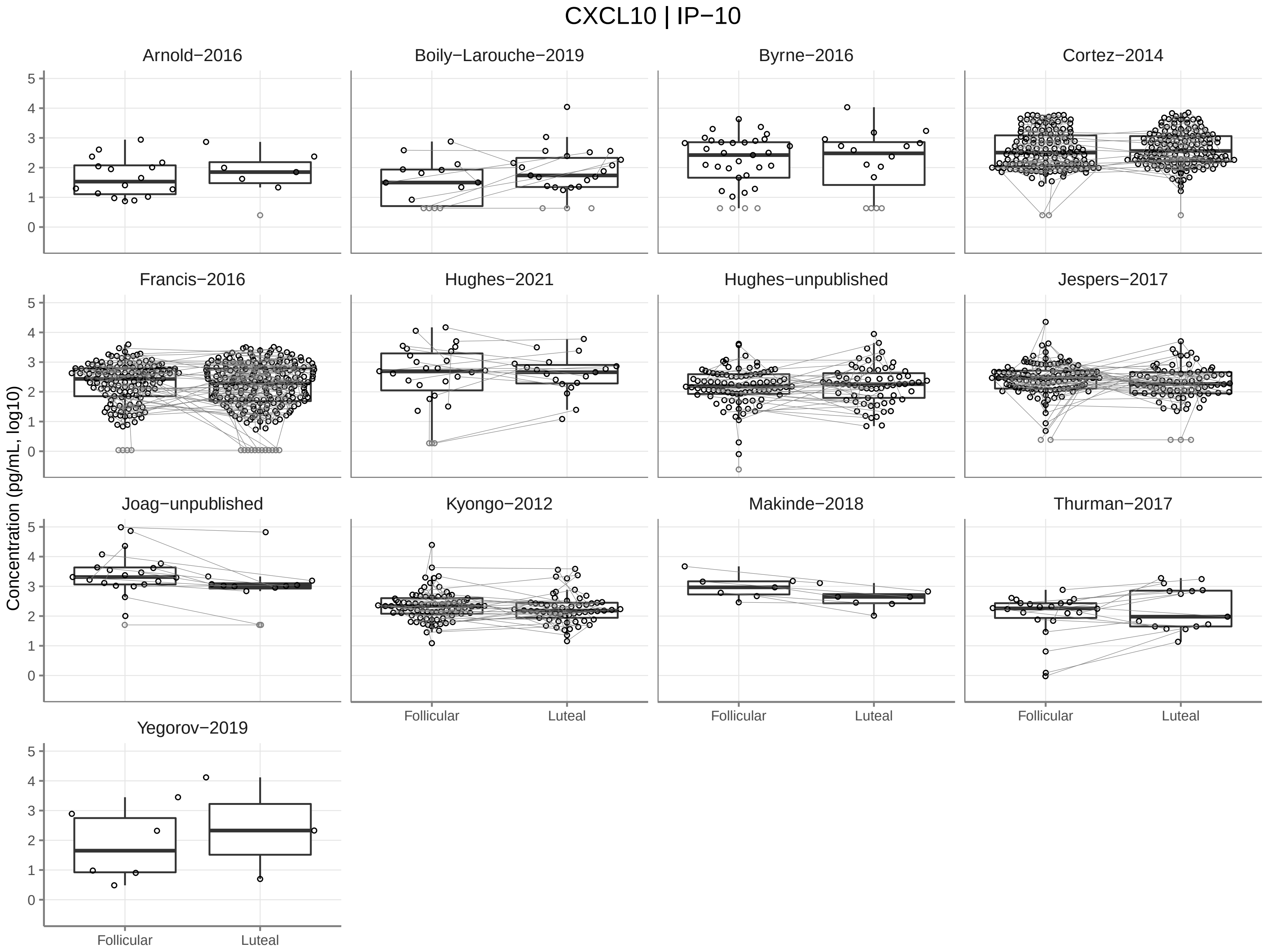

CXCL10 | IP−10
Arnold−2016
Boily−Larouche−2019
Byrne−2016
Cortez−2014
Francis−2016
Hughes−2021
Hughes−unpublished
Jespers−2017
Joag−unpublished
Kyongo−2012
Makinde−2018
Thurman−2017
Yegorov−2019
Concentration (pg/mL, log10)
0
1
2
3
4
5
Follicular
Luteal

CXCL10	TE	seTE	95%−CI	Weight
Joag−unpublished	−1.67	0.5374	−1.67 [−2.72; −0.61]	7.5%
Makinde−2018	−1.32	0.3699	−1.32 [−2.05; −0.60]	9.6%
Jespers−2017	−0.52	0.2645	−0.52 [−1.03; 0.00]	11.0%
Kyongo−2012	−0.37	0.2036	−0.37 [−0.77; 0.03]	11.7%
Francis−2016	−0.08	0.2137	−0.08 [−0.50; 0.33]	11.6%
Byrne−2016	−0.02	0.9566	−0.02 [−1.89; 1.86]	3.9%
Cortez−2014	0.03	0.1726	0.03 [−0.31; 0.37]	12.0%
Hughes−2021	0.17	0.6612	0.17 [−1.13; 1.46]	6.1%
Hughes−unpublished	0.17	0.3234	0.17 [−0.46; 0.80]	10.2%
Arnold−2016	0.38	1.0432	0.38 [−1.67; 2.42]	3.5%
Thurman−2017	1.18	0.5334	1.18 [0.13; 2.23]	7.5%
Boily−Larouche−2019	1.45	0.7981	1.45 [−0.11; 3.01]	5.0%
Yegorov−2019	1.80	3.2284	1.80 [−4.52; 8.13]	0.5%
**Random effects model**			**−0.15 [−0.59; 0.30]**	**100.0%**

Heterogeneity: I^2 = 65%

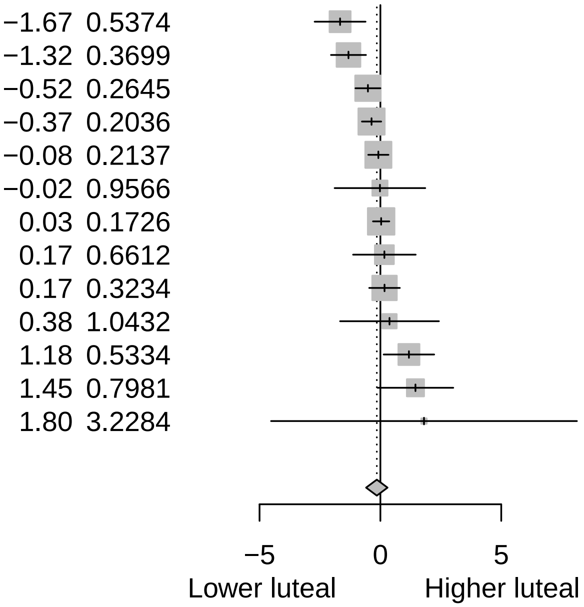

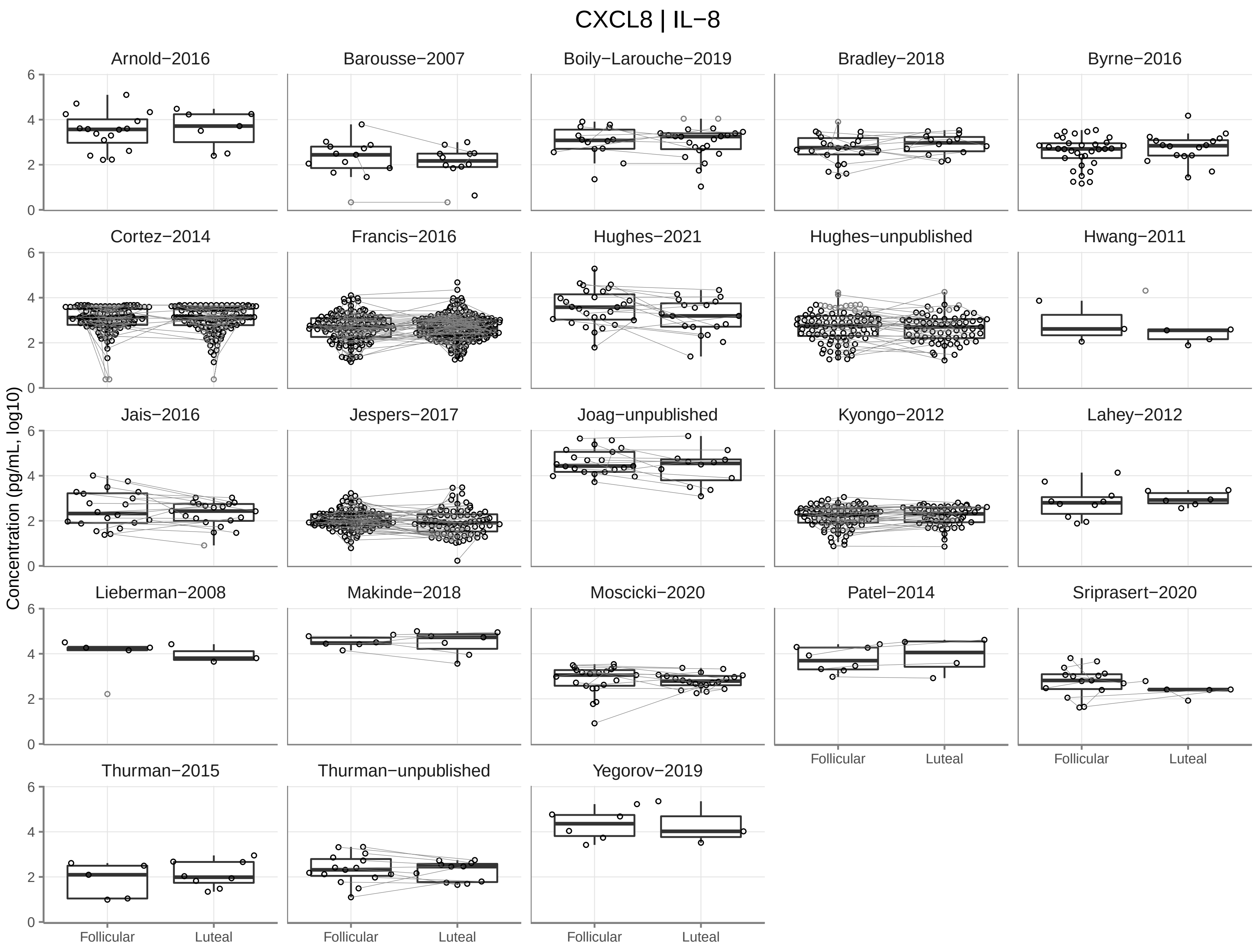

CXCL8 | IL−8
Arnold−2016
Barousse−2007
Boily−Larouche−2019
Bradley−2018
Byrne−2016
Cortez−2014
Francis−2016
Hughes−2021
Hughes−unpublished
Hwang−2011
Jais−2016
Jespers−2017
Joag−unpublished
Kyongo−2012
Lahey−2012
Lieberman−2008
Makinde−2018
Moscicki−2020
Patel−2014
Sriprasert−2020
Thurman−2015
Thurman−unpublished
Yegorov−2019
Concentration (pg/mL, log10)
0
2
4
6
Follicular
Luteal

CXCL8	TE	seTE		95%−CI	Weight
Hughes−2021	−1.60	0.6272	−1.60	[−2.83; −0.37]	2.1%
Barousse−2007	−1.30	0.9933	−1.30	[−3.24; 0.65]	0.9%
Joag−unpublished	−1.01	0.5010	−1.01	[−1.99; −0.02]	3.1%
Jais−2016	−0.69	0.4399	−0.69	[−1.55; 0.17]	3.9%
Hwang−2011	−0.47	2.2811	−0.47	[−4.94; 4.00]	0.2%
Jespers−2017	−0.38	0.1759	−0.38	[−0.73; −0.04]	13.7%
Thurman−unpublished	−0.34	0.4798	−0.34	[−1.28; 0.60]	3.4%
Makinde−2018	−0.28	0.5118	−0.28	[−1.28; 0.73]	3.0%
Hughes−unpublished	−0.26	0.3088	−0.26	[−0.87; 0.34]	6.9%
Shust−2010	−0.20	0.5870	−0.20	[−1.35; 0.95]	2.4%
Cortez−2014	−0.15	0.2036	−0.15	[−0.55; 0.25]	11.8%
Boily−Larouche−2019	−0.11	0.5989	−0.11	[−1.28; 1.06]	2.3%
Moscicki−2020	−0.05	0.4962	−0.05	[−1.02; 0.92]	3.2%
Yegorov−2019	−0.05	1.8178	−0.05	[−3.61; 3.51]	0.3%
Kyongo−2012	0.06	0.1535	0.06	[−0.24; 0.37]	15.5%
Francis−2016	0.19	0.1605	0.19	[−0.13; 0.50]	14.9%
Sriprasert−2020	0.20	0.8802	0.20	[−1.53; 1.92]	1.1%
Lieberman−2008	0.26	1.9510	0.26	[−3.57; 4.08]	0.2%
Arnold−2016	0.29	1.2824	0.29	[−2.22; 2.81]	0.5%
Bradley−2018	0.41	0.5055	0.41	[−0.58; 1.40]	3.1%
Lahey−2012	0.51	1.0502	0.51	[−1.55; 2.57]	0.8%
Patel−2014	0.57	0.4246	0.57	[−0.27; 1.40]	4.2%
Byrne−2016	0.60	0.6747	0.60	[−0.72; 1.92]	1.8%
Thurman−2015	0.88	1.2626	0.88	[−1.59; 3.36]	0.6%
**Random effects model**			**−0.12**	**[−0.31; 0.06]**	**100.0%**

Heterogeneity: I^2 = 10%

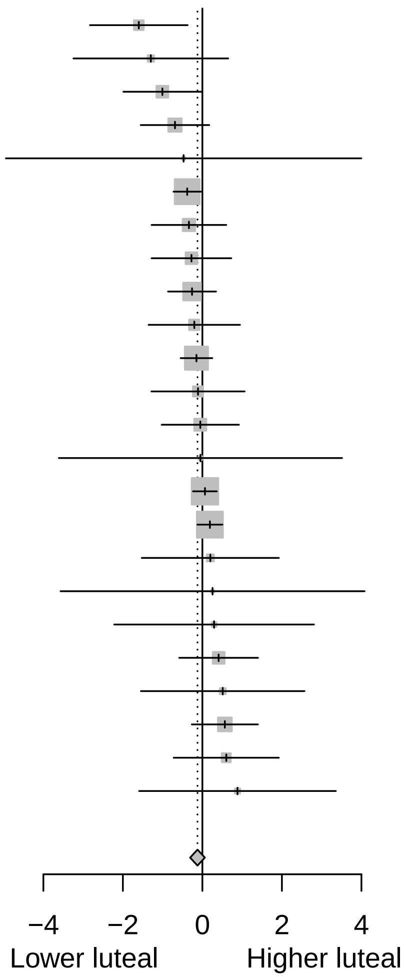

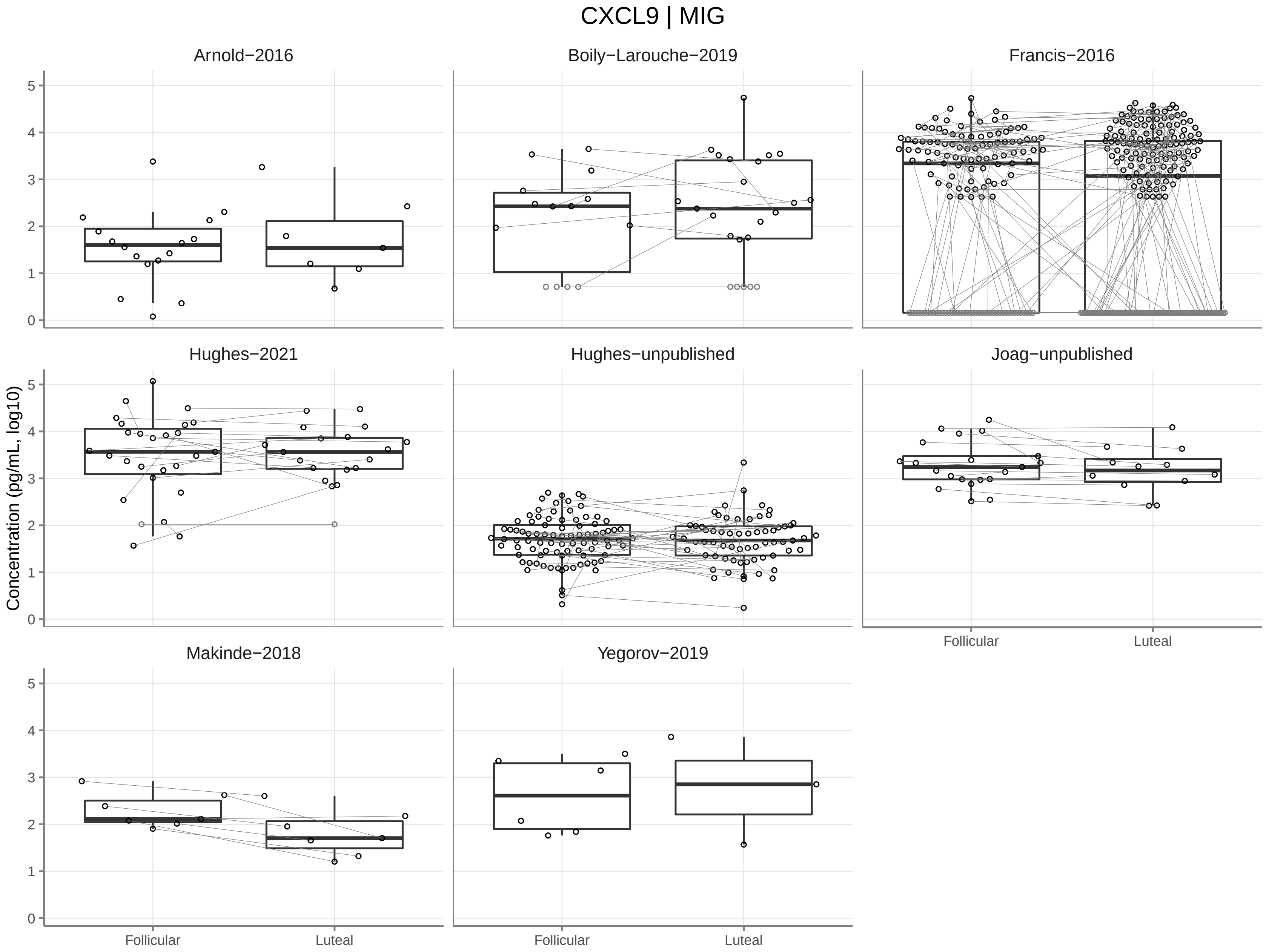
CXCL9 | MIG
Arnold−2016
Boily−Larouche−2019
Francis−2016
Hughes−2021
Hughes−unpublished
Joag−unpublished
Makinde−2018
Yegorov−2019
Concentration (pg/mL, log10)
0
1
2
3
4
5
Follicular
Luteal

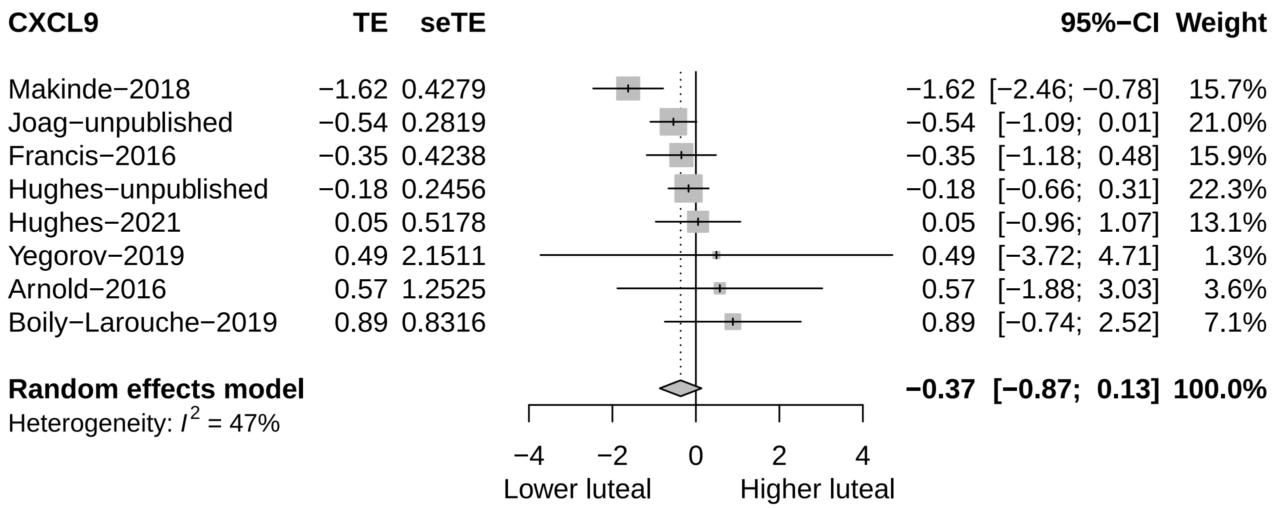
CXCL9
TE
seTE
95%−CI
Weight
Makinde−2018 −1.62 0.4279 −1.62 [−2.46; −0.78] 15.7%
Joag−unpublished −0.54 0.2819 −0.54 [−1.09; 0.01] 21.0%
Francis−2016 −0.35 0.4238 −0.35 [−1.18; 0.48] 15.9%
Hughes−unpublished −0.18 0.2456 −0.18 [−0.66; 0.31] 22.3%
Hughes−2021 0.05 0.5178 0.05 [−0.96; 1.07] 13.1%
Yegorov−2019 0.49 2.1511 0.49 [−3.72; 4.71] 1.3%
Arnold−2016 0.57 1.2525 0.57 [−1.88; 3.03] 3.6%
Boily−Larouche−2019 0.89 0.8316 0.89 [−0.74; 2.52] 7.1%
Random effects model −0.37 [−0.87; 0.13] 100.0%
Heterogeneity: I2 = 47%
−4
−2
0
2
4
Lower luteal
Higher luteal

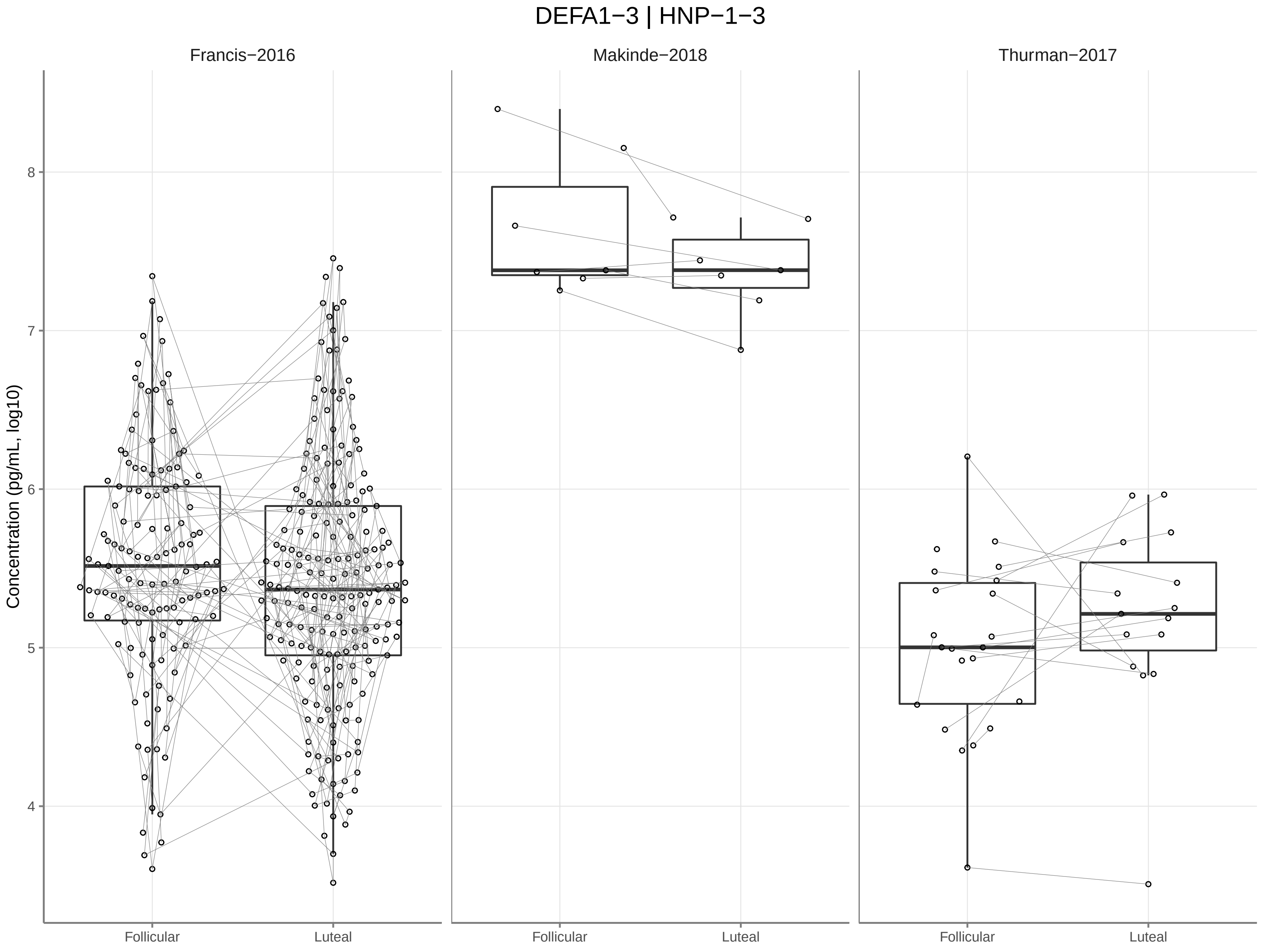

DEFA1−3 | HNP−1−3
Francis−2016
Makinde−2018
Thurman−2017
Concentration (pg/mL, log10)
8
7
6
5
4
Follicular
Luteal
Follicular
Luteal
Follicular
Luteal

**DEFA1−3**	**TE**	**seTE**	**95%−CI**	**Weight**
Makinde−2018	−0.89	0.3346	−0.89 [−1.55; −0.24]	26.8%
Francis−2016	−0.29	0.2406	−0.29 [−0.77; 0.18]	29.7%
Thurman−2017	0.45	0.4965	0.45 [−0.53; 1.42]	21.5%
Shust−2010	0.88	0.4802	0.88 [−0.06; 1.82]	22.0%
**Random effects model**			**−0.04 [−0.79; 0.71]**	**100.0%**

Heterogeneity: I^2 = 73%

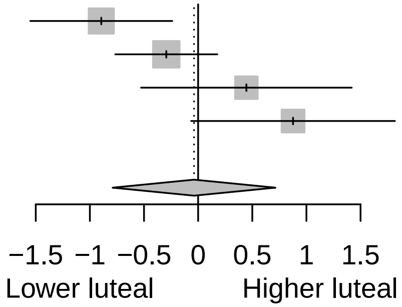

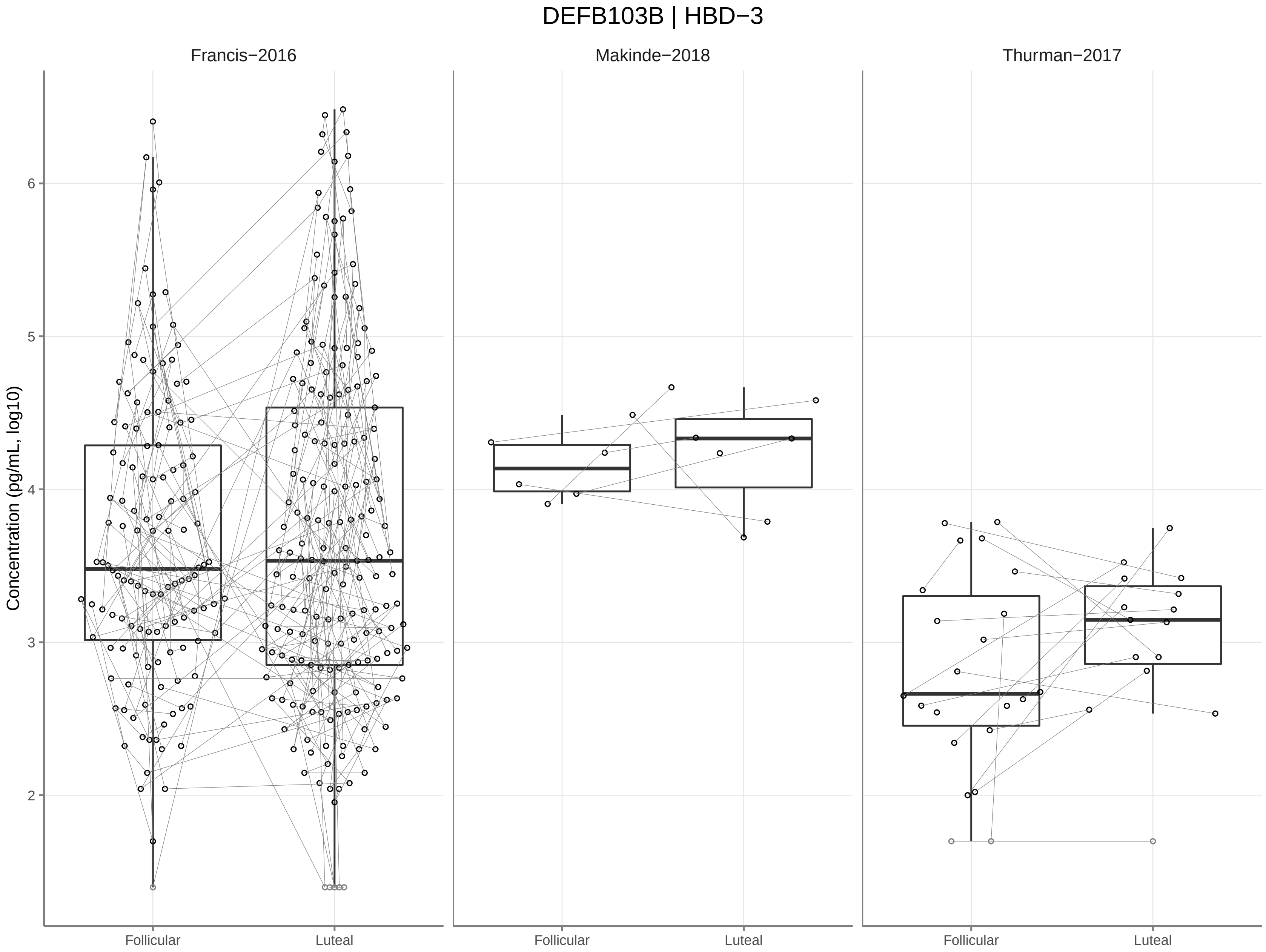

DEFB103B | HBD−3
Francis−2016
Makinde−2018
Thurman−2017
Concentration (pg/mL, log10)
6
5
4
3
2
Follicular
Luteal
Follicular
Luteal
Follicular
Luteal

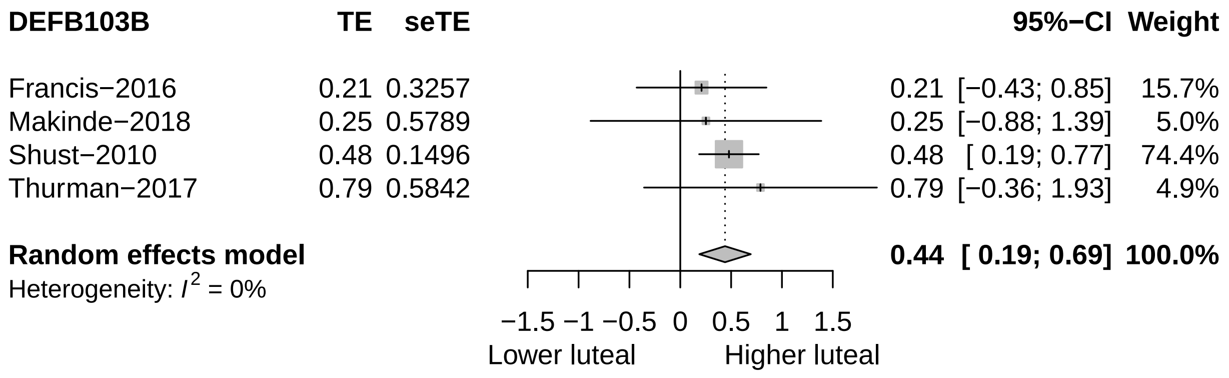
DEFB103B
TE
seTE
95%−CI
Weight
Francis−2016 0.21 0.3257 0.21 [−0.43; 0.85] 15.7%
Makinde−2018 0.25 0.5789 0.25 [−0.88; 1.39] 5.0%
Shust−2010 0.48 0.1496 0.48 [0.19; 0.77] 74.4%
Thurman−2017 0.79 0.5842 0.79 [−0.36; 1.93] 4.9%
Random effects model 0.44 [0.19; 0.69] 100.0%
Heterogeneity: I2 = 0%
−1.5 −1 −0.5 0 0.5 1 1.5
Lower luteal
Higher luteal

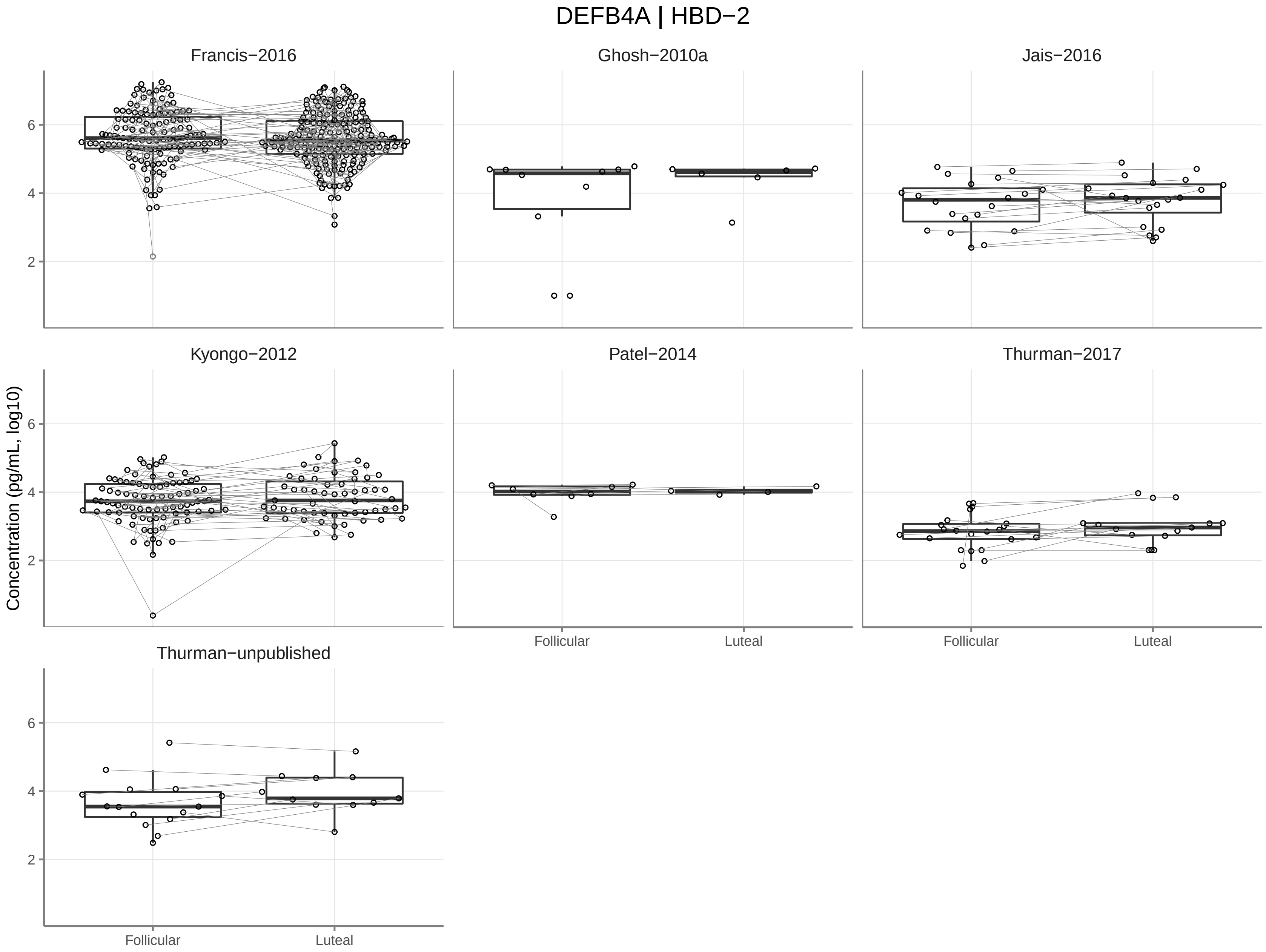

DEFB4A | HBD−2
Francis−2016
Ghosh−2010a
Jais−2016
Kyongo−2012
Patel−2014
Thurman−2017
Thurman−unpublished
Concentration (pg/mL, log10)
2
4
6
Follicular
Luteal

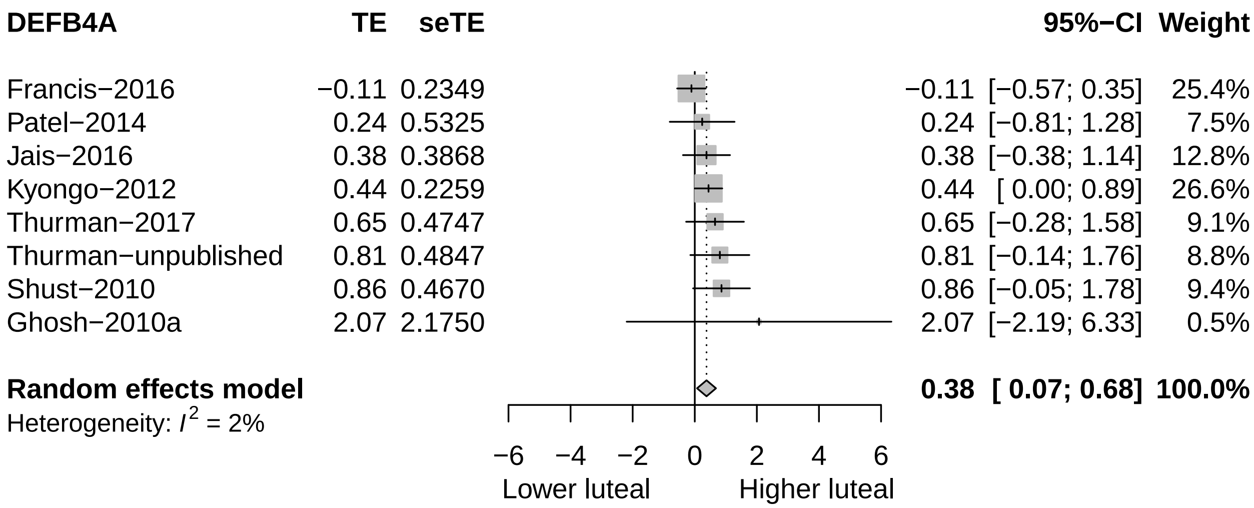
DEFB4A
TE seTE
95%−CI Weight
Francis−2016 −0.11 0.2349 −0.11 [−0.57; 0.35] 25.4%
Patel−2014 0.24 0.5325 0.24 [−0.81; 1.28] 7.5%
Jais−2016 0.38 0.3868 0.38 [−0.38; 1.14] 12.8%
Kyongo−2012 0.44 0.2259 0.44 [0.00; 0.89] 26.6%
Thurman−2017 0.65 0.4747 0.65 [−0.28; 1.58] 9.1%
Thurman−unpublished 0.81 0.4847 0.81 [−0.14; 1.76] 8.8%
Shust−2010 0.86 0.4670 0.86 [−0.05; 1.78] 9.4%
Ghosh−2010a 2.07 2.1750 2.07 [−2.19; 6.33] 0.5%
Random effects model 0.38 [0.07; 0.68] 100.0%
Heterogeneity: I² = 2%
−6 −4 −2 0 2 4 6
Lower luteal
Higher luteal

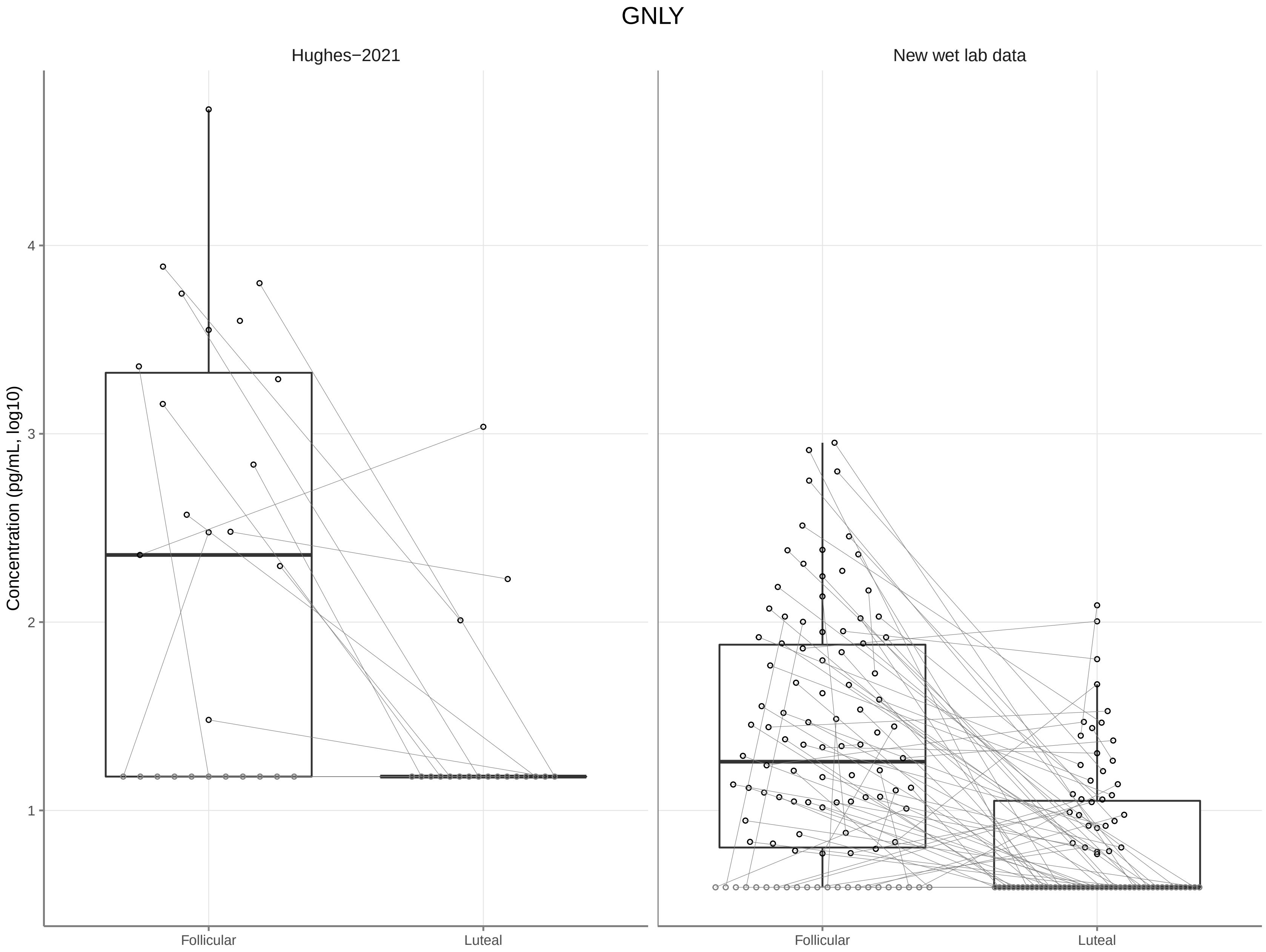
GNLY
Hughes−2021
New wet lab data
Concentration (pg/mL, log10)
4
3
2
1
Follicular
Luteal
Follicular
Luteal

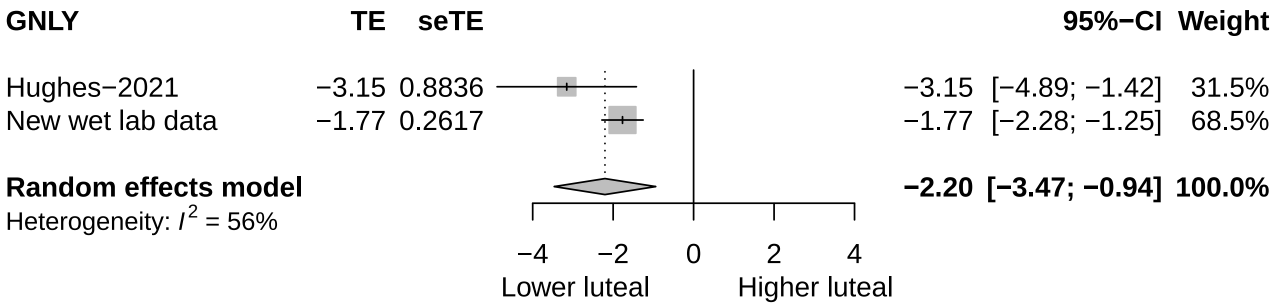
GNLY
TE seTE
95%−CI Weight
Hughes−2021 −3.15 0.8836 −3.15 [−4.89; −1.42] 31.5%
New wet lab data −1.77 0.2617 −1.77 [−2.28; −1.25] 68.5%
Random effects model −2.20 [−3.47; −0.94] 100.0%
Heterogeneity: I2 = 56%
−4 −2 0 2 4
Lower luteal
Higher luteal

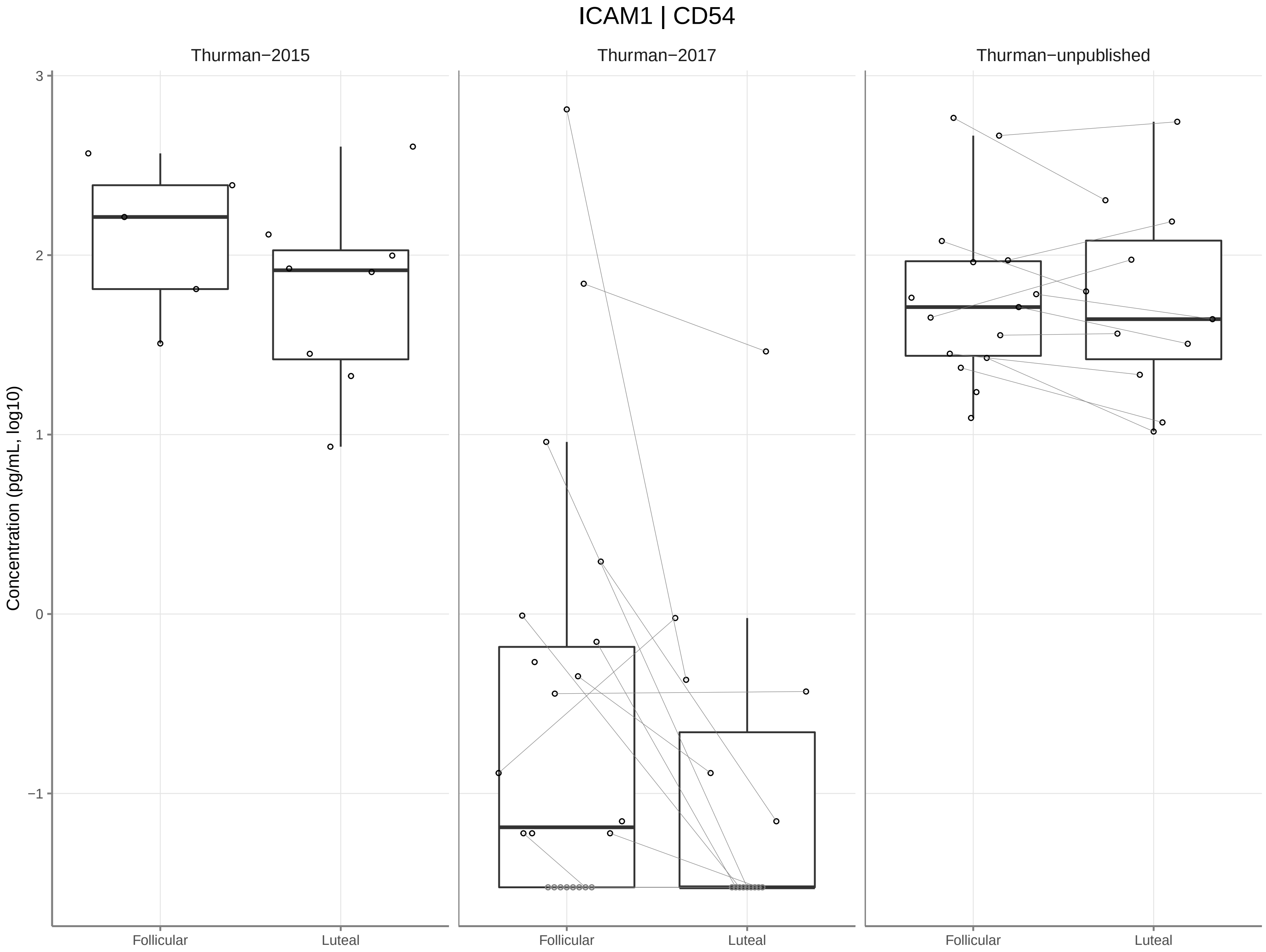

ICAM1 | CD54
Thurman−2015
Thurman−2017
Thurman−unpublished
Concentration (pg/mL, log10)
3
2
1
0
−1
Follicular
Luteal
Follicular
Luteal
Follicular
Luteal

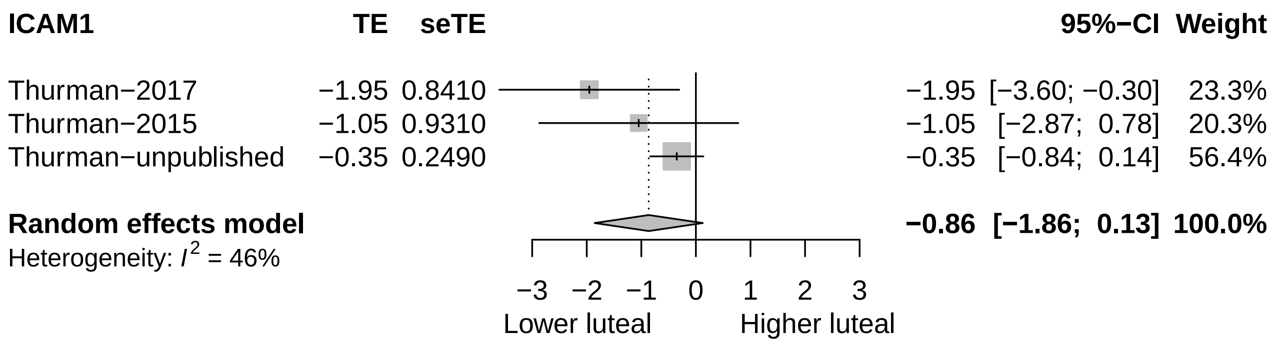
ICAM1
TE
seTE
95%−CI
Weight
Thurman−2017 −1.95 0.8410 −1.95 [−3.60; −0.30] 23.3%
Thurman−2015 −1.05 0.9310 −1.05 [−2.87; 0.78] 20.3%
Thurman−unpublished −0.35 0.2490 −0.35 [−0.84; 0.14] 56.4%
Random effects model −0.86 [−1.86; 0.13] 100.0%
Heterogeneity: I2 = 46%
−3 −2 −1 0 1 2 3
Lower luteal
Higher luteal

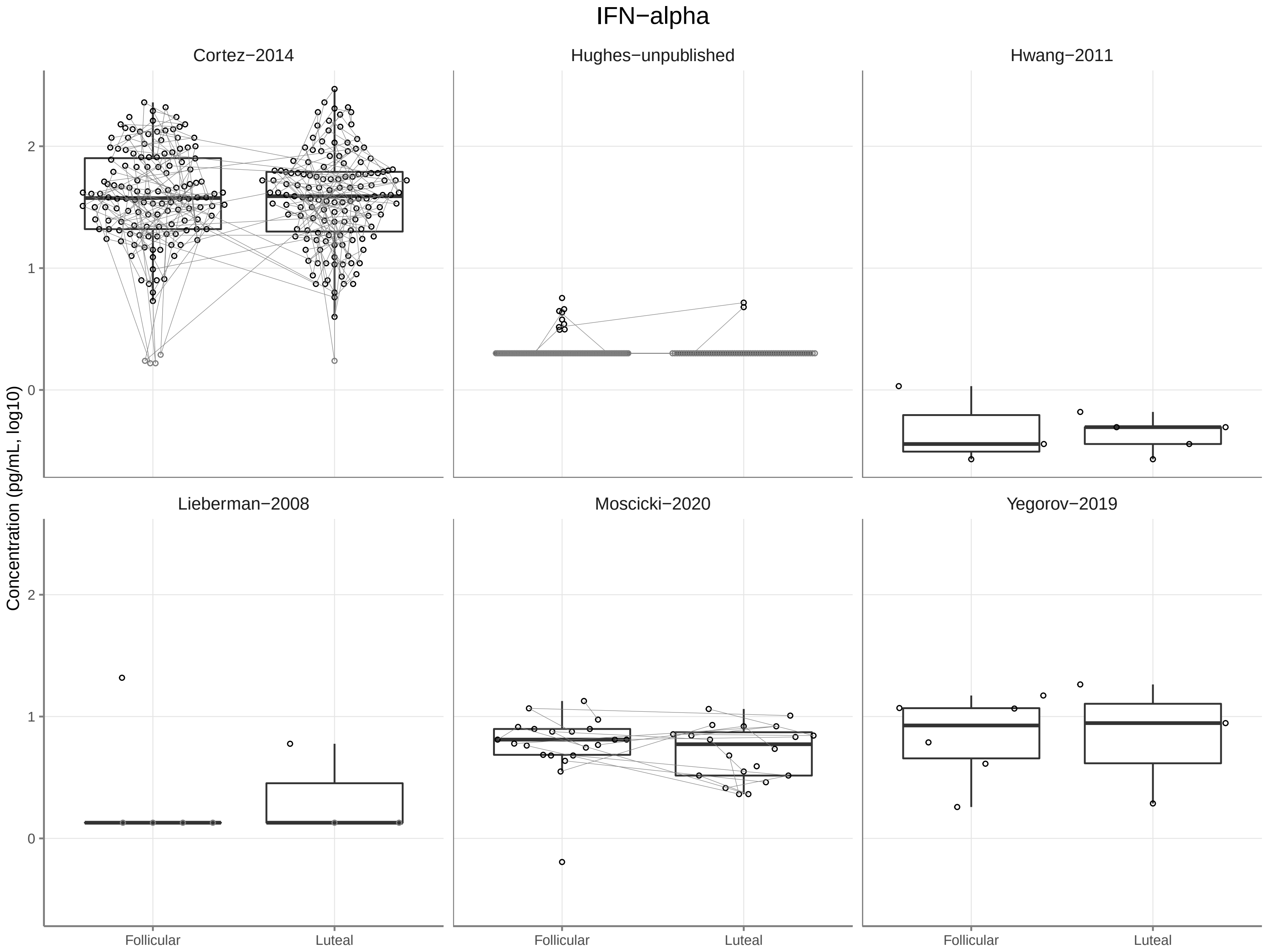

IFN−alpha
Cortez−2014
Hughes−unpublished
Hwang−2011
Lieberman−2008
Moscicki−2020
Yegorov−2019
Concentration (pg/mL, log10)
0
1
2
Follicular
Luteal

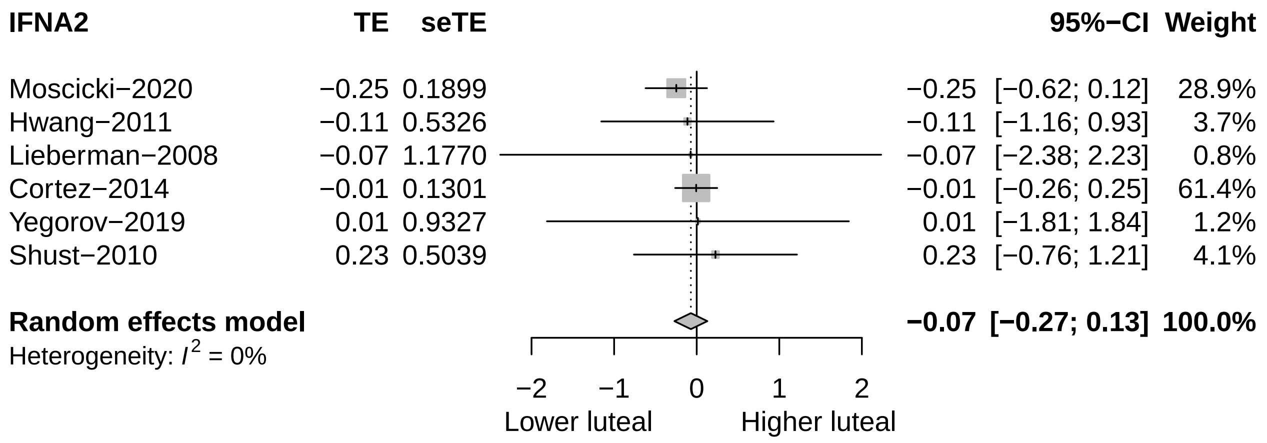
IFNA2
TE
seTE
95%−CI
Weight
Moscicki−2020 −0.25 0.1899 −0.25 [−0.62; 0.12] 28.9%
Hwang−2011 −0.11 0.5326 −0.11 [−1.16; 0.93] 3.7%
Lieberman−2008 −0.07 1.1770 −0.07 [−2.38; 2.23] 0.8%
Cortez−2014 −0.01 0.1301 −0.01 [−0.26; 0.25] 61.4%
Yegorov−2019 0.01 0.9327 0.01 [−1.81; 1.84] 1.2%
Shust−2010 0.23 0.5039 0.23 [−0.76; 1.21] 4.1%
Random effects model −0.07 [−0.27; 0.13] 100.0%
Heterogeneity: I2 = 0%
−2
−1
0
1
2
Lower luteal
Higher luteal

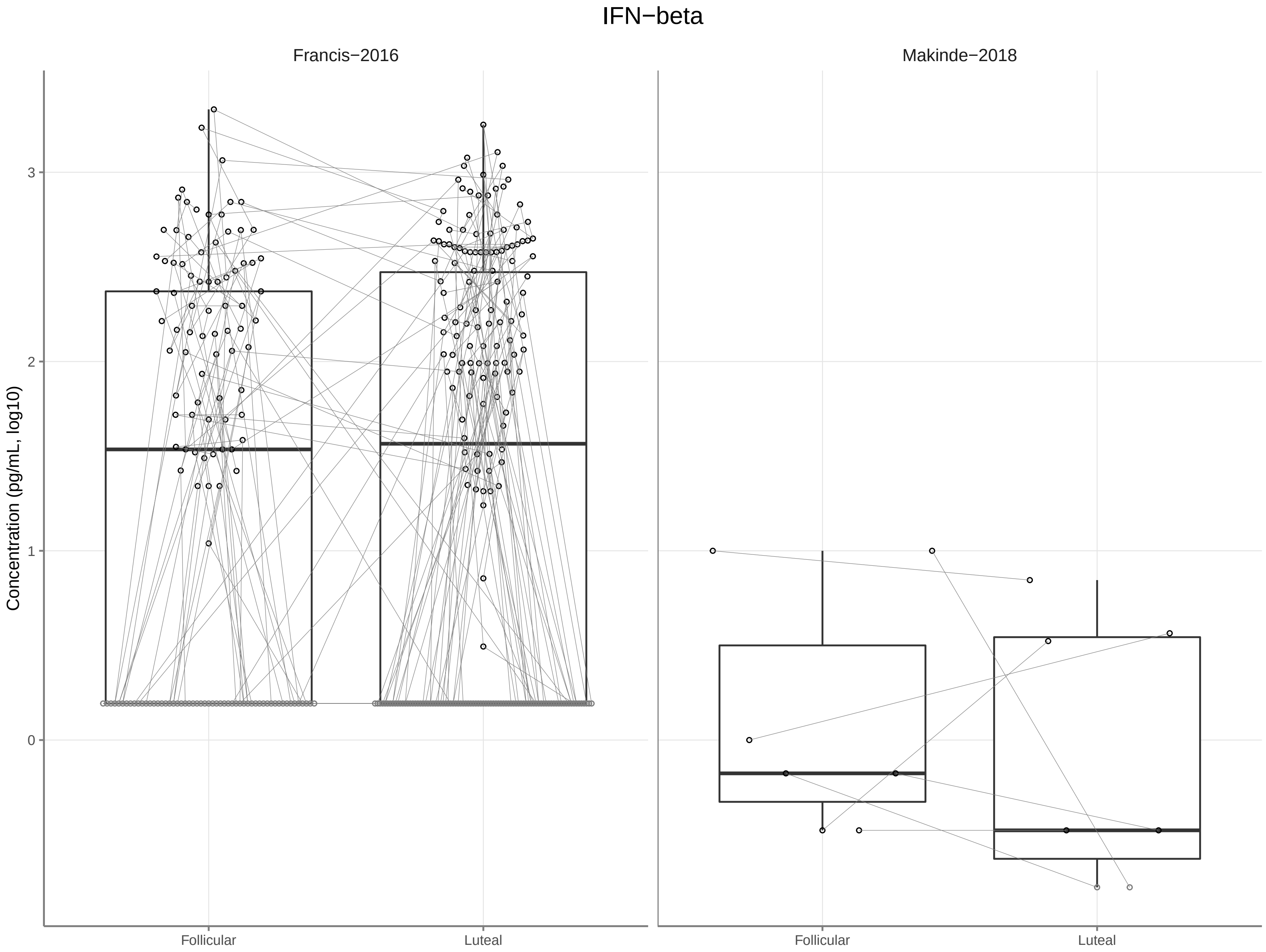

IFN−beta
Francis−2016
Makinde−2018
Concentration (pg/mL, log10)
3
2
1
0
Follicular
Luteal
Follicular
Luteal

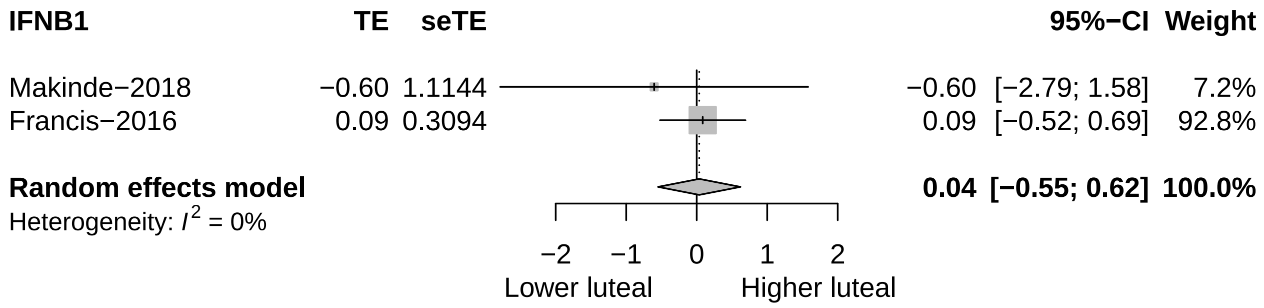
IFNB1
TE
seTE
95%−CI
Weight
Makinde−2018
−0.60
1.1144
−0.60
[−2.79; 1.58]
7.2%
Francis−2016
0.09
0.3094
0.09
[−0.52; 0.69]
92.8%
Random effects model
0.04
[−0.55; 0.62]
100.0%
Heterogeneity: I^2 = 0%
−2
−1
0
1
2
Lower luteal
Higher luteal

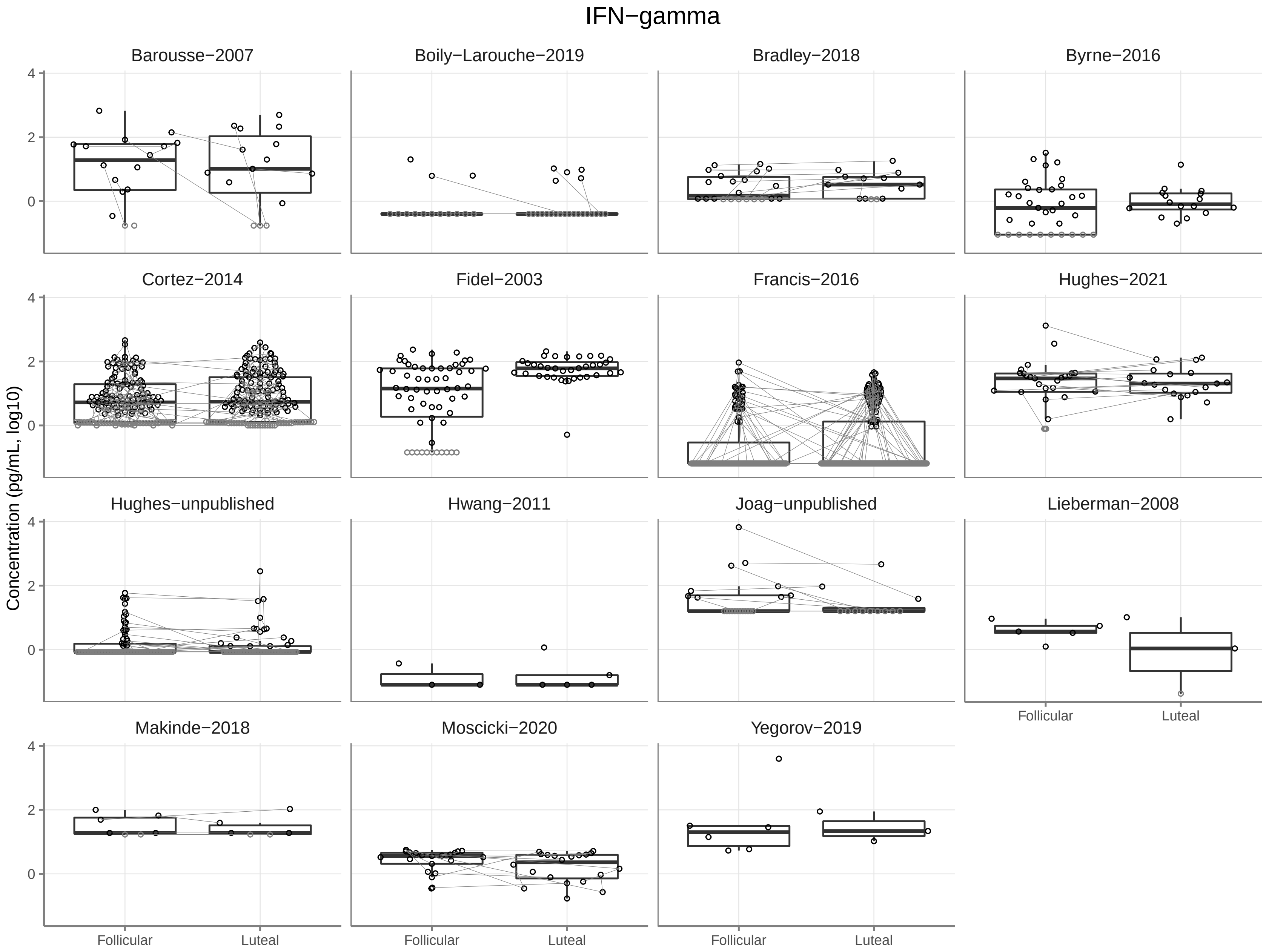
IFN−gamma
Barousse−2007
Boily−Larouche−2019
Bradley−2018
Byrne−2016
Cortez−2014
Fidel−2003
Francis−2016
Hughes−2021
Hughes−unpublished
Hwang−2011
Joag−unpublished
Lieberman−2008
Makinde−2018
Moscicki−2020
Yegorov−2019
Concentration (pg/mL, log10)
4
2
0
Follicular
Luteal

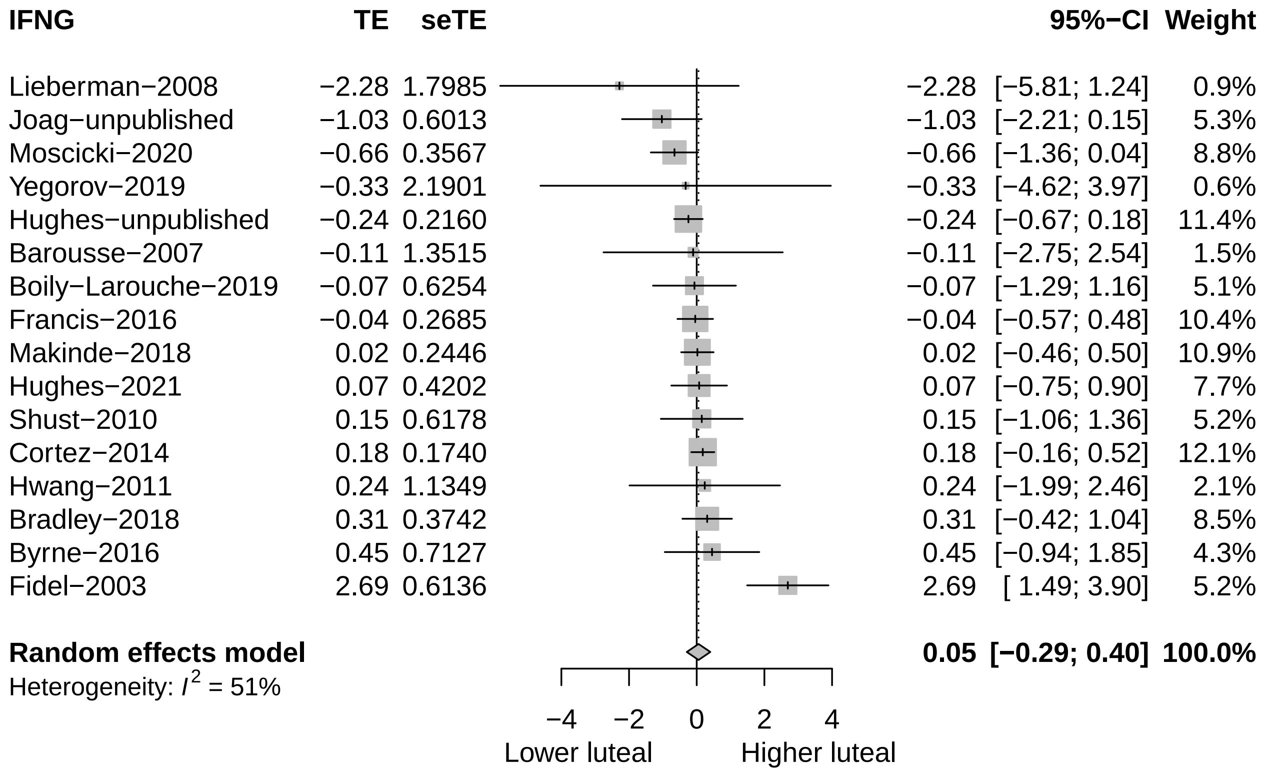
IFNG
TE seTE
95%−CI Weight
Lieberman−2008 −2.28 1.7985 −2.28 [−5.81; 1.24] 0.9%
Joag−unpublished −1.03 0.6013 −1.03 [−2.21; 0.15] 5.3%
Moscicki−2020 −0.66 0.3567 −0.66 [−1.36; 0.04] 8.8%
Yegorov−2019 −0.33 2.1901 −0.33 [−4.62; 3.97] 0.6%
Hughes−unpublished −0.24 0.2160 −0.24 [−0.67; 0.18] 11.4%
Barousse−2007 −0.11 1.3515 −0.11 [−2.75; 2.54] 1.5%
Boily−Larouche−2019 −0.07 0.6254 −0.07 [−1.29; 1.16] 5.1%
Francis−2016 −0.04 0.2685 −0.04 [−0.57; 0.48] 10.4%
Makinde−2018 0.02 0.2446 0.02 [−0.46; 0.50] 10.9%
Hughes−2021 0.07 0.4202 0.07 [−0.75; 0.90] 7.7%
Shust−2010 0.15 0.6178 0.15 [−1.06; 1.36] 5.2%
Cortez−2014 0.18 0.1740 0.18 [−0.16; 0.52] 12.1%
Hwang−2011 0.24 1.1349 0.24 [−1.99; 2.46] 2.1%
Bradley−2018 0.31 0.3742 0.31 [−0.42; 1.04] 8.5%
Byrne−2016 0.45 0.7127 0.45 [−0.94; 1.85] 4.3%
Fidel−2003 2.69 0.6136 2.69 [1.49; 3.90] 5.2%
Random effects model 0.05 [−0.29; 0.40] 100.0%
Heterogeneity: I2 = 51%
−4 −2 0 2 4
Lower luteal
Higher luteal

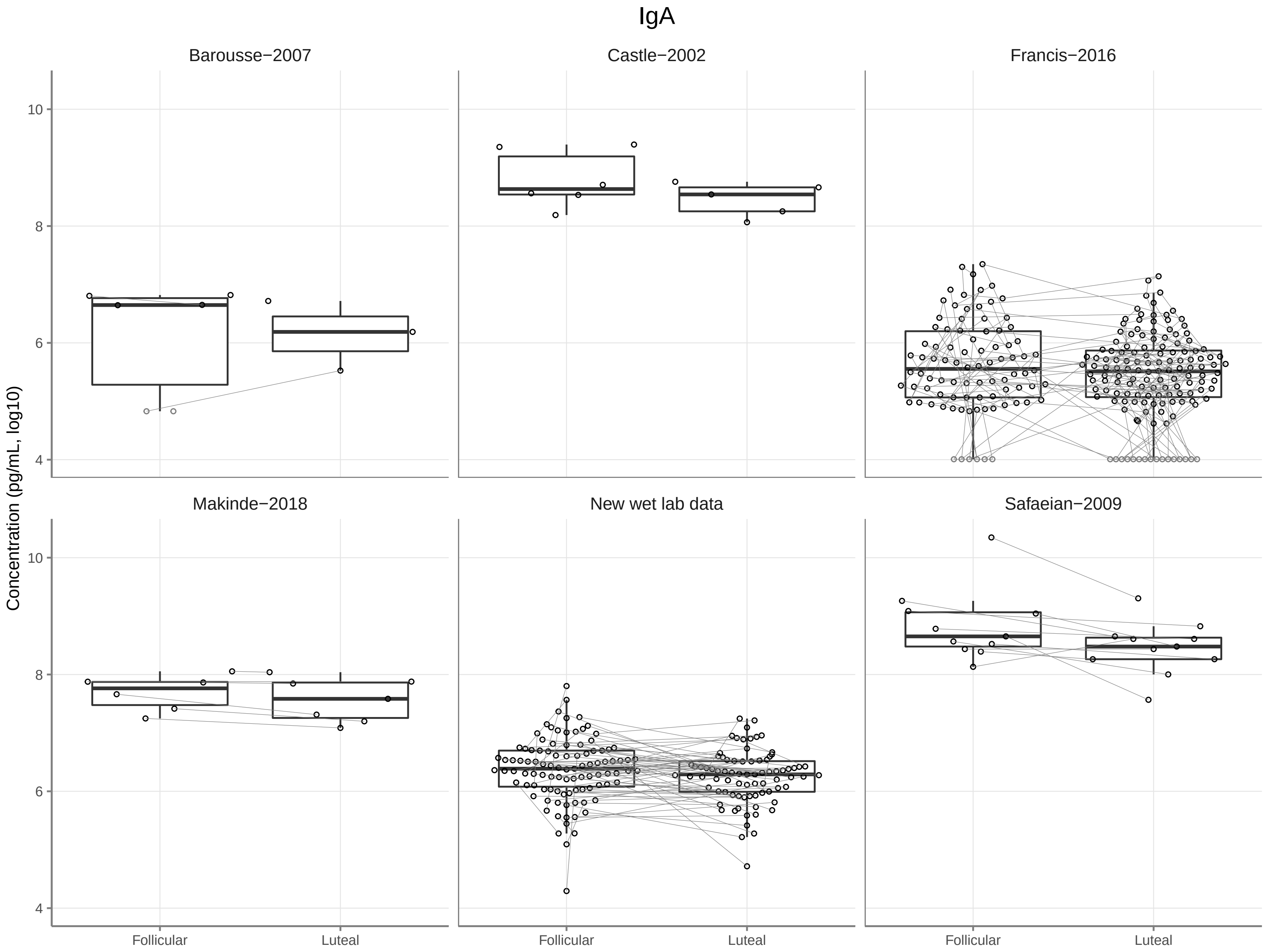
IgA
Barousse−2007
Castle−2002
Francis−2016
Makinde−2018
New wet lab data
Safaeian−2009
Concentration (pg/mL, log10)
10
8
6
4
Follicular
Luteal

IgA	TE	seTE		95%−CI	Weight
Safaeian−2009	−1.27	0.4557	−1.27	[−2.17; −0.38]	13.8%
Castle−2002	−1.11	0.8234	−1.11	[−2.72; 0.51]	10.0%
Shust−2010	−0.67	0.2628	−0.67	[−1.18; −0.15]	15.5%
Francis−2016	−0.56	0.2595	−0.56	[−1.07; −0.06]	15.6%
New wet lab data	−0.45	0.2305	−0.45	[−0.90; 0.00]	15.8%
Makinde−2018	−0.42	0.1900	−0.42	[−0.79; −0.05]	16.0%
Barousse−2007	2.23	0.4998	2.23	[1.25; 3.21]	13.3%
**Random effects model**			**−0.32**	**[−1.12; 0.49]**	**100.0%**

Heterogeneity: I^2 = 82%

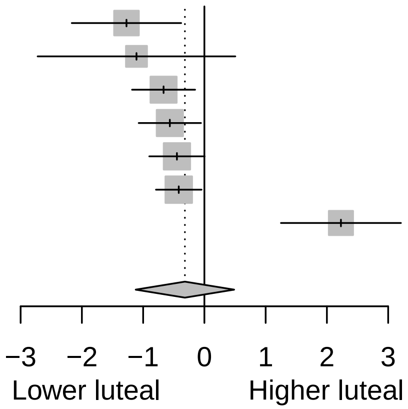

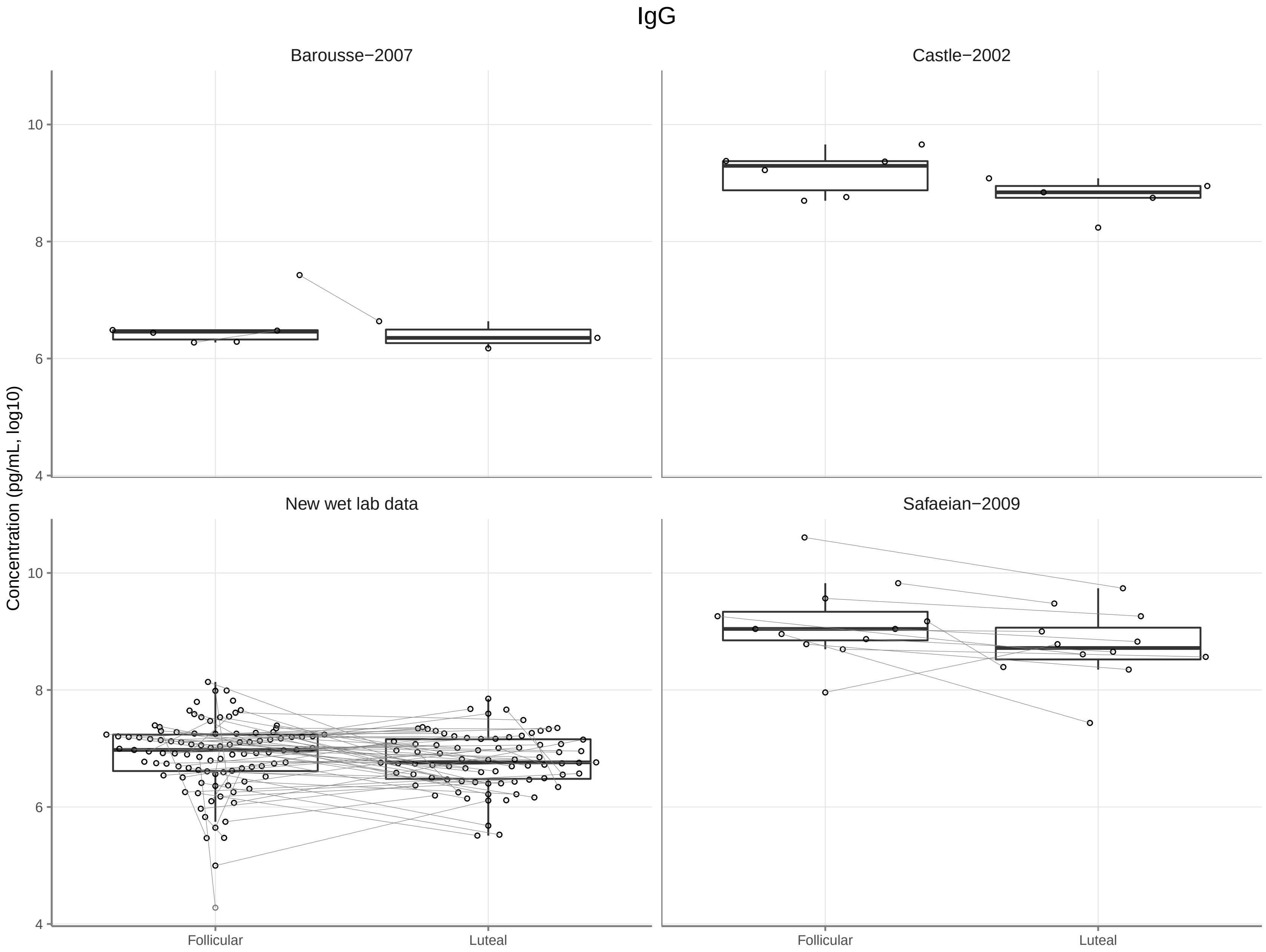

IgG
Barousse−2007
Castle−2002
New wet lab data
Safaeian−2009
Concentration (pg/mL, log10)
10
8
6
4
Follicular
Luteal

IgG	TE	seTE	95%−CI	Weight
Barousse−2007	−1.53	0.7135	−1.53 [−2.93; −0.13]	12.0%
Castle−2002	−1.36	0.7131	−1.36 [−2.76; 0.04]	12.0%
Safaeian−2009	−1.30	0.5381	−1.30 [−2.35; −0.24]	16.8%
New wet lab data	−0.34	0.2548	−0.34 [−0.84; 0.16]	28.4%
Shust−2010	−0.09	0.2031	−0.09 [−0.49; 0.31]	30.7%
**Random effects model**			**−0.69 [−1.29; −0.09]**	**100.0%**

Heterogeneity: I^2 = 58%

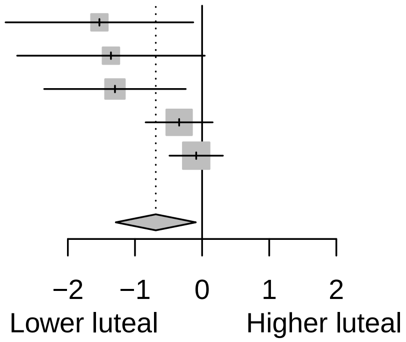

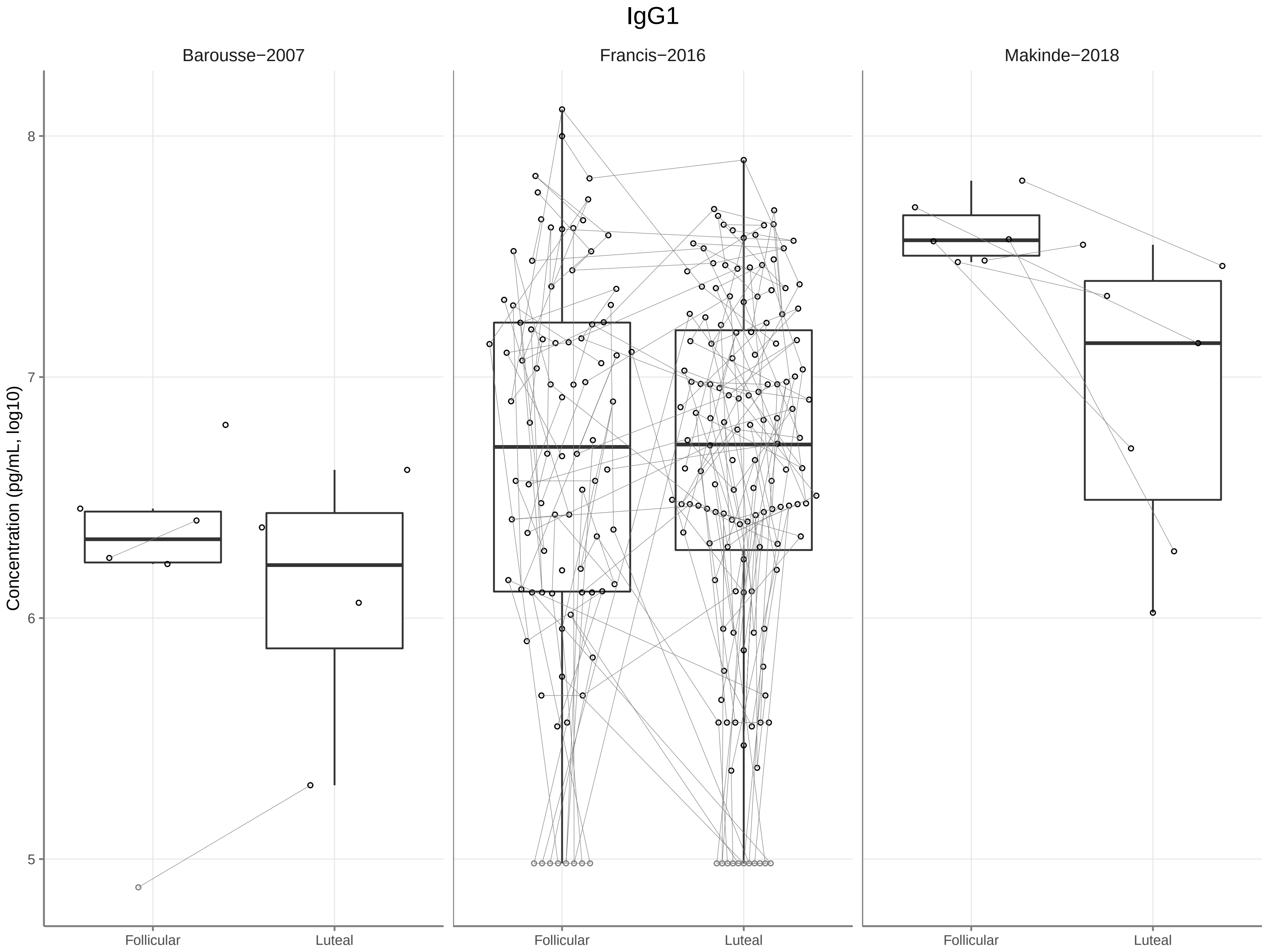
IgG1
Barousse−2007
Francis−2016
Makinde−2018
Concentration (pg/mL, log10)
8
7
6
5
Follicular
Luteal
Follicular
Luteal
Follicular
Luteal

IgG1	TE	seTE	95%−CI	Weight
Makinde−2018	−2.18	0.7863	−2.18 [−3.72; −0.63]	29.5%
Francis−2016	−0.27	0.2628	−0.27 [−0.78; 0.25]	36.5%
Barousse−2007	1.20	0.4881	1.20 [0.24; 2.15]	34.0%
**Random effects model**			**−0.33 [−2.14; 1.48]**	**100.0%**

Heterogeneity: I^2 = 86%

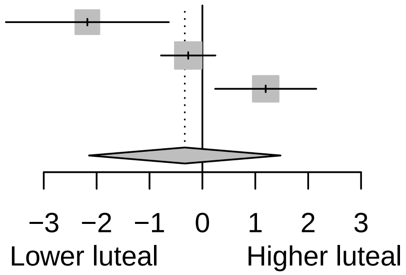

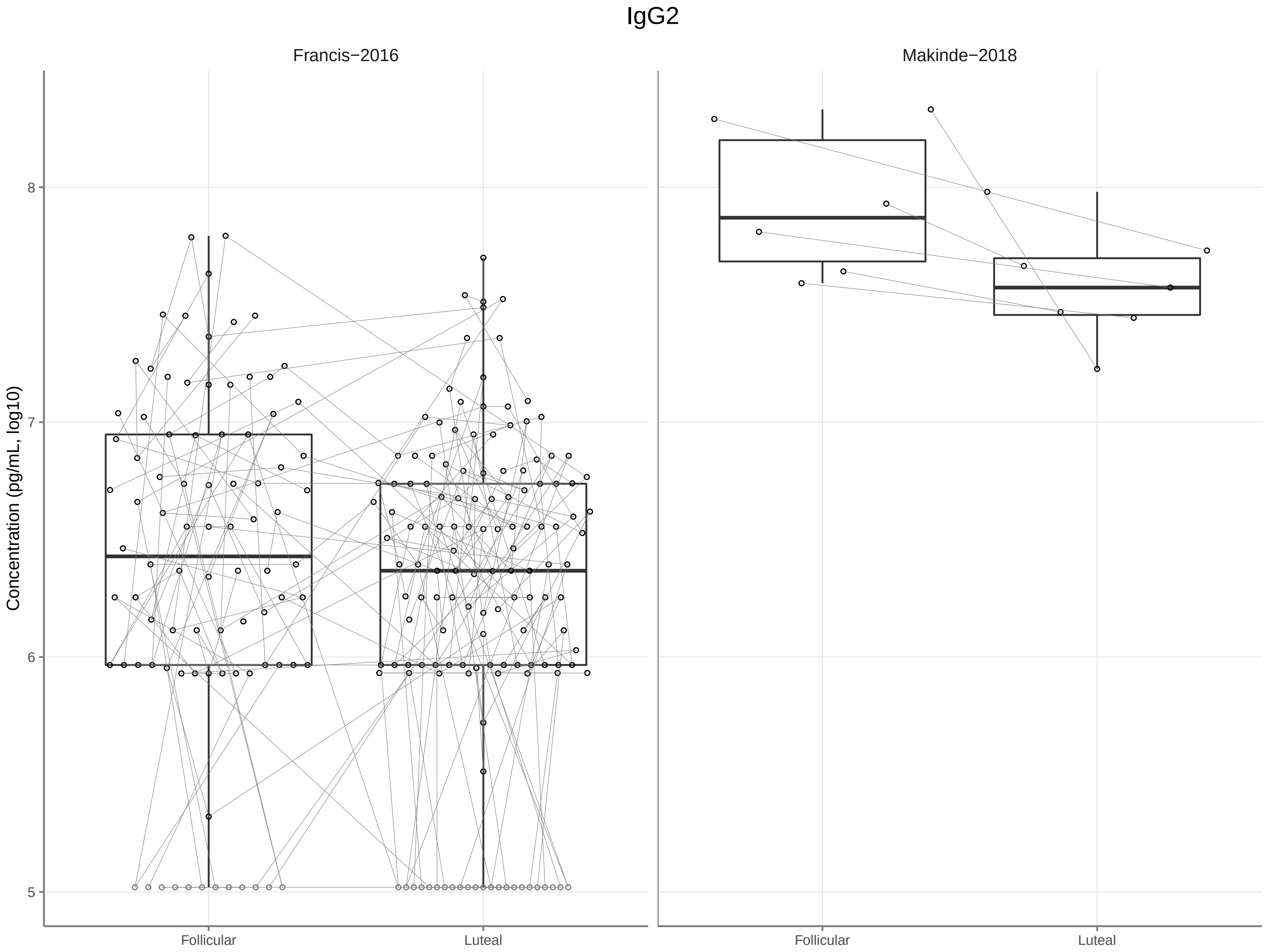
IgG2
Francis−2016
Makinde−2018
Concentration (pg/mL, log10)
8
7
6
5
Follicular
Luteal
Follicular
Luteal

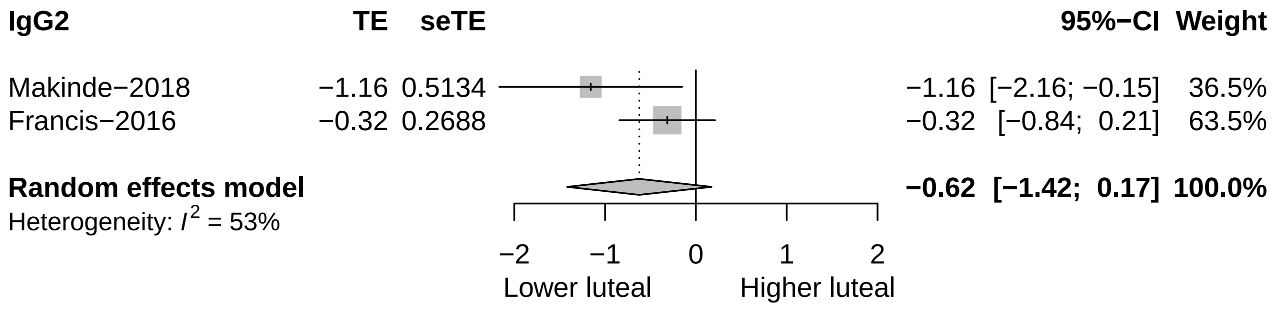
IgG2
TE
seTE
95%−CI
Weight
Makinde−2018 −1.16 0.5134 −1.16 [−2.16; −0.15] 36.5%
Francis−2016 −0.32 0.2688 −0.32 [−0.84; 0.21] 63.5%
Random effects model −0.62 [−1.42; 0.17] 100.0%
Heterogeneity: I^2 = 53%
−2
−1
0
1
2
Lower luteal
Higher luteal

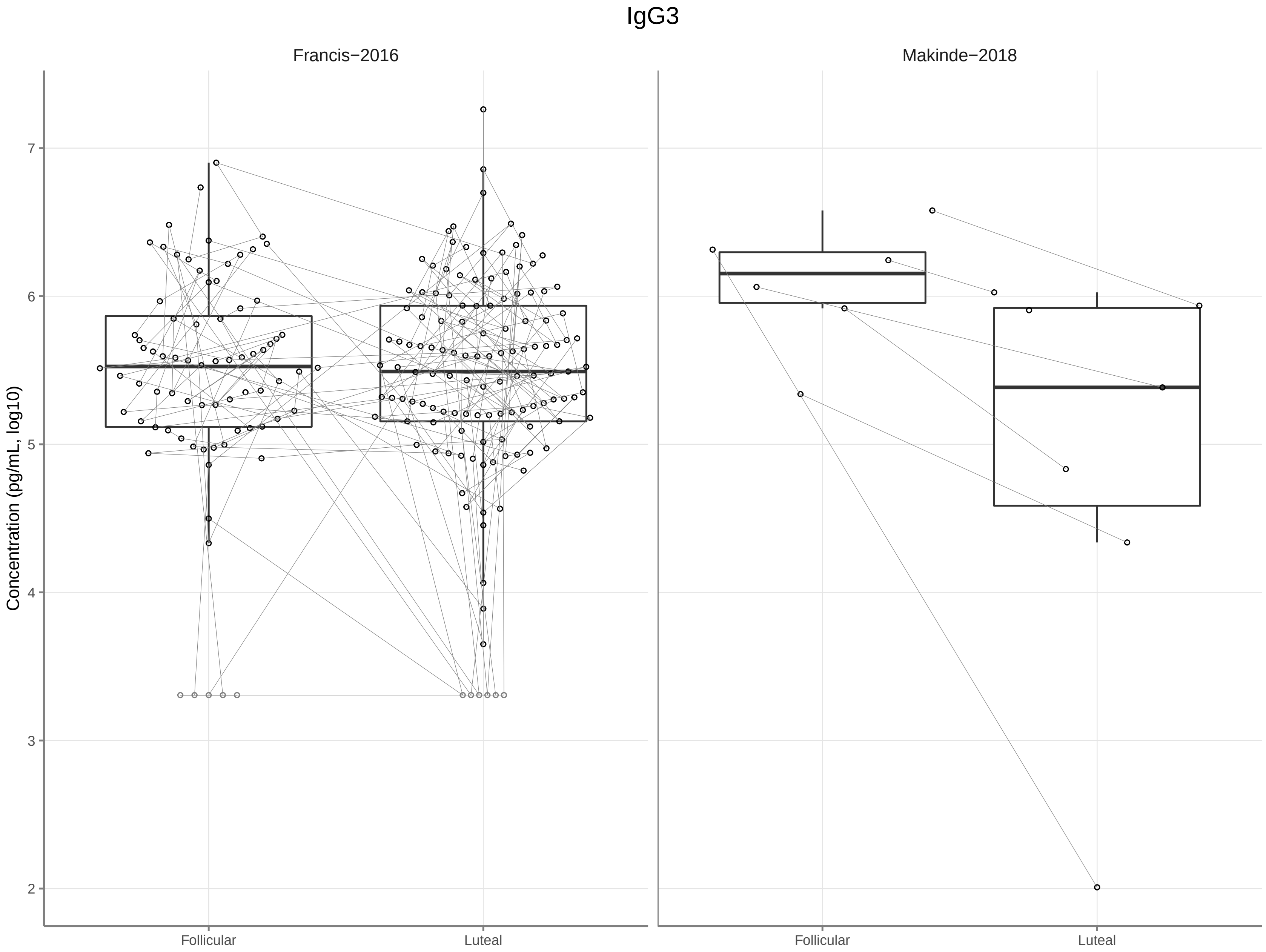

IgG3
Francis−2016
Makinde−2018
Concentration (pg/mL, log10)
7
6
5
4
3
2
Follicular
Luteal
Follicular
Luteal

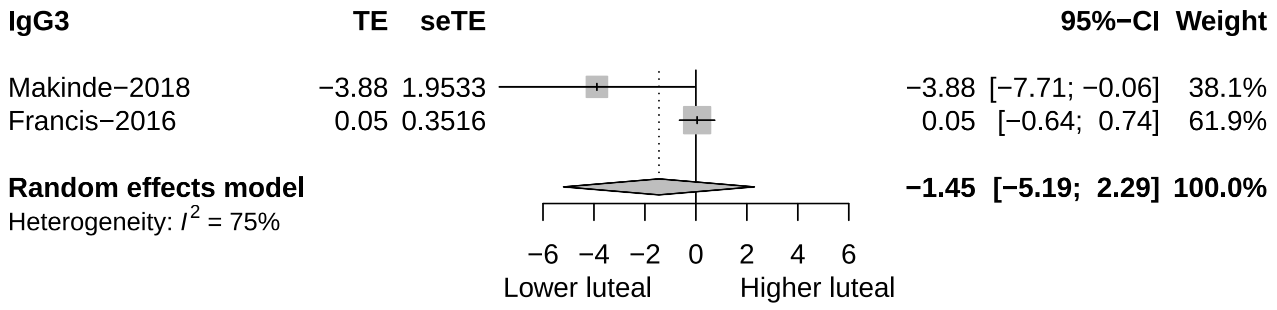
IgG3
TE
seTE
95%−CI
Weight
Makinde−2018
−3.88
1.9533
−3.88 [−7.71; −0.06]
38.1%
Francis−2016
0.05
0.3516
0.05 [−0.64; 0.74]
61.9%
Random effects model
−1.45 [−5.19; 2.29]
100.0%
Heterogeneity: I^2 = 75%
−6 −4 −2 0 2 4 6
Lower luteal
Higher luteal

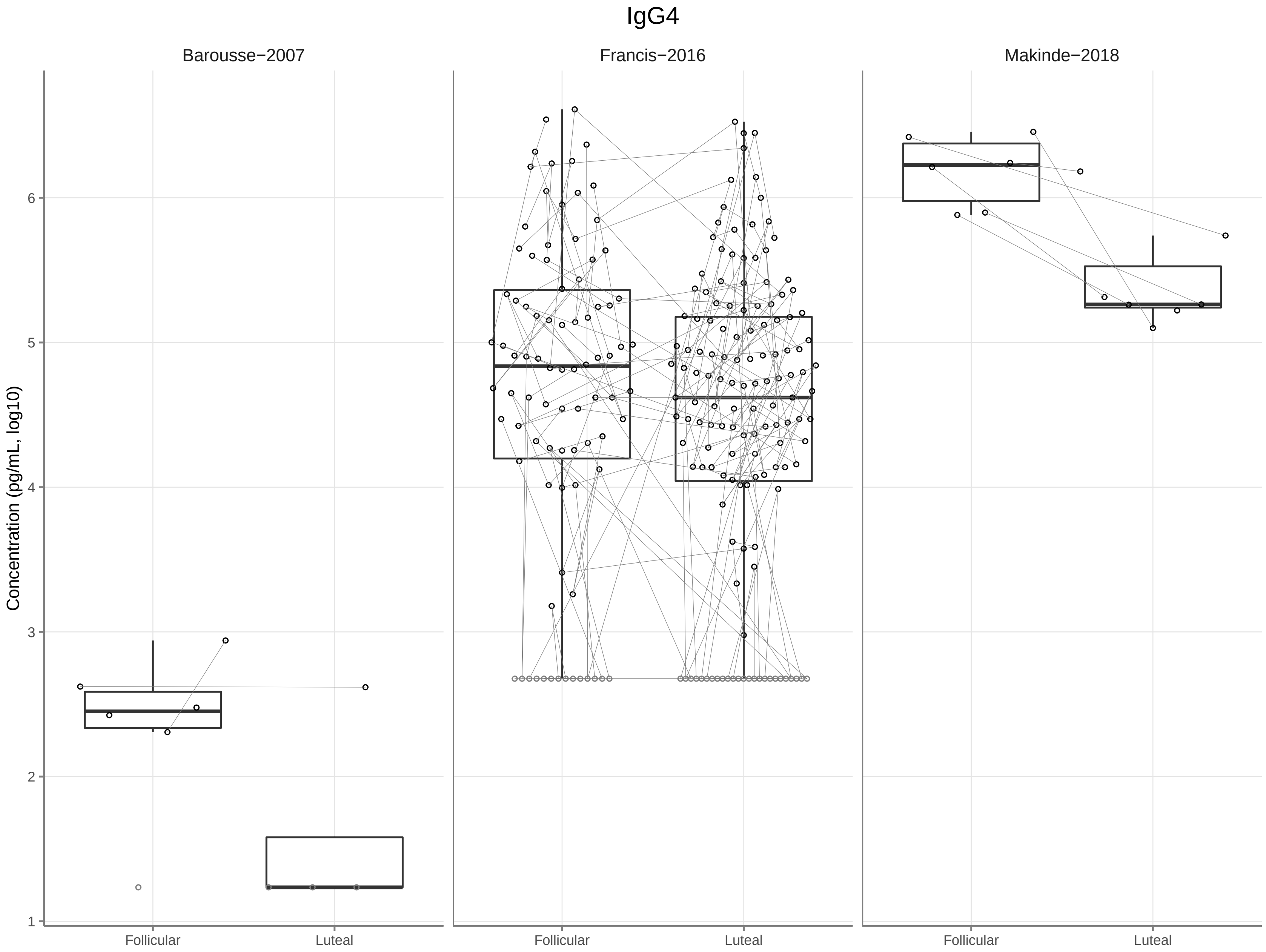

IgG4
Barousse−2007
Francis−2016
Makinde−2018
Concentration (pg/mL, log10)
1
2
3
4
5
6
Follicular
Luteal
Follicular
Luteal
Follicular
Luteal

IgG4	TE	seTE	95%−CI	Weight
Makinde−2018	−2.45	0.5454	−2.45 [−3.52; −1.38]	37.3%
Barousse−2007	−2.01	1.2501	−2.01 [−4.46; 0.44]	15.9%
Francis−2016	−0.90	0.3282	−0.90 [−1.54; −0.26]	46.8%
**Random effects model**			**−1.65 [−2.81; −0.50]**	**100.0%**

Heterogeneity: I^2 = 68%

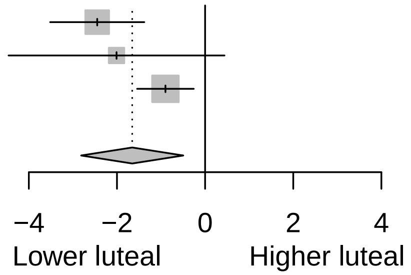

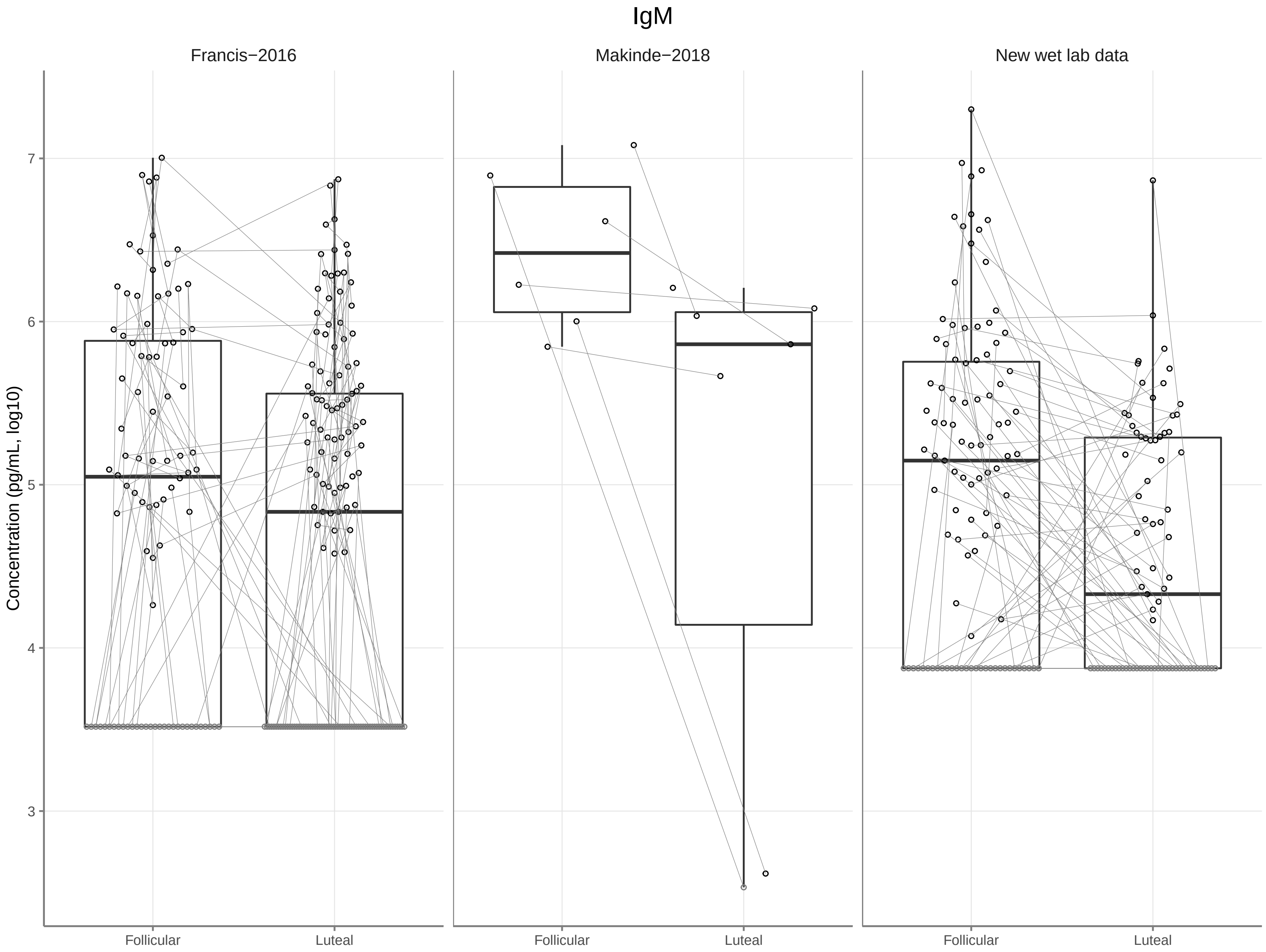
IgM
Francis−2016
Makinde−2018
New wet lab data
Concentration (pg/mL, log10)
7
6
5
4
3
Follicular
Luteal
Follicular
Luteal
Follicular
Luteal

IgM	TE	seTE	95%−CI	Weight
Makinde−2018	−4.81	2.3413	−4.81 [−9.40; −0.22]	2.0%
New wet lab data	−1.60	0.4312	−1.60 [−2.45; −0.76]	42.5%
Francis−2016	−0.98	0.3551	−0.98 [−1.67; −0.28]	55.5%
**Random effects model**			**−1.32 [−1.96; −0.67]**	**100.0%**
Heterogeneity: I^2 = 44%				

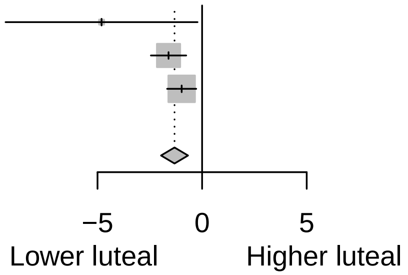

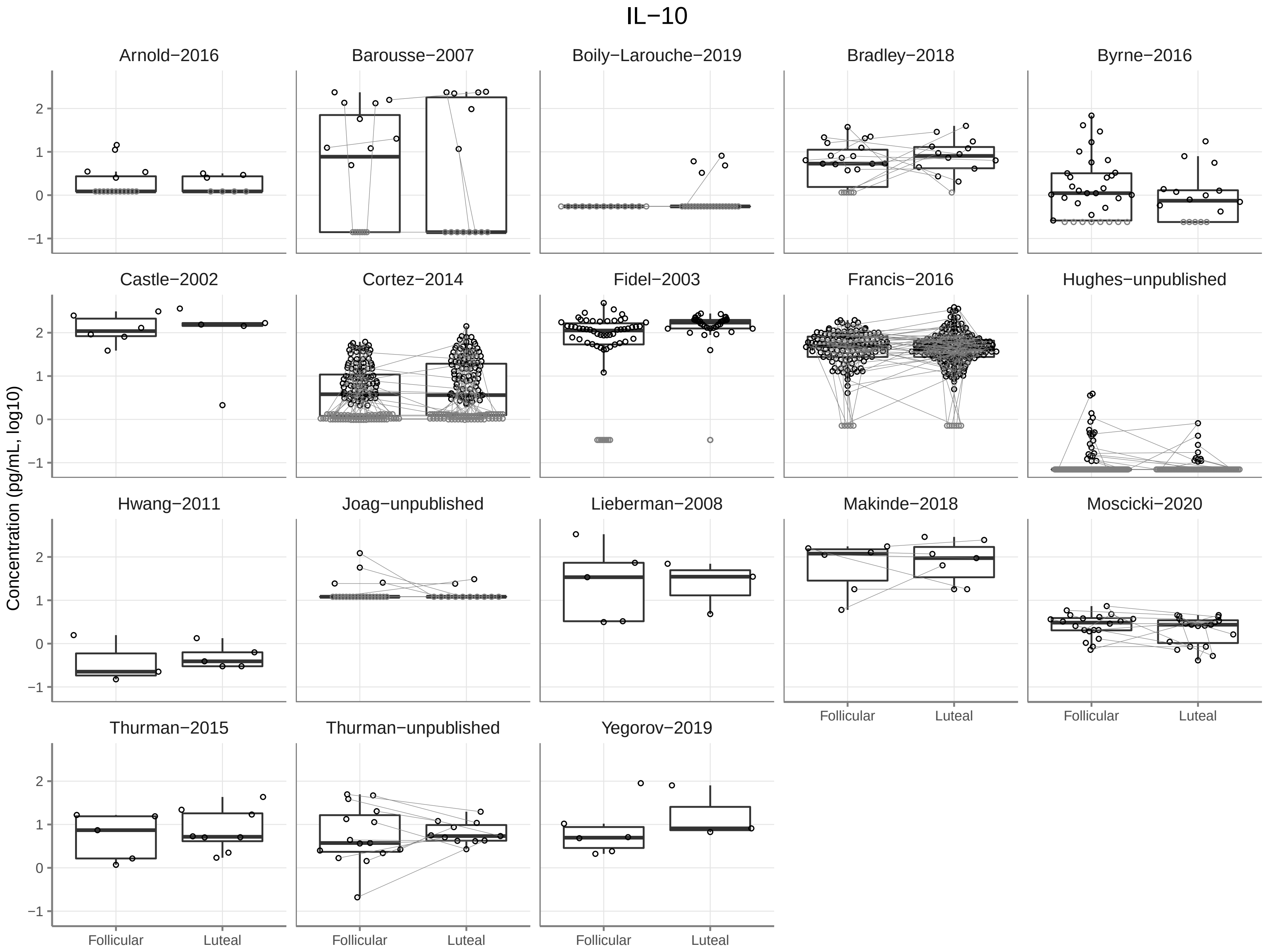

IL−10
Arnold−2016
Barousse−2007
Boily−Larouche−2019
Bradley−2018
Byrne−2016
Castle−2002
Cortez−2014
Fidel−2003
Francis−2016
Hughes−unpublished
Hwang−2011
Joag−unpublished
Lieberman−2008
Makinde−2018
Moscicki−2020
Thurman−2015
Thurman−unpublished
Yegorov−2019
Concentration (pg/mL, log10)
Follicular
Luteal

IL10	TE	seTE	95%−CI	Weight
Byrne−2016	−0.66	0.6778	−0.66 [−1.99; 0.67]	2.4%
Castle−2002	−0.62	1.2919	−0.62 [−3.15; 1.92]	0.7%
Barousse−2007	−0.38	1.7765	−0.38 [−3.86; 3.10]	0.4%
Hughes−unpublished	−0.37	0.1655	−0.37 [−0.70; −0.05]	15.9%
Moscicki−2020	−0.34	0.2827	−0.34 [−0.89; 0.22]	9.4%
Joag−unpublished	−0.17	0.2753	−0.17 [−0.71; 0.37]	9.7%
Arnold−2016	−0.15	0.4831	−0.15 [−1.09; 0.80]	4.3%
Lieberman−2008	−0.10	1.9358	−0.10 [−3.90; 3.69]	0.3%
Francis−2016	0.07	0.1409	0.07 [−0.20; 0.35]	17.6%
Thurman−unpublished	0.17	0.6070	0.17 [−1.02; 1.36]	2.9%
Cortez−2014	0.21	0.1528	0.21 [−0.09; 0.51]	16.8%
Makinde−2018	0.28	0.8402	0.28 [−1.37; 1.93]	1.6%
Hwang−2011	0.40	0.9376	0.40 [−1.44; 2.23]	1.3%
Thurman−2015	0.51	0.9650	0.51 [−1.38; 2.40]	1.2%
Bradley−2018	0.55	0.4270	0.55 [−0.29; 1.39]	5.2%
Boily−Larouche−2019	0.57	0.3577	0.57 [−0.13; 1.27]	6.8%
Fidel−2003	1.19	0.5796	1.19 [0.05; 2.32]	3.1%
Yegorov−2019	1.22	1.4055	1.22 [−1.53; 3.98]	0.6%
**Random effects model**			**0.05 [−0.17; 0.26]**	**100.0%**
Heterogeneity: I^2 = 15%				

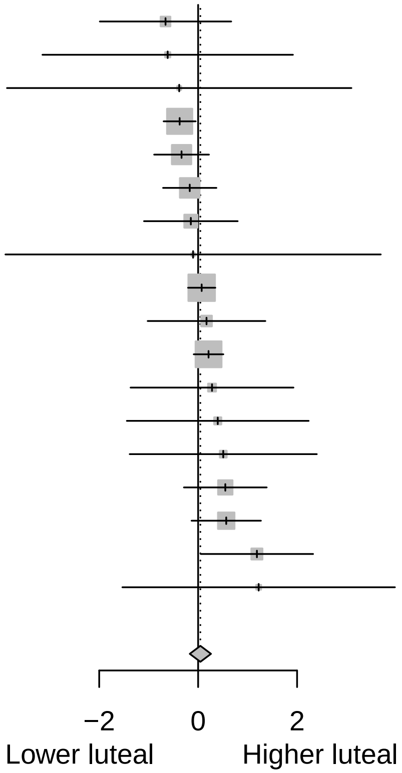

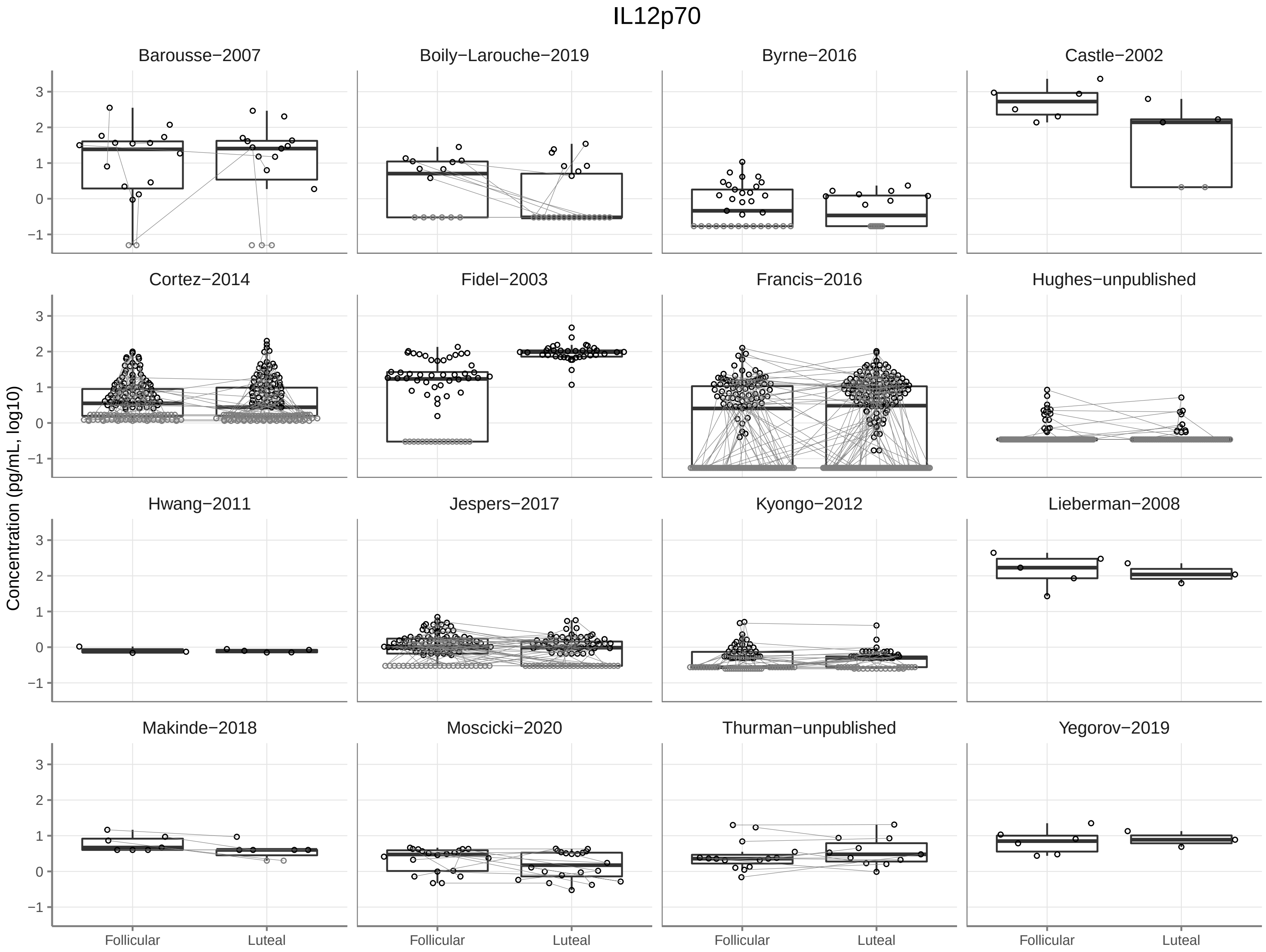
IL12p70
Barousse−2007
Boily−Larouche−2019
Byrne−2016
Castle−2002
Cortez−2014
Fidel−2003
Francis−2016
Hughes−unpublished
Hwang−2011
Jespers−2017
Kyongo−2012
Lieberman−2008
Makinde−2018
Moscicki−2020
Thurman−unpublished
Yegorov−2019
Concentration (pg/mL, log10)
3
2
1
0
−1
Follicular
Luteal

IL12p70	TE	seTE	95%−CI	Weight
Castle−2002	−3.79	1.7023	−3.79 [−7.13; −0.46]	1.9%
Boily−Larouche−2019	−1.28	0.8796	−1.28 [−3.00; 0.44]	4.4%
Makinde−2018	−0.71	0.2844	−0.71 [−1.27; −0.15]	7.5%
Moscicki−2020	−0.46	0.3143	−0.46 [−1.08; 0.16]	7.4%
Byrne−2016	−0.43	0.5503	−0.43 [−1.51; 0.65]	6.1%
Jespers−2017	−0.31	0.1436	−0.31 [−0.59; −0.02]	8.0%
Lieberman−2008	−0.27	1.0309	−0.27 [−2.29; 1.75]	3.7%
Hughes−unpublished	−0.16	0.1328	−0.16 [−0.42; 0.10]	8.1%
Kyongo−2012	−0.09	0.1387	−0.09 [−0.36; 0.18]	8.0%
Hwang−2011	−0.05	0.1555	−0.05 [−0.36; 0.25]	8.0%
Cortez−2014	−0.00	0.1657	−0.00 [−0.33; 0.32]	8.0%
Thurman−unpublished	0.20	0.2949	0.20 [−0.38; 0.78]	7.5%
Yegorov−2019	0.23	0.7402	0.23 [−1.22; 1.68]	5.1%
Francis−2016	0.30	0.3043	0.30 [−0.30; 0.90]	7.4%
Barousse−2007	0.40	1.3957	0.40 [−2.34; 3.13]	2.6%
Fidel−2003	3.69	0.5308	3.69 [2.65; 4.73]	6.2%
**Random effects model**			**−0.01 [−0.54; 0.52]**	**100.0%**

Heterogeneity: I^2 = 78%

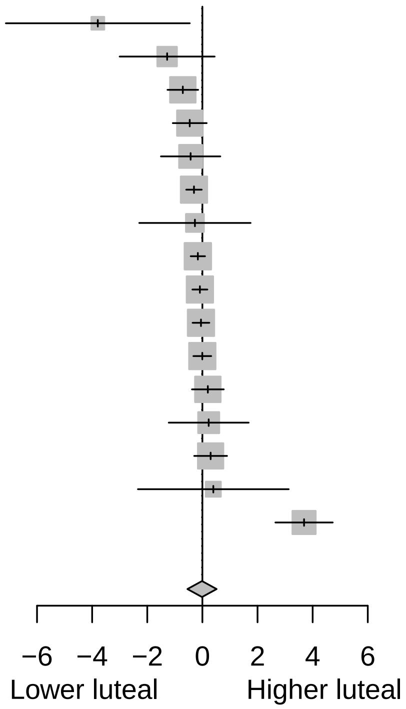

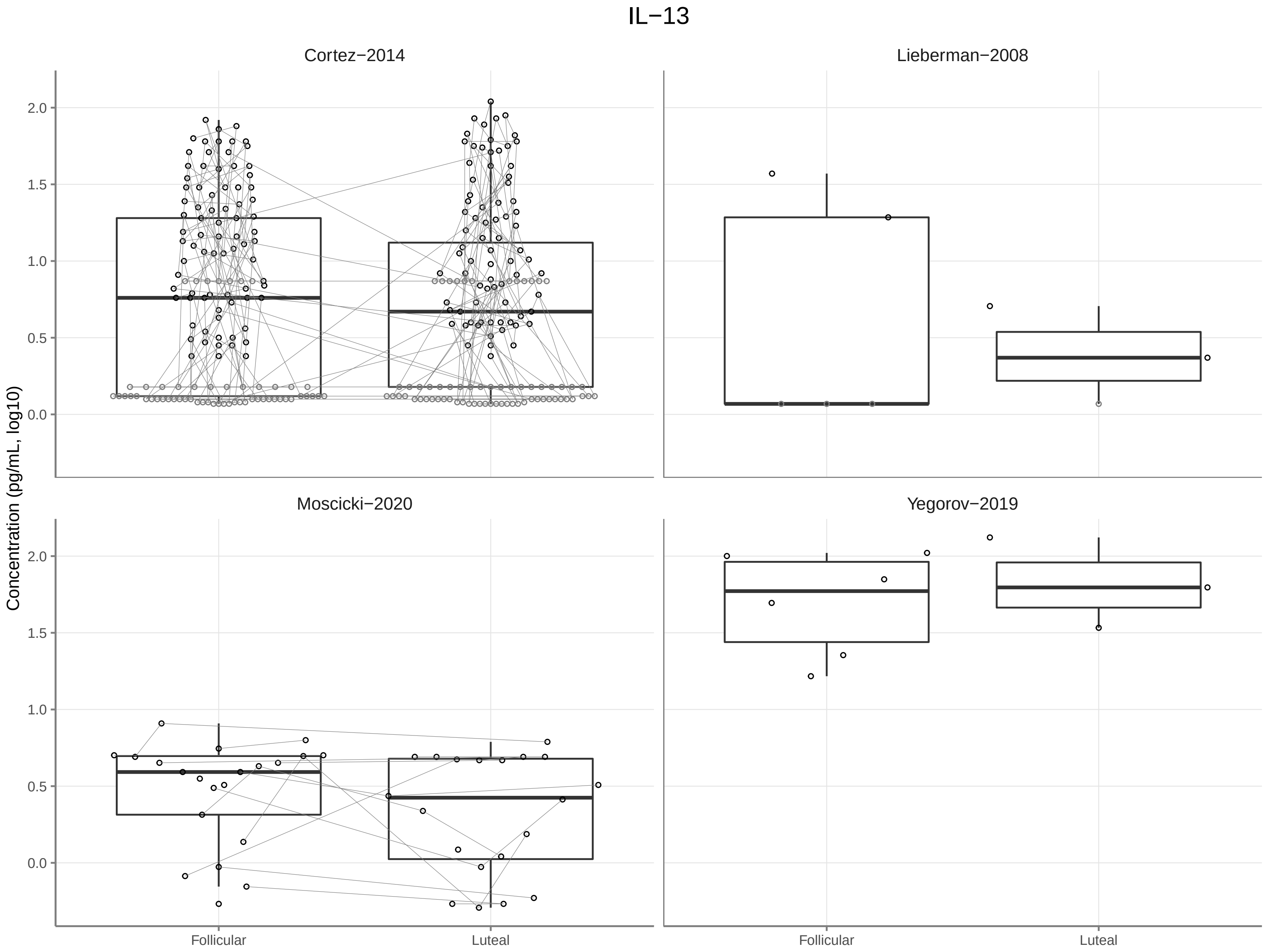
IL−13
Cortez−2014
Lieberman−2008
Moscicki−2020
Yegorov−2019
Concentration (pg/mL, log10)
2.0
1.5
1.0
0.5
0.0
Follicular
Luteal

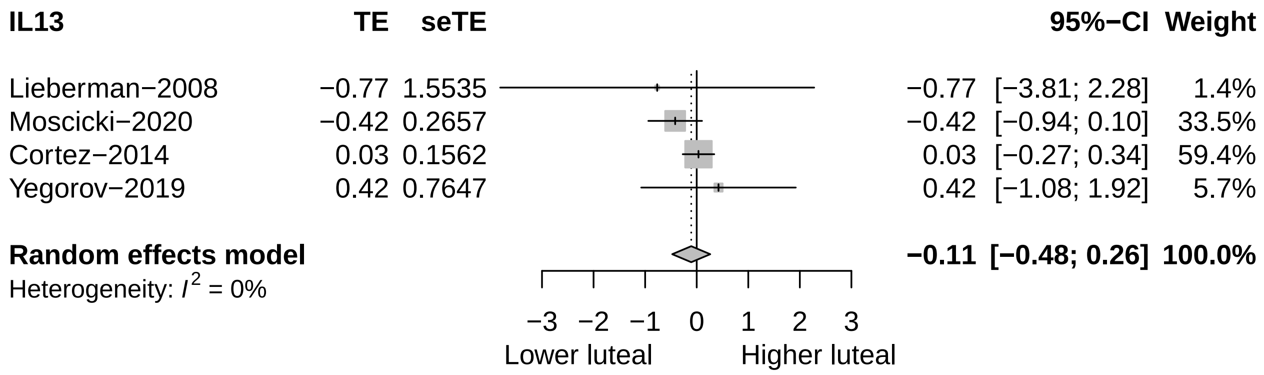
IL13
TE
seTE
95%−CI
Weight
Lieberman−2008 −0.77 1.5535 −0.77 [−3.81; 2.28] 1.4%
Moscicki−2020 −0.42 0.2657 −0.42 [−0.94; 0.10] 33.5%
Cortez−2014 0.03 0.1562 0.03 [−0.27; 0.34] 59.4%
Yegorov−2019 0.42 0.7647 0.42 [−1.08; 1.92] 5.7%
Random effects model −0.11 [−0.48; 0.26] 100.0%
Heterogeneity: I2 = 0%
−3 −2 −1 0 1 2 3
Lower luteal
Higher luteal

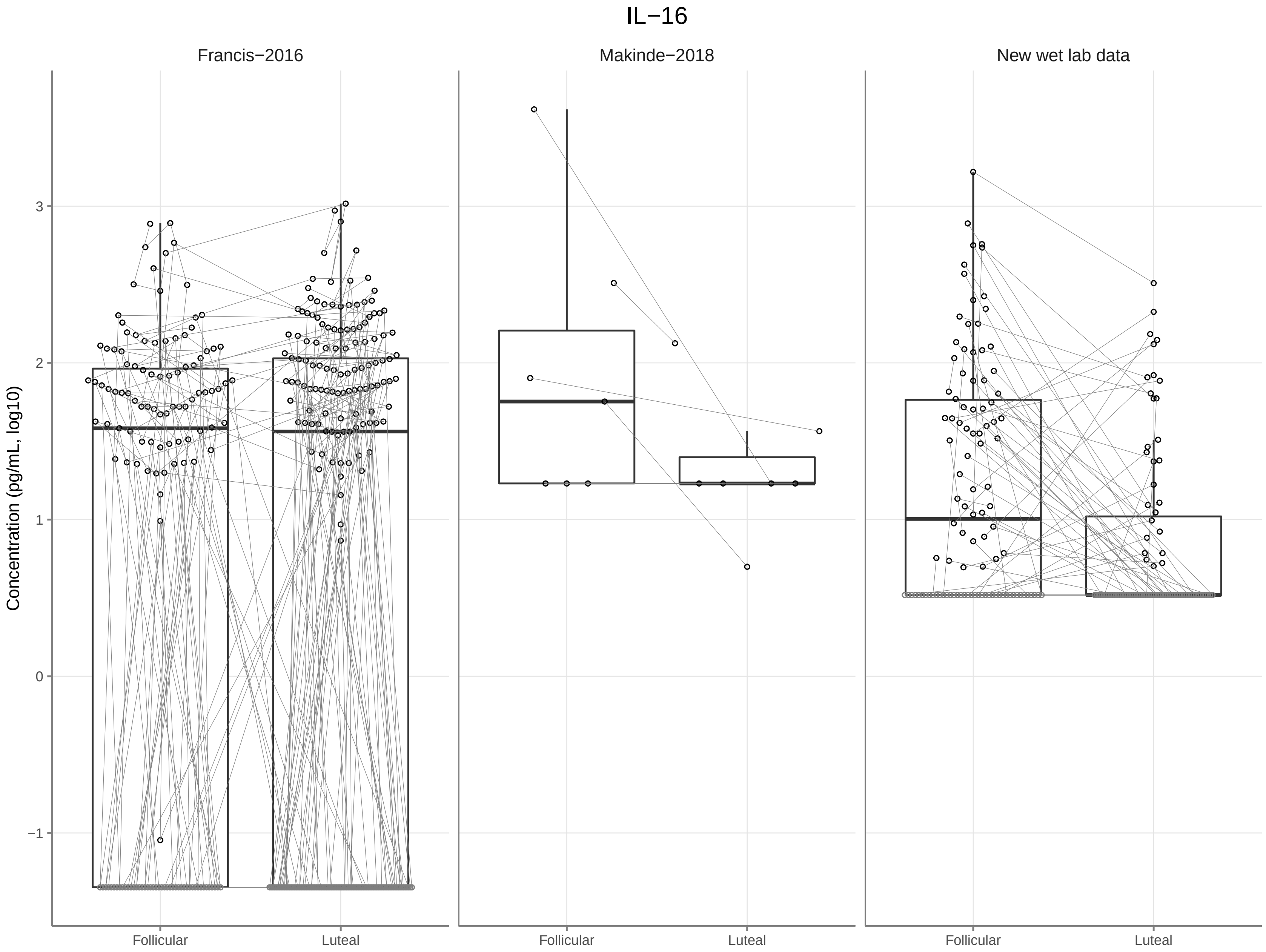

IL−16
Francis−2016
Makinde−2018
New wet lab data
Concentration (pg/mL, log10)
3
2
1
0
−1
Follicular
Luteal
Follicular
Luteal
Follicular
Luteal

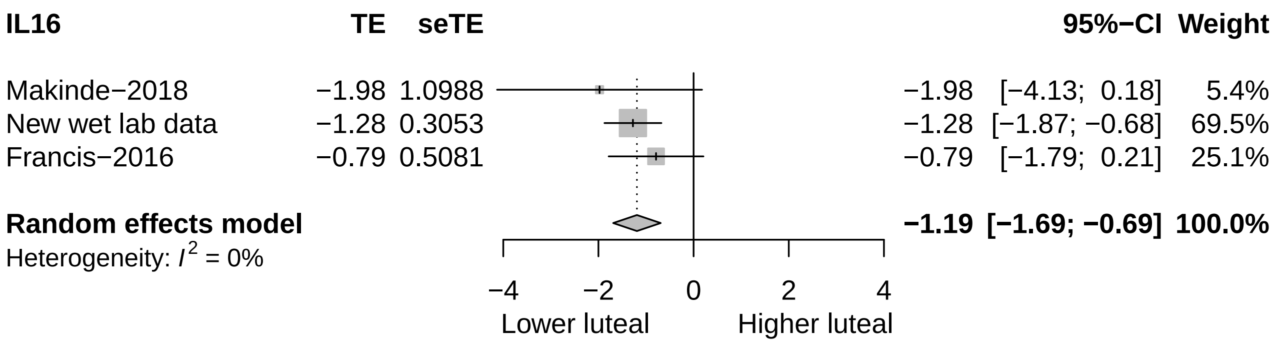
IL16
TE
seTE
95%−CI
Weight
Makinde−2018 −1.98 1.0988 −1.98 [−4.13; 0.18] 5.4%
New wet lab data −1.28 0.3053 −1.28 [−1.87; −0.68] 69.5%
Francis−2016 −0.79 0.5081 −0.79 [−1.79; 0.21] 25.1%
Random effects model −1.19 [−1.69; −0.69] 100.0%
Heterogeneity: I² = 0%
−4
−2
0
2
4
Lower luteal
Higher luteal

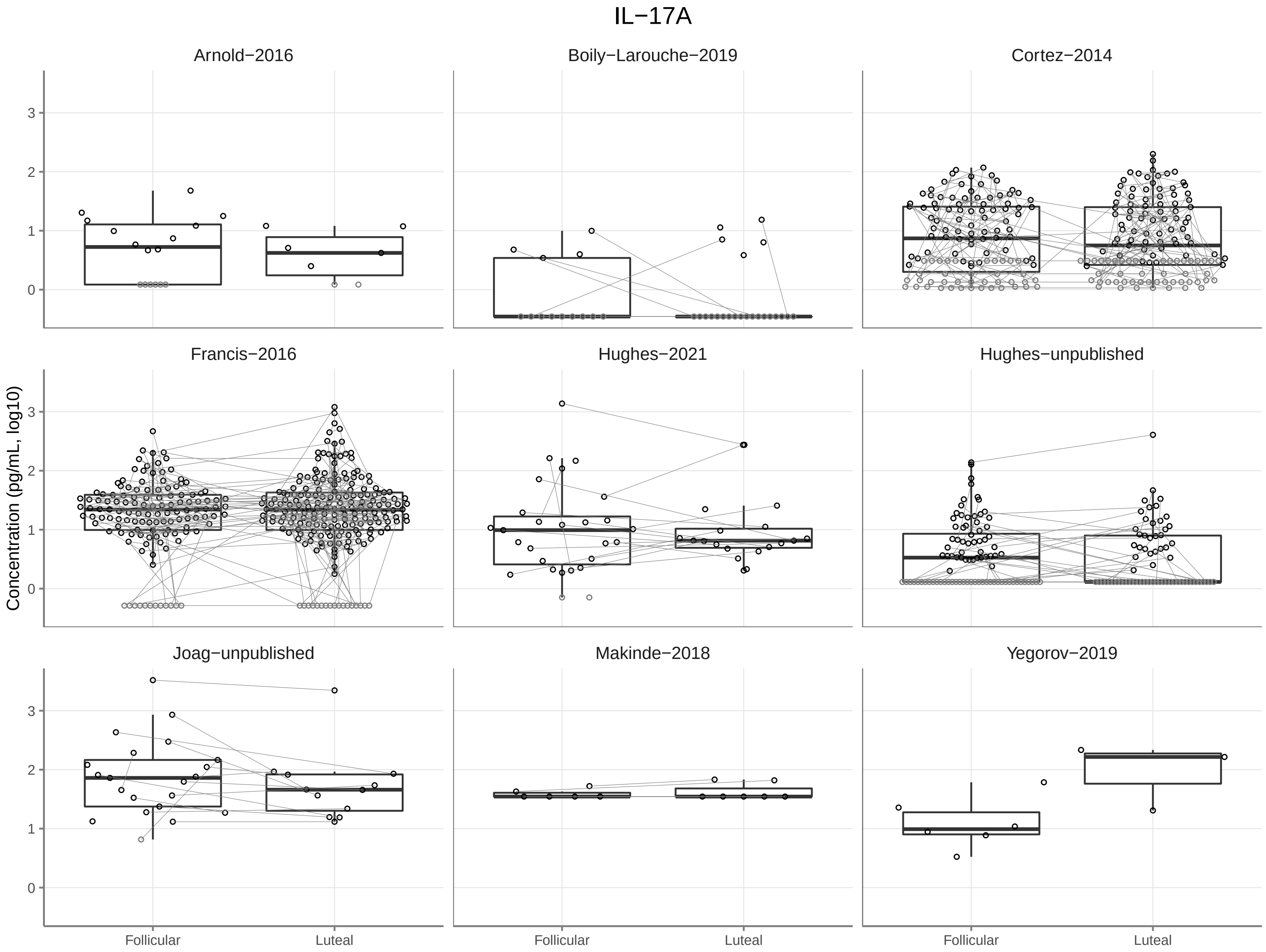

IL−17A
Arnold−2016
Boily−Larouche−2019
Cortez−2014
Francis−2016
Hughes−2021
Hughes−unpublished
Joag−unpublished
Makinde−2018
Yegorov−2019
Concentration (pg/mL, log10)
0
1
2
3
Follicular
Luteal

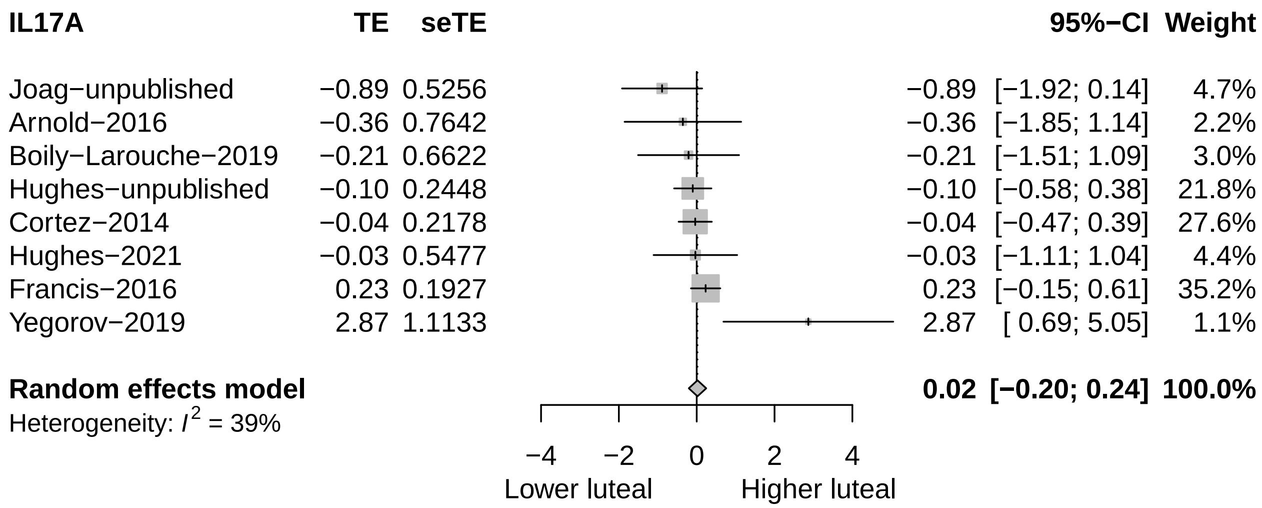
IL17A
TE
seTE
95%−CI
Weight
Joag−unpublished −0.89 0.5256 −0.89 [−1.92; 0.14] 4.7%
Arnold−2016 −0.36 0.7642 −0.36 [−1.85; 1.14] 2.2%
Boily−Larouche−2019 −0.21 0.6622 −0.21 [−1.51; 1.09] 3.0%
Hughes−unpublished −0.10 0.2448 −0.10 [−0.58; 0.38] 21.8%
Cortez−2014 −0.04 0.2178 −0.04 [−0.47; 0.39] 27.6%
Hughes−2021 −0.03 0.5477 −0.03 [−1.11; 1.04] 4.4%
Francis−2016 0.23 0.1927 0.23 [−0.15; 0.61] 35.2%
Yegorov−2019 2.87 1.1133 2.87 [0.69; 5.05] 1.1%
Random effects model 0.02 [−0.20; 0.24] 100.0%
Heterogeneity: I2 = 39%
−4
−2
0
2
4
Lower luteal
Higher luteal

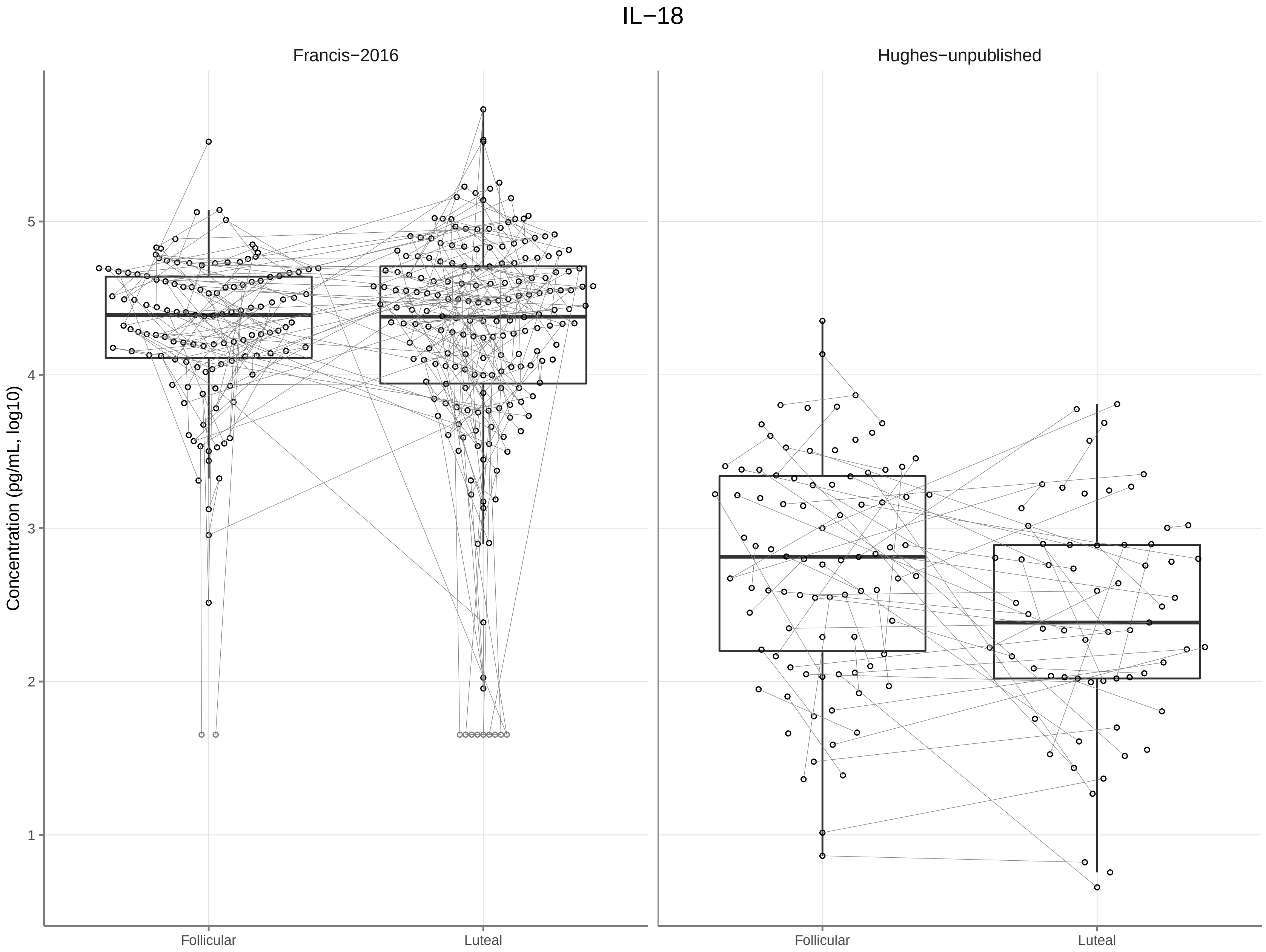
IL−18
Francis−2016
Hughes−unpublished
Concentration (pg/mL, log10)
1
2
3
4
5
Follicular
Luteal
Follicular
Luteal

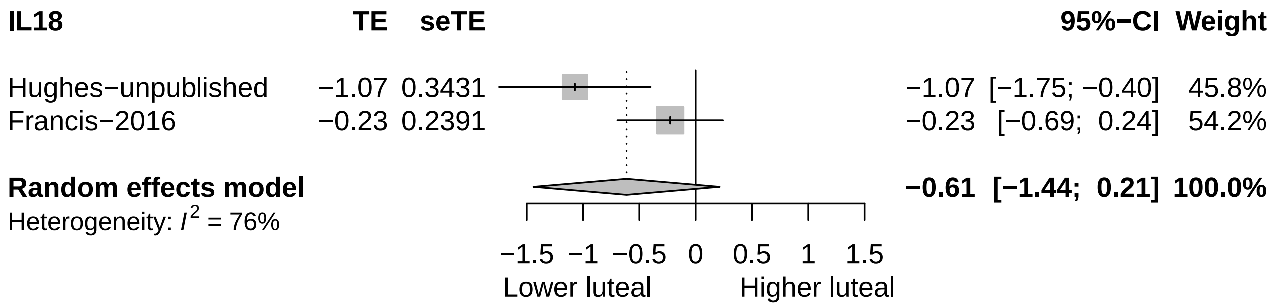
IL18
TE seTE
95%−CI Weight
Hughes−unpublished −1.07 0.3431 −1.07 [−1.75; −0.40] 45.8%
Francis−2016 −0.23 0.2391 −0.23 [−0.69; 0.24] 54.2%
Random effects model −0.61 [−1.44; 0.21] 100.0%
Heterogeneity: I^2 = 76%
−1.5 −1 −0.5 0 0.5 1 1.5
Lower luteal
Higher luteal

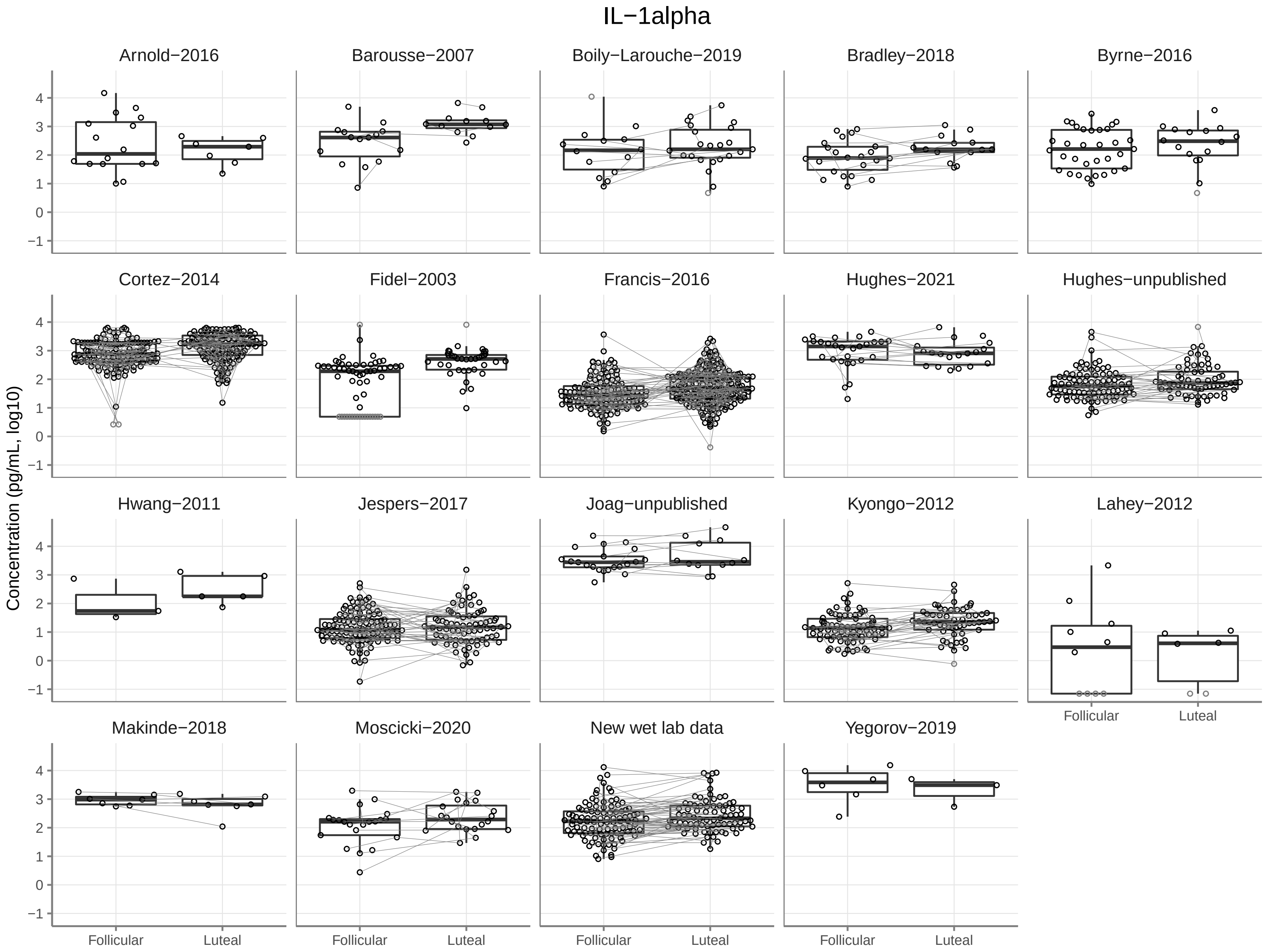
IL−1alpha
Arnold−2016
Barousse−2007
Boily−Larouche−2019
Bradley−2018
Byrne−2016
Cortez−2014
Fidel−2003
Francis−2016
Hughes−2021
Hughes−unpublished
Hwang−2011
Jespers−2017
Joag−unpublished
Kyongo−2012
Lahey−2012
Makinde−2018
Moscicki−2020
New wet lab data
Yegorov−2019
Concentration (pg/mL, log10)
Follicular
Luteal

IL1A	TE	seTE	95%−CI	Weight
Lahey−2012	−0.84	2.4102	−0.84 [−5.57; 3.88]	0.3%
Arnold−2016	−0.78	1.2891	−0.78 [−3.31; 1.74]	0.9%
Yegorov−2019	−0.58	1.4336	−0.58 [−3.39; 2.23]	0.7%
Makinde−2018	−0.56	0.4201	−0.56 [−1.38; 0.27]	5.0%
Hughes−2021	−0.14	0.5118	−0.14 [−1.15; 0.86]	3.9%
Shust−2010	0.07	0.2350	0.07 [−0.39; 0.53]	8.4%
Jespers−2017	0.15	0.2146	0.15 [−0.27; 0.57]	8.8%
Joag−unpublished	0.31	0.3478	0.31 [−0.37; 0.99]	6.2%
Hughes−unpublished	0.48	0.2325	0.48 [0.02; 0.93]	8.4%
Byrne−2016	0.48	0.7289	0.48 [−0.95; 1.91]	2.3%
Boily−Larouche−2019	0.55	0.7444	0.55 [−0.91; 2.01]	2.2%
Francis−2016	0.60	0.1643	0.60 [0.28; 0.92]	9.9%
New wet lab data	0.73	0.2097	0.73 [0.32; 1.14]	8.9%
Kyongo−2012	0.73	0.1791	0.73 [0.38; 1.09]	9.6%
Bradley−2018	0.82	0.3957	0.82 [0.05; 1.60]	5.4%
Cortez−2014	0.83	0.1832	0.83 [0.47; 1.19]	9.5%
Moscicki−2020	1.03	0.6014	1.03 [−0.15; 2.21]	3.1%
Hwang−2011	1.48	1.4535	1.48 [−1.37; 4.33]	0.7%
Barousse−2007	1.56	0.7995	1.56 [−0.01; 3.13]	2.0%
Fidel−2003	2.40	0.5276	2.40 [1.36; 3.43]	3.8%
**Random effects model**			**0.54 [0.29; 0.78]**	**100.0%**

Heterogeneity: $I^2 = 51\%$

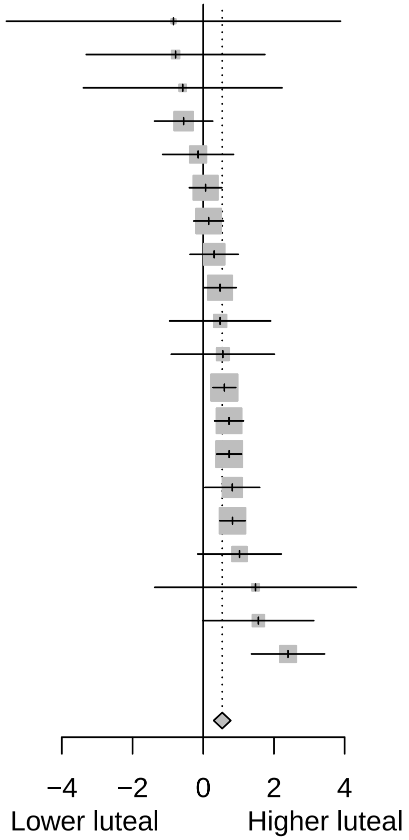

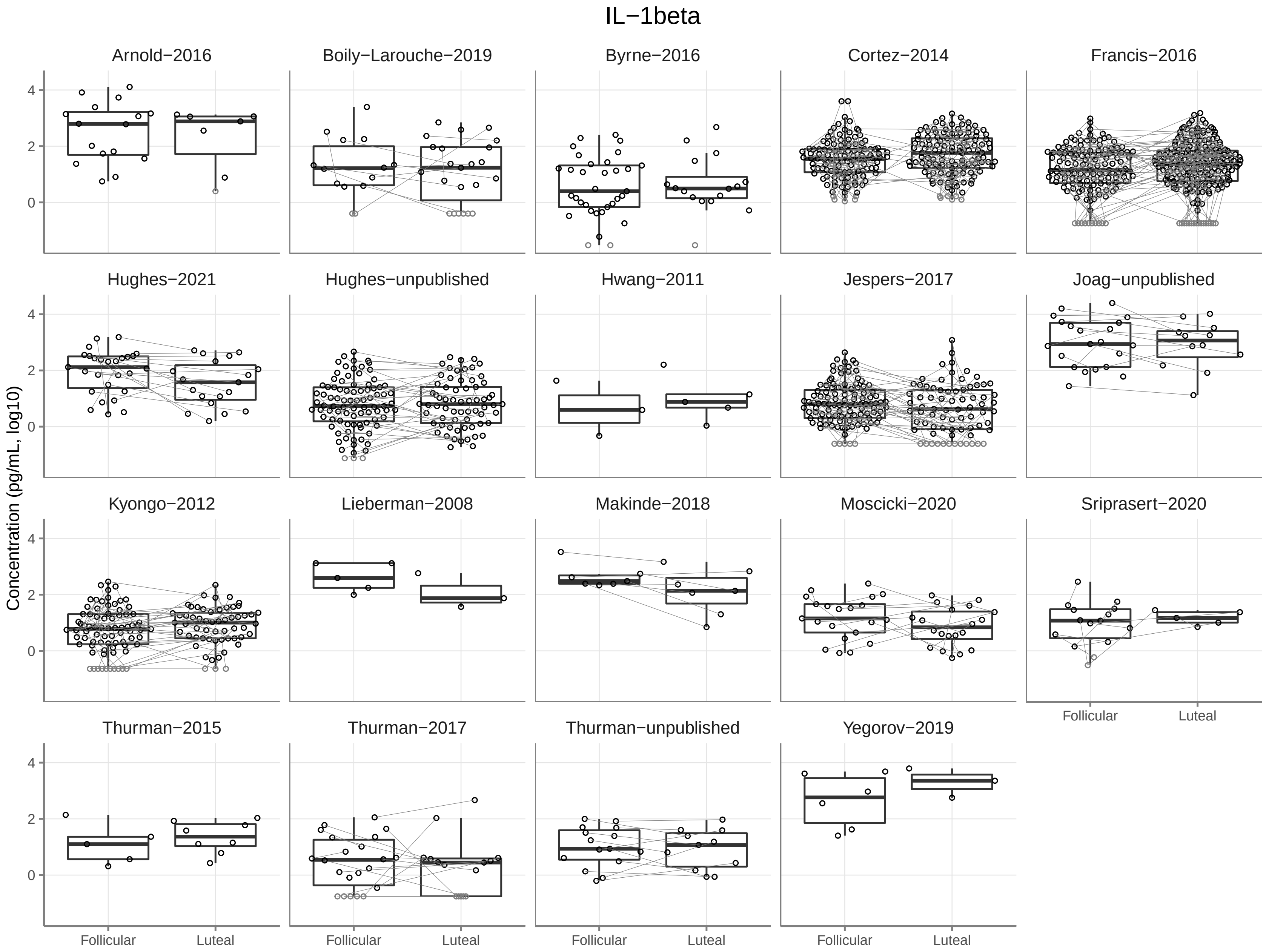

IL−1beta
Arnold−2016
Boily−Larouche−2019
Byrne−2016
Cortez−2014
Francis−2016
Hughes−2021
Hughes−unpublished
Hwang−2011
Jespers−2017
Joag−unpublished
Kyongo−2012
Lieberman−2008
Makinde−2018
Moscicki−2020
Sriprasert−2020
Thurman−2015
Thurman−2017
Thurman−unpublished
Yegorov−2019
Concentration (pg/mL, log10)
4
2
0
Follicular
Luteal

IL1B	TE	seTE	95%−CI	Weight
Lieberman−2008	−1.82	1.3314	−1.82 [−4.43; 0.79]	1.7%
Makinde−2018	−1.79	0.7942	−1.79 [−3.35; −0.23]	4.0%
Hughes−2021	−1.42	0.7888	−1.42 [−2.97; 0.13]	4.1%
Moscicki−2020	−1.08	0.7469	−1.08 [−2.54; 0.38]	4.4%
Joag−unpublished	−0.93	0.5715	−0.93 [−2.05; 0.19]	6.2%
Arnold−2016	−0.78	1.6471	−0.78 [−4.01; 2.45]	1.2%
Shust−2010	−0.68	0.6786	−0.68 [−2.01; 0.65]	5.0%
Jespers−2017	−0.58	0.3015	−0.58 [−1.17; 0.01]	10.9%
Boily−Larouche−2019	−0.46	1.2346	−0.46 [−2.88; 1.96]	2.0%
Thurman−2017	−0.29	0.9369	−0.29 [−2.12; 1.55]	3.1%
Thurman−unpublished	−0.19	0.6310	−0.19 [−1.42; 1.05]	5.5%
Hughes−unpublished	0.17	0.4056	0.17 [−0.63; 0.96]	8.8%
Byrne−2016	0.29	1.0691	0.29 [−1.81; 2.38]	2.5%
Francis−2016	0.38	0.2618	0.38 [−0.14; 0.89]	11.7%
Kyongo−2012	0.42	0.2819	0.42 [−0.13; 0.98]	11.3%
Cortez−2014	0.51	0.2609	0.51 [−0.01; 1.02]	11.7%
Thurman−2015	0.83	1.1925	0.83 [−1.50; 3.17]	2.1%
Hwang−2011	1.18	2.0896	1.18 [−2.92; 5.27]	0.7%
Sriprasert−2020	1.34	1.1323	1.34 [−0.88; 3.56]	2.3%
Yegorov−2019	2.19	2.0360	2.19 [−1.80; 6.19]	0.8%
**Random effects model**			**−0.15 [−0.52; 0.21]**	**100.0%**

Heterogeneity: I^2 = 40%

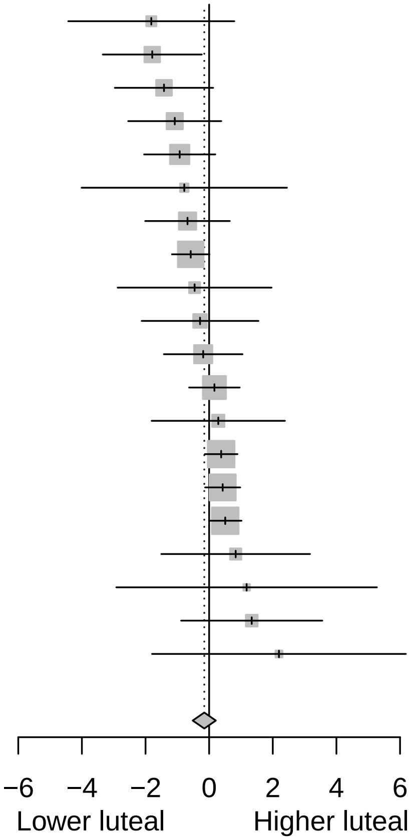

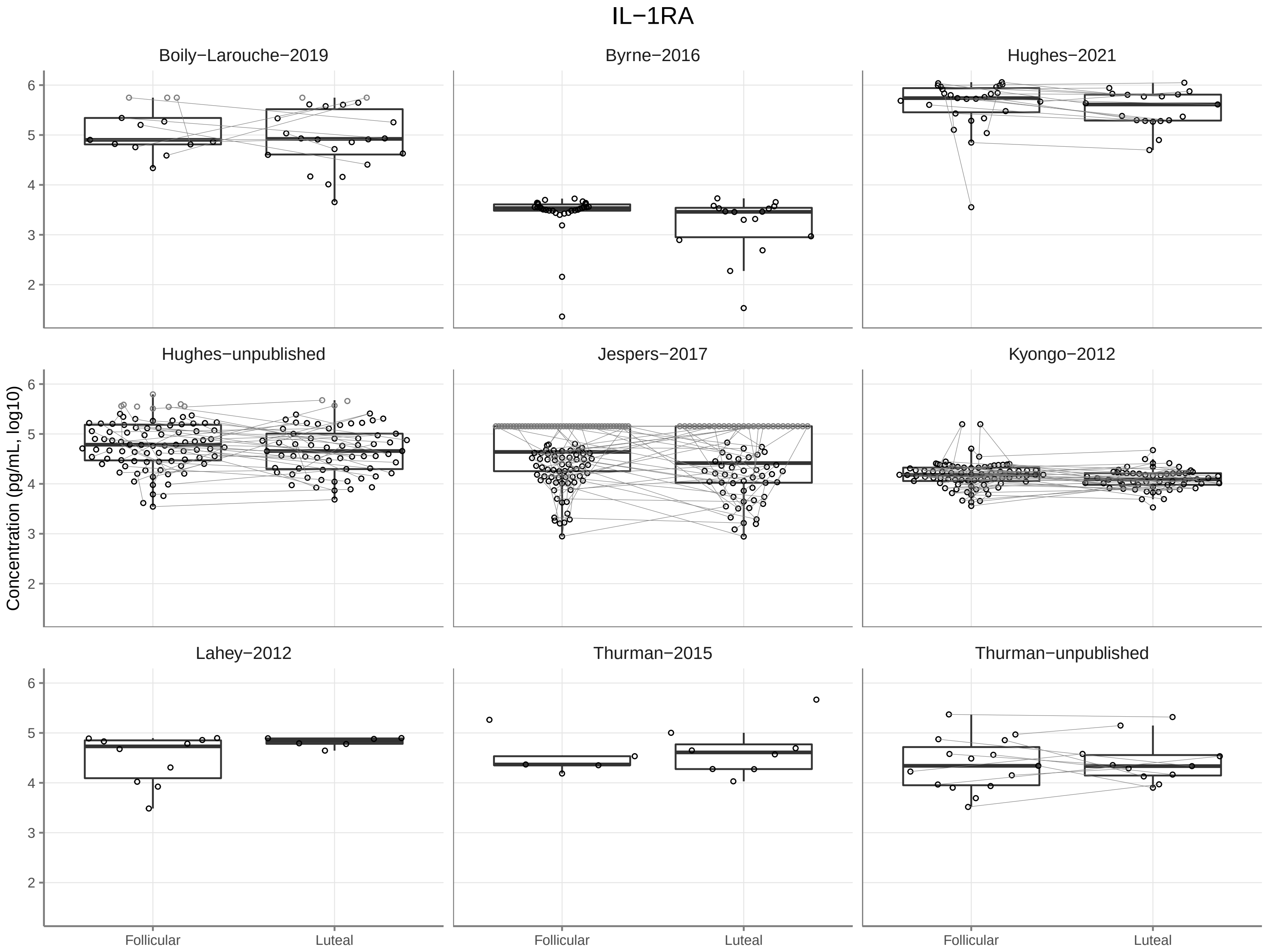
IL−1RA
Boily−Larouche−2019
Byrne−2016
Hughes−2021
Hughes−unpublished
Jespers−2017
Kyongo−2012
Lahey−2012
Thurman−2015
Thurman−unpublished
Concentration (pg/mL, log10)
Follicular
Luteal
2
3
4
5
6

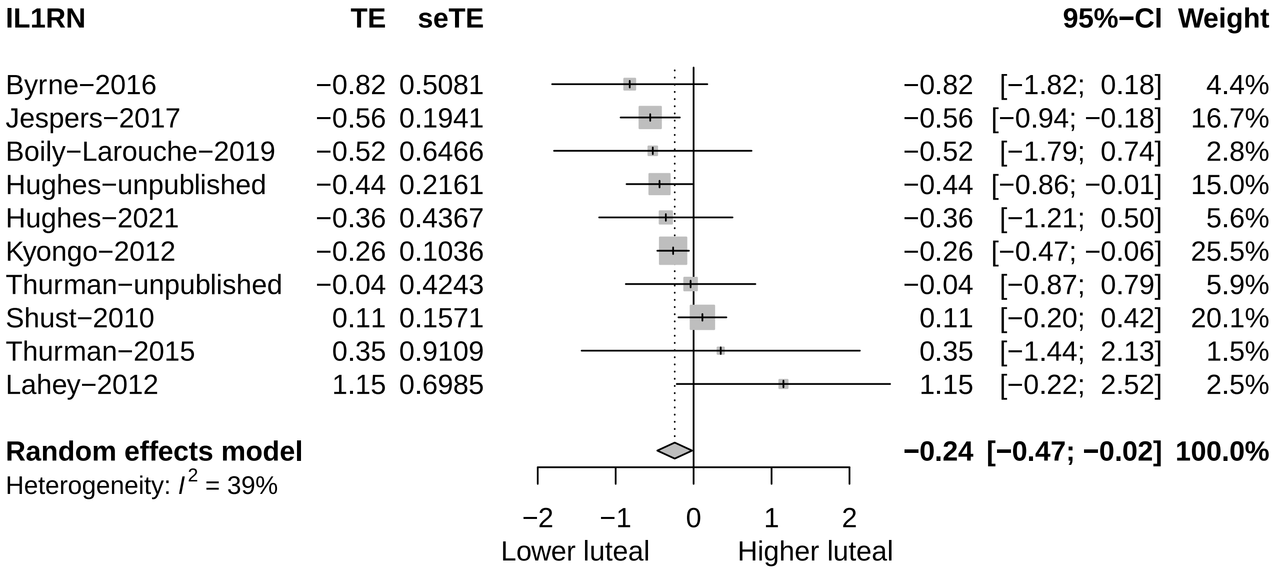
IL1RN
TE
seTE
95%−CI
Weight
Byrne−2016 −0.82 0.5081 −0.82 [−1.82; 0.18] 4.4%
Jespers−2017 −0.56 0.1941 −0.56 [−0.94; −0.18] 16.7%
Boily−Larouche−2019 −0.52 0.6466 −0.52 [−1.79; 0.74] 2.8%
Hughes−unpublished −0.44 0.2161 −0.44 [−0.86; −0.01] 15.0%
Hughes−2021 −0.36 0.4367 −0.36 [−1.21; 0.50] 5.6%
Kyongo−2012 −0.26 0.1036 −0.26 [−0.47; −0.06] 25.5%
Thurman−unpublished −0.04 0.4243 −0.04 [−0.87; 0.79] 5.9%
Shust−2010 0.11 0.1571 0.11 [−0.20; 0.42] 20.1%
Thurman−2015 0.35 0.9109 0.35 [−1.44; 2.13] 1.5%
Lahey−2012 1.15 0.6985 1.15 [−0.22; 2.52] 2.5%
Random effects model −0.24 [−0.47; −0.02] 100.0%
Heterogeneity: I2 = 39%
−2 −1 0 1 2
Lower luteal
Higher luteal

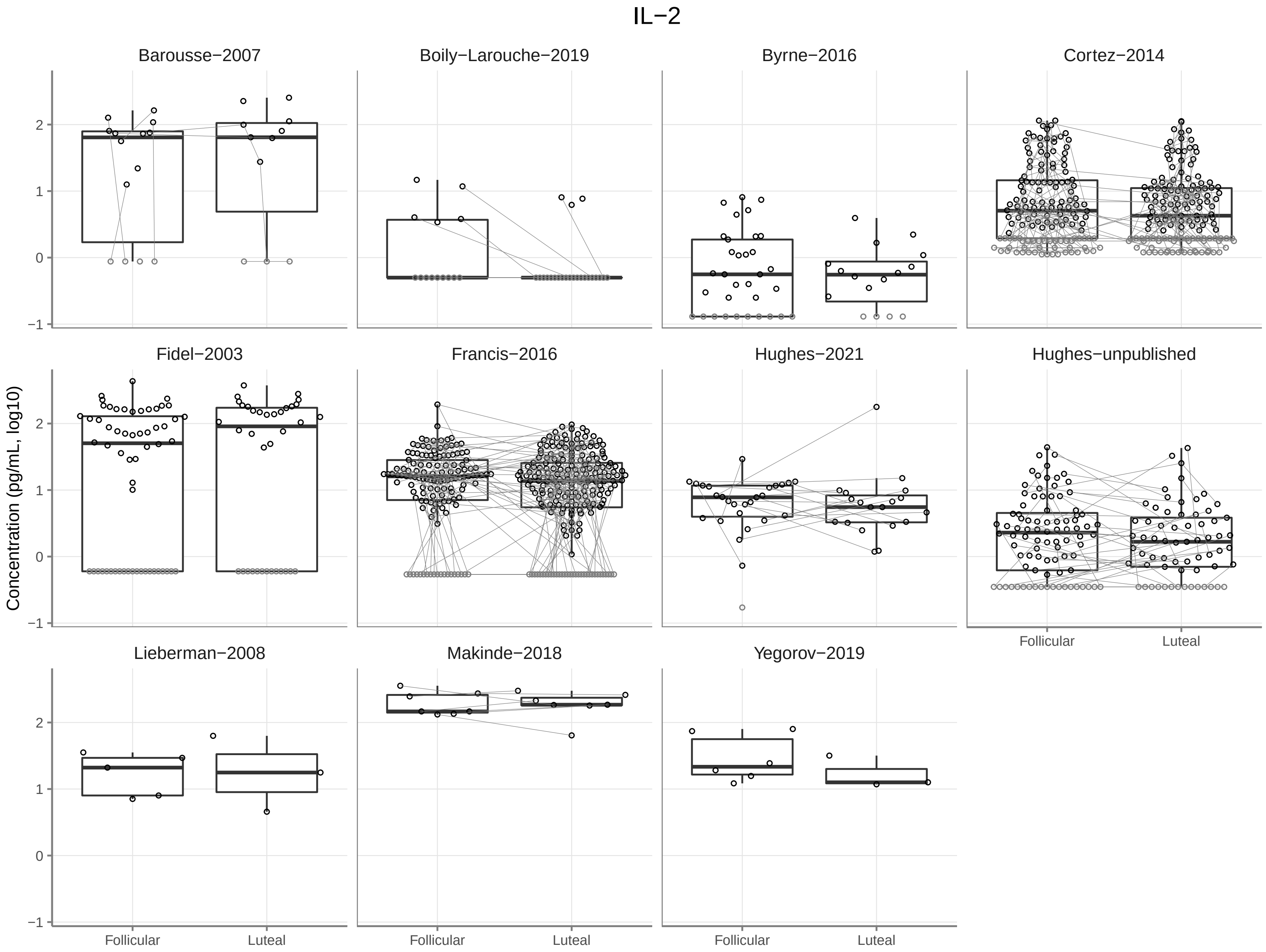
IL−2
Barousse−2007
Boily−Larouche−2019
Byrne−2016
Cortez−2014
Fidel−2003
Francis−2016
Hughes−2021
Hughes−unpublished
Lieberman−2008
Makinde−2018
Yegorov−2019
Concentration (pg/mL, log10)
−1
0
1
2
Follicular
Luteal

IL2	TE	seTE	95%−CI	Weight
Boily−Larouche−2019	−0.81	0.5271	−0.81 [−1.84; 0.22]	2.9%
Yegorov−2019	−0.76	0.7569	−0.76 [−2.25; 0.72]	1.4%
Hughes−unpublished	−0.25	0.2621	−0.25 [−0.76; 0.26]	11.6%
Byrne−2016	−0.23	0.5666	−0.23 [−1.34; 0.88]	2.5%
Francis−2016	−0.17	0.1770	−0.17 [−0.51; 0.18]	25.4%
Cortez−2014	−0.12	0.1464	−0.12 [−0.40; 0.17]	37.2%
Makinde−2018	−0.07	0.2509	−0.07 [−0.56; 0.42]	12.7%
Hughes−2021	−0.02	0.4531	−0.02 [−0.91; 0.87]	3.9%
Lieberman−2008	0.06	1.0236	0.06 [−1.95; 2.06]	0.8%
Fidel−2003	0.39	0.7921	0.39 [−1.16; 1.94]	1.3%
Barousse−2007	0.48	1.2725	0.48 [−2.01; 2.98]	0.5%
**Random effects model**			**−0.16 [−0.33; 0.02]**	**100.0%**

Heterogeneity: $I^2 = 0\%$

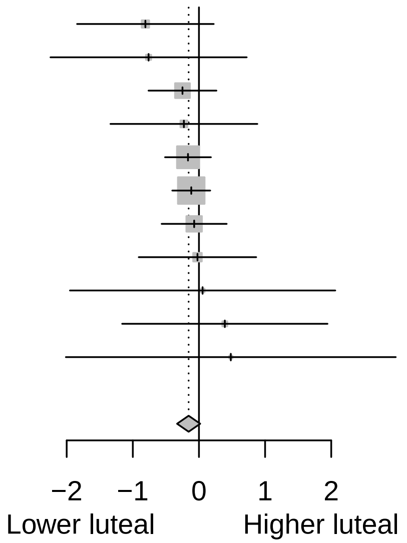

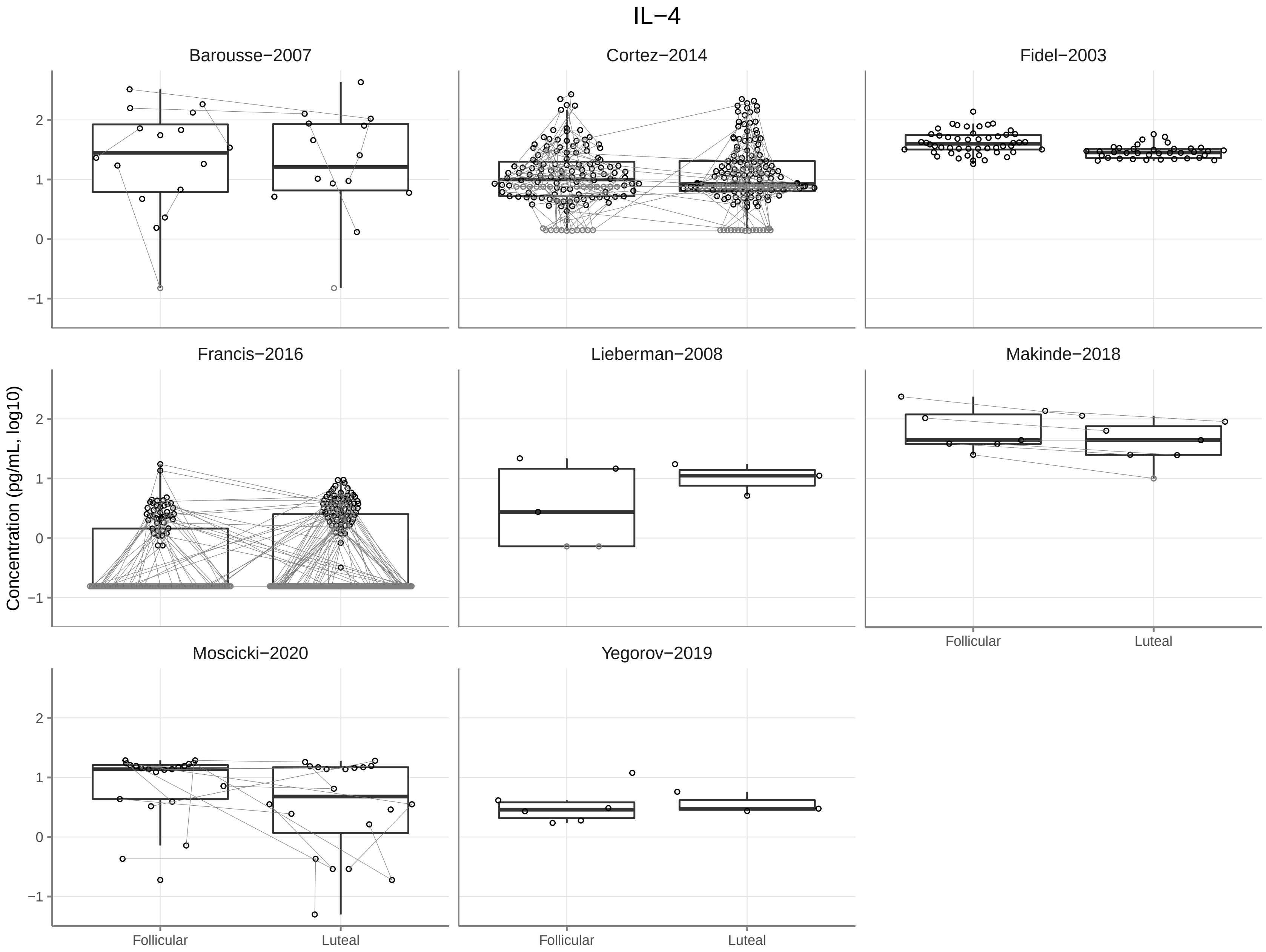
IL−4
Barousse−2007
Cortez−2014
Fidel−2003
Francis−2016
Lieberman−2008
Makinde−2018
Moscicki−2020
Yegorov−2019
Concentration (pg/mL, log10)
2
1
0
−1
Follicular
Luteal

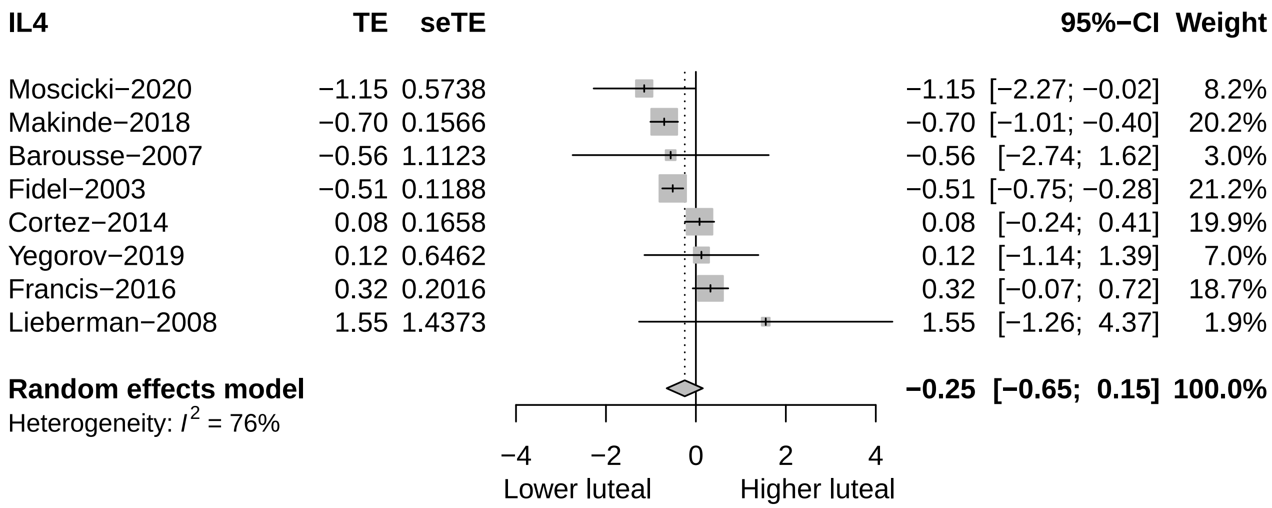
IL4
TE
seTE
95%−CI
Weight
Moscicki−2020 −1.15 0.5738 −1.15 [−2.27; −0.02] 8.2%
Makinde−2018 −0.70 0.1566 −0.70 [−1.01; −0.40] 20.2%
Barousse−2007 −0.56 1.1123 −0.56 [−2.74; 1.62] 3.0%
Fidel−2003 −0.51 0.1188 −0.51 [−0.75; −0.28] 21.2%
Cortez−2014 0.08 0.1658 0.08 [−0.24; 0.41] 19.9%
Yegorov−2019 0.12 0.6462 0.12 [−1.14; 1.39] 7.0%
Francis−2016 0.32 0.2016 0.32 [−0.07; 0.72] 18.7%
Lieberman−2008 1.55 1.4373 1.55 [−1.26; 4.37] 1.9%
Random effects model −0.25 [−0.65; 0.15] 100.0%
Heterogeneity: I2 = 76%
−4
−2
0
2
4
Lower luteal
Higher luteal

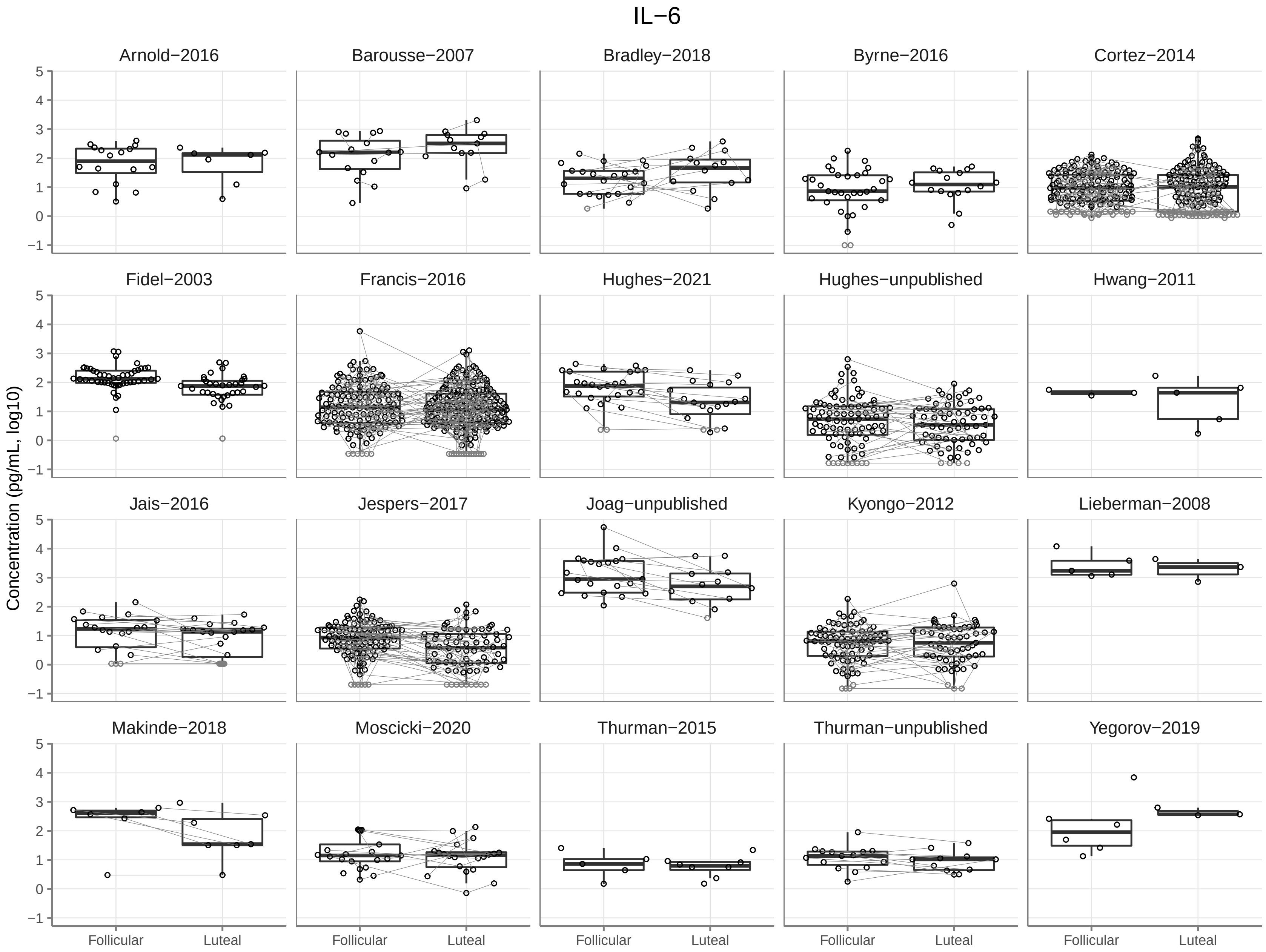

IL−6
Arnold−2016
Barousse−2007
Bradley−2018
Byrne−2016
Cortez−2014
Fidel−2003
Francis−2016
Hughes−2021
Hughes−unpublished
Hwang−2011
Jais−2016
Jespers−2017
Joag−unpublished
Kyongo−2012
Lieberman−2008
Makinde−2018
Moscicki−2020
Thurman−2015
Thurman−unpublished
Yegorov−2019
Concentration (pg/mL, log10)
Follicular
Luteal

IL6	TE	seTE	95%−CI	Weight
Makinde−2018	−2.01	0.6312	−2.01 [−3.25; −0.78]	3.9%
Joag−unpublished	−1.69	0.6450	−1.69 [−2.95; −0.42]	3.8%
Hughes−2021	−1.58	0.4312	−1.58 [−2.43; −0.74]	5.7%
Fidel−2003	−1.24	0.3760	−1.24 [−1.98; −0.51]	6.3%
Jespers−2017	−1.12	0.2404	−1.12 [−1.59; −0.65]	8.0%
Hwang−2011	−1.04	1.6326	−1.04 [−4.24; 2.16]	0.9%
Moscicki−2020	−0.68	0.5239	−0.68 [−1.71; 0.34]	4.7%
Jais−2016	−0.66	0.3596	−0.66 [−1.36; 0.05]	6.5%
Thurman−unpublished	−0.43	0.3879	−0.43 [−1.19; 0.33]	6.2%
Lieberman−2008	−0.42	1.0137	−0.42 [−2.40; 1.57]	2.0%
Hughes−unpublished	−0.39	0.3869	−0.39 [−1.15; 0.37]	6.2%
Thurman−2015	−0.20	0.7510	−0.20 [−1.67; 1.27]	3.1%
Francis−2016	−0.16	0.2313	−0.16 [−0.62; 0.29]	8.1%
Cortez−2014	−0.08	0.1623	−0.08 [−0.39; 0.24]	8.9%
Shust−2010	−0.05	0.3962	−0.05 [−0.82; 0.73]	6.1%
Kyongo−2012	−0.04	0.2774	−0.04 [−0.59; 0.50]	7.5%
Arnold−2016	−0.03	1.0072	−0.03 [−2.01; 1.94]	2.0%
Byrne−2016	0.55	0.7274	0.55 [−0.87; 1.98]	3.2%
Barousse−2007	0.78	0.8532	0.78 [−0.89; 2.45]	2.6%
Bradley−2018	0.91	0.6292	0.91 [−0.32; 2.14]	3.9%
Yegorov−2019	1.72	1.9377	1.72 [−2.08; 5.52]	0.6%
**Random effects model**			**−0.49 [−0.80; −0.18]**	**100.0%**

Heterogeneity: I^2 = 60%

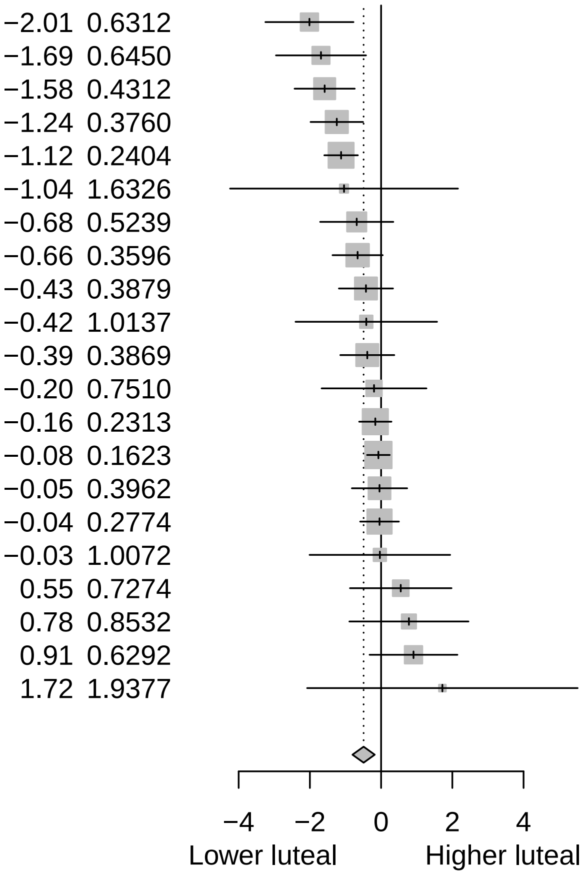

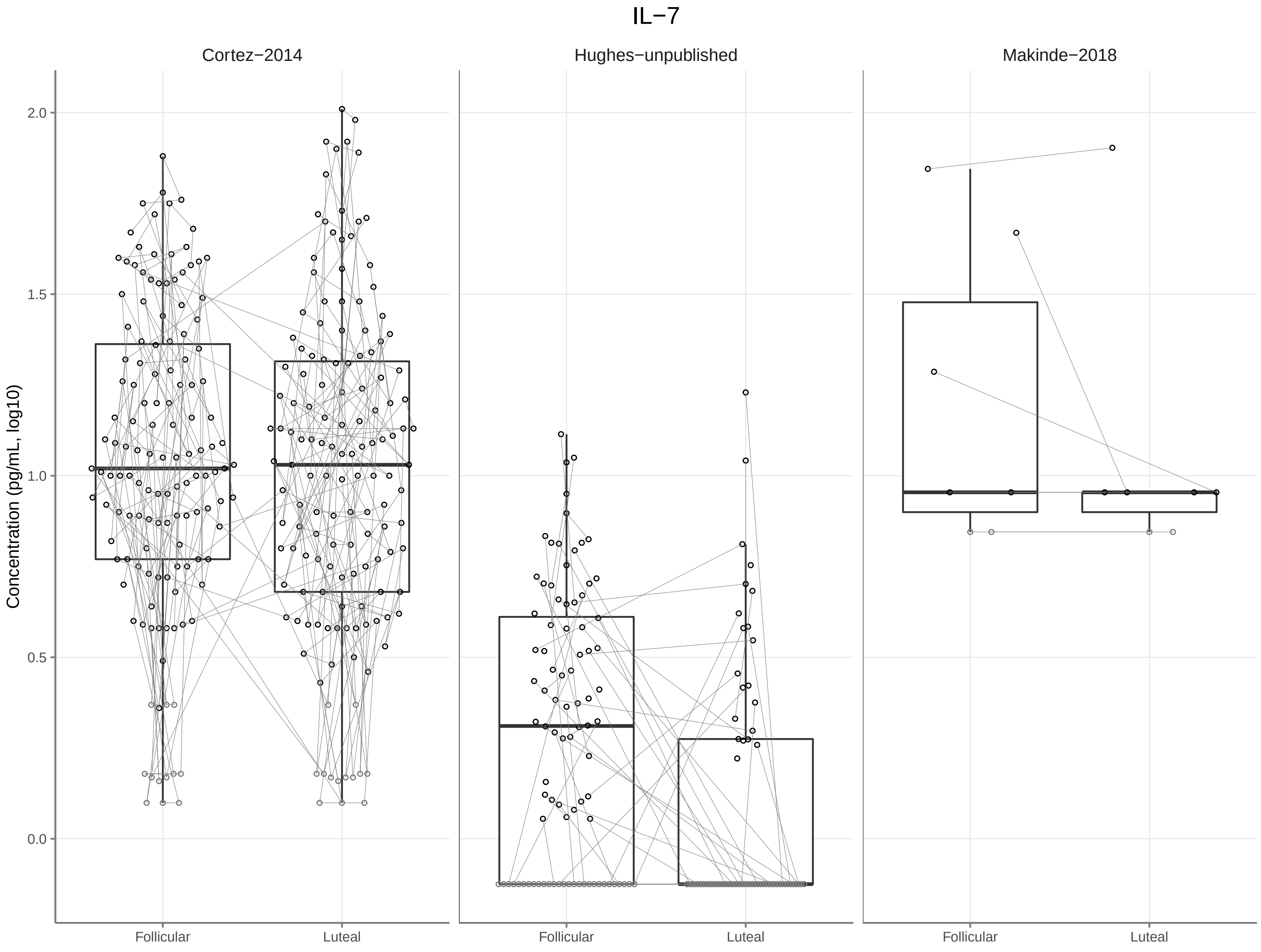

IL−7
Cortez−2014
Hughes−unpublished
Makinde−2018
Concentration (pg/mL, log10)
2.0
1.5
1.0
0.5
0.0
Follicular
Luteal
Follicular
Luteal
Follicular
Luteal

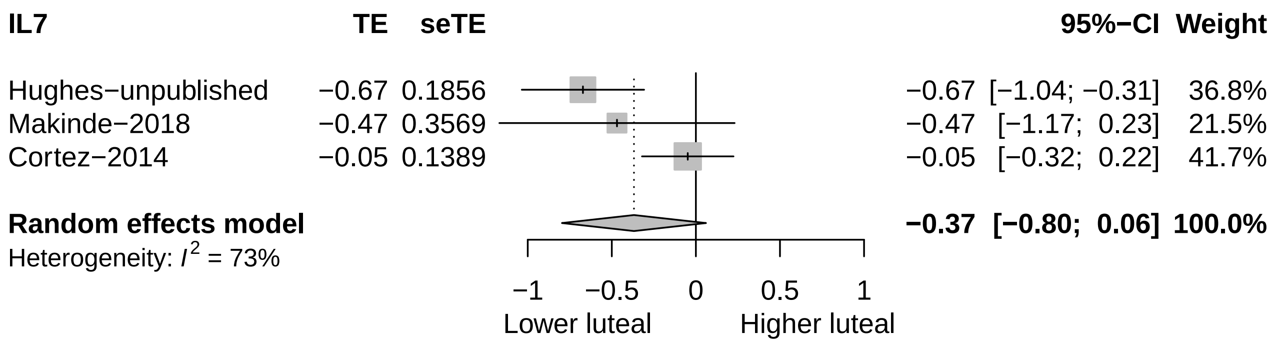
IL7
TE seTE
95%−CI Weight
Hughes−unpublished −0.67 0.1856 −0.67 [−1.04; −0.31] 36.8%
Makinde−2018 −0.47 0.3569 −0.47 [−1.17; 0.23] 21.5%
Cortez−2014 −0.05 0.1389 −0.05 [−0.32; 0.22] 41.7%
Random effects model −0.37 [−0.80; 0.06] 100.0%
Heterogeneity: I2 = 73%
−1 −0.5 0 0.5 1
Lower luteal
Higher luteal

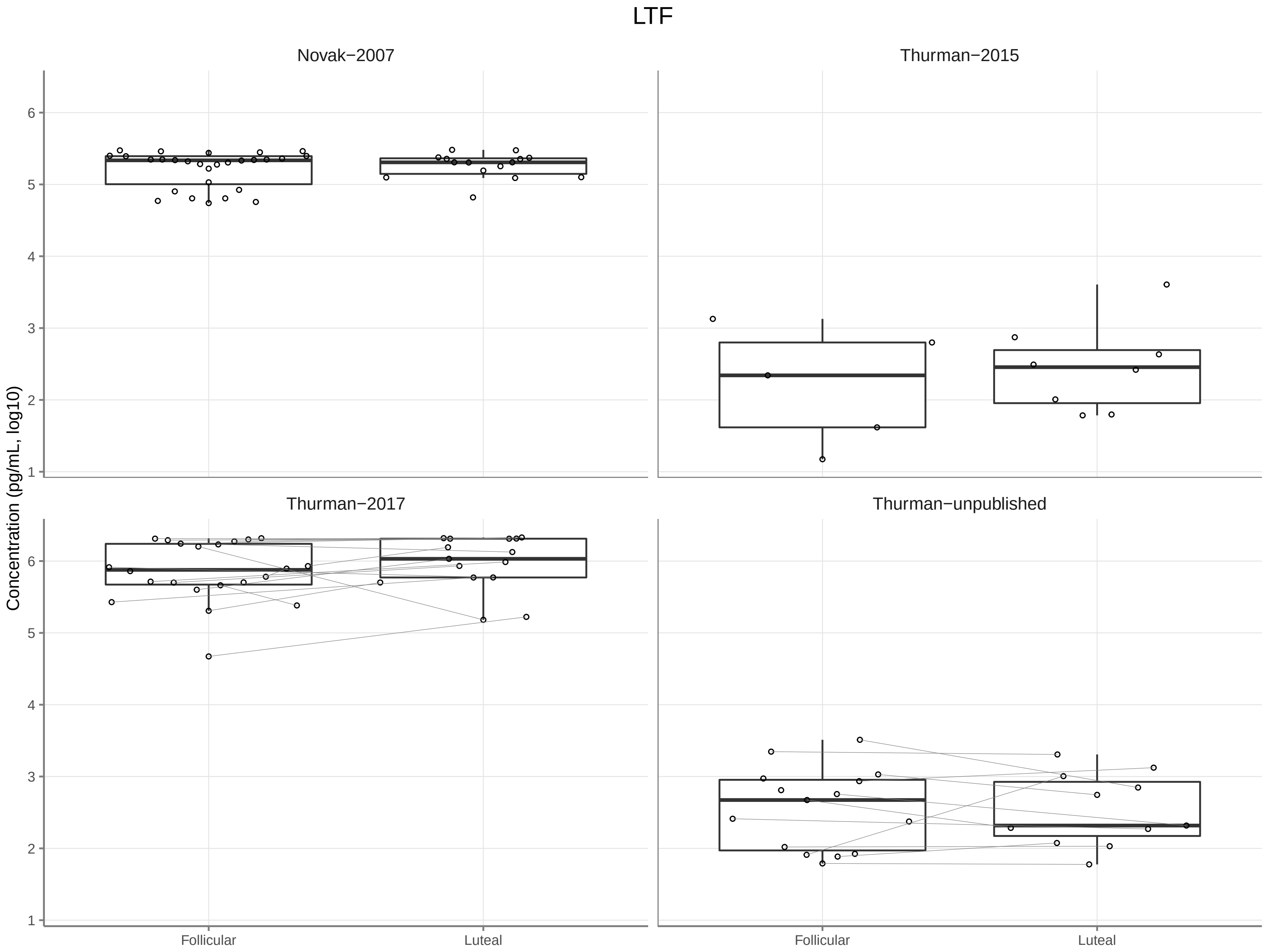

LTF
Novak−2007
Thurman−2015
Thurman−2017
Thurman−unpublished
Concentration (pg/mL, log10)
1
2
3
4
5
6
Follicular
Luteal

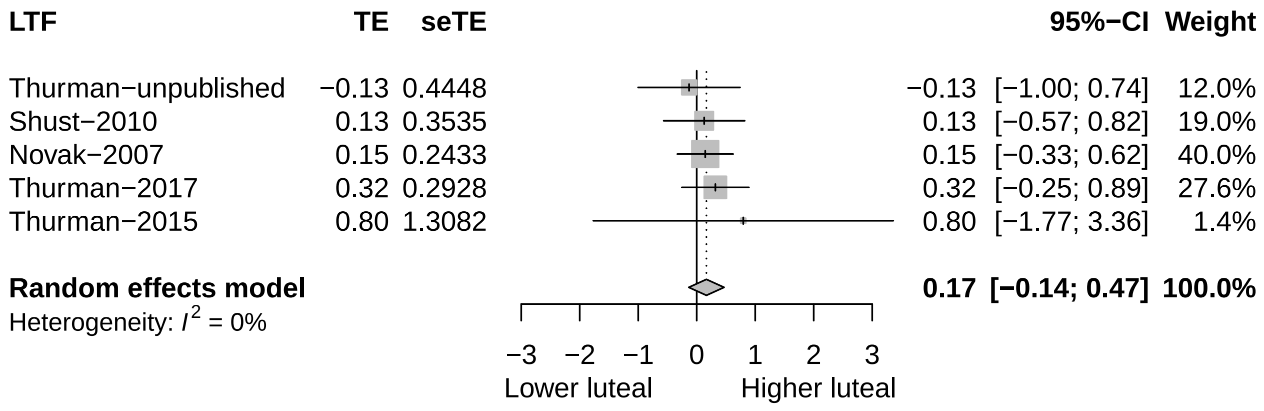
LTF TE seTE 95%−CI Weight
Thurman−unpublished −0.13 0.4448 −0.13 [−1.00; 0.74] 12.0%
Shust−2010 0.13 0.3535 0.13 [−0.57; 0.82] 19.0%
Novak−2007 0.15 0.2433 0.15 [−0.33; 0.62] 40.0%
Thurman−2017 0.32 0.2928 0.32 [−0.25; 0.89] 27.6%
Thurman−2015 0.80 1.3082 0.80 [−1.77; 3.36] 1.4%
Random effects model 0.17 [−0.14; 0.47] 100.0%
Heterogeneity: I^2 = 0%
−3 −2 −1 0 1 2 3
Lower luteal
Higher luteal

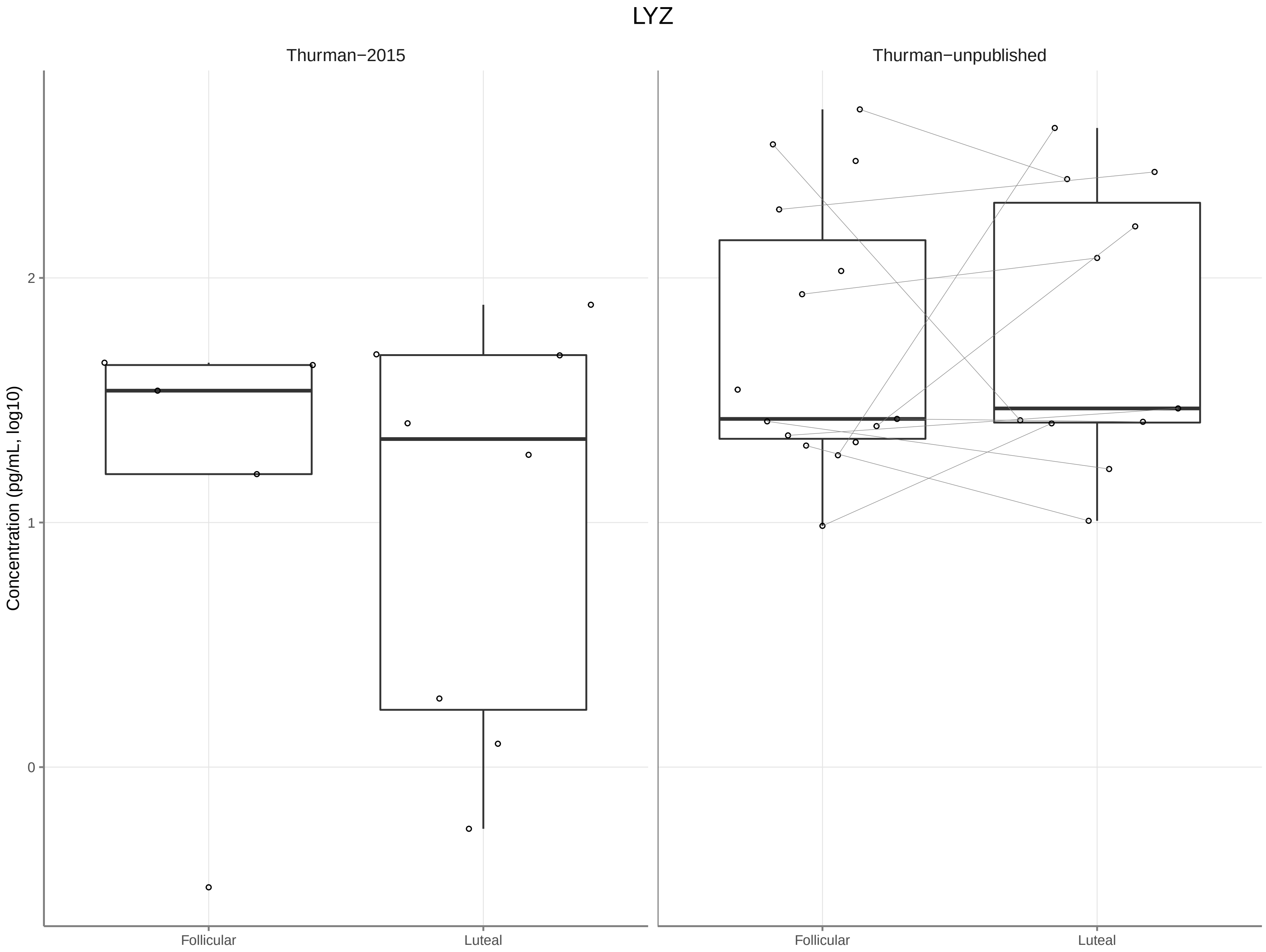
LYZ
Thurman−2015
Thurman−unpublished
Concentration (pg/mL, log10)
2
1
0
Follicular
Luteal
Follicular
Luteal

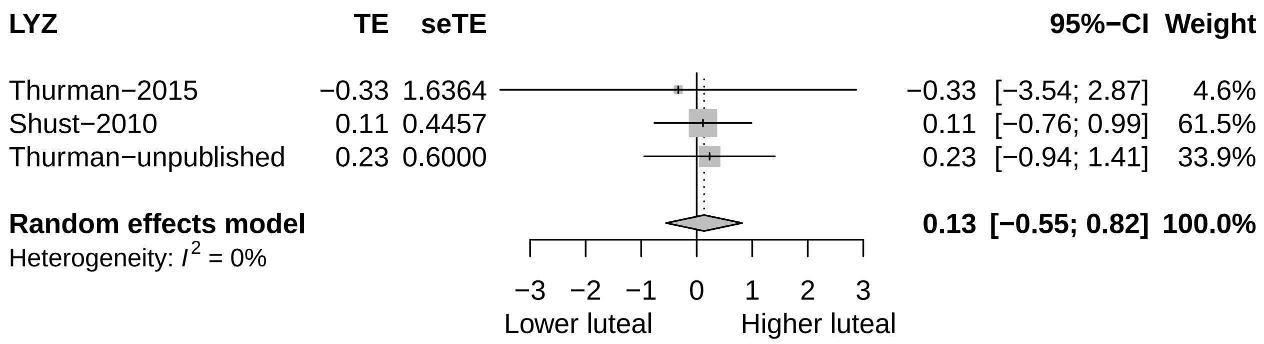
LYZ
TE
seTE
95%−CI
Weight
Thurman−2015 −0.33 1.6364 −0.33 [−3.54; 2.87] 4.6%
Shust−2010 0.11 0.4457 0.11 [−0.76; 0.99] 61.5%
Thurman−unpublished 0.23 0.6000 0.23 [−0.94; 1.41] 33.9%
Random effects model 0.13 [−0.55; 0.82] 100.0%
Heterogeneity: I^2 = 0%
−3 −2 −1 0 1 2 3
Lower luteal
Higher luteal

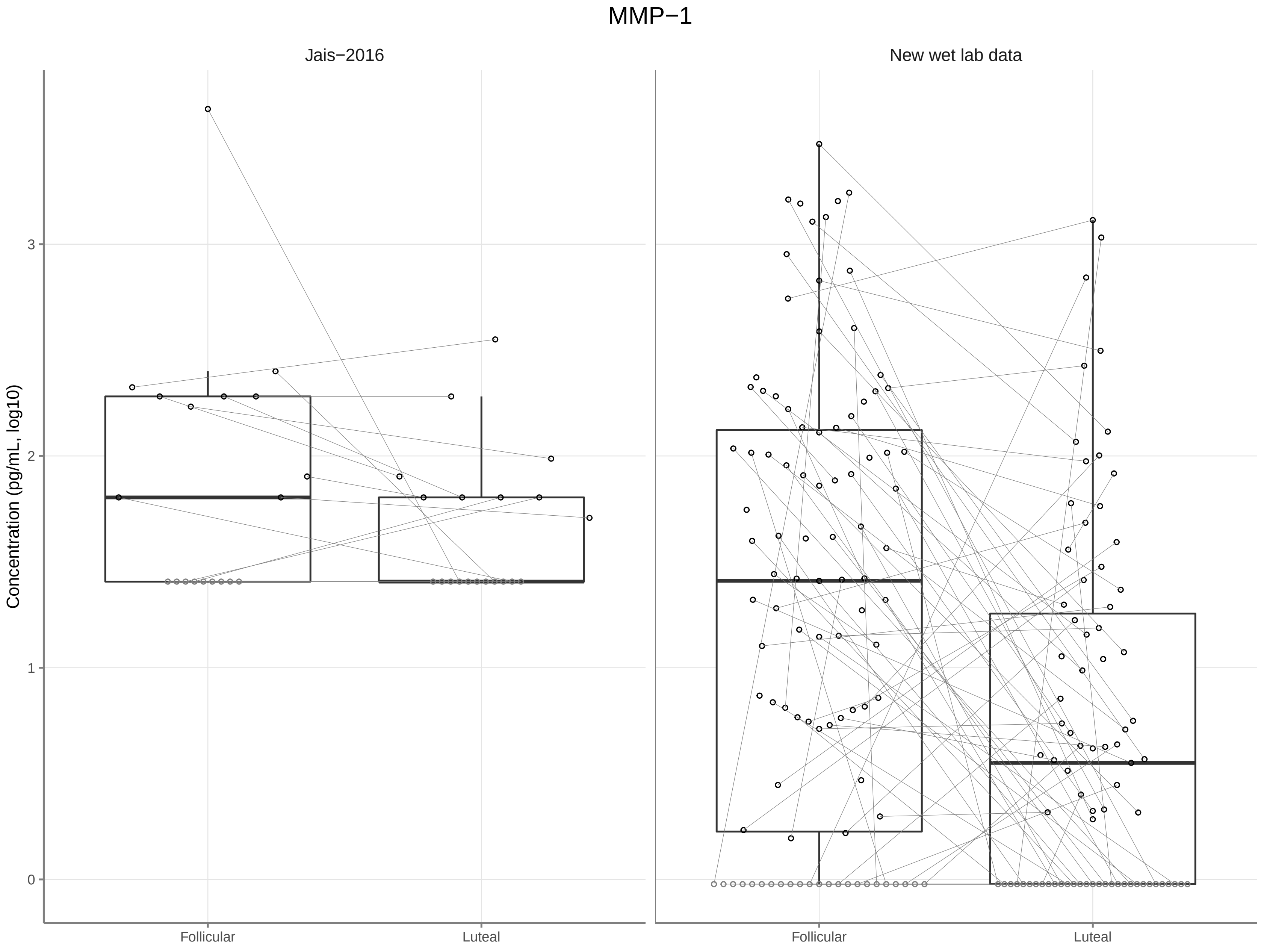

MMP−1
Jais−2016
New wet lab data
Concentration (pg/mL, log10)
3
2
1
0
Follicular
Luteal
Follicular
Luteal

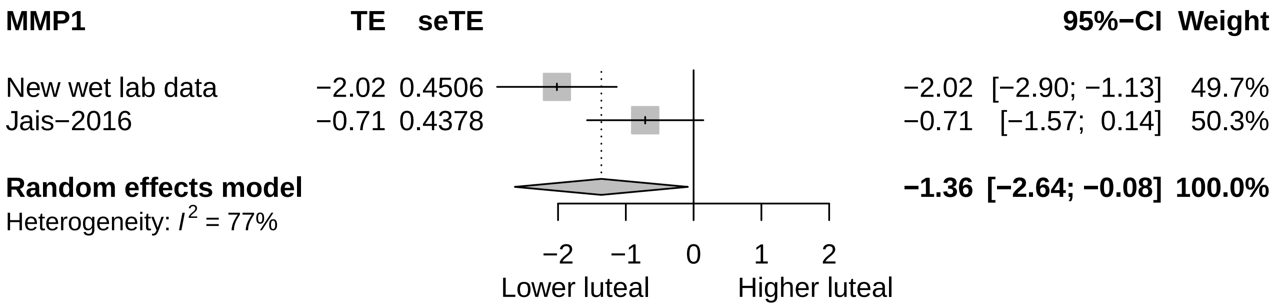
MMP1
TE seTE
95%−CI Weight
New wet lab data −2.02 0.4506 −2.02 [−2.90; −1.13] 49.7%
Jais−2016 −0.71 0.4378 −0.71 [−1.57; 0.14] 50.3%
Random effects model −1.36 [−2.64; −0.08] 100.0%
Heterogeneity: I2 = 77%
−2 −1 0 1 2
Lower luteal
Higher luteal

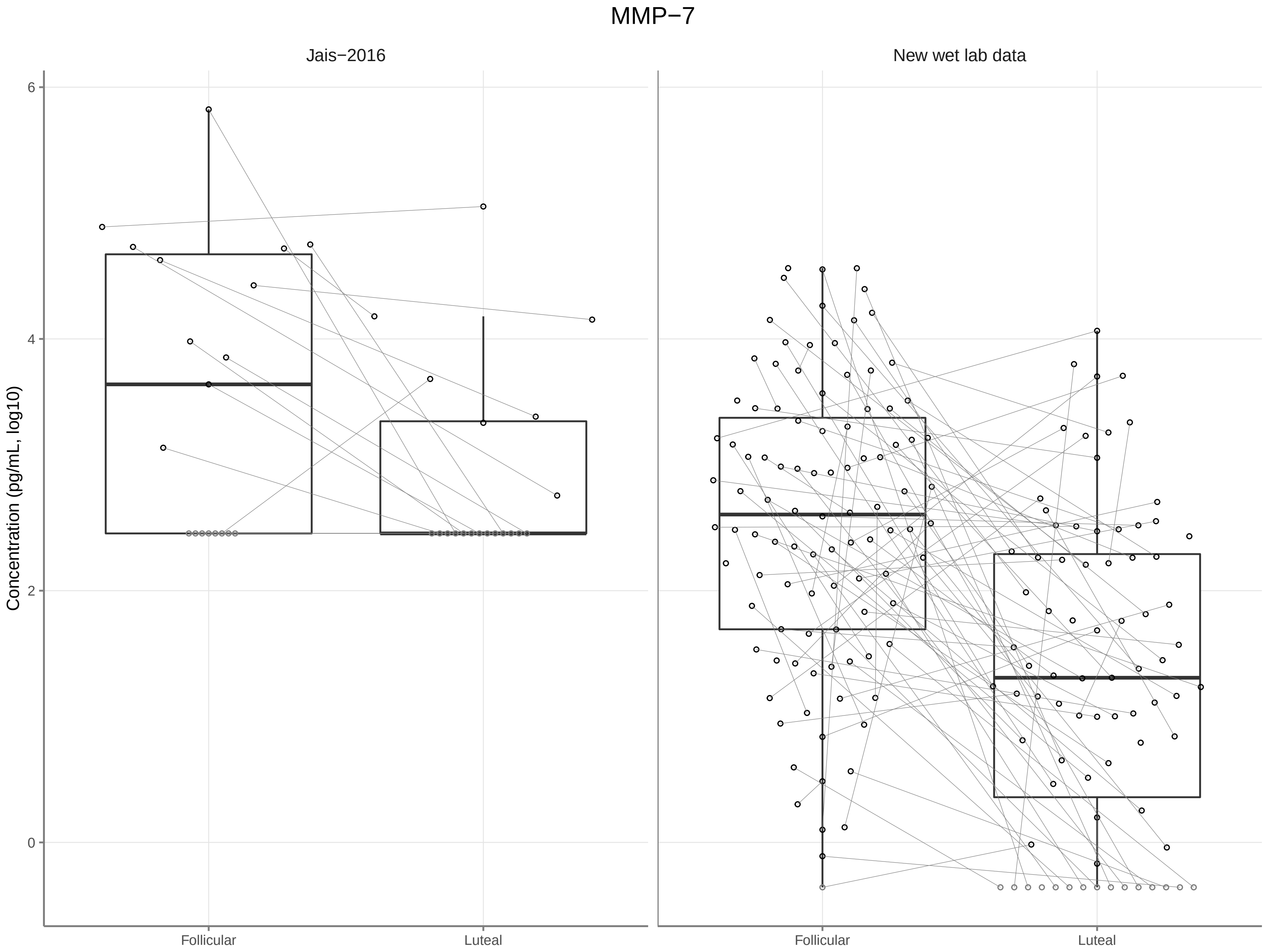

MMP−7
Jais−2016
New wet lab data
Concentration (pg/mL, log10)
6
4
2
0
Follicular
Luteal
Follicular
Luteal

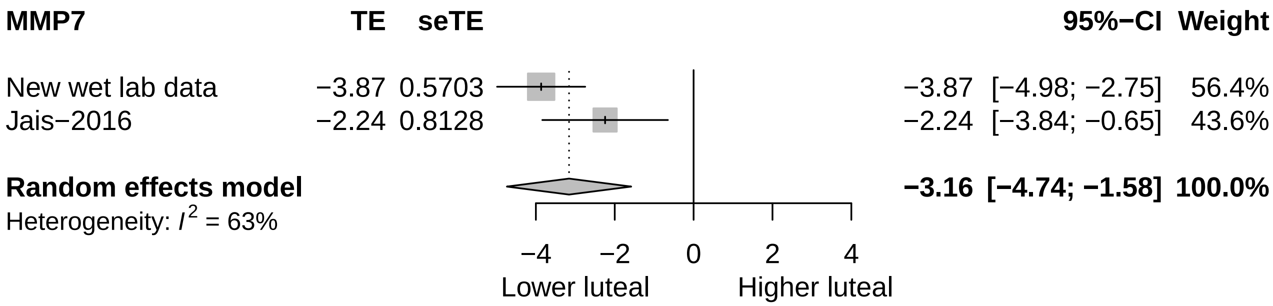
MMP7
TE
seTE
95%−CI
Weight
New wet lab data
−3.87
0.5703
−3.87
[−4.98; −2.75]
56.4%
Jais−2016
−2.24
0.8128
−2.24
[−3.84; −0.65]
43.6%
Random effects model
−3.16
[−4.74; −1.58]
100.0%
Heterogeneity: I2 = 63%
−4
−2
0
2
4
Lower luteal
Higher luteal

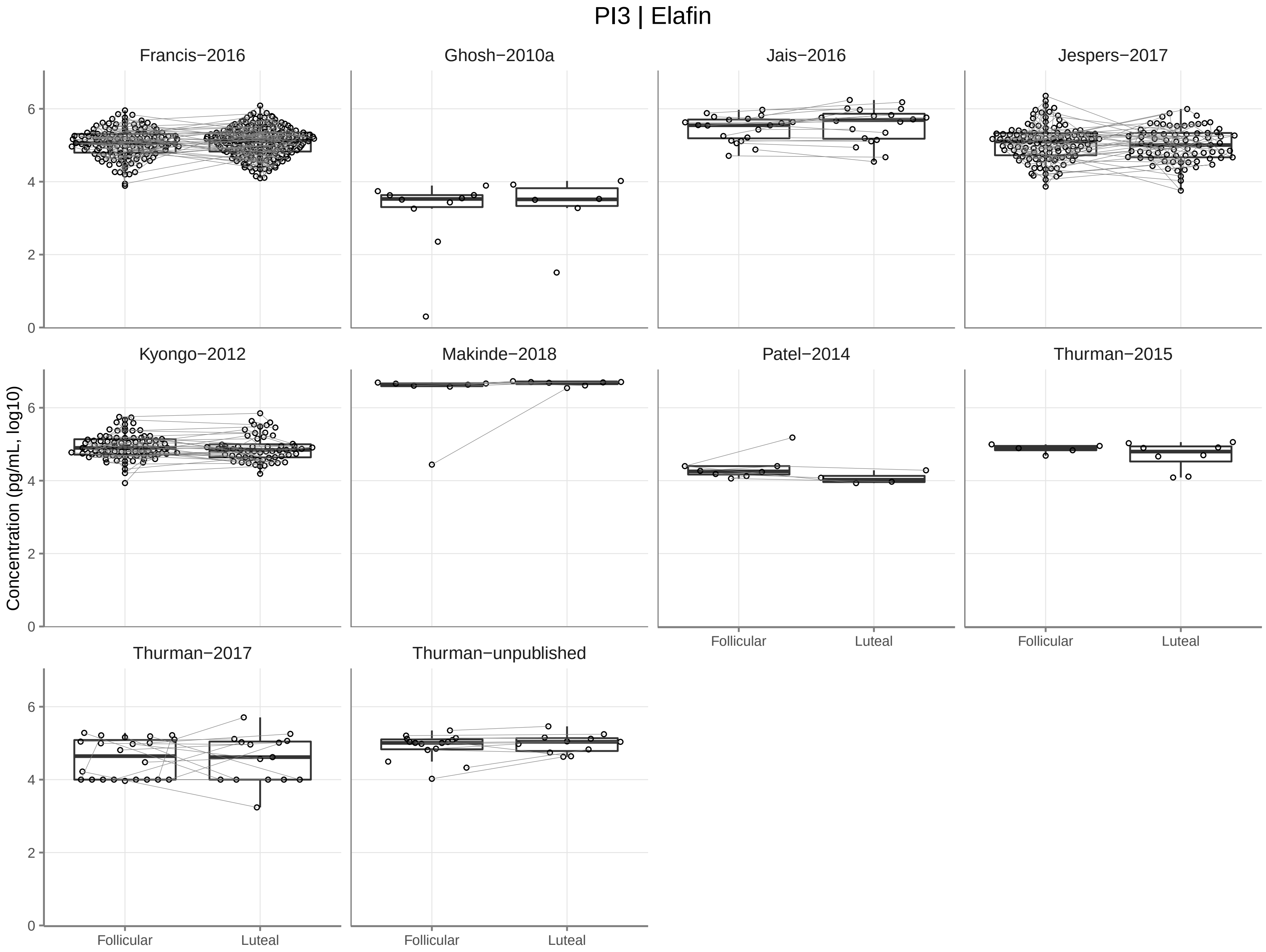

PI3 | Elafin
Francis−2016
Ghosh−2010a
Jais−2016
Jespers−2017
Kyongo−2012
Makinde−2018
Patel−2014
Thurman−2015
Thurman−2017
Thurman−unpublished
Concentration (pg/mL, log10)
0
2
4
6
Follicular
Luteal

PI3	TE	seTE		95%−CI	Weight
Patel−2014	−0.96	0.5999	−0.96	[−2.14; 0.21]	1.9%
Thurman−2015	−0.63	0.5986	−0.63	[−1.80; 0.54]	1.9%
Jespers−2017	−0.19	0.1993	−0.19	[−0.58; 0.20]	13.0%
Kyongo−2012	−0.15	0.1015	−0.15	[−0.35; 0.05]	28.7%
Francis−2016	−0.05	0.1036	−0.05	[−0.25; 0.15]	28.2%
Thurman−2017	−0.03	0.6569	−0.03	[−1.32; 1.26]	1.6%
Jais−2016	0.30	0.1780	0.30	[−0.05; 0.65]	15.2%
Thurman−unpublished	0.31	0.2576	0.31	[−0.20; 0.81]	8.7%
Ghosh−2010a	0.54	1.7575	0.54	[−2.90; 3.99]	0.2%
Makinde−2018	1.14	0.9762	1.14	[−0.77; 3.05]	0.7%
**Random effects model**			**−0.03**	**[−0.19; 0.13]**	**100.0%**

Heterogeneity: $I^2 = 25\%$

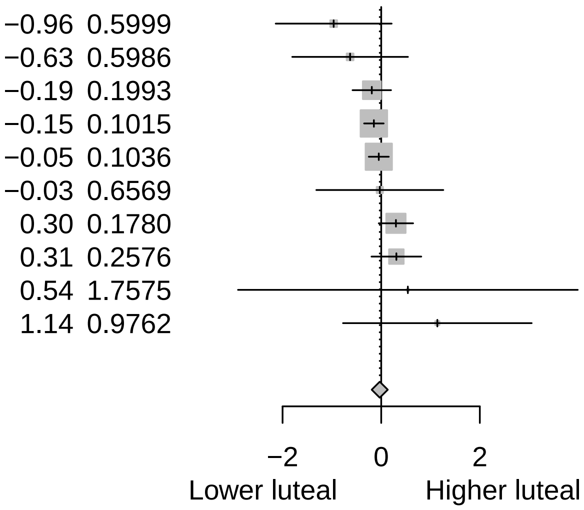

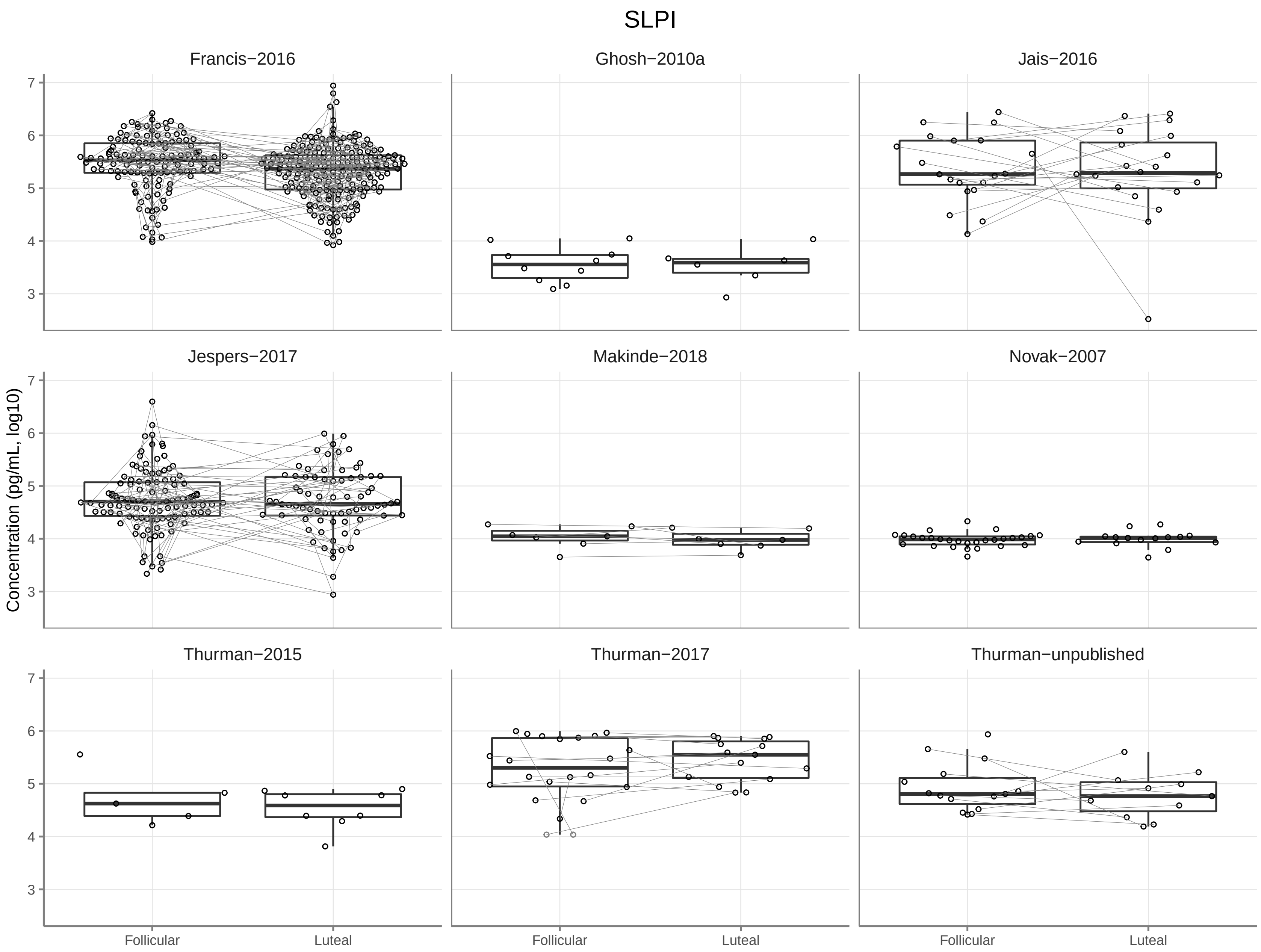

SLPI
Francis−2016
Ghosh−2010a
Jais−2016
Jespers−2017
Makinde−2018
Novak−2007
Thurman−2015
Thurman−2017
Thurman−unpublished
Concentration (pg/mL, log10)
3
4
5
6
7
Follicular
Luteal

SLPI	TE	seTE	95%−CI	Weight
Thurman−2015	−0.65	0.8204	−0.65 [−2.26; 0.96]	1.7%
Francis−2016	−0.51	0.1621	−0.51 [−0.83; −0.19]	20.9%
Thurman−unpublished	−0.46	0.5798	−0.46 [−1.59; 0.68]	3.2%
Jais−2016	−0.30	0.7978	−0.30 [−1.87; 1.26]	1.8%
Makinde−2018	−0.18	0.2216	−0.18 [−0.61; 0.26]	14.9%
Ghosh−2010a	−0.10	0.5953	−0.10 [−1.27; 1.07]	3.0%
Jespers−2017	−0.10	0.2515	−0.10 [−0.59; 0.40]	12.6%
Novak−2007	0.06	0.1475	0.06 [−0.23; 0.35]	22.8%
Shust−2010	0.07	0.2156	0.07 [−0.36; 0.49]	15.4%
Thurman−2017	0.50	0.5314	0.50 [−0.54; 1.54]	3.8%
**Random effects model**			**−0.14 [−0.35; 0.08]**	**100.0%**

Heterogeneity: I^2 = 11%

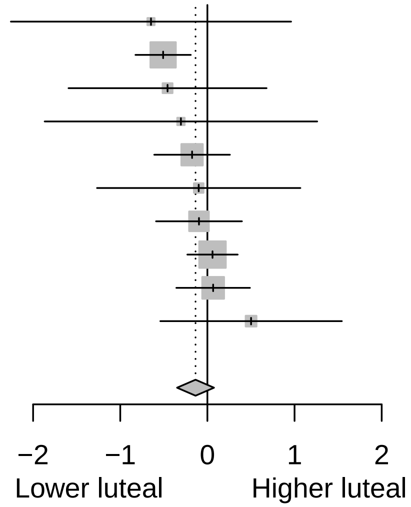

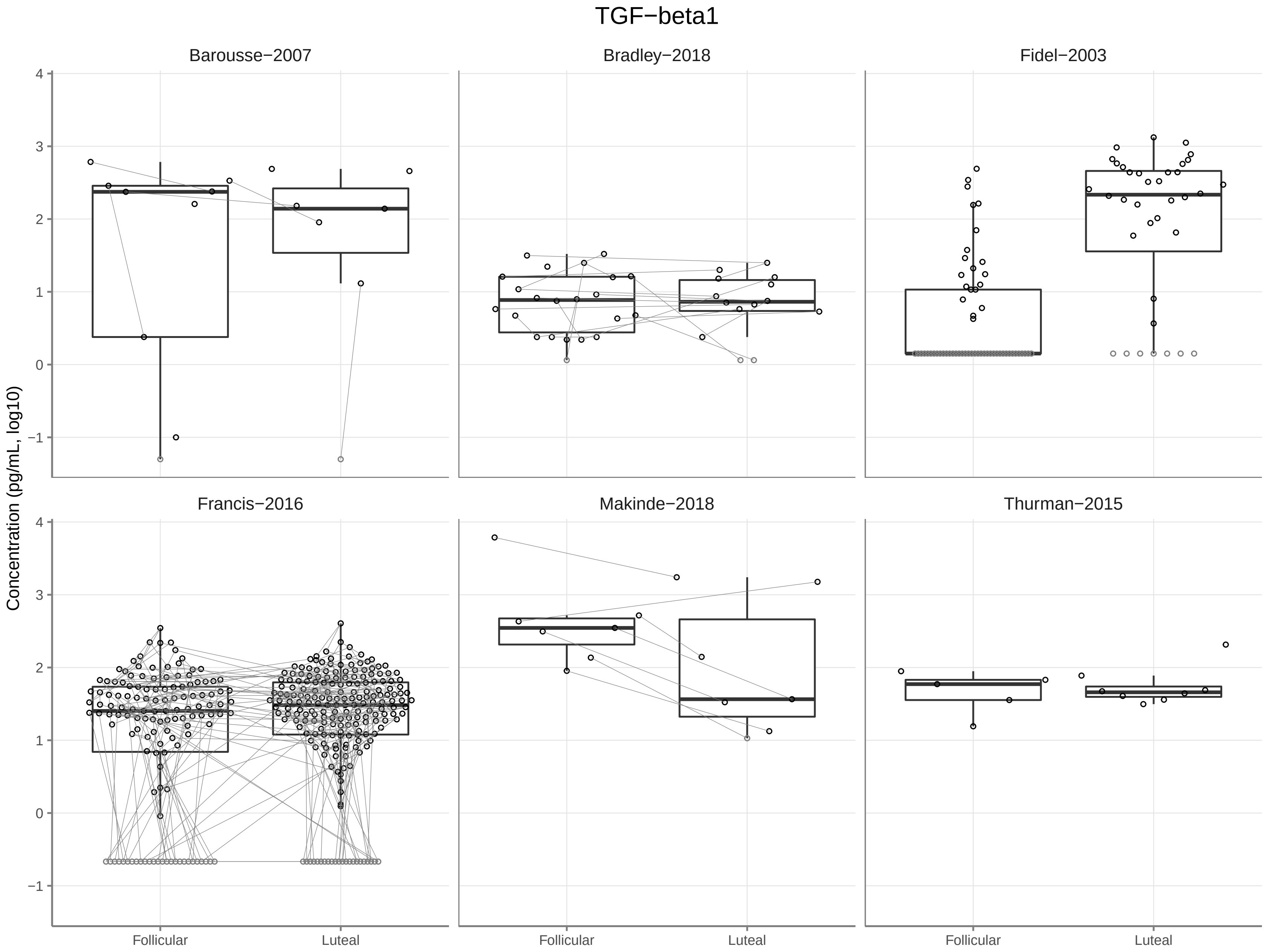
TGF−beta1
Barousse−2007
Bradley−2018
Fidel−2003
Francis−2016
Makinde−2018
Thurman−2015
Concentration (pg/mL, log10)
4
3
2
1
0
−1
Follicular
Luteal

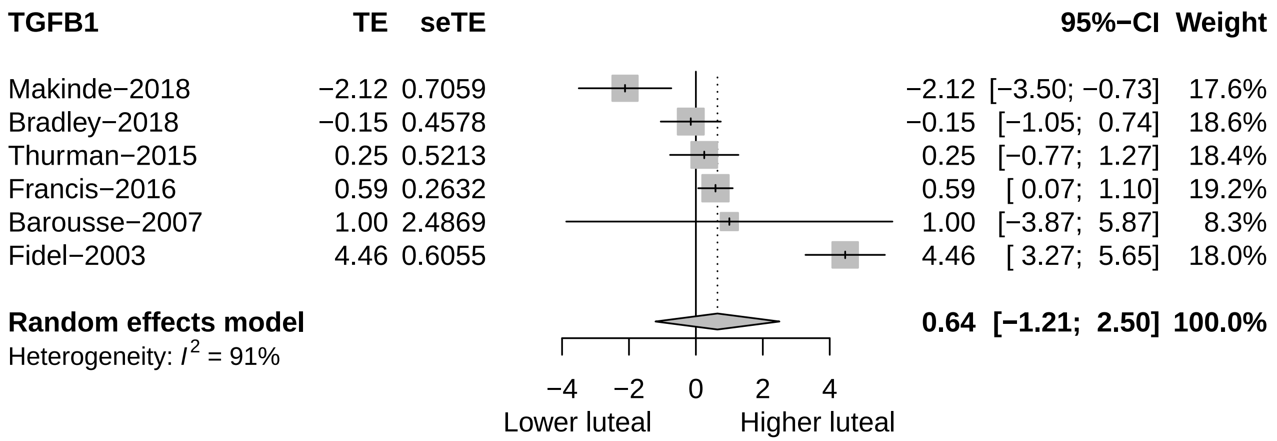
TGFB1
TE seTE
95%−CI Weight
Makinde−2018 −2.12 0.7059 −2.12 [−3.50; −0.73] 17.6%
Bradley−2018 −0.15 0.4578 −0.15 [−1.05; 0.74] 18.6%
Thurman−2015 0.25 0.5213 0.25 [−0.77; 1.27] 18.4%
Francis−2016 0.59 0.2632 0.59 [0.07; 1.10] 19.2%
Barousse−2007 1.00 2.4869 1.00 [−3.87; 5.87] 8.3%
Fidel−2003 4.46 0.6055 4.46 [3.27; 5.65] 18.0%
Random effects model 0.64 [−1.21; 2.50] 100.0%
Heterogeneity: I^2 = 91%
−4 −2 0 2 4
Lower luteal
Higher luteal

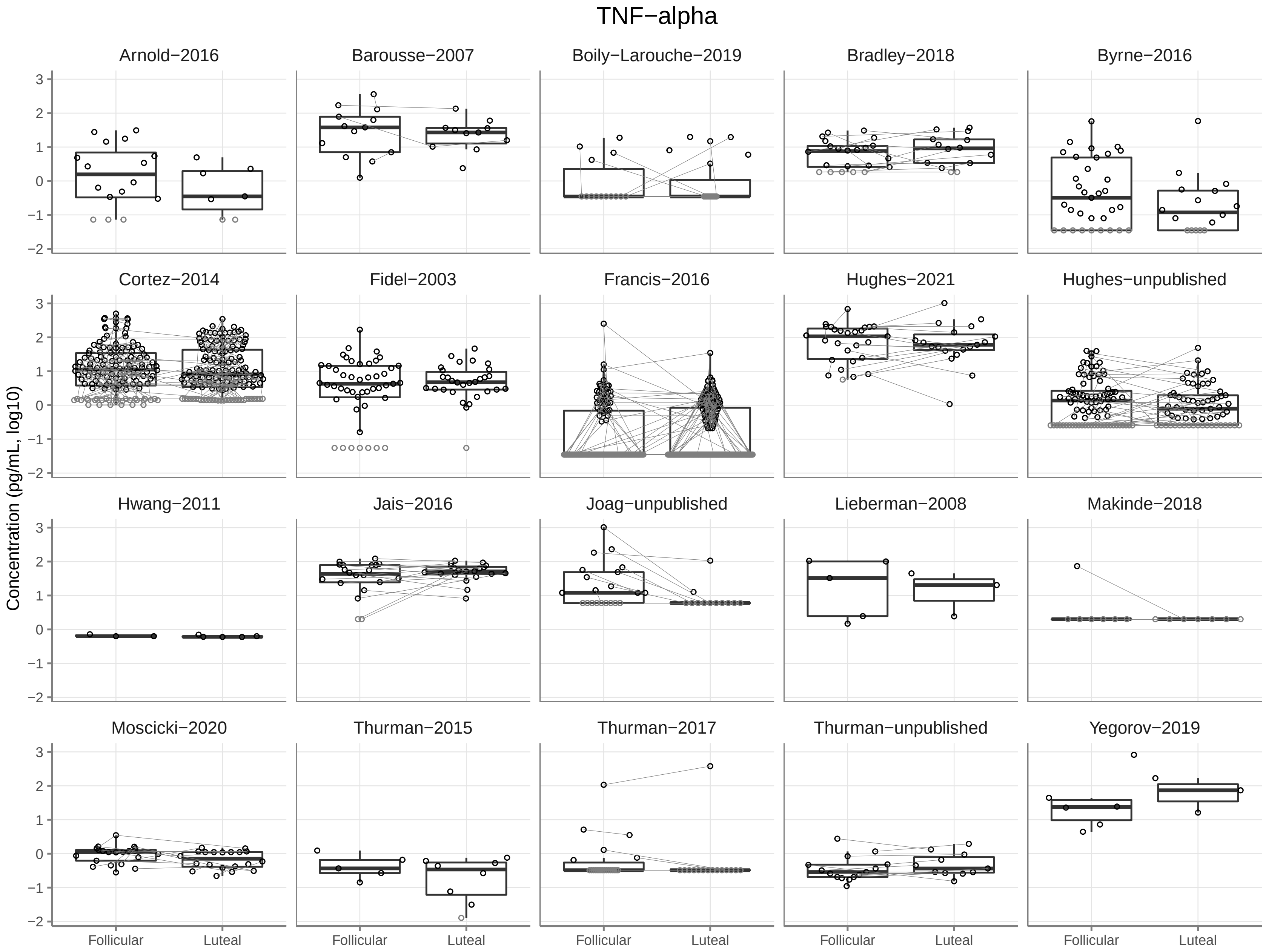
TNF−alpha
Arnold−2016
Barousse−2007
Boily−Larouche−2019
Bradley−2018
Byrne−2016
Cortez−2014
Fidel−2003
Francis−2016
Hughes−2021
Hughes−unpublished
Hwang−2011
Jais−2016
Joag−unpublished
Lieberman−2008
Makinde−2018
Moscicki−2020
Thurman−2015
Thurman−2017
Thurman−unpublished
Yegorov−2019
Concentration (pg/mL, log10)
Follicular
Luteal

TNF	TE	seTE	95%−CI	Weight
Arnold−2016	−1.52	1.3111	−1.52 [−4.09; 1.05]	0.5%
Joag−unpublished	−1.43	0.5814	−1.43 [−2.57; −0.29]	2.2%
Thurman−2015	−1.22	1.0863	−1.22 [−3.35; 0.90]	0.7%
Byrne−2016	−1.18	0.9578	−1.18 [−3.06; 0.70]	0.8%
Barousse−2007	−0.86	0.6416	−0.86 [−2.12; 0.40]	1.8%
Makinde−2018	−0.74	0.7423	−0.74 [−2.20; 0.71]	1.4%
Moscicki−2020	−0.50	0.2283	−0.50 [−0.94; −0.05]	11.3%
Hughes−unpublished	−0.40	0.2901	−0.40 [−0.96; 0.17]	7.7%
Lieberman−2008	−0.35	1.9795	−0.35 [−4.23; 3.53]	0.2%
Thurman−2017	−0.10	0.1918	−0.10 [−0.47; 0.28]	14.4%
Boily−Larouche−2019	−0.07	0.7513	−0.07 [−1.54; 1.41]	1.4%
Cortez−2014	−0.05	0.1872	−0.05 [−0.42; 0.31]	14.9%
Francis−2016	0.05	0.1951	0.05 [−0.33; 0.43]	14.1%
Thurman−unpublished	0.13	0.2223	0.13 [−0.31; 0.56]	11.7%
Hughes−2021	0.13	0.4279	0.13 [−0.70; 0.97]	3.9%
Bradley−2018	0.38	0.3444	0.38 [−0.29; 1.06]	5.8%
Jais−2016	0.53	0.3746	0.53 [−0.20; 1.27]	5.0%
Fidel−2003	0.55	0.6111	0.55 [−0.65; 1.75]	2.0%
Yegorov−2019	0.99	1.7115	0.99 [−2.36; 4.35]	0.3%
**Random effects model**			**−0.10 [−0.28; 0.07]**	**100.0%**

Heterogeneity: $I^2 = 23\%$

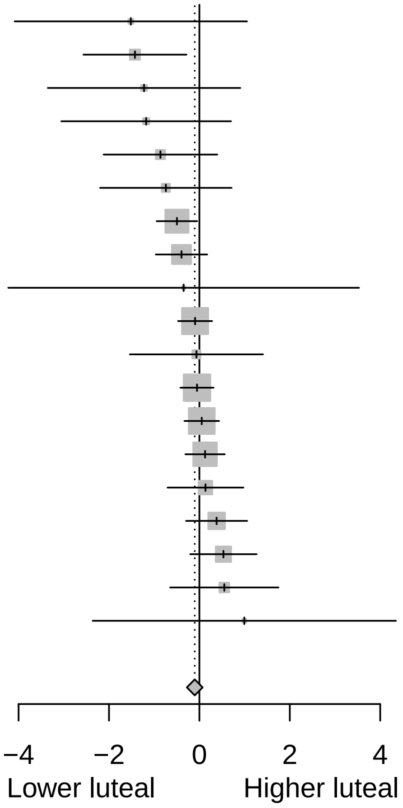

Log-odds difference in detectability between follicular and luteal phase

This section shows the meta-analyses for each immune factor where more than half of the samples were undetectable. For each immune mediator, there are two pages. The first page shows the individual participant data (concentrations of immune mediators) for each sample in each study. The second page shows a forest plot, showing the difference between luteal and follicular phase for each study separately.

Notes:

- New wet lab data indicates the exploratory and validation measurements performed for this meta-analysis, as described in the results section.

Concentration plots

Each symbol shows the concentration of the indicated immune mediator in a single sample. Each study is plotted separately. Lines connect samples from the same participant; in some cases participants provided multiple samples in the same phase, in which case multiple symbols within the same phase may be connected. Pale grey symbols are below the lower limit of detection and are assigned the value of half the lower limit of detection.

Forest plots

Each row represents a different study, with the vertical line in the middle of each square indicating the mean and the horizontal line indicating the 95% confidence interval. Positive numbers indicate greater detectability during the luteal phase (compared to the follicular phase), while negative numbers indicate lower detectability during the luteal phase (compared to the follicular phase). The size of the squares is proportional to how heavily the study is weighted in the meta-analysis.

The center of the diamond and the vertical dotted line indicates the meta-effect as determined by the random effects model. The width of the diamond indicates the 95% confidence interval of the meta-effect.

TE, treatment effect (log-odds of proportion detectable in luteal vs follicular phase); seTE, standard error of the treatment effect; 95%-CI, 95% confidence interval around the treatment effect; Weight, the percentage of the meta-estimate contributed by each study.

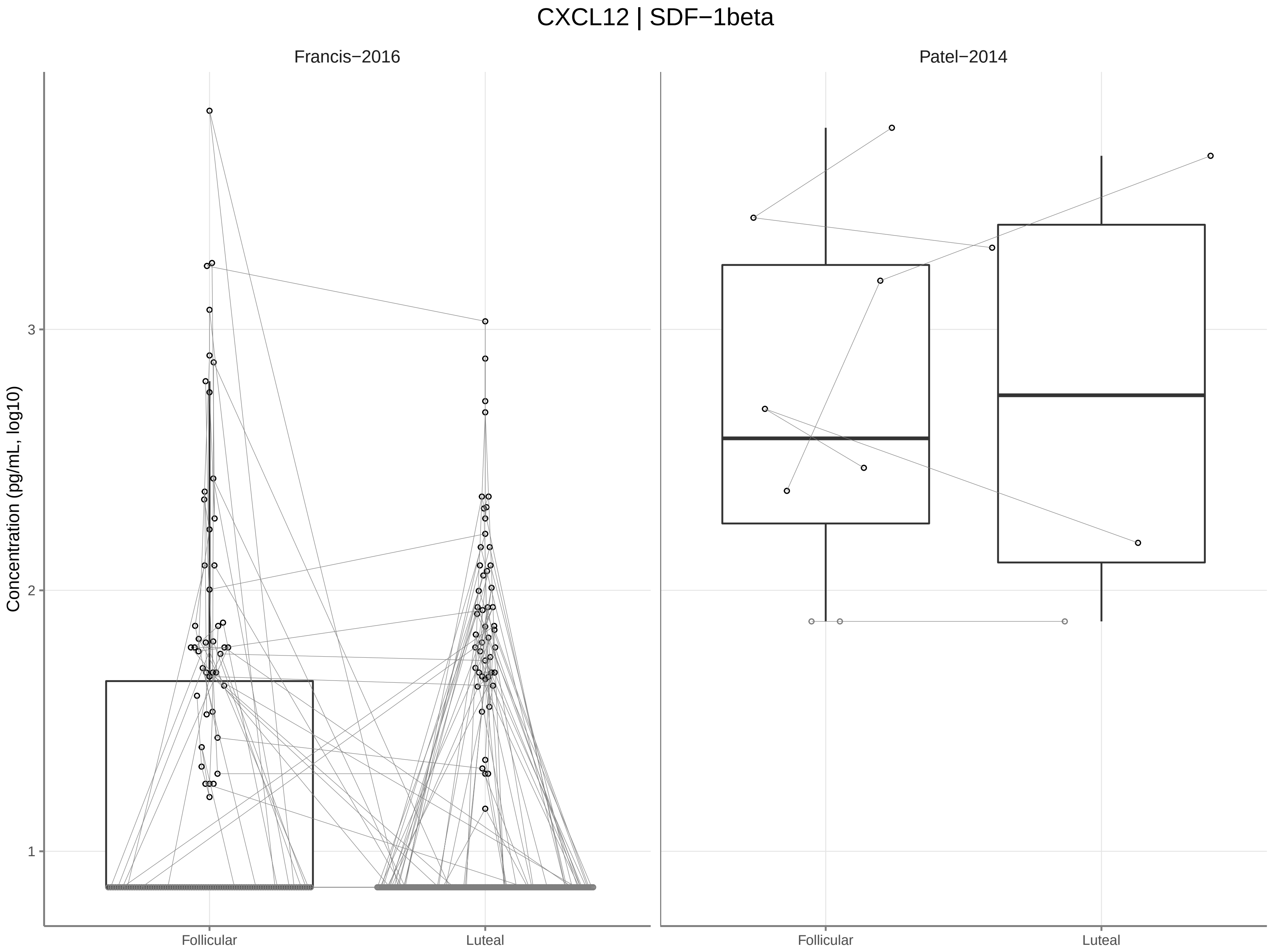
CXCL12 | SDF−1beta
Francis−2016
Patel−2014
Concentration (pg/mL, log10)
3
2
1
Follicular
Luteal
Follicular
Luteal

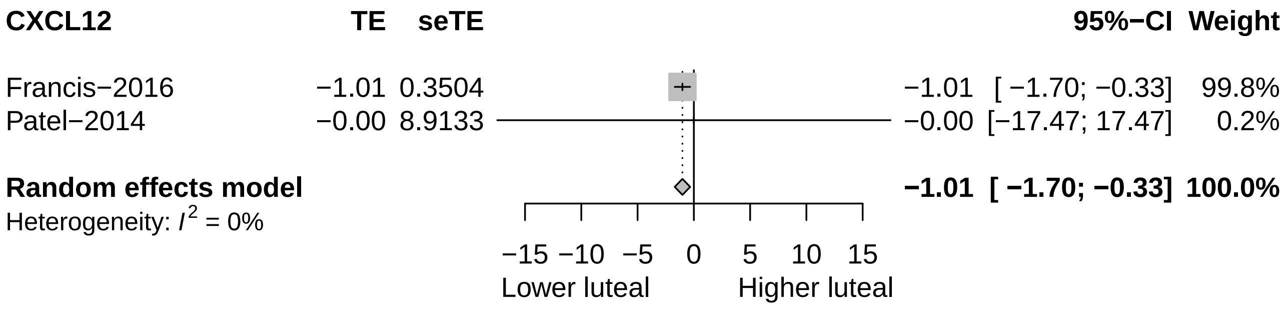
CXCL12
TE
seTE
95%−CI
Weight
Francis−2016 −1.01 0.3504 −1.01 [−1.70; −0.33] 99.8%
Patel−2014 −0.00 8.9133 −0.00 [−17.47; 17.47] 0.2%
Random effects model −1.01 [−1.70; −0.33] 100.0%
Heterogeneity: I2 = 0%
−15 −10 −5 0 5 10 15
Lower luteal
Higher luteal

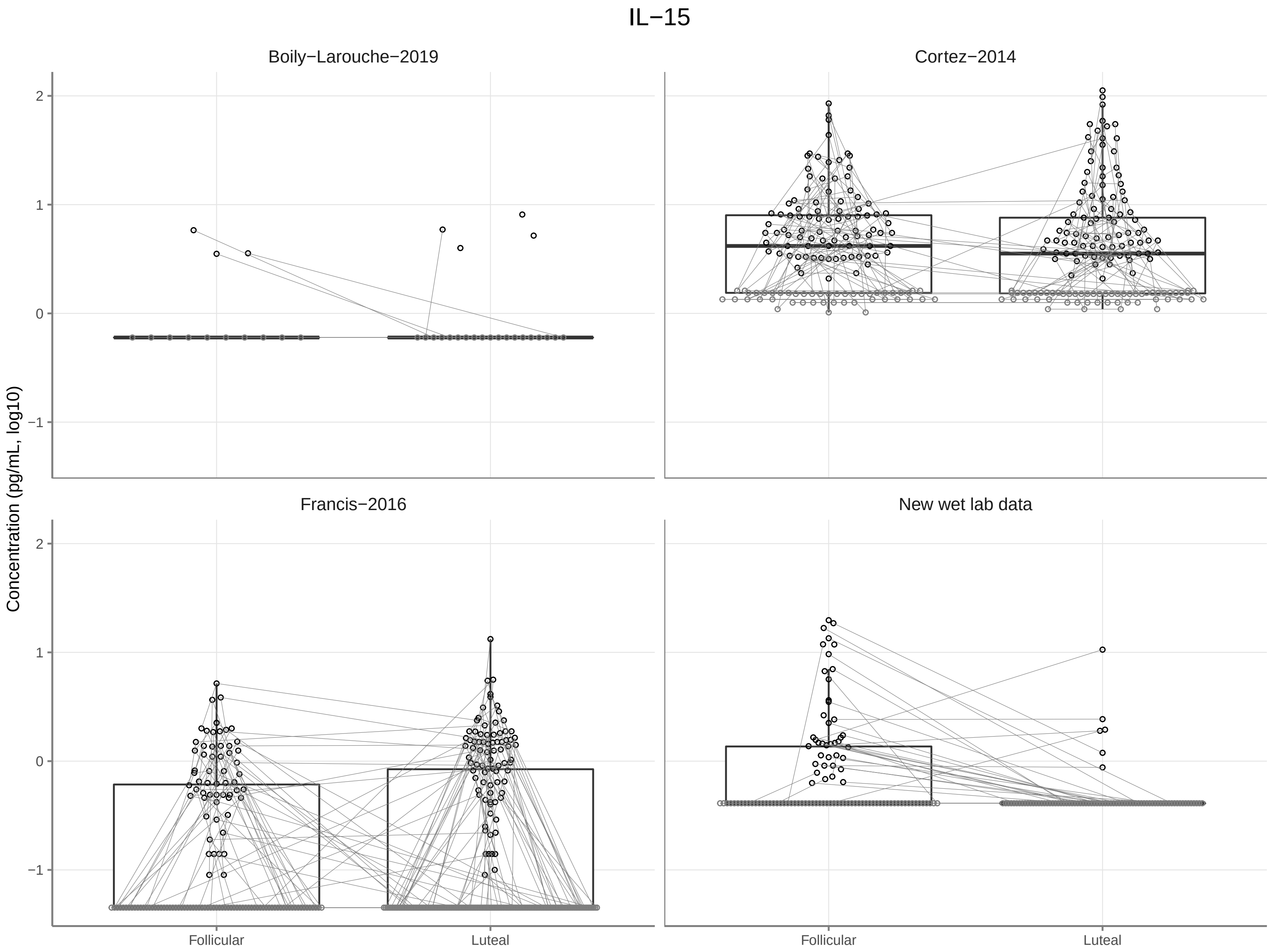

IL−15
Boily−Larouche−2019
Cortez−2014
Francis−2016
New wet lab data
Concentration (pg/mL, log10)
2
1
0
−1
Follicular
Luteal

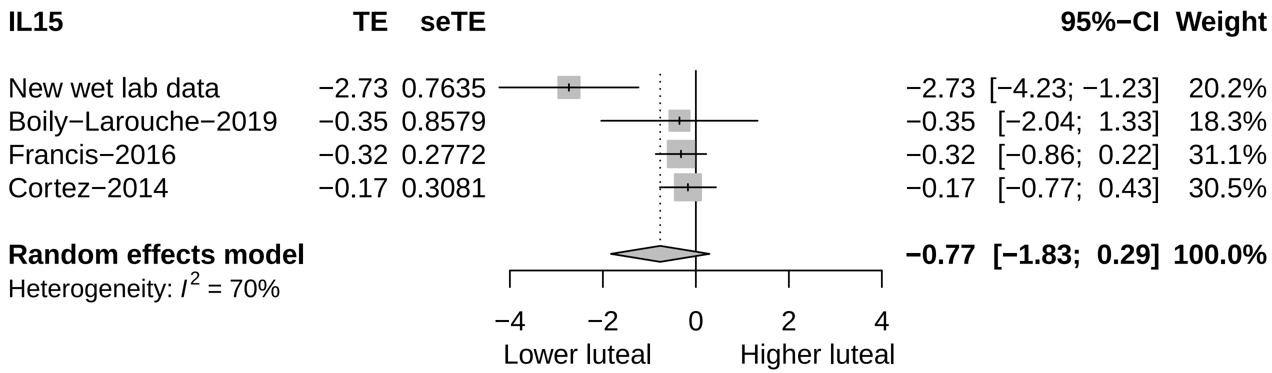
IL15
TE
seTE
95%−CI
Weight
New wet lab data −2.73 0.7635 −2.73 [−4.23; −1.23] 20.2%
Boily−Larouche−2019 −0.35 0.8579 −0.35 [−2.04; 1.33] 18.3%
Francis−2016 −0.32 0.2772 −0.32 [−0.86; 0.22] 31.1%
Cortez−2014 −0.17 0.3081 −0.17 [−0.77; 0.43] 30.5%
Random effects model −0.77 [−1.83; 0.29] 100.0%
Heterogeneity: I^2 = 70%
−4
−2
0
2
4
Lower luteal
Higher luteal
