# Supplementary material for: Changes in concentrations of cervicovaginal immune mediators across the menstrual cycle: a systematic review and meta-analysis of individual patient data
Source: BMC Med. 2022 Oct 5;20:353. doi: 10.1186/s12916-022-02532-9 (PMC9533580; doi:10.1186/s12916-022-02532-9)
Supplement: Supplementary file 5 — Additional file 5: Figure S1. Assessment of publication bias. A Funnel plots. Symbols show the effect of the menstrual cycle (x-axis) and the standard error of that effect (y-axis, reversed). Each symbol shows an individual study. Vertical solid line shows no effect. Vertical dashed line shows the meta-estimate of effect. Diagonal dashed lines enclose the region expected to include 95% of studies based on the estimated meta-effect and the standard errors. B Results of Egger’s tests for publication bias. Figure S2. Periovulatory meta-analyses. A The log2 difference between periovulatory and follicular phases (log2-pg/mL of the follicular phase minus log2-pg/mL of the periovulatory phase). For TGF-β1, the error bars for one study and the meta-estimate extend off-scale. B The log2 difference between periovulatory and luteal phases (log2-pg/mL of the luteal phase minus log2-pg/mL of the periovulatory phase). For IL-10, the error bars for one study extend off-scale. Each row represents a different immune mediator, with the symbols showing the mean and the lines showing the 95% confidence intervals. Gray symbols indicate individual studies and black the meta-estimates as determined by inverse-variance pooling random effects models. Black filled symbols indicate p < 0.05 while white filled symbols indicate p > 0.05. Positive numbers indicate higher during the follicular or luteal phase, while negative numbers indicate higher during the periovulatory phase. Fig S3. Subgroup analysis: Does the effect of menstrual cycle differ by assay method, geographical region, or method of determining menstrual phase? A Meta-analyses, comparing all studies (black circles) to studies grouped by assay method (ELISA: blue squares; MSD: yellow triangles; Luminex: green diamonds). B Meta-analyses, comparing all studies (black circles) to studies grouped by geographical region of sample origin (Africa: blue diamonds; Europe: red squares; North America: green triangles). C Meta-analyses, compari [file 12916_2022_2532_MOESM5_ESM.zip › Supplementary Tables (Table S1-S4).docx]

Table S1. Summary of immune mediators measured in single studies.

| Immune mediator | Name | Study | Median (IQR) | Percent detected | Model | Difference | Standard error | P-value |
| --- | --- | --- | --- | --- | --- | --- | --- | --- |
| APOA1 | apolipoprotein A1 | Francis-2016 | 47.7 (9.5-388.1) | 99 | Linear | -1.77 | 0.32 | 8.3E-8 |
| Albumin | albumin | Francis-2016 | 2.3E6 (9.7E5-5.4E6) | 100 | Linear | -0.67 | 0.18 | 1.9E-4 |
| IgE | immunoglobulin E | Fidel-2003 | 5.7E4 (1.0E4-1.2E5) | 85 | Linear | -2.91 | 0.76 | 2.5E-4 |
| IVL | involucrin | Francis-2016 | 3.4E5 (1.2E5-1.1E6) | 99 | Linear | -0.87 | 0.26 | 0.001 |
| PIGR | polymeric immunoglobulin receptor | Francis-2016 | 6.3E4 (2.1E4-1.4E5) | 99 | Linear | -0.44 | 0.18 | 0.016 |
| SELL \| CD62L | selectin L | Makinde-2018 | 276.7 (88-673.3) | 88 | Linear | -2.63 | 0.98 | 0.044 |
| TGF-α | transforming growth factor alpha | Francis-2016 | 23.4 (11.7-42.1) | 92 | Linear | -0.23 | 0.12 | 0.068 |
| CTSB | cathepsin B | Jais-2017 | 2.2E4 (9.4E3-3.3E4) | 98 | Linear | -0.52 | 0.28 | 0.078 |
| MMP-2 | matrix metallopeptidase 2 | Jais-2016 | 700 (525-700) | 75 | Linear | -0.80 | 0.43 | 0.081 |
| IL2RA \| CD25 | interleukin 2 receptor subunit alpha | Boily-Larouche-2019 | 3 (3-3) | 14 | Logistic | -1.54 | 0.95 | 0.106 |
| IL-3 | interleukin 3 | Cortez-2014 | 3.5 (1.9-7) | 60 | Linear | 0.11 | 0.089 | 0.211 |
| LT-α | lymphotoxin alpha | Cortez-2014 | 2.7 (2-6.3) | 40 | Logistic | 0.24 | 0.32 | 0.454 |
| SELP \| CD62 | selectin P | Makinde-2018 | 2.2E5 (1.9E5-2.6E5) | 100 | Linear | 0.15 | 0.23 | 0.540 |
| VEGF | vascular endothelial growth factor A | Lieberman-2008 | 524.9 (231.2-1017.3) | 99 | Linear | -0.55 | 0.94 | 0.579 |
| DEFA5 \| HD-5 | defensin alpha 5 | Thurman-2017 | 1.0E6 (8.4E5-1.3E6) | 100 | Linear | -0.07 | 0.12 | 0.591 |
| IL-5 | interleukin 5 | Cortez-2014 | 1.1 (1-2) | 51 | Logistic | 0.18 | 0.34 | 0.595 |
| SERPINB3 \| SCCA-1 | serpin family B member 3 | Francis-2016 | 1.1E5 (3.8E4-2.5E5) | 98 | Linear | 0.10 | 0.19 | 0.602 |
| DEFB104A \| HBD-4 | defensin beta 104A | Francis-2016 | 230.7 (61.1-591.1) | 80 | Linear | -0.13 | 0.25 | 0.614 |
| S100A8 | S100 calcium binding protein A8 | Francis-2016 | 1.0E4 (6.3E3-1.6E4) | 100 | Linear | -0.02 | 0.086 | 0.800 |
| TGF-β2 | transforming growth factor beta 2 | Thurman-2015 | 279.2 (199.6-400.6) | 97 | Linear | -0.19 | 1.05 | 0.858 |
| MADCAM1 | mucosal vascular addressin cell adhesion molecule 1 | Novak-2007 | 175.7 (175.7-353.2) | 26 | Logistic | -0.12 | 0.68 | 0.859 |
| IL12p40 | interleukin 12 p40 | Cortez-2014 | 14.8 (5.6-36.5) | 77 | Linear | -0.03 | 0.17 | 0.860 |
| MMP-9 | matrix metallopeptidase 9 | Jais-2016 | 2.0E4 (4.5E3-4.5E4) | 98 | Linear | 0.02 | 0.71 | 0.981 |
| TGF-β3 | transforming growth factor beta 3 | Thurman-2015 | 0.4 (0.4-1.2) | 36 | Logistic | 36.97 | 1496 | 0.98 |

Median and interquartile ranges of concentrations are given in pg/mL. Percent detected indicates the percent of samples in which the immune mediator was measured above the lower limit of detection. The model column indicates the type of model used, either linear if the immune mediator was detectable in more than half of samples, or logistic if not. For linear models, the Difference column shows log2 difference between phases (log2-pg/mL of the luteal phase minus log2-pg/mL of the follicular phase). For logistic models, the Difference column shows logistic fold change between phases (log-odds of proportion detectable in luteal vs. follicular phase). Positive numbers indicate higher concentrations in the luteal phase (relative to the follicular phase), while negative numbers indicates lower concentrations in the luteal phase (relative to the follicular phase).

Table S2. Summary of follicular vs. periovulatory meta-analyses.

| Category | Immune mediator | Name | Log2 difference | Standard error | P-value | FDR | Holm-Bonferroni | I^2^ | Number of studies |
| --- | --- | --- | --- | --- | --- | --- | --- | --- | --- |
| Chemokine CX-type | CXCL8 \| IL-8 | C-X-C motif chemokine ligand 8 | 0.74 | 0.34 | 0.029 | 0.143 | 0.257 | 0 | 2 |
| Interferon | IFN-γ | interferon gamma | 0.11 | 0.25 | 0.677 | 0.777 | 1 | 0 | 3 |
| Interleukin 1 family | IL-1α | interleukin 1 alpha | -1.19 | 0.94 | 0.205 | 0.514 | 1 | 88 | 3 |
| Interleukin 2 family | IL-2 | interleukin 2 | 1.79 | 1.79 | 0.317 | 0.528 | 1 | 92 | 2 |
|  | IL-4 | interleukin 4 | 0.43 | 0.14 | 0.002 | 0.022 | 0.022 | 0 | 2 |
| Interleukin Other | IL-10 | interleukin 10 | 1.65 | 1.57 | 0.295 | 0.528 | 1 | 94 | 3 |
|  | IL-6 | interleukin 6 | 0.72 | 0.35 | 0.043 | 0.143 | 0.342 | 27 | 3 |
|  | IL12p70 | interleukin 12 p70 | 0.1 | 0.27 | 0.699 | 0.777 | 1 | 0 | 2 |
| Other | TNF-α | tumor necrosis factor | -0.19 | 0.95 | 0.837 | 0.837 | 1 | 85 | 3 |
|  | TGF-β1 | transforming growth factor beta 1 | -1.99 | 2.59 | 0.443 | 0.633 | 1 | 97 | 2 |

Log2 difference, difference between phases (log2-pg/mL of the follicular phase minus log2-pg/mL of the periovulatory phase) with positive numbers indicating higher concentrations in the follicular phase; FDR, false discovery rate; I^2^, statistical heterogeneity between studies, from low (0) to high (100).

Table S3. Summary of luteal vs. periovulatory meta-analyses.

| Category | Immune mediator | Name | Log2 difference | Standard error | P-value | FDR | Holm-Bonferroni | I^2^ | Number of studies |
| --- | --- | --- | --- | --- | --- | --- | --- | --- | --- |
| Chemokine CX-type | CXCL8 \| IL-8 | C-X-C motif chemokine ligand 8 | 0.67 | 0.34 | 0.046 | 0.459 | 0.459 | 0 | 2 |
| Interferon | IFN-γ | interferon gamma | 1.12 | 1.01 | 0.268 | 0.579 | 1 | 82 | 3 |
| Interleukin 1 family | IL-1α | interleukin 1 alpha | 0.29 | 0.37 | 0.438 | 0.626 | 1 | 35 | 3 |
| Interleukin 2 family | IL-2 | interleukin 2 | 1.92 | 2.04 | 0.347 | 0.579 | 1 | 93 | 2 |
|  | IL-4 | interleukin 4 | 0.07 | 0.15 | 0.629 | 0.786 | 1 | 0 | 2 |
| Interleukin Other | IL-10 | interleukin 10 | 2.29 | 1.84 | 0.215 | 0.579 | 1 | 96 | 3 |
|  | IL12p70 | interleukin 12 p70 | 1.98 | 1.98 | 0.318 | 0.579 | 1 | 96 | 2 |
|  | IL-6 | interleukin 6 | 0.67 | 0.71 | 0.346 | 0.579 | 1 | 67 | 3 |
| Other | TGF-β1 | transforming growth factor beta 1 | 0.19 | 0.5 | 0.707 | 0.786 | 1 | 0 | 2 |
|  | TNF-α | tumor necrosis factor | 0.12 | 0.81 | 0.881 | 0.881 | 1 | 78 | 3 |

Log2 difference, difference between phases (log2-pg/mL of the luteal phase minus log2-pg/mL of the periovulatory phase) with positive numbers indicating higher concentrations in the luteal phase; FDR, false discovery rate; I^2^, statistical heterogeneity between studies, from low (0) to high (100).

Table S4. Covariates adjusted for in multivariate analysis of each study.

| **Study name** | **Covariates** |
| --- | --- |
| Byrne-2016 | None - no covariates available |
| Makinde-2018 | None - no covariates available |
| Patel-2014 | None - no covariates available |
| Barousse-2007 | None - too few samples |
| Novak-2007 | None - too few samples |
| Thurman-2015 | None - too few samples |
| Arnold-2016 | Age, Smoking, HPV, HSV-2 serology, Mycoplasma genitalium infection |
| Boily-Larouche-2019 | BV |
| Bradley-2018 | Lactobacillus dominant flora |
| Castle-2002 | Blood detection, HPV, Reproductive tract infection |
| Cortez-2014 | BV, CD4 count, Cervical HIV viral load, Vaginal washing, Gonorrhea, Gravidity, Syphilis, Trichomonas, Vaginal HIV viral load, Vaginal pH, Yeast |
| Fidel-2003 | Serum estradiol (log2) |
| Francis-2016 | BV, Blood detection, PSA (ng/mL), Trichomonas |
| Ghosh-2010 | BV, CD4 count, Race, Serum HIV, Trichomonas |
| Hughes-2021 | Blood detection, BV, Condom use, Days since last intercourse, Hsv2, Vaginal pH, Yeast |
| Hughes-unpublished | Age, BV, Chlamydia, HSV-2 serology, PSA, Whether participant has ever engaged in sexual intercourse |
| Hwang-2011 | Age, Ectopy, PMN in wet mount |
| Jais-2016 | Age, BV, Blood detection, WBC in CVL (log2 count), Race, Vaginal pH |
| Jais-2017 | Age, BV, Blood detection, WBC in CVL (log2 count), Race, Trichomonas, Vaginal pH |
| Jespers-2017 | BV, Cervical epithelial abnormalities, Cervical mucus quantity, Vaginal washing, HSV-2 serology, PSA, Vaginal discharge, Vaginal pH |
| Joag-unpublished | Age, BV, Vaginal pH |
| Kyongo-2012 | Age, BV, Ectopy, PSA, Vaginal pH |
| Lahey-2012 | BV, CD4 count, Race, Serum HIV viral load, Trichomonas |
| Lieberman-2008 | Days since last intercourse, WBC in wet mount |
| Moscicki-2020 | Age, HPV |
| New wet lab data | Blood detection, BV, Chlamydia, PSA, Trichomonas |
| Sriprasert-2020 | Age, BMI, Presence of intermediate and/or superficial cells, Vaginal pH |
| Thurman-2017 | Age, BMI, BV, Gravidity, Parity, Race, Vaginal pH |
| Thurman-unpublished | Age, BMI, BV, Gravidity, Parity, Race |
| Yegorov-2019 | BV |
