# Supplementary figures and images for: Changes in concentrations of cervicovaginal immune mediators across the menstrual cycle: a systematic review and meta-analysis of individual patient data
Source: BMC Med. 2022 Oct 5;20:353. doi: 10.1186/s12916-022-02532-9 (PMC9533580; doi:10.1186/s12916-022-02532-9)

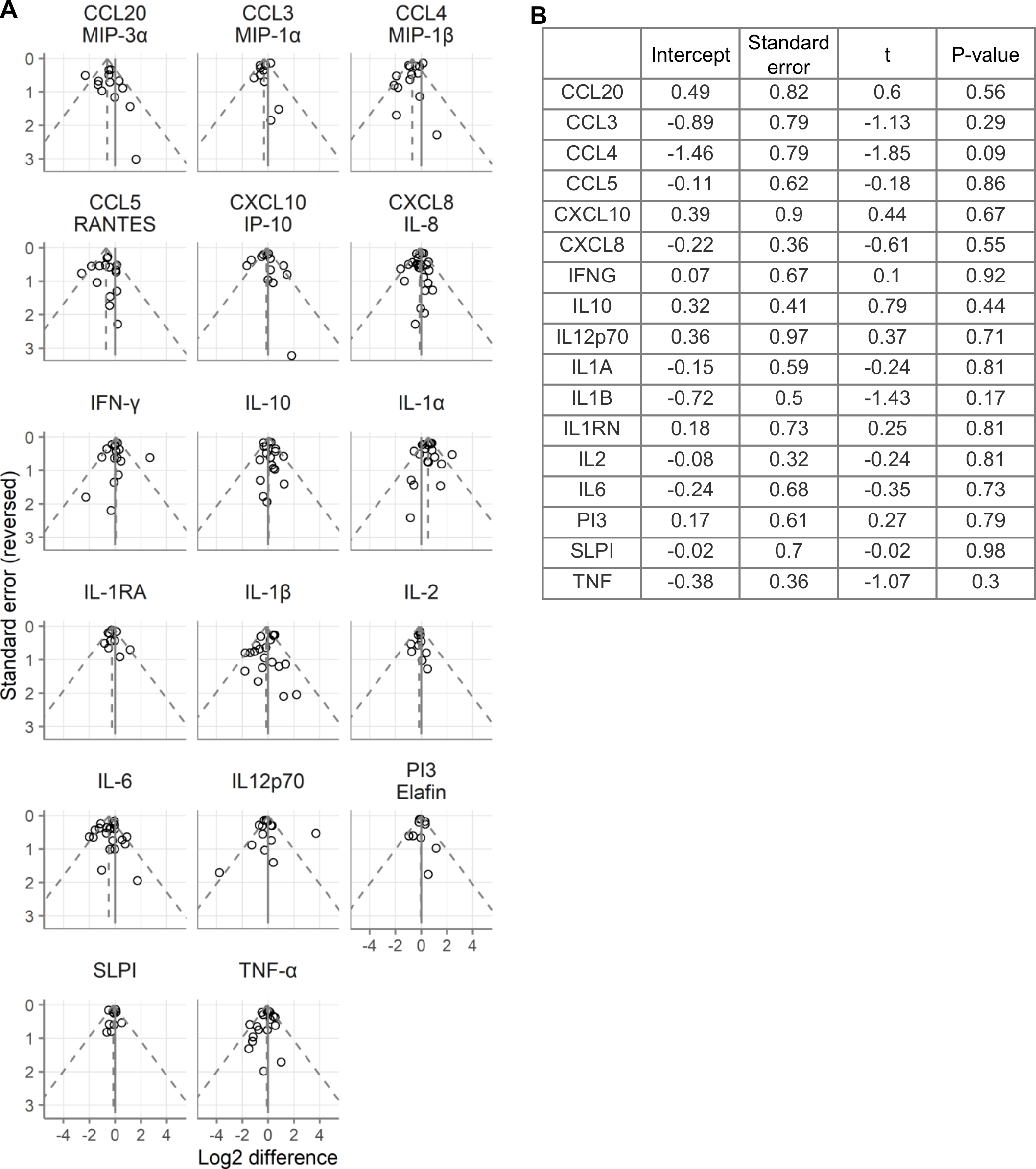

Supplement: Supplementary file 5 — Additional file 5: Figure S1. Assessment of publication bias. A Funnel plots. Symbols show the effect of the menstrual cycle (x-axis) and the standard error of that effect (y-axis, reversed). Each symbol shows an individual study. Vertical solid line shows no effect. Vertical dashed line shows the meta-estimate of effect. Diagonal dashed lines enclose the region expected to include 95% of studies based on the estimated meta-effect and the standard errors. B Results of Egger’s tests for publication bias. Figure S2. Periovulatory meta-analyses. A The log2 difference between periovulatory and follicular phases (log2-pg/mL of the follicular phase minus log2-pg/mL of the periovulatory phase). For TGF-β1, the error bars for one study and the meta-estimate extend off-scale. B The log2 difference between periovulatory and luteal phases (log2-pg/mL of the luteal phase minus log2-pg/mL of the periovulatory phase). For IL-10, the error bars for one study extend off-scale. Each row represents a different immune mediator, with the symbols showing the mean and the lines showing the 95% confidence intervals. Gray symbols indicate individual studies and black the meta-estimates as determined by inverse-variance pooling random effects models. Black filled symbols indicate p < 0.05 while white filled symbols indicate p > 0.05. Positive numbers indicate higher during the follicular or luteal phase, while negative numbers indicate higher during the periovulatory phase. Fig S3. Subgroup analysis: Does the effect of menstrual cycle differ by assay method, geographical region, or method of determining menstrual phase? A Meta-analyses, comparing all studies (black circles) to studies grouped by assay method (ELISA: blue squares; MSD: yellow triangles; Luminex: green diamonds). B Meta-analyses, comparing all studies (black circles) to studies grouped by geographical region of sample origin (Africa: blue diamonds; Europe: red squares; North America: green triangles). C Meta-analyses, compari [file 12916_2022_2532_MOESM5_ESM.zip › figS1R3.tiff]

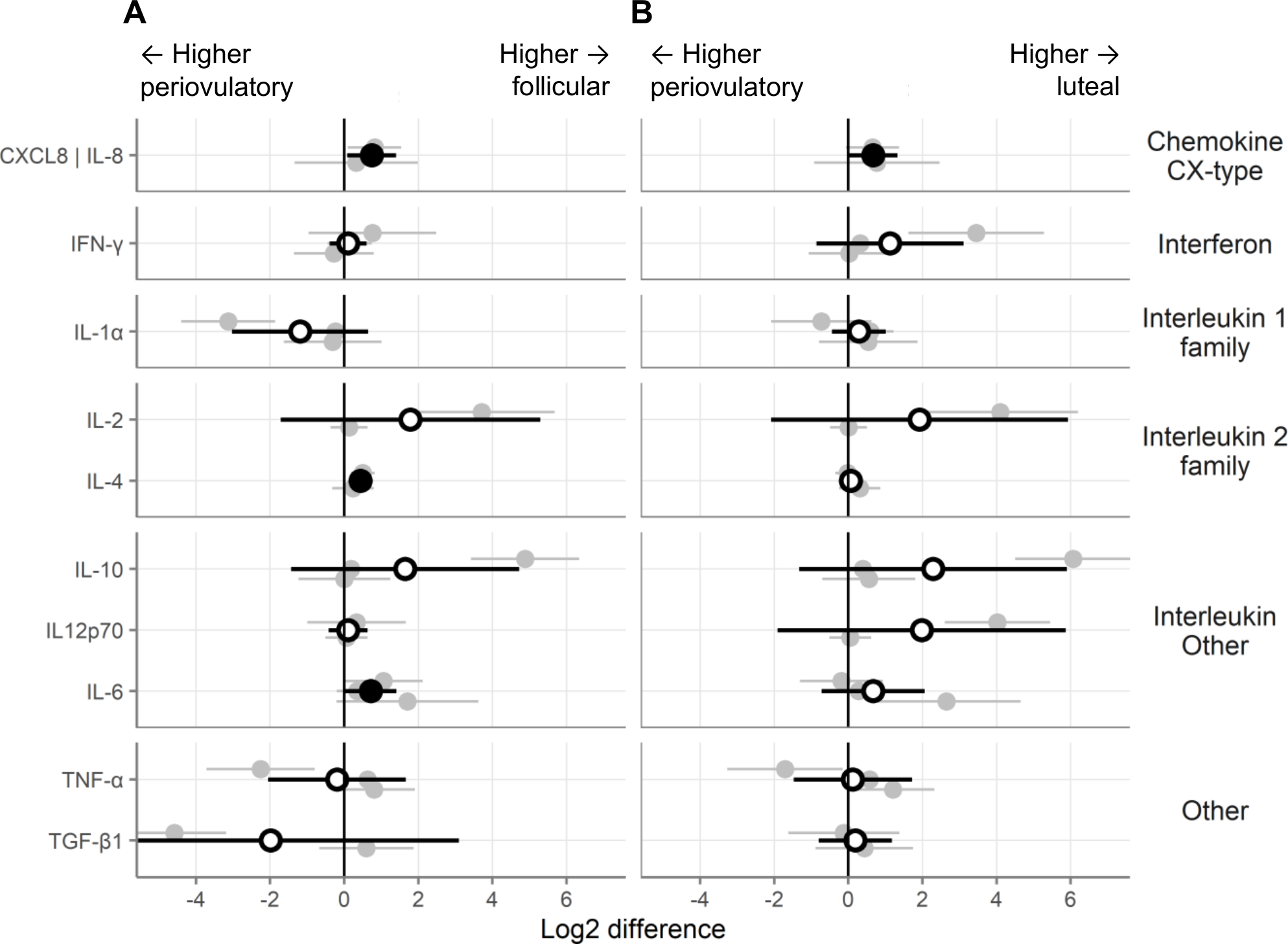

Supplement: Supplementary file 5 — Additional file 5: Figure S1. Assessment of publication bias. A Funnel plots. Symbols show the effect of the menstrual cycle (x-axis) and the standard error of that effect (y-axis, reversed). Each symbol shows an individual study. Vertical solid line shows no effect. Vertical dashed line shows the meta-estimate of effect. Diagonal dashed lines enclose the region expected to include 95% of studies based on the estimated meta-effect and the standard errors. B Results of Egger’s tests for publication bias. Figure S2. Periovulatory meta-analyses. A The log2 difference between periovulatory and follicular phases (log2-pg/mL of the follicular phase minus log2-pg/mL of the periovulatory phase). For TGF-β1, the error bars for one study and the meta-estimate extend off-scale. B The log2 difference between periovulatory and luteal phases (log2-pg/mL of the luteal phase minus log2-pg/mL of the periovulatory phase). For IL-10, the error bars for one study extend off-scale. Each row represents a different immune mediator, with the symbols showing the mean and the lines showing the 95% confidence intervals. Gray symbols indicate individual studies and black the meta-estimates as determined by inverse-variance pooling random effects models. Black filled symbols indicate p < 0.05 while white filled symbols indicate p > 0.05. Positive numbers indicate higher during the follicular or luteal phase, while negative numbers indicate higher during the periovulatory phase. Fig S3. Subgroup analysis: Does the effect of menstrual cycle differ by assay method, geographical region, or method of determining menstrual phase? A Meta-analyses, comparing all studies (black circles) to studies grouped by assay method (ELISA: blue squares; MSD: yellow triangles; Luminex: green diamonds). B Meta-analyses, comparing all studies (black circles) to studies grouped by geographical region of sample origin (Africa: blue diamonds; Europe: red squares; North America: green triangles). C Meta-analyses, compari [file 12916_2022_2532_MOESM5_ESM.zip › figS2R3.tiff]

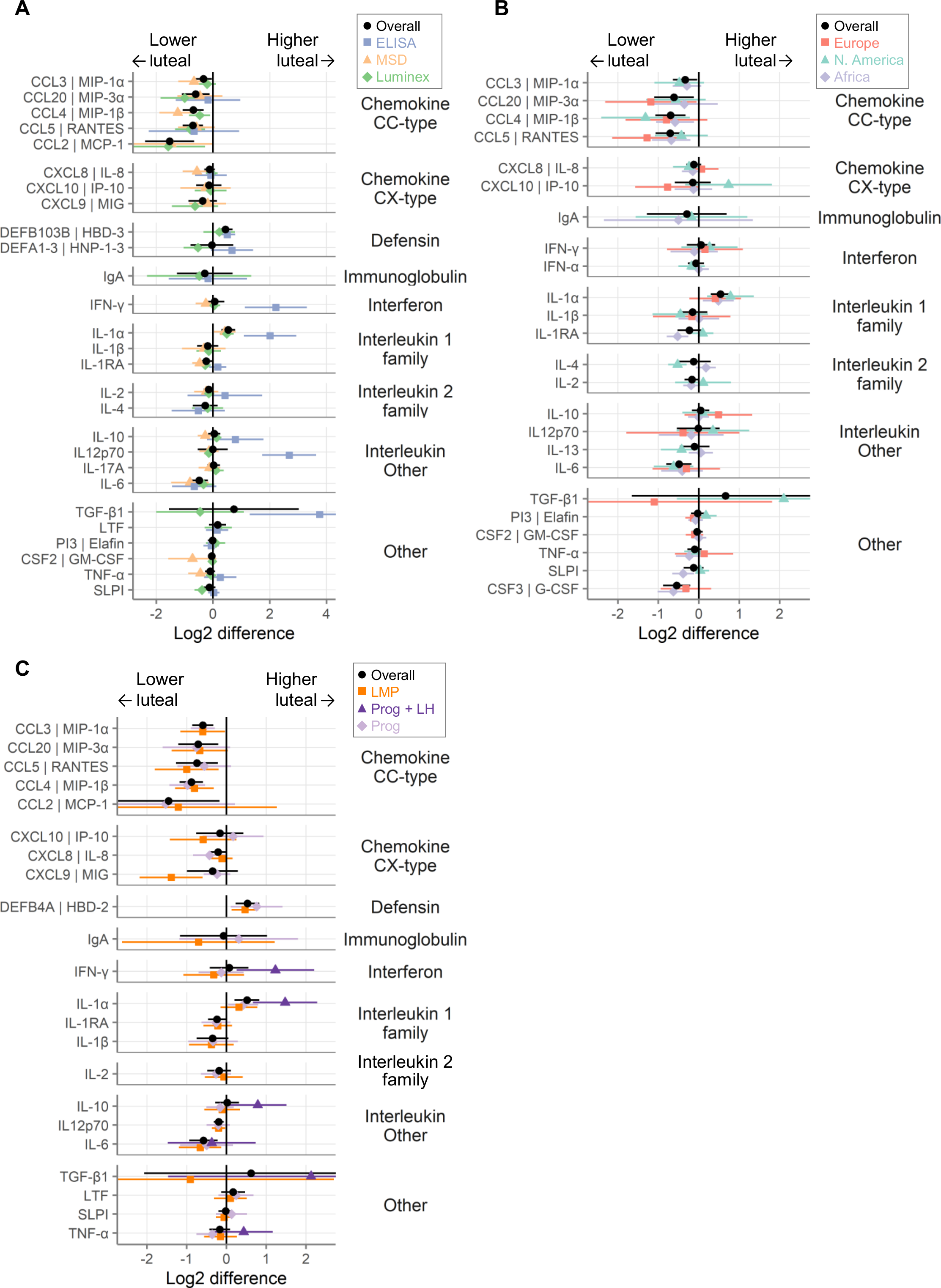

Supplement: Supplementary file 5 — Additional file 5: Figure S1. Assessment of publication bias. A Funnel plots. Symbols show the effect of the menstrual cycle (x-axis) and the standard error of that effect (y-axis, reversed). Each symbol shows an individual study. Vertical solid line shows no effect. Vertical dashed line shows the meta-estimate of effect. Diagonal dashed lines enclose the region expected to include 95% of studies based on the estimated meta-effect and the standard errors. B Results of Egger’s tests for publication bias. Figure S2. Periovulatory meta-analyses. A The log2 difference between periovulatory and follicular phases (log2-pg/mL of the follicular phase minus log2-pg/mL of the periovulatory phase). For TGF-β1, the error bars for one study and the meta-estimate extend off-scale. B The log2 difference between periovulatory and luteal phases (log2-pg/mL of the luteal phase minus log2-pg/mL of the periovulatory phase). For IL-10, the error bars for one study extend off-scale. Each row represents a different immune mediator, with the symbols showing the mean and the lines showing the 95% confidence intervals. Gray symbols indicate individual studies and black the meta-estimates as determined by inverse-variance pooling random effects models. Black filled symbols indicate p < 0.05 while white filled symbols indicate p > 0.05. Positive numbers indicate higher during the follicular or luteal phase, while negative numbers indicate higher during the periovulatory phase. Fig S3. Subgroup analysis: Does the effect of menstrual cycle differ by assay method, geographical region, or method of determining menstrual phase? A Meta-analyses, comparing all studies (black circles) to studies grouped by assay method (ELISA: blue squares; MSD: yellow triangles; Luminex: green diamonds). B Meta-analyses, comparing all studies (black circles) to studies grouped by geographical region of sample origin (Africa: blue diamonds; Europe: red squares; North America: green triangles). C Meta-analyses, compari [file 12916_2022_2532_MOESM5_ESM.zip › figS3R3.tiff]

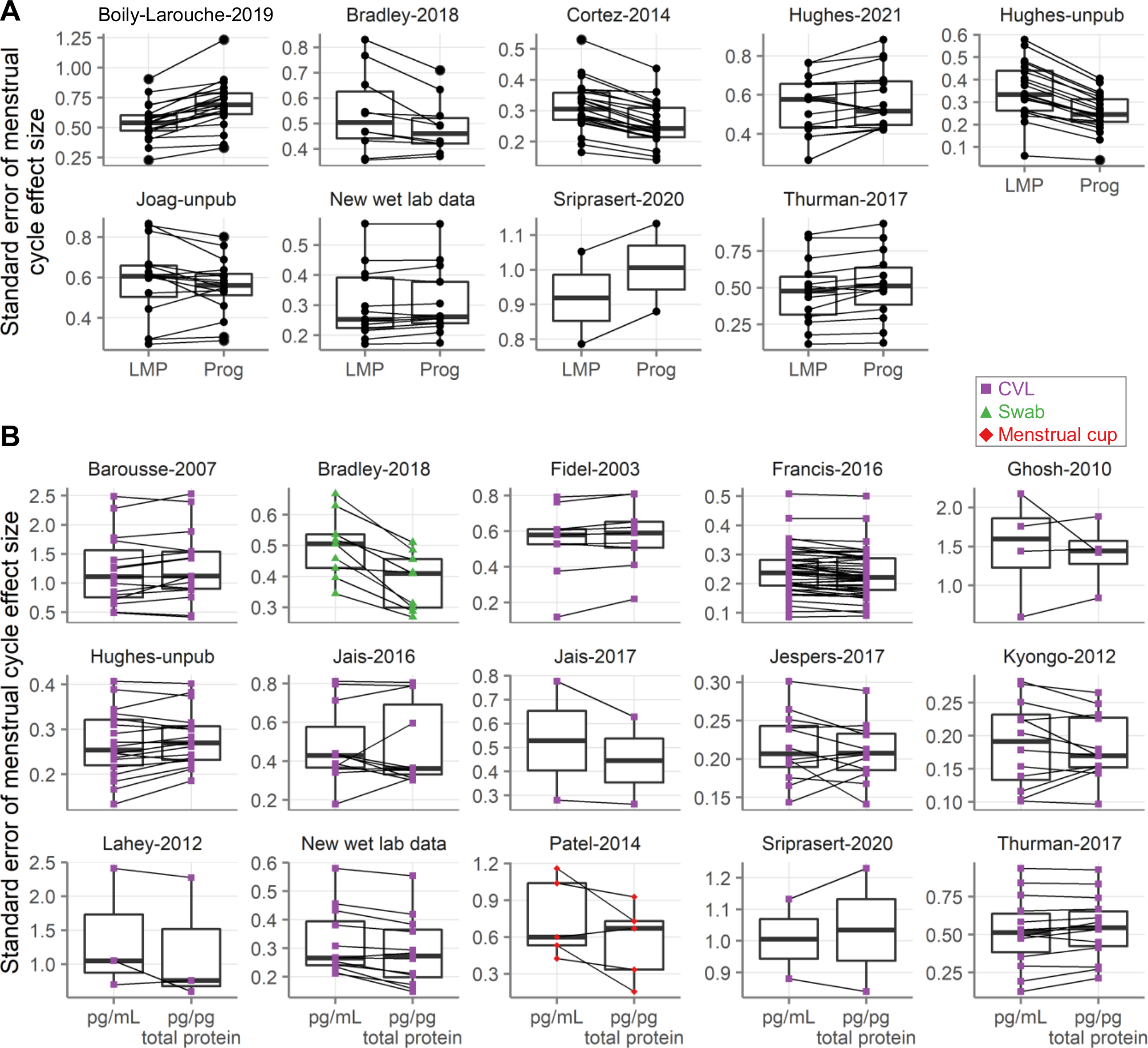

Supplement: Supplementary file 5 — Additional file 5: Figure S1. Assessment of publication bias. A Funnel plots. Symbols show the effect of the menstrual cycle (x-axis) and the standard error of that effect (y-axis, reversed). Each symbol shows an individual study. Vertical solid line shows no effect. Vertical dashed line shows the meta-estimate of effect. Diagonal dashed lines enclose the region expected to include 95% of studies based on the estimated meta-effect and the standard errors. B Results of Egger’s tests for publication bias. Figure S2. Periovulatory meta-analyses. A The log2 difference between periovulatory and follicular phases (log2-pg/mL of the follicular phase minus log2-pg/mL of the periovulatory phase). For TGF-β1, the error bars for one study and the meta-estimate extend off-scale. B The log2 difference between periovulatory and luteal phases (log2-pg/mL of the luteal phase minus log2-pg/mL of the periovulatory phase). For IL-10, the error bars for one study extend off-scale. Each row represents a different immune mediator, with the symbols showing the mean and the lines showing the 95% confidence intervals. Gray symbols indicate individual studies and black the meta-estimates as determined by inverse-variance pooling random effects models. Black filled symbols indicate p < 0.05 while white filled symbols indicate p > 0.05. Positive numbers indicate higher during the follicular or luteal phase, while negative numbers indicate higher during the periovulatory phase. Fig S3. Subgroup analysis: Does the effect of menstrual cycle differ by assay method, geographical region, or method of determining menstrual phase? A Meta-analyses, comparing all studies (black circles) to studies grouped by assay method (ELISA: blue squares; MSD: yellow triangles; Luminex: green diamonds). B Meta-analyses, comparing all studies (black circles) to studies grouped by geographical region of sample origin (Africa: blue diamonds; Europe: red squares; North America: green triangles). C Meta-analyses, compari [file 12916_2022_2532_MOESM5_ESM.zip › figS4R3.tiff]
